# Lead and arsenic intoxications by traditional and alternative medicine: men are more sensitive than women

**DOI:** 10.1007/s00210-024-03317-y

**Published:** 2024-07-27

**Authors:** Lucia Gerke, Roland Seifert

**Affiliations:** Institute of Pharmacology, Hannover Medical School, Carl-Neuberg-Straße 1, D-30625 Hannover, Germany

**Keywords:** Traditional medicine, Alternative medicine, Ayurveda, Metal intoxication, Lead, Arsenic, Blood lead level, Anemia, Basophilic stippling, Chelating agents

## Abstract

Traditional and alternative medicines are widely used around the world and include for example herbal medicine, Ayurveda, traditional Chinese medicine, and indigenous therapies. Due to the long history and the mostly natural origin of traditional remedies, it is often assumed that they are harmless, but in recent decades more and more case reports have been published in which traditional medicine has caused metal poisoning. This paper provides an analysis of published cases in which patients have suffered metal poisoning due to traditional or alternative medicines. A systematic literature search was performed on PubMed, whereby 210 patient cases from a total of 102 case reports and 30 case series were identified and then analyzed about various aspects. Most of the traditional medicines involved come from Asia and are mainly contaminated with lead and arsenic. The analyzed patient cases show a high degree of heterogeneity with regard to age, sex, intake reason, symptoms, and severity of intoxication. The metal intoxication itself and the cause of the poisoning often remained unrecognized for a long time, which resulted in many patients undergoing unnecessary diagnostic methods and ineffective therapeutic approaches before the correct diagnosis was made. The evaluation of the available patient cases revealed a higher sensitivity to metal poisoning in children compared to adults and a higher sensitivity in men compared to women. Anemia and basophilic stippling were frequently observed and became more common as the metal content in the blood increased. Hopefully, this paper raises awareness of the potential dangers of traditional and alternative medicines, both from the patient’s and the doctor’s perspective, so that in case of intoxication, treatment can be initiated quickly using the correct diagnostic methods. As ingested metals do not only circulate in the blood but also accumulate in soft tissues and bones, long-term monitoring is necessary to ensure that patients make a full recovery. Doctors should be aware that, in contrast to common belief, men are more sensitive to this type of intoxication than women, necessitating particular attention for diagnosis and treatment.

**Supplementary Information:**

The online version contains supplementary material available at 10.1007/s00210-024-03317-y.

## Introduction

**Fig. 1:**
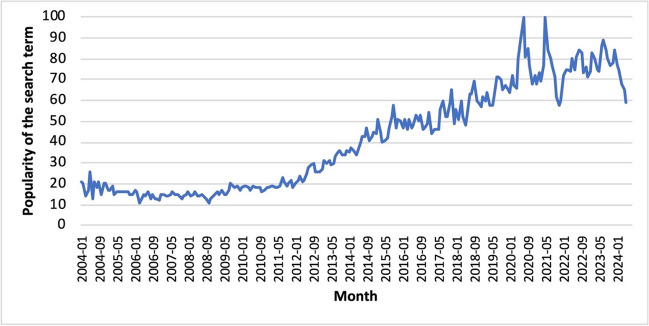
Development over time of Google searches for the term “Ayurvedic Medicine” since 2004 (https://trends.google.de/trends/explore?date=all&q=Ayurvedic%20Medicine&hl=de, accessed May 12, 2024)

On August 17 and 18, 2023, the first global summit on Traditional Medicine hosted by the World Health Organization (WHO) took place in Gandhinagar, India. The WHO states that more than 80% of the world’s population in over 170 of WHO’s 194 member states currently use some kind of traditional medicine, such as herbal medicine, yoga, Ayurveda, acupuncture, and indigenous therapies (World Health Organization (WHO) 2023a). The widespread use and popularity among large parts of the population are also reflected in the number of Google searches. In particular, the search term “Ayurvedic Medicine” has gained popularity in recent years (Fig. 1).

Both alternative and traditional medicine are distinct from conventional medicine. Traditional medicine has a long history and is based on the theories, beliefs, and experiences of various cultures, from which different knowledge, skills, and practices have developed over the centuries. Whether these are explainable or not is irrelevant. Alternative medicine is also referred to as complementary medicine and includes practices that are neither part of conventional medicine nor part of the traditional medicine of the respective country. In some countries, the transition from alternative to traditional medicine is fluid (World Health Organization (WHO) 2019).

WHO’s interest in traditional medicine goes back a long way. It began in 1976 with the establishment of its traditional medicine program. Today, WHO aims to achieve evidence-based integration of traditional medicine (World Health Organization (WHO) 2023b).

**Fig. 2:**
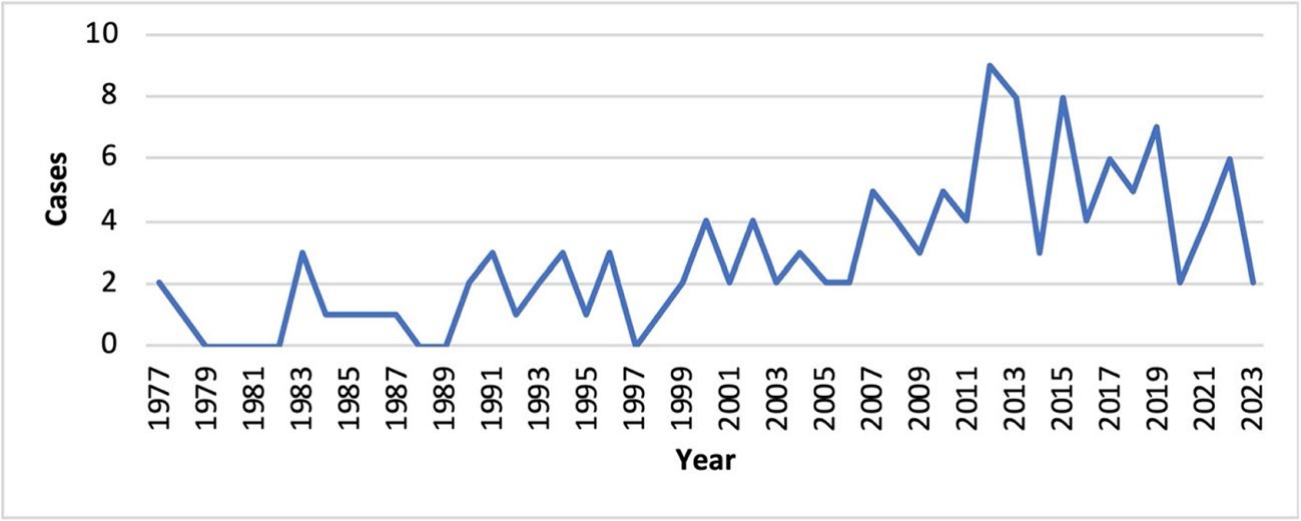
Quantity of published case reports per year (1977–2023)

However, the widespread use of traditional and alternative medicine (T&AM) also has its downsides. Over the years, an increasing number of case reports has been published describing metal intoxication caused by contaminated T&AM (Fig. 2). Particularly in the case of traditional forms of medicine, some of which are based on thousands of years of experience, the question arises as to why such incidents continue to occur. Healthcare systems vary from country to country, and there is often a lack of quality control and standardization of medicines. There are also significant differences in the healthcare workforce and its level of training across countries. For example, a study of healthcare workers in India was published in 2016. According to this study, only 23.3% of healthcare workers in India have medical qualifications, with significant differences between urban areas (29.2% with medical qualifications) and rural areas (14.6% with medical qualifications) (Anand and Fan 2016). This combination of inadequate quality control of medicines and, in some cases, a lack of knowledge among healthcare workers can pose a risk to patients. To get an overview of how widespread metal poisoning by T&AM is, what effects it has, and how the safety of T&AM can be improved, we analyzed published case reports according to various criteria.

## Materials and methods

**Fig. 3:**
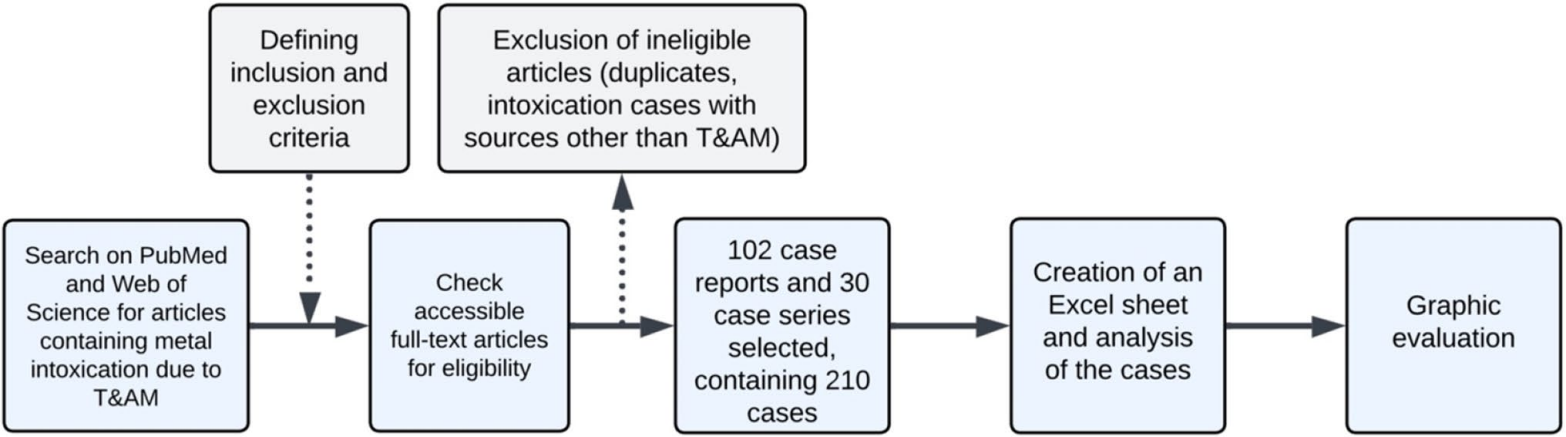
Overview of our method for extracting and analyzing relevant case reports, presented in a flow chart

First, case reports were selected through a systematic literature search on PubMed (https://pubmed.ncbi.nlm.nih.gov). These case reports were evaluated individually, and the references of each case report were checked for further reports. All case reports were then compared with each other and analyzed for various drug- and patient-related aspects (Fig. 3).

### Search strategy

We searched PubMed for case reports and case series that have been published before September 2023. Therefore, we used combinations of different search terms: *metal intoxication, heavy metal contamination, traditional medicine, alternative medicine, herbal medicine, Ayurveda, lead poisoning, arsenic poisoning,* and *mercury poisoning.* After searching PubMed, we also searched the Web of Science for relevant cases. However, this search did not yield any articles that we had not already extracted from PubMed. We did not use Google Scholar as a bibliographic database, as Google Scholar has been criticized for listing predatory journals due to poor quality control (Singh et al. 2022), which we wish to exclude in this review.

### Selection criteria

Only case reports and case series in which the metal poisoning was attributed to the use of T&AM were selected. Cases in which the cause of intoxication was unknown or in which other sources could have been the cause of the poisoning (e.g., poisoning from occupational or environmental sources) were excluded. Cases published in more than one journal were only counted once.

Patient-related criteria such as age, sex, and country of origin did not play a role in the selection process. Missing information in this regard did not lead to the exclusion of the case report. The course of the disease was also not a selection criterion; mild, moderate, and severe as well as asymptomatic cases were included.

In addition to English case reports and case series, papers written in other languages were also included, and there were no language restrictions. Where necessary, these were translated with the help of DeepL (https://www.deepl.com/translator).

### Data analysis

A total of 210 patient cases were collected from 102 case reports and 30 case series (Anderson et al. 2001; Atre et al. 2006; Auyeung et al. 2002; Babu et al. 2012; Bayly et al. 1995; Beigel et al. 1998; Bose et al. 1983; Brearley and Forsythe 1978; Breeher et al. 2013; Breyre and Green-McKenzie 2016; Budnik et al. 2016; Centers for Disease Control and Prevention (CDC) 1983a, b, 1984, 1993, 1999, 2002, 2004, 2012; Chakraborti et al. 2003; Chambial et al. 2017; Chan et al. 1977; Chang et al. 2013; Chen et al. 2021; Choi et al. 2005; Ciocan et al. 2021; Creemers et al. 2008; Datta-Mitra and Ahmed 2015; Deng et al. 2016; Desai and Staszewski 2012; Dolan et al. 1991; Dunbabin et al. 1992; Fernández et al. 2015; Ferson et al. 2022; Garnier and Poupon 2006; Geraldine et al. 2007; Gerdsen et al. 2000; Giampreti et al. 2011; Gitelman et al. 2023; Gopinath et al. 2021; Gulia et al. 2015; Gunturu et al. 2011; Gupta et al. 2011; Hanjani et al. 2007; Hardin et al. 2023; Hochholzer et al. 2014; Horiuchi et al. 2022; Hsiao et al. 2019; Ibrahim and Latif 2002; Jain et al. 2019; Jayachandar and Kotabagi 2010; Jeon et al. 2015; Kanen and Perenboom 2005; Kang et al. 2019; Karri et al. 2008; Karwowski et al. 2017; Keen et al. 1994; Kew et al. 1993; Khandpur et al. 2008; Kim et al. 2012, 2013; Kulshrestha 1996; Kumar et al. 2010; Lee et al. 2010, 2004; Leiba et al. 2010; Levit et al. 2007; Li et al. 2000; Lightfoote et al. 1977; Lim et al. 2019; Lin et al. 2012a, b; Ma et al. 2022; Madan et al. 2007; Madhusudhanan and Lall 2007; Mahdi et al. 2020; Markowitz 1994; Mathee et al. 2015; McElvaine et al. 1990; Meiman et al. 2015; Mitchell-Heggs et al. 1990; Moore and Adler 2000; Moorthy et al. 2018; Muller et al. 2013; Muzi et al. 2001; Orchard et al. 2015; Perharic et al. 1994; Pham and Sharma 2023; Philips et al. 2018, 2022; Pierce et al. 2012; Pinto et al. 2014; Pontifex and Garg 1985; Prakash et al. 2009; Prpić-Majić et al. 1996; Rahman et al. 1986; Raut et al. 2021; Raviraja et al. 2008, Raviraja et al. 2010; Sadler and Bell 2017; Saryan 1991; Sathe et al. 2013; Schilling et al. 2004; Senthilkumaran et al. 2017; Shamshirsaz et al. 2009; Shinde et al. 2022; Siefring et al. 2018; Singh et al. 2009; Smitherman and Harber 1991; Soni and Dayal 2019; Spilchuk and Thompson 2019; Spriewald et al. 1999; Tait et al. 2002; Tang et al. 2017; Toniolo et al. 2011; Tsai et al. 2017; Tsitsikas et al. 2012; Tsutsui et al. 2013; Vonderen et al. 2000; Weide et al. 2003; Wijeratne et al. 2011; Woolf et al. 2008; Wu and Deng 2013; Wu et al. 1996, 2013; Yanamandra et al. 2020; Ying et al. 2016, 2018; Zhao and Lv 2018; Zheng et al. 2019; Zhou et al. 2015; Zhu and Zheng 2012). A detailed documentation of all raw data is provided in Supplementary Figs. S1-S21 and Tables S1 and S2. The cases are documented in Tables S2A-S2F.

The aim of this paper is to summarize all available data on these cases of metal intoxications associated with T&AM. The following information was extracted from each of the 210 patient cases: age and sex of the patient, reasons for taking the remedy, intake duration and amount of metal ingested, laboratory results (e.g., metal concentration in blood), therapy, and clinical outcome. In addition, information was collected on the remedy that was taken and its origin. Not all of the above-mentioned information was always given in each report. If some information was missing, the category of the case report was marked as “data not available” (N/A) and excluded from the analysis of the corresponding criterion, but the case report was not completely excluded from this review.

Finally, we looked at whether there were any correlations between the different criteria. It was investigated whether age and sex had an influence on the severity of poisoning and whether there was a linear relationship between the amount of metal ingested and the course of the disease. It was also investigated whether certain laboratory parameters became more noticeable with increasing metal levels in the blood.

## Results and discussion

### Publication year and publication country

The first extracted case reports date back to 1977 (Fig. 2). Up to and including 1999, only a few case reports were published (zero to three per year). Since 2000, there has been a clear growth trend with two to nine case reports per year. From 1977 to 1999, an average of 1.22 case reports were published per year, and from 2000 to 2023, the publication volume increased to an average of 4.33 published case reports per year. In 2020, there was a decrease in the number of published cases. This can be attributed to the COVID-19 pandemic, which severely restricted both international trade and tourism (Škare et al. 2021; Xiang 2023), as 35.2% of the drugs were imported prior to use and 19.5% of the patients obtained the drugs while traveling abroad.

The majority of the case reports come from Asia (47.7%), North America (25%), and Europe (19.7%), while only 6.8% come from Oceania, 0.8% from Africa, and no case reports come from South America (Fig. S1A). A particularly large number of reports have been published in the USA (22%), India (20.5%), and China (11.4%), which together account for more than 50% of the 132 reports (Fig. S1B).

### Patient characteristics: age, sex, and ethnicity

**Fig. 4:**
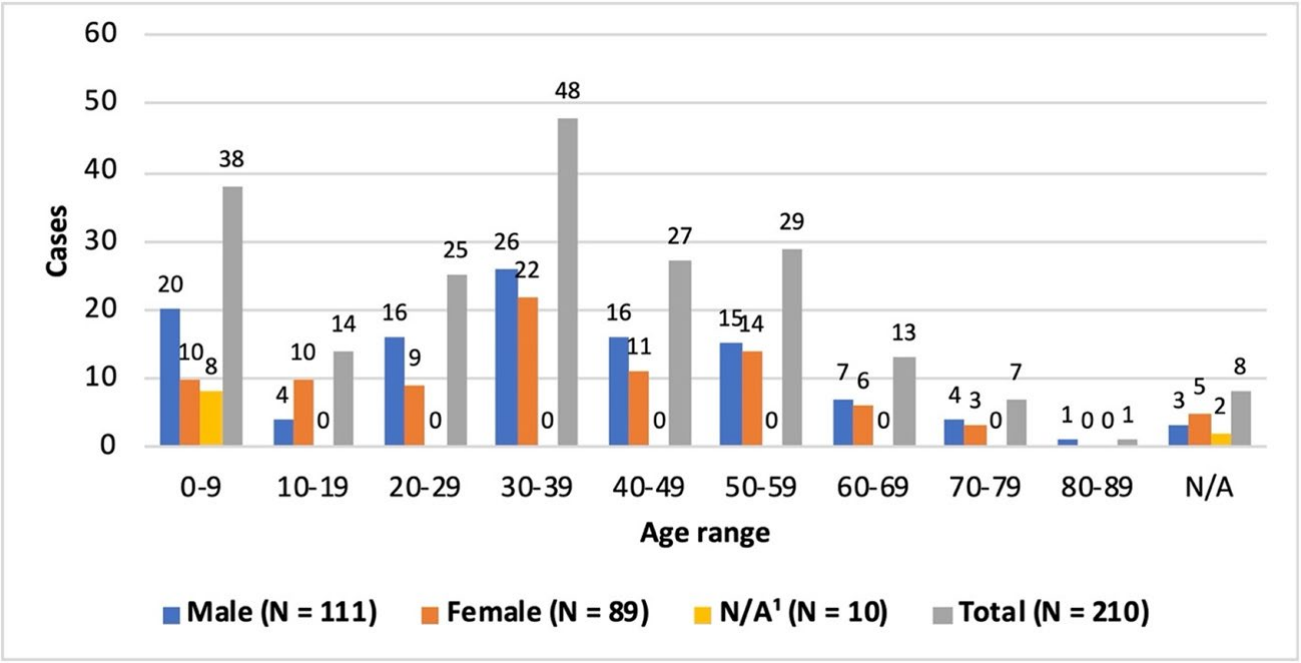
Sex-related age distribution—Classification into age groups of 10 years each and comparison within the groups by sex. Patients with unknown age and/or sex are reported as N/A

Of the 210 patients, 111 are male (52.9%), 89 are female (42.4%), and the sex of 10 patients—mostly neonates and infants—is unknown (4.8%, Fig. S2). The age distribution is broad, with patients ranging in age from 1 day to 80 years, but there are two clear age peaks and the number of patients decreases significantly from the age of 60 (Fig. 4). The first age peak ranges from one day to 9 years, with 43% of the group of patients being younger than 12 months. This can be attributed to the intention of parents to provide beneficial care for their children. Due to their young age, many of these patients were not able to express any complaints themselves or it was difficult to classify these complaints correctly. The second age peak includes patients between the ages of 30 and 39. Many patients in this age group are concerned about anti-aging, and want to improve their appearance or increase their fertility.

The socioeconomic status of the patients was not analyzed in the case reports. The ethnicity of the patients was also often barely mentioned or not mentioned at all. In 133 cases (63.3%), no information on ethnicity was provided. In 77 cases (36.7%), the ethnic background was mentioned, although in some cases only the continent (e.g., Asian) and not the country of origin or the exact ethnic group was mentioned. Of the 77 cases, 81.8% were of Asian descent, 9.1% were South American, 7.8% European, and only 1.3% African.

### Kind of medication and metal contamination

**Fig. 5:**
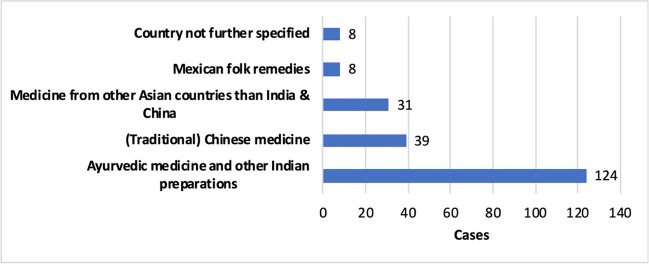
Specification of the T&AM used—Classification of drugs by country of origin. In addition to Ayurvedic medicine, Indian drugs also include other Indian preparations for which it is not certain whether they can be classified as Ayurvedic drugs. The Mexican folk remedies are known as Azarcón and Greta.

Ayurvedic medicine, which originated in India, and traditional Chinese medicine are particularly well known (Bodeker et al. 2005). This is reflected in the medicines used in the case reports (Fig. 5). There, 124 patients (59.1%) used Ayurvedic or other Indian preparations and 39 patients (18.6%) took traditional Chinese preparations. Another 31 patients (14.8%) chose T&AM from other Asian countries. Only eight patient cases (3.8%) involved non-Asian medicines, which were Mexican folk remedies. 83.5% of medications were taken orally, 3.4% were applied dermally, and 1.3% were applied nasally (Fig. S3).

**Fig. 6:**
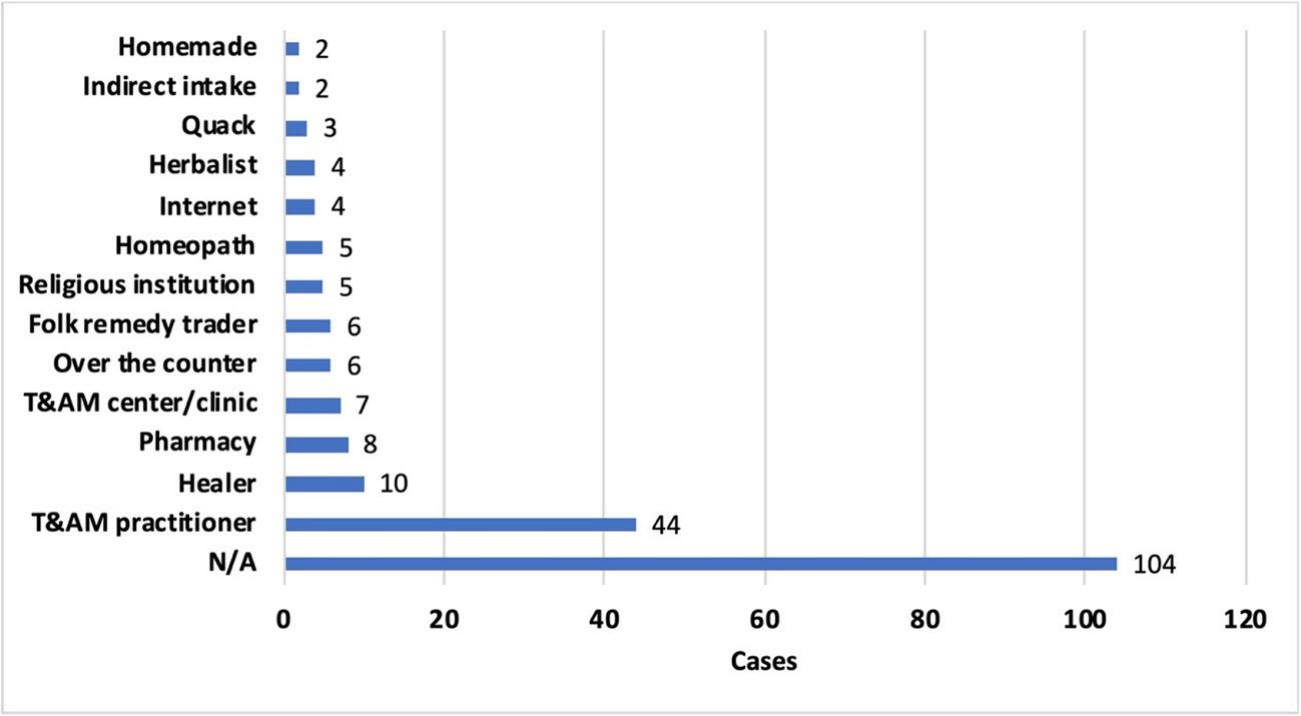
Obtaining of the medication—Who or where did the patients turn to in order to acquire T&AM? Some patients obtained the T&AM without medical consultation, buying it over the counter or from traders, ordering it over the Internet, or making it themselves. But even in cases of consultation, the designation of the person or institution consulted does not necessarily indicate the level of medical qualification.

Where or from whom the patients obtained the T&AM preparations is not known in 49.5% of the cases. In the other 50.5% of cases, the source of procurement varied (Fig. 6). In the cases where the source was mentioned, most patients contacted people who described themselves as traditional medicine practitioners or doctors. However, this designation does not indicate the education level or medical qualification. A WHO study of the health workforce in India shows that only 45% of all doctors in India have a medical degree; 30.8% have at most a secondary school education. On average, health workers from rural areas have lower qualifications than those from urban regions (Anand and Fan 2016). Folk healers with unknown medical qualifications, pharmacies and clinics, or even non-medical facilities such as supermarkets or religious institutions were also frequently visited. Parents rarely purchased medicines for their children from practitioners or pharmacies, but rather from unqualified sources such as folk remedy traders or religious institution (Fig. S4). Ideally, prescribers should have undergone qualified training before prescribing T&AM, but this is difficult to achieve, especially in rural and educationally disadvantaged areas. A general ban on prescribing T&AM without qualified training would deny a large proportion of the population access to T&AM, while access to allopathic medicines is also often unavailable in these areas (Bodeker et al. 2005).

WHO research has shown that only a small proportion of the countries in which T&AM is sold and used have national policies to regulate such medicines (World Health Organization (WHO) 2005). This leads to a lack of control over the ingredients, production, and sale of T&AM in many areas. Due to the lack of quality control, some T&AM may be contaminated with metals, whereby different causes of contamination must be distinguished. Depending on the medicine, metal contamination can be unintentional, e.g., for environmental reasons, or intentional. One spectrum of Ayurveda, the “Rasashastra”, includes medicines to which metals are intentionally added, mostly lead, gold, iron, mercury, arsenic, copper, zinc, and silver. It is believed that these metals have their own therapeutic benefits and also help to enhance the effect of the medicine. According to ancient Ayurvedic texts, the metals should be purified and oxidized beforehand and have a certain dosage so that they are safe to take (Gogtay et al. 2002).

**Fig. 7:**
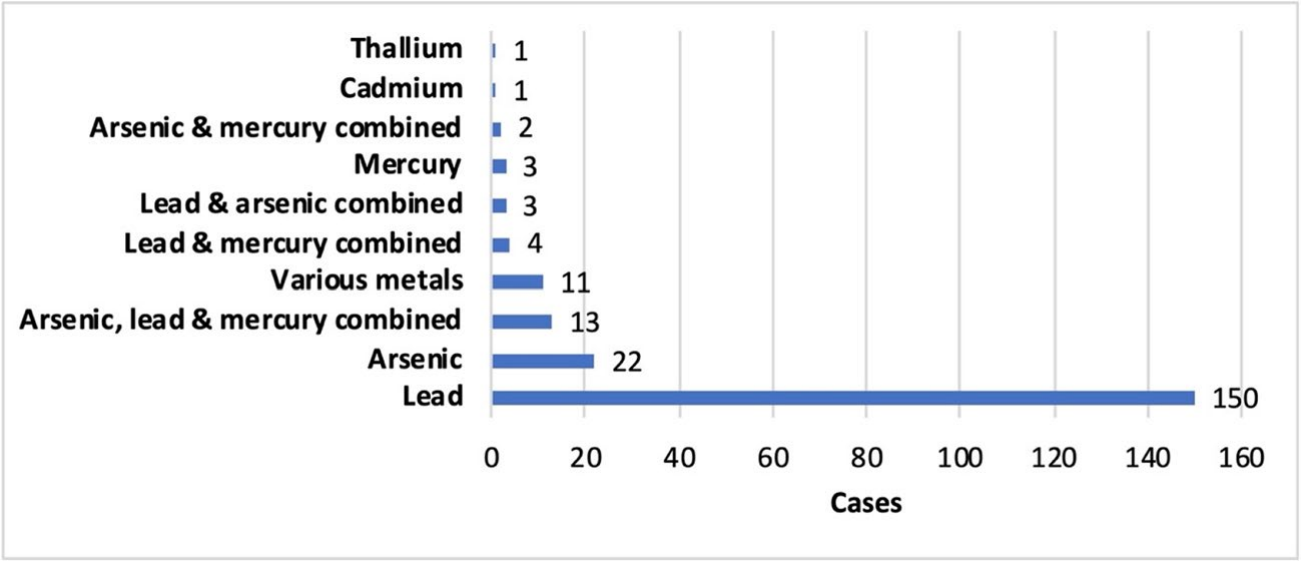
Overview of the metals with which the T&AM were contaminated in the available case reports. Some T&AM were contaminated with more than one metal. This graph provides only an overview of the metals present and does not indicate any information about the metal concentration. Not all metals were present in the same concentration. Some metals were only detected at low levels.

In the majority of the case reports analyzed, the metal poisoning was due to lead (74.2%, Fig. 7); 10.3% of the patients suffered from arsenic poisoning. In 15.7% of the cases, medications were taken that were contaminated with two or more metals, with mercury often being involved in addition to lead and arsenic. Poisoning by mercury alone occurred only sporadically (1.4%), as did poisoning by cadmium (0.5%) or thallium (0.5%).

In the cases of poisoning with only one metal, this metal was mentioned in the title of the case report in 95.9% of the cases. In cases of poisoning with more than one metal, only 7.9% of the cases mentioned all metals in the title. In 65.8%, only lead was mentioned in the title, while arsenic and mercury were not mentioned in more than 55% of the titles, despite high concentrations in some cases. There was also no mention of other metals in the titles, as these were only present in small amounts in the drugs.

The form in which the metals were present was not mentioned in 84.3% of the cases. In the cases where the form was mentioned, it was almost exclusively inorganic compounds, mostly in the form of oxides or sulfides (e.g., lead tetroxides and arsenic disulfides). Only in one case was a metal present in organic form.

### Reasons for intake and their average age

**Fig. 8:**
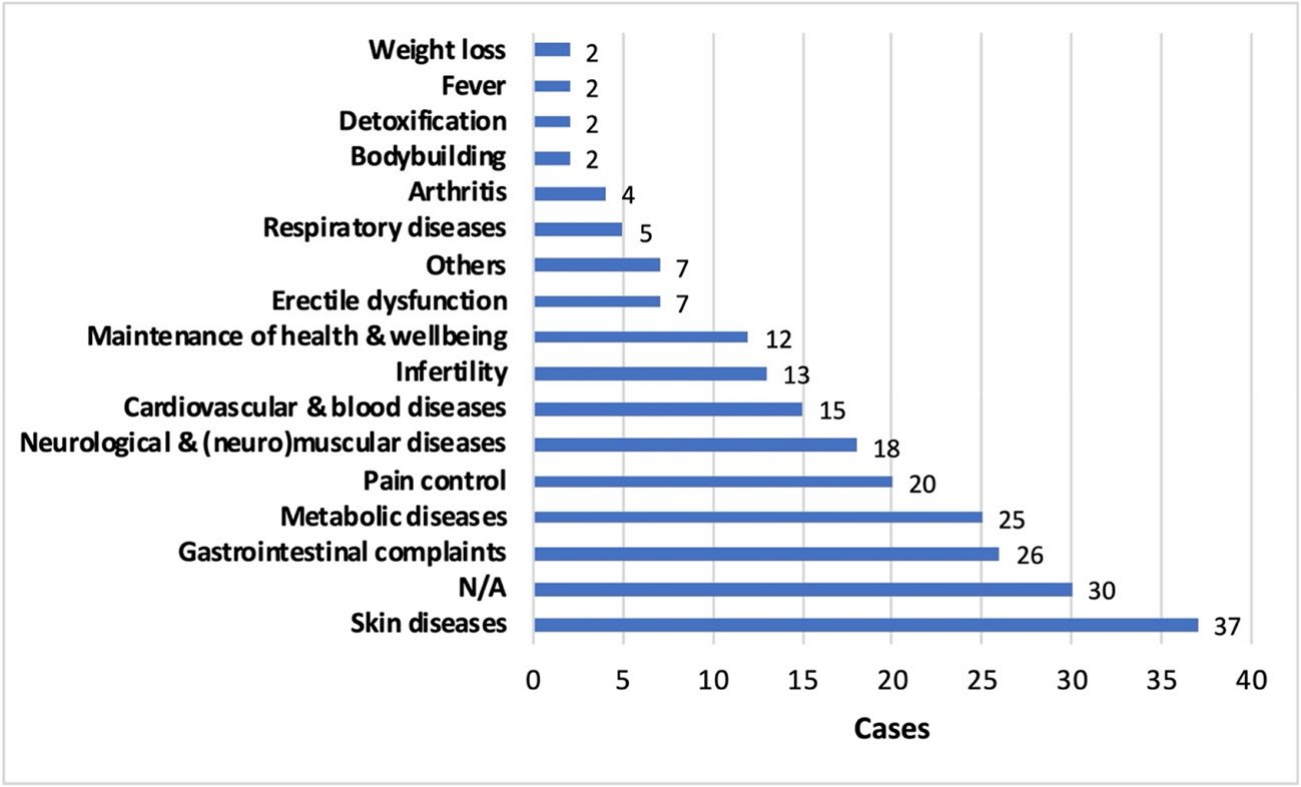
Intake reasons for T&AM by main categories, each of which includes several intake reasons. The following reasons for intake were the most common in each category: Skin diseases: acne, dermatitis, psoriasis; Gastrointestinal complaints: abdominal colic, indigestion; Metabolic diseases: diabetes; Pain control: back pain, angina pectoris; Neurological and (neuro)muscular diseases: developmental delay, seizures, weakness; Cardiovascular and blood diseases: hypertension, atherosclerosis prevention; Respiratory diseases: rhinitis

In 30 cases (13.2%), no reason was given for taking T&AM, whereas in some cases more than one reason was given per person (Fig. 8). The most common reasons for taking T&AM were skin diseases (16.3%), gastrointestinal complaints (11.5%), metabolic disorders (11%), pain (8.8%), and neurological and (neuro)muscular diseases (7.9%). The wide range of reasons why patients take T&AM shows that although T&AM is more commonly used for certain reasons, it is basically used for all possible diseases or conditions. There are no specific conditions that are more likely to be treated with T&AM. Therefore, certain pre-existing conditions alone do not indicate whether T&AM has been taken.

Two patients were affected by indirect intake, as the mothers suffered from lead poisoning during pregnancy due to ingestion of T&AM. Since lead can cross the placental barrier (Rísová 2019), the fetuses were already exposed in utero and tested positive for elevated blood lead levels (BLL) postpartum.

**Fig. 9:**
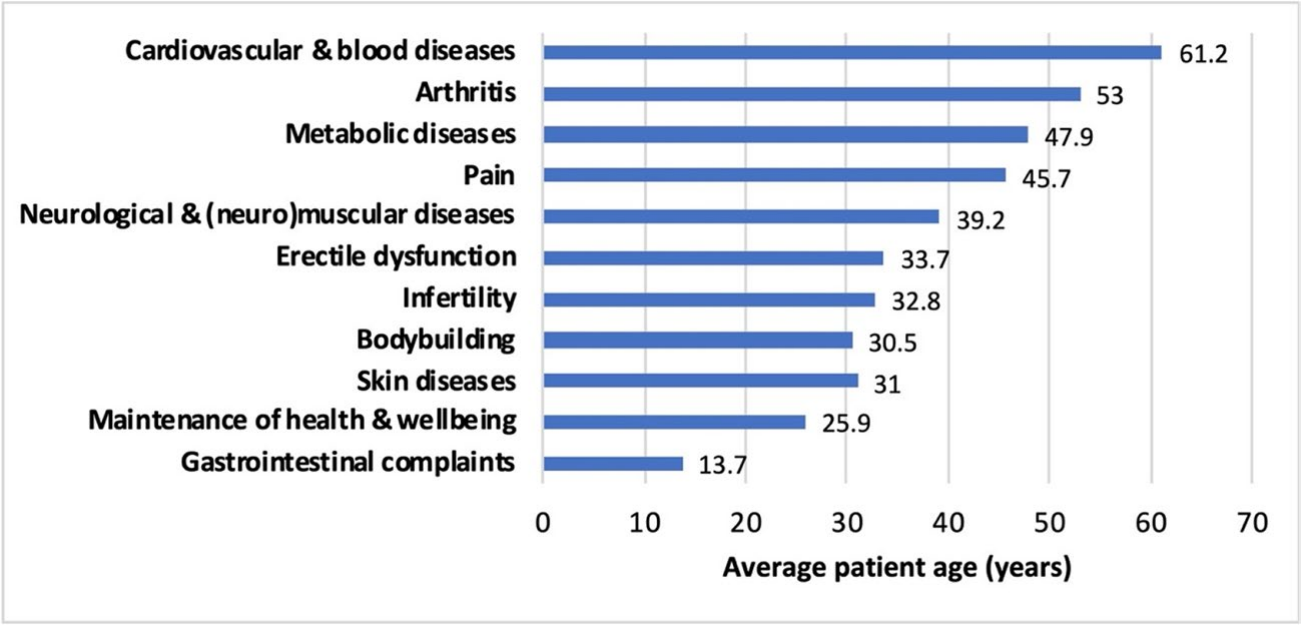
Average patient age for selected intake reasons—We divided the age of all patients who reported a reason for taking T&AM in a particular category by the number of patients in that category.

The lowest average age by far is for gastrointestinal complaints (ø 13.7 years), as the patients here were often only a few weeks or months old. In these cases, the parents probably wanted to promote the child’s intestinal health or they suspected gastrointestinal complaints as the cause of frequent crying. On the one hand, patients under the age of 35 often gave reasons for taking T&AM that affected their external appearance, for example skin diseases (ø 31 years, e.g., acne) or muscle growth (ø 30.5 years). On the other hand, infertility and erectile dysfunction are central reasons for use in this age group (ø 32.8 and 33.7 years). This can be explained by the fact that at younger ages, distress is often caused by dissatisfaction with physical appearance, unfulfilled desire for children, and sexual dysfunction. At older ages, typical age-related illnesses such as arthritis (ø 53 years) and cardiovascular diseases (ø 61.2 years) become more frequent reasons for taking T&AM (Fig. 9).

### Duration of the medication use and amount of metals ingested

**Fig. 10:**
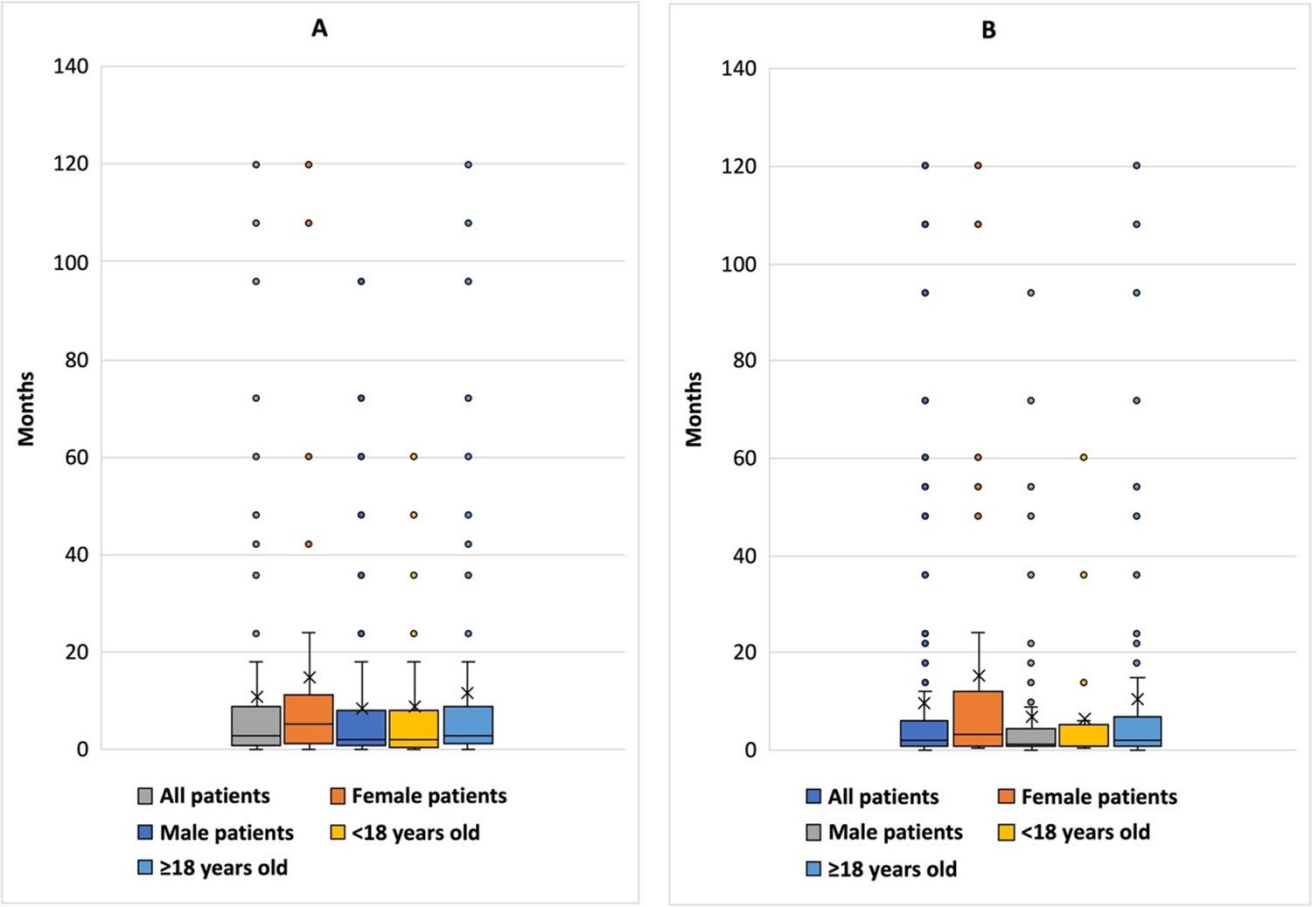
Sex- and age-related comparison of intake duration (**A**) and time from first use of T&AM to first symptoms of intoxication (latency, **B**) using box plots; 10A: the following patients were excluded from these box plots: patients with unknown or unclear intake duration (*N* = 65), and patients with only indirect intake (*N* = 2); 10B: the following patients were excluded from these box plots: patients with unknown latency period (*N* = 72), patients whose first symptoms appeared only after cessation of the medication (*N* = 7), patients with only indirect intake (*N* = 2), and asymptomatic patients (*N* = 19); there is no separate box plot for patients whose sex is unknown

Box plots were used to compare different groups of patients with regard to the duration of T&AM use (Fig. 10A). In 31% of cases, no exact intake duration was reported, so these cases are not included in the box plots. Women took the medication for an average of 14.74 months, while men took it for an average of only 8.68 months. Patients over the age of 18 took the medication for an average of 2.8 months longer than underage patients.

Forty-five percent of patients with an asymptomatic course took their medication for 1 to 6 months. Fifteen percent took the drug for more than 6 months and another 15% for more than 12 months without any symptoms of intoxication occurring. The time between the first intake of T&AM and the onset of symptoms varies widely (Fig. 10B). Some patients have used T&AM for years before experiencing symptoms of intoxication. On the other hand, there are many patients in whom symptoms appeared after only a few days or weeks. In men, the average time to onset of symptoms was 6.94 months, while in women it was 15.07 months. Underage patients also had symptoms of intoxication significantly earlier (ø6.35 months) than adult patients (ø10.38 months). In 3.3% of patients, symptoms of intoxication appeared later, after the drug had already been discontinued. After the onset of symptoms, 23.3% of patients immediately consulted a physician; 29.1% of patients continued to take their T&AM despite symptoms and did not suspect the drug to be the cause of their symptoms. On average, these patients continued to take the T&AM for 1.46 months after the onset of symptoms (women 1.47 months, men 1.45 months). Adolescent patients discontinued the medication sooner than adult patients, after an average of 1.04 months. This may be because parents react more sensitively to changes and symptoms in their children than adults do in themselves.

In the majority of cases, the metal concentration in the medication and the amount ingested were not reported, or the information was incomplete, so that the actual daily and total amount ingested could not be determined in most cases. In the cases of lead intoxication, 43 case reports mentioned how much lead the patients had ingested. For arsenic intoxication, only two case reports provided information on the amount ingested.

When comparing women and men, men consumed more lead per day on average (112.46 mg) than women (102.5 mg, Fig. S5A), but women consumed a significantly higher mean total amount of lead (25.24 g) than men (12.03 g) due to their longer average intake period (Fig. S5B). Two patients knowingly exceeded the recommended daily dosage; it can be assumed that all other patients adhered to the recommended dosage, at least there was no information to the contrary in the case reports.

In some cases where the metal content of T&AM has been measured, it has been found that the metal content within a preparation can vary considerably from tablet to tablet. This is presumably due to the fact that there are no legally required standardized manufacturing processes for T&AM. Therefore, if only one tablet is analyzed in a given case, it is possible that the metal content of this one tablet is significantly lower or higher than in the other tablets, which in turn leads to a falsification of the extrapolated amount of metal ingested.

### Clinical symptoms

**Fig. 11:**
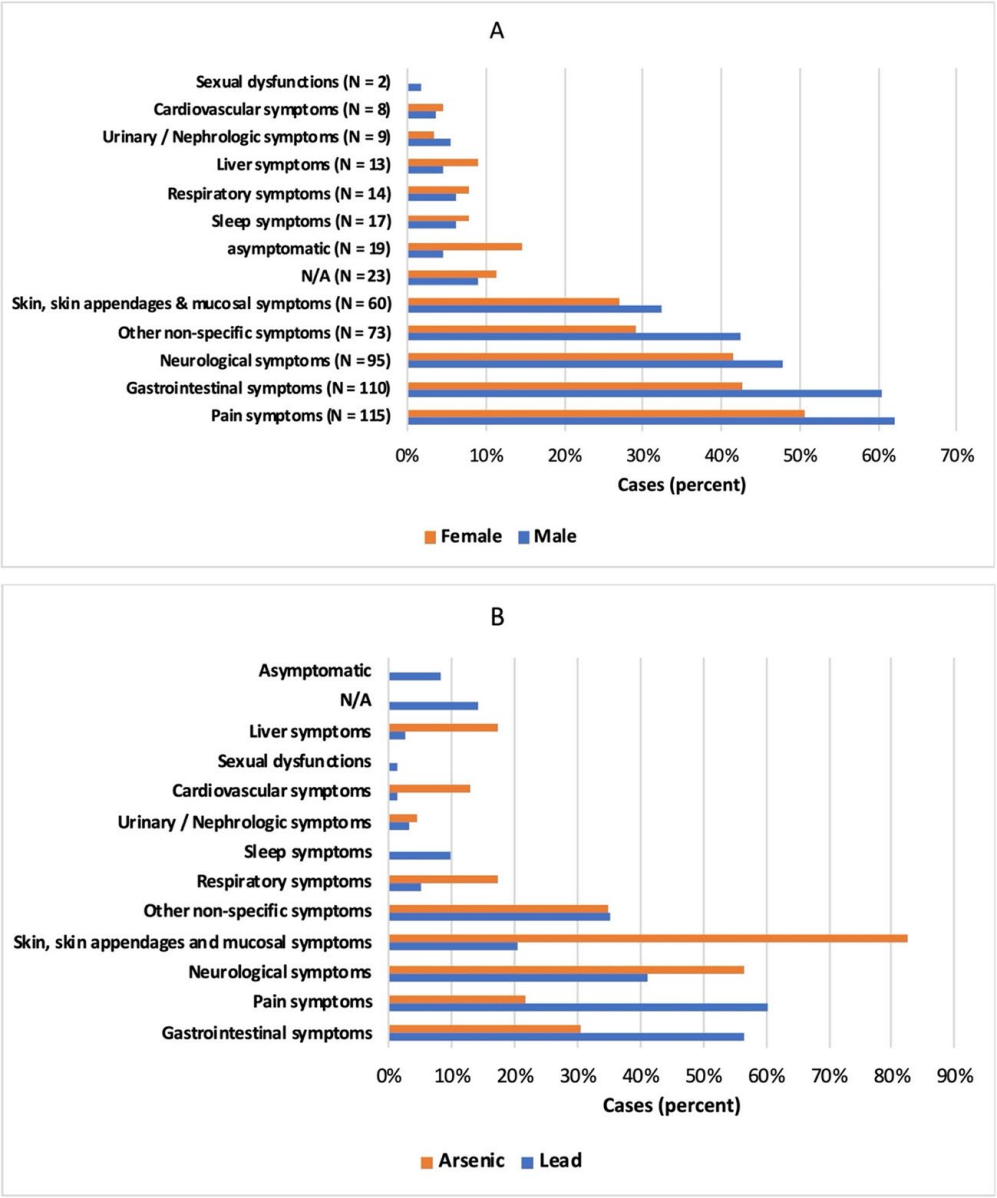
Differences in the frequency of various symptoms depending on sex (**A**) and the metal ingested (**B**); 11A: patients with unknown sex were excluded from this figure; 11B: this figure includes only cases of lead or arsenic poisoning and excludes poisoning with other or multiple metals

Clinical symptoms caused by metal poisoning were widespread; 9.1% of the patients were asymptomatic, and in 11% of the cases no information on symptoms was provided. By far the most common symptoms were pain, gastrointestinal complaints, neurological symptoms, other non-specific symptoms (e.g., fever, malaise, and weight loss), and symptoms affecting the skin, skin appendages, or mucous membranes. Within each symptom category, we calculated how many men out of all male patients and how many women out of all female patients were affected by a symptom in that symptom category (Fig. 11A). Since this figure is a specific comparison between women and men, the symptoms of the 10 patients with unknown sex are not included in this figure. Overall, it is noticeable that men had more symptoms on average than women. The symptom categories that affected women more frequently than men on average were all categories with a comparatively low overall prevalence (e.g., respiratory symptoms). In addition, women were more likely to be asymptomatic (14.6%) than men (4.5%). Comparing lead and arsenic poisoning, it is noticeable that some symptoms are typical of poisoning with each metal (Fig. 11B). The graphical representation of all subcategorical symptoms is shown in the Supplementary Figures S6A-S6K.

Gastrointestinal symptoms occurred in 56.4% of lead poisoning cases, but only in 30.4% of all arsenic poisoning cases. Pain was also more common in lead poisoning (60.3%) than in arsenic poisoning (21.7%). Symptoms of the skin, skin appendages, or mucous membranes occurred in 82.6% of arsenic poisonings, but only in 20.5% of lead poisonings. Neurological symptoms were also slightly more common in arsenic poisoning (56.5%) than in lead poisoning (41%).

Lead-associated encephalopathy was observed in 5.2% of all patients. Among minors, 12.5% had a lead encephalopathy, compared with only 3.1% of adults. The minor patients with encephalopathy were without exception very young neonates or infants ranging in age from a few days to a maximum of 3 years. The central nervous system of children is more susceptible to metals due to incomplete development, making them more vulnerable to lead encephalopathy (Needleman 2004).

### Laboratory findings

When metal poisoning is suspected, the level of metal in the patient’s blood should be measured. For example, the blood lead level indicates the concentration of lead in the blood. There is no general limit below which lead in the blood is safe. In fact, it is now assumed that even the lowest concentrations of lead can cause damage, so the BLL should ideally be as close to 0.00 μmol/l as possible and not exceed a maximum of 0.48 μmol/l (Advisory Committee on Childhood Lead Poisoning Prevention of the Centers for Disease Control and Prevention 2012; Canfield et al. 2005; Patrick 2006). Approximately two thirds of lead poisoning case reports cite a supposedly normal range for BLL, although in most cases no source for this reference range was given. Looking at the BLL reference values given in the case reports, there is a downward trend over the years. Before 1990, the recommendation was a maximum value of 2–3 μmol/l, and between 1990 and 2000 it was around 0.5–1.5 μmol/l. In general, since the turn of the century, reference values have been reported much more frequently in case reports than before, with a mean of 0.75 μmol/l and a median of 0.48 μmol/l, indicating that higher BLLs were considered normal in the past (Fig. S7). Reference values for the arsenic content in the blood were reported in only five case reports, ranging from 0.24 to 0.82 μmol/l.

**Fig. 12:**
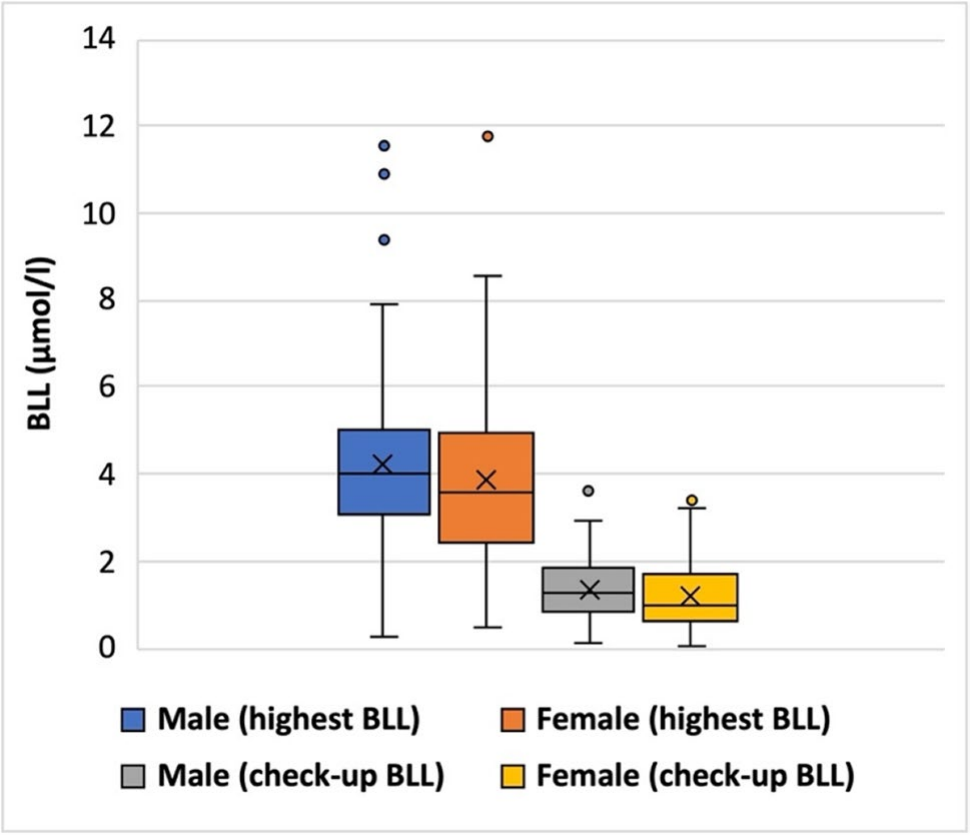
BLL—Sex comparison (highest measured BLL and BLL measured at the last check-up); the following patients were excluded from these box plots: patients with unknown BLL (*N* = 7), and patients with unknown sex (*N* = 10); there is no separate box plot for patients whose sex is unknown; since the BLL refers only to lead, these box plots include only patients who consumed lead-contaminated T&AM

The patients’ BLLs range from 0.29 to 11.8 μmol/l. When the highest BLLs measured in males and females are compared using a box plot (Fig. 12), it shows that males have a higher mean BLL (4.24 μmol/l) than females (3.85 μmol/l). The median BLL is also higher in men than in women (4.02 versus 3.6 μmol/l). Minors have a higher mean BLL (4.26 μmol/l) than adults (4.01 μmol/l, Fig. S8), which may be due to the fact that children’s intestines absorb metals more easily than those of adults (Needleman 2004).

In principle, a correlation between the BLL and the amount of lead ingested is conceivable, but in our evaluations, no linear relationship was found between the amount/duration of ingestion and blood metal levels, which may be related to the heterogeneity of the cases. Comparison is difficult because all patients took different medications in different ways. In addition, the metals contained in the medication may be present in different forms, e.g., organic or inorganic, and therefore have different absorption rates. In a case series in which seven patients were taking the same contaminated medication and therefore the general conditions were much more uniform, it was found that the higher the amount of lead ingested, the higher the BLL (Lim et al. 2019).

Blood arsenic levels were measured in 16 patients and ranged from 0.09 to 23.4 μmol/l (Fig. S9). By far the highest values, 16.29 and 23.4 μmol/l, were measured post-mortem in patients who died of arsenic poisoning. The patient with the third highest measured blood arsenic level of 4.39 μmol/l survived and had a normal blood arsenic level again after 6 months.

In addition to the metal content, the patients’ blood can be tested for other parameters, including the presence of anemia and basophilic stippling of the erythrocytes. Lead has a strong binding capacity for sulfhydryl groups and can therefore interfere with enzymes and structural proteins (Needleman 2004). Within the heme synthesis pathway, lead can inhibit δ-aminolevulinic acid dehydratase (δ-ALAD) and ferrochelatase. This leads to an accumulation of δ-aminolevulinic acid (δ-ALA) and porphyrins in the blood (Ahamed and Siddiqui 2007). In the present case reports, elevated levels of δ-ALA and various porphyrins (mainly zinc protoporphyrins, coproporphyrins, and free erythrocyte protoporphyrins) were measured in 19.5%. In 5.7% no elevated levels were found, and in 74.8% it was not stated whether these levels were measured. Inhibition of the enzymes involved in heme synthesis can lead to anemia. In addition to the enzymes involved in heme synthesis, lead also inhibits erythrocyte pyrimidine-5′-nucleotidase, which in turn is associated with basophilic stippling. Erythrocytes with basophilic stippling contain many basophilic granules that are filled with aggregated ribosomes and are signs of impaired erythropoiesis (Cheson et al. 1984). Anemia was diagnosed in more than every second patient (57.3%) and basophilic stippling in more than every third patient (33.8%). Anemia was excluded in only 9.9% and basophilic stippling in 14.8%. In almost one third of the cases (32.9%), no information was provided on the examination for anemia, and in 51.4% of the cases, no information was provided on basophilic stippling, so it remains unclear how many patients actually had anemia and/or basophilic stippling (Fig. S10). In 62.8% of the confirmed anemias, the anemia was further specified. The most common anemias were normo- and microcytosis as well as normo- and hypochromia, with normochromia usually associated with normocytosis and hypochromia usually associated with microcytosis. Macrocytosis was observed in only two cases and hyperchromia in none (Fig. S11). Hemolysis was seen in 10 cases. In addition to erythrocyte abnormalities, abnormalities of other cell types were noted in some cases. Leukopenia or leukocytosis and thrombocytopenia or thrombocytosis were occasionally observed.

**Fig. 13:**
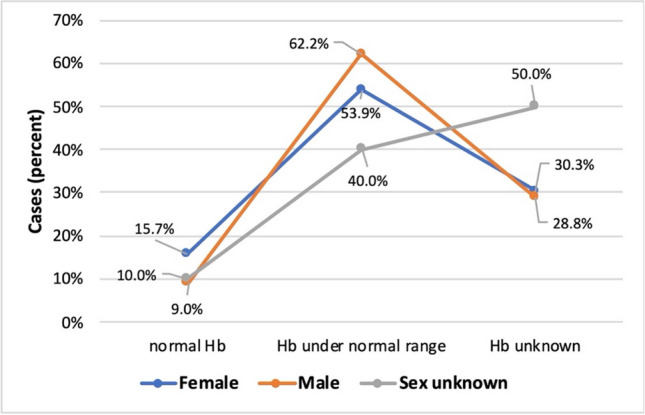
Relationship between normal and low hemoglobin levels by sex in the form of a line chart, where the orange line represents female patients and the blue line represents male patients. The gray line represents patients whose sex is unknown. The percentages indicate the number of patients with normal, low, or unknown Hb compared by sex.

Among women, 15.7% had a normal hemoglobin (Hb) level, compared with only 9% of men. Instead, more men (62.2%) than women (53.9%) had low Hb levels, suggesting that men are more likely to develop metal-associated anemia than women (Fig. 13).

**Fig. 14:**
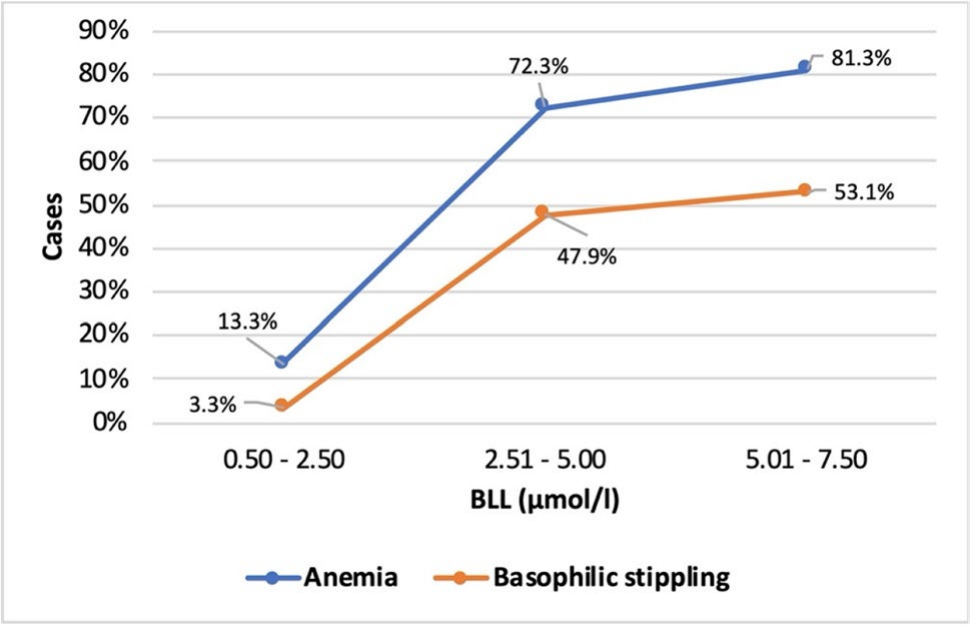
Correlation between BLL and quantity of anemia and basophilic stippling. The patients’ BLLs were first divided into 3 groups according to their level (in μmol/l). The percentages indicate how many patients in each BLL group were diagnosed with anemia and/or basophilic stippling in addition to an elevated BLL.

Anemia and basophilic stippling were more common at higher BLLs. At BLLs of 0.5 to 2.5 μmol/l, only 13.3% showed anemia and 3.3% showed basophilic stippling. At higher BLLs in the range of 2.51 to 5 μmol/l, these values increased massively. Here, 72.3% had anemia and 47.9% had basophilic stippling. In the highest measured BLLs of 5.01 to 7.5 μmol/l, 81.3% were diagnosed with anemia and 53.1% had basophilic stippling (Fig. 14). This shows that anemia and basophilic stippling can indicate lead intoxication, but mainly high level lead intoxication. Since there are significantly fewer published cases of arsenic and mercury poisoning and poisoning with other metals, there are correspondingly fewer values available for the levels of these metals in the blood, so that a correlation of these values was not investigated.

In addition to hematologic effects, hepatic and renal changes due to metals can also be detected in the blood. Liver abnormalities were found in 23.8% of patients, mainly an increase in liver enzymes (19.1%, including aspartate aminotransferase and alanine aminotransferase) and hyperbilirubinemia (10.5%). In addition, urinary abnormalities such as elevated creatinine levels, hyponatremia, and proteinuria were occasionally observed.

### Diagnosis

In many case reports, there was a significant delay before the correct diagnosis was made. Usually, various, sometimes superfluous, examinations and tests were performed, and sometimes incorrect diagnoses were initially made. Often, there were clear signs of metal intoxication that were not correctly interpreted until a very late stage. However, it was not uncommon for delays to occur because patients did not disclose the use of T&AM. 18.6% of the patients did not mention the intake in their medical history, but only after being asked directly, sometimes several times. Because many T&AMs are largely derived from plants, many people believe that T&AMs are harmless and cannot have any side effects or risks. Patients rely on the safety of the medications and therefore do not consider it necessary to mention their use. In addition to unintentional non-mentioning, intentional non-disclosure may occur when a patient does not want to admit to taking the medication because of the reason for taking it (e.g., sexual dysfunction).

Of the patients, 9.5% did not report the use of T&AM at the outset, but reported it during the course of the examination; 5.2% of the patients reported the use directly in the medical history, but the drugs were not initially suspected and were therefore investigated only after a considerable delay; 6.2% of the patients were examined on suspicion because a family member or acquaintance had been diagnosed with metal poisoning; and 4.8% of the patients were found to have elevated metal levels during a routine examination. The exact time to diagnosis was usually not given, but in some cases it was given as several months; 18.1% of patients were initially prescribed medication for symptomatic treatment because the cause of the illness was unknown, and 19.1% were discharged with an incorrect or no diagnosis, but the majority of these patients presented again and were then correctly diagnosed. In about one fourth of the patients (23.8%), the laboratory values were the deciding factor in suspecting metal poisoning, whereupon the metal load in the body was investigated.

The most commonly performed tests include gastrointestinal endoscopy, abdominal ultrasound and radiography, computed tomography (usually of the abdomen), and chest radiography. These examinations were often without pathological findings, but it is remarkable that 45% of the patients under the age of two were found to have increased bone density in the metaphyses, which is due to the deposition of lead in the bones the fact that lead is preferentially deposited in areas of active bone growth (Smith and Hursh, 1977, as of Wittmers et al. 1988). Radiodense particles containing metal were seen in the abdominal radiographs of five patients. Three patients underwent surgery due to an unclear cause of illness, e.g., an appendectomy was performed as part of an exploratory laparotomy, but there was no appendicitis.

The diagnosis can be simplified for physicians if they know that contaminated T&AM can lead to metal poisoning. With this background knowledge, they can ask patients with symptoms suggestive of metal poisoning specifically about the ingestion of T&AM, unless they have not reported the ingestion on their own. The evaluation of the available case reports showed that some symptoms were particularly common, but were mostly non-specific. Since these non-specific symptoms, such as abdominal pain, nausea, loss of appetite and weight, facial pallor, and weakness, can have various causes, diagnosis without suspicion of metal poisoning can be massively delayed, making physicians dependent on patient cooperation. Anemia and basophilic stippling, especially in combination with abdominal pain, have been shown to be clear indications of metal intoxication, although metal intoxication does not necessarily involve these symptoms. If metal poisoning is suspected, appropriate blood and laboratory tests can be performed promptly, and the correct diagnosis can be made more quickly. In this way, unnecessary, often invasive examinations involving X-rays can be avoided.

### Treatment

The treatment of metal poisoning can be divided into conservative and interventional approaches. In 42.6% of the patients treated conservatively, no further therapeutic intervention was performed other than discontinuation of the drug. In 29.8% of patients, supportive measures were carried out in addition to discontinuation of the medication, including symptom relief (e.g., with analgesics), micronutrient supplementation, treatment of skin lesions (usually caused by arsenic), and physiotherapy.

**Fig. 15:**
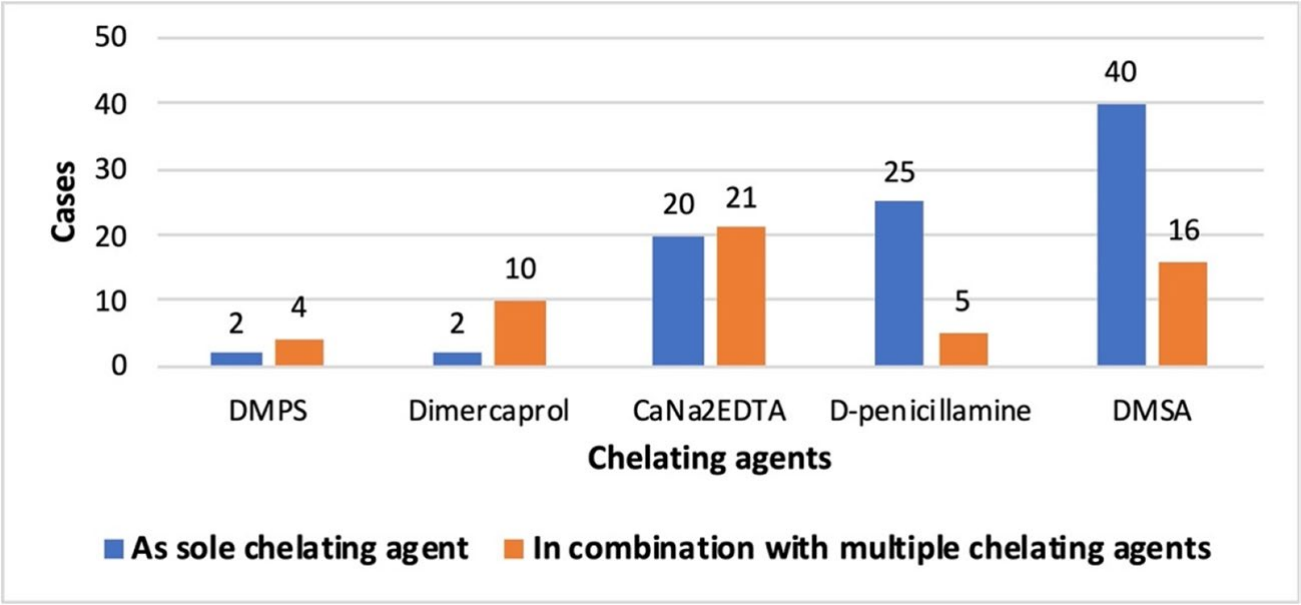
This bar chart shows the absolute number of chelating agents used. The blue bars represent the cases where only one chelator was used (either in one or more cycles), and the orange bars represent the cases where multiple chelators were used during the course of chelation therapy.

If conservative therapy is not considered sufficient, chelation therapy can also be used, which was performed in 64.8% of patients to reduce the metal burden in the body. Chelation therapy was performed about 5% more often in men than in women. In 22.4% of all patients, chelation therapy was explicitly not given. In another 12.9%, it was not stated whether a chelation therapy was administered. Only one chelator was used in 65.4% of the cases and more than one chelator in 18.4% of the cases. In 16.2% of the cases, no details were given about the chelation therapy and the chelating agents used. Dimercaptosuccinic acid (DMSA), calcium disodium ethylenediamine tetraacetate (CaNa2EDTA), and D-penicillamine were the most commonly used. Dimercaprol and Dimercapto-propanesulfonic acid (DMPS), on the other hand, were rarely used, and when they were, they were usually used in combination with other chelators (Fig. 15). The use of Dimercaprol and DMPS each caused severe side effects once, so that it was necessary to switch to a different chelator.

Chelation therapy was performed in 74% of lead poisoning cases and only 22.7% of arsenic poisoning cases.

An important decision criterion for or against chelation therapy is the metal concentration in the blood. When chelation therapy was not used in lead poisoning cases, the average BLL was 2.95 μmol/l. In cases where chelation therapy was performed, the average BLL was 4.38 μmol/l.

In cases of acute intoxication, it is particularly important to identify the cause as quickly as possible and to initiate appropriate treatment to minimize the accumulation of metal in the bones. After ingestion, lead initially enters the bloodstream. With prolonged exposure, the lead is deposited in soft tissues, organs, and especially bones. The half-life of lead deposited in bone is 16 to 20 years. Studies have shown that chelation therapy is only effective in removing metals from the blood, but not from the bone (Chisolm 2001). Treatment of chronic metal poisoning is therefore much more difficult and prolonged than acute poisoning, making early diagnosis all the more important.

### Clinical outcome and sex-specific differences

At the time of the last check-up mentioned in the case report, the patients’ health status varied. Clinical improvement was observed in 51.9% of the patients. We define clinical improvement here as follows: At the time of the last check-up, the patient was still symptomatic and/or the metal concentration in the blood had not yet reached the normal range specified in the case report. Complete recovery was neither confirmed nor questioned; 22.4% of the patients had a complete clinical recovery, i.e., they are symptom-free, and the laboratory values have normalized according to the information in the case report. Incomplete recovery affected 4.8% (10 cases). In these patients, a complete recovery is very doubtful or not to be expected in the further course. Seventy percent of patients with incomplete recovery have residual neuropathy. Thirty percent reported paresthesia, and 20% reported residual weakness. One patient developed multiple arsenic-associated squamous cell carcinomas after 15 months, and another patient suffered permanent anoxic brain damage.

Arsenic was involved in 80% of these cases, while lead was present in only 40% of cases with incomplete recovery. In 38.7% of patients with lead intoxication, the BLL was no longer measured or at least not reported at a follow-up appointment. Only 9.4% of the patients had a BLL ≤0.5 μmol/l at the last follow-up and were therefore in the normal range; 40.9% had an elevated BLL in the range of 0.51 to 2.0 μmol/l, and 8.84% had a BLL >2 μmol/l.

The severe treatability of chronic metal poisoning requires long-term monitoring. Different blood metal levels are considered normal from country to country and sometimes even from hospital to hospital. As a result, patients with sometimes still very high blood metal levels are declared healthy and treatment is discontinued. At the time the case reports were published, no further follow-up appointments were planned for the majority of patients. Of all patients who were not fully recovered at the time of the last follow-up visit, only 5.9% were scheduled for future follow-up. Despite incomplete recovery (e.g., still significantly elevated BLL), the majority of patients were followed up less than 6 months after diagnosis (Fig. S12), although the half-life of lead stored in the bone can be up to 20 years.

Bone remodeling processes may release lead into the blood and thus be responsible for an elevated BLL as an endogenous source of exposure, even though external lead exposure is no longer present (Klaassen 2018). Bone turnover increasing conditions are among others pregnancy, lactation, menopause, and osteoporosis (Garnero et al. 1996; Silbergeld 1991). Without long-term monitoring, a renewed increase in metal levels in the blood may go unnoticed. Furthermore, patients should be regularly examined after severe metal poisoning due to possible long-term effects. For example, there are patients who suffer from residual neuropathy after metal poisoning. Since arsenic is carcinogenic and can cause skin lesions, there is an increased risk of skin cancer after chronic arsenic poisoning (Tang et al. 2023), so possible skin lesions should be monitored regularly in the long term.

**Fig. 16:**
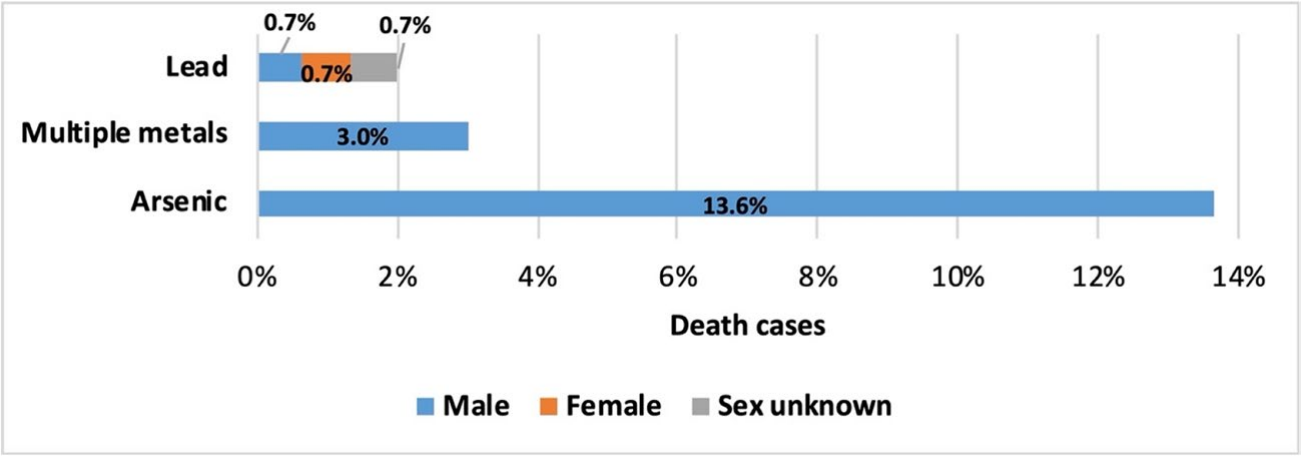
Fatality analysis—Percentage of deaths in relation to the total number of intoxication cases with the respective metal (Lead: three deaths out of 150 total cases of lead intoxication; multiple metals: one death out of 33 total cases; arsenic: three deaths out of 22 total cases); not included in this figure: three miscarriages

In seven cases (3.3%), the metal intoxication was fatal. Of the seven deaths, 57.1% were diagnosed with metal poisoning as the cause of death only at the post-mortem examination. Arsenic and lead were the cause of three deaths each. In relation to the total number of poisonings with the respective metal, deaths related to arsenic (13.6%) are more than six times more frequent than deaths related to lead (2%, Fig. 16). One death was caused by poisoning with more than one metals; 71.4% of the deaths were male, only one was female, and the sex of one patient was unknown. In three women, miscarriage occurred in temporal connection with the metal intoxication. Exposure to metals can increase the risk of spontaneous abortions (Borja-Aburto et al. 1999), but a causal connection has not been proven in these cases.

The evaluation of all patient cases shows that men seem to be more sensitive to metal poisoning than women. On average, men have a shorter duration of intake and a lower total intake than women. Nevertheless, men had an earlier onset of symptoms on average and more total symptoms than women. Women were more likely to have an asymptomatic course. In addition, the average BLL was higher in men than in women, and men were more likely to have a low Hb level. Due to the more severe course of the disease, men also required chelation therapy more often, but even after chelation, the average BLL was still higher in men (1.36 μmol/l) than in women (1.23 μmol/l, Fig. 12). In addition, more men than women died from metal poisoning.

It is not entirely understood why men appear to be more susceptible to metal poisoning than women. In addition to environmental and occupational factors (workplaces with increased metal exposure), which may cause men to have higher average metal levels in their blood due to greater exposure, certain behavioral (e.g., alcohol and nicotine consumption) and biological factors may also be responsible for men being more vulnerable to metal exposure in general. Depending on the metal, different factors are thought to be responsible for the increased susceptibility. Lead is bound to erythrocytes in the blood, and because men have a higher average hematocrit than women, they have more erythrocytes in their blood to which ingested lead can be bound (Gade et al. 2021). Higher BLLs can in turn lead to more severe symptoms. Arsenic is metabolized primarily by methylation. The resulting products vary in toxicity. For example, monomethylamine (MMA) is considered more toxic than dimethylamine (DMA). Studies have shown that men have higher levels of MMA and lower levels of DMA than women, meaning that they have a higher concentration of the more toxic MMA component in their blood when arsenic is metabolized. Since inorganic arsenic is first methylated to MMA and then to DMA in a second step, the lower MMA/DMA ratio in women also reflects a higher methylation capacity compared to men (Gade et al. 2021; Shen et al. 2016). The more efficient methylation in women may be due to hormonal factors. Estrogen promotes the methylation of arsenic by stimulating the synthesis of phosphatidylcholine, which in turn indirectly promotes the formation of methyl donors for arsenic methylation (Tseng 2009). In addition to sex, alcohol and nicotine consumption, which can be higher in men than in women, also have a negative impact on methylation capacity (Gade et al. 2021; Shen et al. 2016; Tseng 2009).

These and other possible factors should be further investigated in systematic studies in order to explain the discrepancy in physical responses to metal exposure between men and women.

### Bibliometric analysis

A total of 91 journals published one or more of the 132 case reports analyzed. Of these journals, 25.3% were from the USA and 23.1% from the UK; 9.9% of the journals were from India and a further 5.5% each from the Netherlands and South Korea (Fig. S13). The most frequently represented publishers are Wiley, Elsevier, Sage Publications, Springer, and Medknow Publications, which together publish 38.5% of the 91 journals (Tab. S1). In most cases, the language of publication is English (94.7%). Two case reports (1.5%) each are written in Korean and German and one (0.8%) each in French, Dutch, and Spanish.

The majority of case reports (77.3%) present one patient case. All others are case series, of which 9.85% contain two and 6.8% three patient cases. The remaining 6.1% contain five or more patient cases (Fig. S14).

The case reports vary greatly in the number of times they have been cited since publication. The citation frequency ranges from zero to 90 citations with a mean of 17.5 citations (as of 16/09/2023, Fig. S15). Similar to the citation frequency, the Journal Impact Factor (JIF) of the journals shows a wide range. In the years in which the case reports were published, the journals had a JIF ranging from a minimum of 0.3 to a maximum of 53.49, with a mean value of 1.76 (Fig. S16); 92.5% had a JIF ≤10 at the time of publication, and only five journals had a JIF >10. The JIF of most journals increased over time so that in 2022 the corresponding journals had a JIF ranging from 0.27 to 158.5 with a mean value of 9.58 (Fig. S17). It is conceivable that case reports published in a journal with a high JIF were cited more frequently on average than case reports published in a journal with a comparatively low JIF. However, our analyses did not reveal a linear relationship between the citation frequency and the JIF in the year of publication (Fig. S18), nor between the citation frequency and the JIF in 2022 (Fig. S19).

The 25 most frequently cited case reports were published between 1977 and 2013, with the average year of publication being 1996 (Fig. S20). Ninety-two percent of these 25 case reports are currently more than 15 years old, and 60% are more than 25 years old. Even the youngest case report among the Top 25 most cited case reports is more than 10 years old. This shows that current publications mainly draw their information from older case reports, and only a few authors refer to more recent case reports. In addition, case reports from the USA (28%), the UK (20%), and India (12%) are increasingly cited (Fig. S21). A total of eight case reports (6.1%) have no citations. Two each came from Australia and China, and one case report each came from Canada, India, Israel, and South Korea.

## Limitations

There are limitations to the results of this systematic analysis. Many case reports from different countries were selected through the systematic search in PubMed, but it is unclear how high the number of published case reports is that were not found despite the systematic search. Most of the case reports refer to lead poisoning by Indian T&AM, while poisoning with other metals by T&AM from other countries is relatively rarely represented.

The case reports were analyzed for various aspects, but not all patient and examination data were available for each case, so that cases with missing information could not be included in the analysis of a corresponding category. In addition, we only had access to the case reports themselves and not to the original examination results, so we had to assume the accuracy of the data.

Due to the high heterogeneity of the cases and the partial lack of patient data, including the amount and duration of intake and the medication itself, group comparisons and testing for statistical significance were only possible to a very limited extent. Because these were not randomized clinical trials, but individual case reports, the results are limited to the available case reports and are not generalizable. If possible, these limitations should be addressed in future publications in order to gain further insights.

## Conclusions

By evaluating many patient cases about various categories, it was possible to generate information and correlations about metal poisoning caused by T&AM. Metal intoxications caused by T&AM are still widespread. The poisonings were primarily caused by lead and some by arsenic and other metals due to T&AM, which originated mainly from Asia. These poisonings often present similar symptoms that are specific for the respective metal, but non-specific for diagnosis (e.g., abdominal pain), which can make diagnosis difficult in the absence of anamnestic information from the patient. After reviewing all the available cases, it is clear that there is a lack of defined standards and quality controls to check for contamination and that there is a great need for education on the part of patients as well as prescribers and practitioners. Large sections of the population are unaware that T&AM are often contaminated with metals, so that these drugs are usually only suspected at a late stage as the cause of corresponding symptoms of illness. In addition to extensive quality control and education, post-treatment monitoring is needed for a much longer period of time to ensure that the blood metal levels have decreased to a level that is as safe as possible in the long term.

Our present paper also provides an unexpected case study for the importance to include sex and gender aspects into a research program. When we initiated the project, we had the preconceived opinion that women should be more sensitive to lead and arsenic intoxication than men. But the results showed the opposite of our expectation. Therefore, the present study may also raise the awareness of pharmacologists, toxicologists, patients, and T&AM consumers alike that men are more sensitive to T&AM intoxication than assumed by general wisdom. This is the first step towards correcting sex imbalances in the diagnosis and treatment of lead and arsenic intoxications.

## Supplementary information

ESM 1 (DOCX 783 kb)

## Data Availability

All source data for this study are available upon reasonable request. Summaries of the case characteristics are described in Tables S2A-2F.

## Abbreviations

BLL: Blood lead level (concentration)
CaNa_2_EDTA: Calcium disodium ethylenediaminetetraacetic acid
DMA: Dimethylamine
DMPS: 2,3-Dimercapto-1-propanesulfonic acid
DMSA: Dimercaptosuccinic acid
Hb: Hemoglobin
JIF: Journal impact factor
MMA: Monomethylamine
N/A: Data not available
T&AM: Traditional and alternative medicine
WHO: World Health Organization
δ-ALAD: Delta-aminolevulinic acid dehydratase
δ-ALA: Delta-aminolevulinic acid
