# Supplementary material for: Lead and arsenic intoxications by traditional and alternative medicine: men are more sensitive than women
Source: Naunyn Schmiedebergs Arch Pharmacol. 2024 Jul 27;398(1):799–818. doi: 10.1007/s00210-024-03317-y (PMC11787186; doi:10.1007/s00210-024-03317-y)
Supplement: Supplementary file 1 — (DOCX 783 kb) [file 210_2024_3317_MOESM1_ESM.docx]

# Supplemental data

# Lead and arsenic intoxications by traditional and alternative medicine: Men are more sensitive than women

# Lucia Gerke and Roland Seifert

**Supplemental Figures and Tables**

*Fig. S1: Number of publications per continent in percent (A) and number of publications per country in total numbers (B); ^1^USA = United States of America, ^2^UK = United Kingdom, ^3^UAE = United Arab Emirates*

*Fig. S2: This pie chart shows the sex distribution of the 210 cases in total numbers, with male patients colored blue and female patients colored orange. The gray area represents patients with unknown sex.*

*Fig. S3: This bar chart shows the form of administration of the T&AM. Some patients used more than one T&AM in more than one dosage form. Oral dosage forms include tablets, capsules, powders, liquids, sprays, syrups, and pastes. Ointments, powders, and patches were applied dermally. A few patients used a nasal spray.*

*Fig. S4: Obtaining of the medication (minor patients) – Who or where did the parents turn to in order to acquire T&AM for their children? The designation of the person or institution consulted does not necessarily indicate the level of medical qualification.*

*
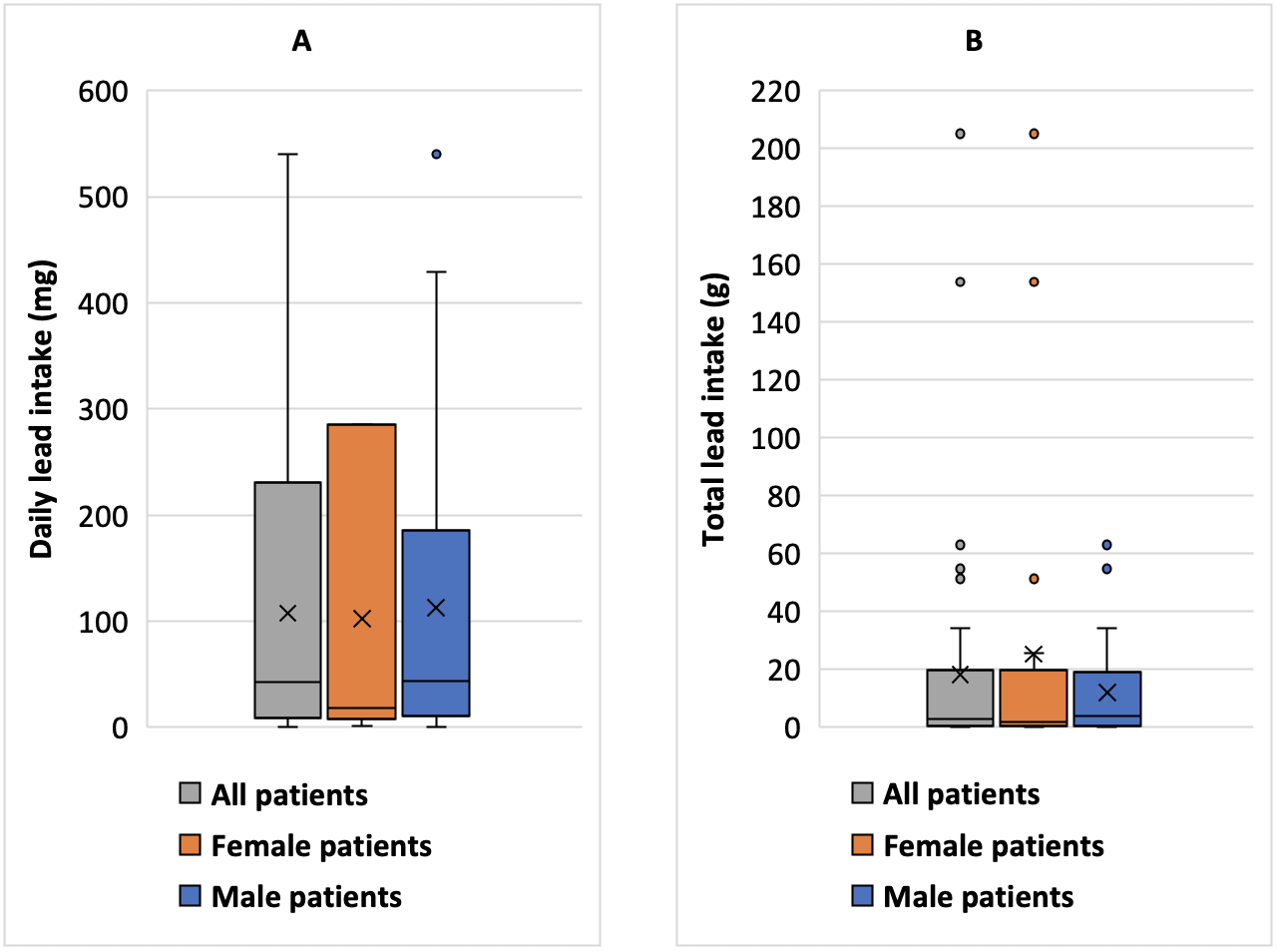
*

*Fig. S5: Box plots of the daily lead intake in milligrams (A) and total lead intake in grams (B) sorted by sex. The* ***x*** *indicates the mean value. A) The daily lead intake was reported in 43 cases (21 females and 22 males); B) The total lead intake was reported in 42 cases (20 females and 22 males).*

*Fig. S6A: Representation of all types of pain symptoms in total numbers. Some patients had more than one pain symptom and are therefore represented multiple times in this bar chart; ^1^others = left eyeball pain, anal pain*

*Fig. S6B: Representation of all types of gastrointestinal symptoms in total numbers. Some patients had more than one gastrointestinal symptom and are therefore represented multiple times in this bar chart; ^1^other gastrointestinal symptoms = dark colored stools, intestinal pseudo-obstruction, rectal blood loss, gaseous distension of the abdomen, not further specified gastrointestinal symptoms, epigastric discomfort, watery and green stool (later contained blood), gastrointestinal illness*

*Fig. S6C: Representation of all types of neurological and (neuro)muscular symptoms in total numbers. Some patients had more than one of these symptoms and therefore appear more than once in this bar chart.*

*Fig. S6D: Representation of all types of constitutional symptoms in total numbers. Some patients had more than one constitutional symptom and therefore appear more than once in this bar chart.*

*Fig. S6E: Total number of all types of symptoms affecting the skin, skin appendages and/or mucous membranes. Some patients had more than one of these symptoms and therefore appear more than once in this bar chart.*

*Fig. S6F: Representation of all types of symptoms affecting the sleep quality in total numbers.*

*Fig. S6G: Total numbers of all types of respiratory symptoms. Some patients had more than one respiratory symptom and therefore appear more than once in this bar chart.*

*Fig. S6H: Representation of all types of liver symptoms in total numbers. Some patients had more than one liver symptom and therefore appear more than once in this bar chart.*

*Fig. S6I: Total numbers of all types of symptoms affecting the renal or urinary system. Some patients had more than one of these symptoms and therefore appear more than once in this bar chart.*

*Fig. S6J: Representation of all types of symptoms affecting the cardiovascular system in total numbers.*

*Fig. S6K: Representation of all symptoms that affect a patient’s sexual function.*

*Fig. S7: Representation of all BLL limits given in the analyzed articles in form of a scatter chart with a trend line. These are the BLL values in µmol/l that are still considered normal according to the corresponding article. The X-axis indicates the year in which the article was published.*


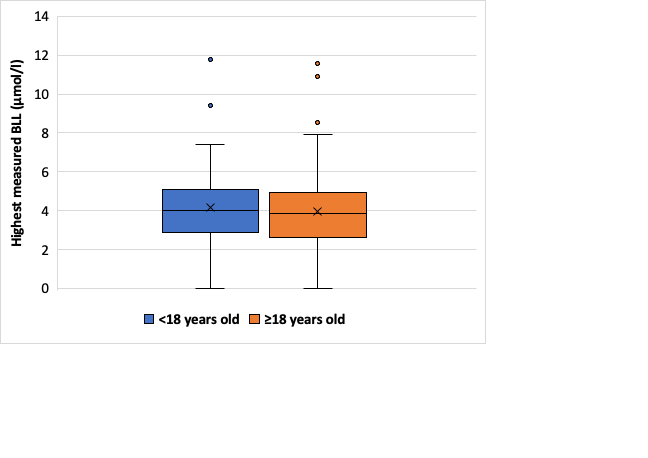


*Fig. S8: Comparison of the highest measured BLL in minor and adult patients. Patients with unknown BLL were excluded from these box plots. Since the BLL refers only to lead, these box plots include only patients who consumed lead-contaminated T&AM.*


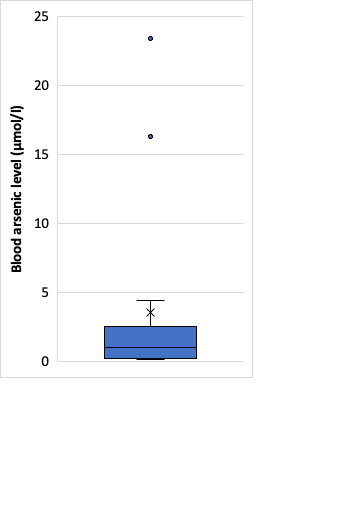


*Fig. S9: Box plot of the blood arsenic levels given in the case reports (N = 16) in form of a box plot. The blood arsenic levels are expressed in µmol/l.*

*Fig. S10: Representation of the occurrence of certain laboratory signs of metal poisoning: Anemia and basophilic stippling. The blue bars represent the cases in which anemia or basophilic stippling was diagnosed and the orange bars represents the cases in which anemia and basophilic stippling were ruled out. The gray bars represent the cases where it is unknown whether the patients were tested for anemia and basophilic stippling.*

*Fig. S11: Representation of anemia types in total numbers. Some patients had anemia where two types were combined, e.g. normocytic, normochromic anemia, so these patients are represented more than once in this bar chart.*

*Fig. S12: Follow-up regime – overview of the time of the last examination of the patients. Patients were categorized according to their health status, with the blue bars representing patients who were already fully cured at the time of the article’s publication. The orange bars represent patients who have experienced clinical improvement but are not yet considered fully cured. The gray bars represent patients who are not expected to make a full recovery in the future. ^1^These patients were followed up, but the time and number of follow-up visits were not reported; ^2^The last reported follow-up visit was immediately after the end of chelation therapy; ^3^These patients were scheduled to be followed up after the publication of the article, so it is not known over what time period further controls occurred.*

*Fig. S13: Total numbers of the countries of origin of the journals that published one or more of the case reports analyzed; ^1^USA = United States of America*

| **Publisher** | **Quantity (Percent)** |
| --- | --- |
| Wiley | 9 (9.9%) |
| Elsevier / Elsevier imprint | 9 (9.9%) |
| Sage Publications | 6 (6.6%) |
| Springer | 6 (6.6%) |
| Medknow Publications (Wolters Kluwer) | 5 (5.5%) |
| BioMed Central (BMC) | 4 (4.4%) |
| BMJ Publishing Group | 4 (4.4%) |
| Taylor & Francis | 4 (4.4%) |
| Lippincott Williams & Wilkins | 3 (3.3%) |
| Oxford University Press | 3 (3.3%) |
| Acta Dermato-Venereologica | 1 (1.1%) |
| Adis International Limited | 1 (1.1%) |
| American Academy of Pediatrics | 1 (1.1%) |
| American Medical Association | 1 (1.1%) |
| American Society of Neuroradiology | 1 (1.1%) |
| Centers Disease Control & Prevention | 1 (1.1%) |
| Canadian Medical Association | 1 (1.1%) |
| F1000 Research Ltd | 1 (1.1%) |
| Frontiers Media SA | 1 (1.1%) |
| Galenos Publishing House | 1 (1.1%) |
| Georg Thieme Verlag KG | 1 (1.1%) |
| Hindawi Limited | 1 (1.1%) |
| Hong Kong Academy of Medicine Press | 1 (1.1%) |
| Israel Medical Association Journal | 1 (1.1%) |
| Japan Society of Internal Medicine | 1 (1.1%) |
| Korean Academy of Medical Sciences | 1 (1.1%) |
| Korean Association of Internal Medicine | 1 (1.1%) |
| Korean Pediatric Society | 1 (1.1%) |
| Korean Society of Gastroenterology | 1 (1.1%) |
| Korean Society of Occupational & Environmental Medicine | 1 (1.1%) |
| Maney Publishing | 1 (1.1%) |
| Mansa STM Publishers | 1 (1.1%) |
| Marcel Dekker Inc | 1 (1.1%) |
| Massachusetts Medical Society | 1 (1.1%) |
| Masson Editeur | 1 (1.1%) |
| Mattioli 1885 | 1 (1.1%) |
| MDPI | 1 (1.1%) |
| Nature Portfolio | 1 (1.1%) |
| New Zealand Medical Association | 1 (1.1%) |
| Oman Medical Specialty Board | 1 (1.1%) |
| Pure Earth Blacksmith Institute | 1 (1.1%) |
| Quadrant Healthcom Inc | 1 (1.1%) |
| Royal Australian College General Practitioners | 1 (1.1%) |
| Saudi Medical Journal | 1 (1.1%) |
| Tehran University of Medical Sciences | 1 (1.1%) |
| Tohoku University Medical Press | 1 (1.1%) |
| Urban & Vogel | 1 (1.1%) |
| Vereniging Nederlands Tijdschrift voor Geneeskunde | 1 (1.1%) |

*Tab. S1: Tabular list of all publishers of the journals that have published one or more case reports. Both the total number and the percentage (in brackets) are given.*

*Fig. S14: Representation of the number of patients included in each case report or case series. By case reports we mean articles that report on a single patient. Case series always include more than one patient.*


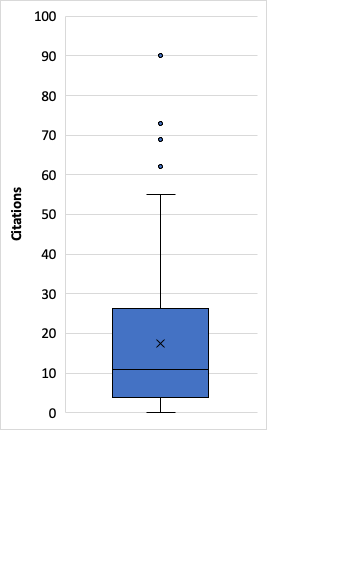


*Fig. S15: Box plot of the number of citations of the analyzed case reports/series (as of September 16, 2023 on https://clarivate.com/products/scientific-and-academic-research/research-discovery-and-workflow-solutions/webofscience-platform/). The box plot includes values from 94 articles. The citations of 39 articles are unknown. The citations of case series with more than one patient are counted only once.*


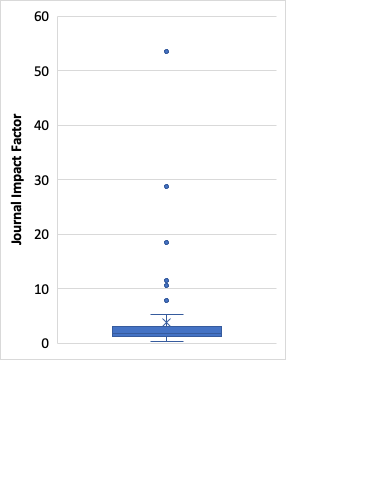


*Fig. S16: Box plot of the Journal Impact Factor of the publishing journals by the year of the publication (as of September 16, 2023 on https://clarivate.com/products/scientific-and-academic-research/research-analytics-evaluation-and-management-solutions/journal-citation-reports/). The box plot includes the JIF of 67 journals. The JIF of 24 journals by the year of the publication is unknown.*


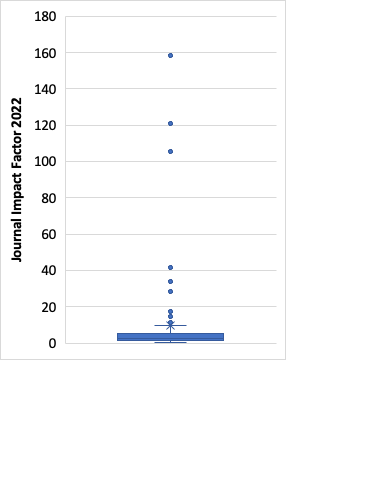


*Fig. S17: Box plot of the Journal Impact Factor of the publishing journals in 2022 (as of September 16, 2023 on https://clarivate.com/products/scientific-and-academic-research/research-analytics-evaluation-and-management-solutions/journal-citation-reports/). The box plot includes the JIF of 81 journals. The JIF 2022 of 10 journals is unknown.*

*Fig. S18: Correlation between the citation frequency (as of September 16, 2023 on https://clarivate.com/products/scientific-and-academic-research/research-discovery-and-workflow-solutions/webofscience-platform/) and the JIF of the publishing journal by the year of the publication.*

*Fig. S19: Correlation between the citation frequency (as of September 16, 2023 on https://clarivate.com/products/scientific-and-academic-research/research-discovery-and-workflow-solutions/webofscience-platform/) and the JIF of the publishing journal by the year 2022.*


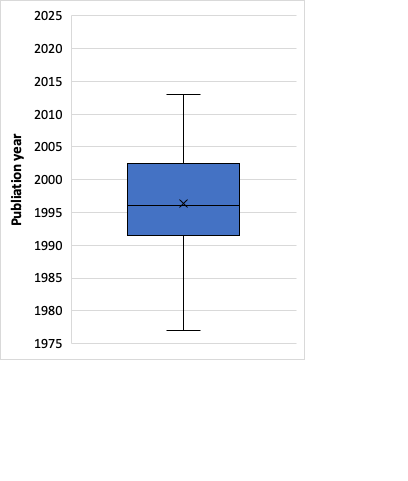


*Fig. S20: This box plot represents the publication year of the 25 most cited case reports/series (as of September 16, 2023 on https://clarivate.com/products/scientific-and-academic-research/research-discovery-and-workflow-solutions/webofscience-platform/).*

*Fig. S21: This bar chart represents the publication country of the journals that published the 25 most cited case reports/series (as of September 16, 2023 on https://clarivate.com/products/scientific-and-academic-research/research-discovery-and-workflow-solutions/webofscience-platform/).*

| **Case number** | **Reference** | **Publica-tion year** | **Number of patients in the article** | **Sex / Age (in years)** | **Ethnic origin** | **Publication country** | **Citations (as of 16.09.2023)** | **Journal (ISO Abbreviation)** |
| --- | --- | --- | --- | --- | --- | --- | --- | --- |
| 1 | Chan et al., 1977 | 1977 | 1 | M / <1 | Chinese | China | 42 | Clin. Toxicol. |
| 2 | Lightfoote et al., 1977 | 1977 | 1 | F / 59 | N/A | USA | 42 | JAMA-J. Am. Med. Assoc. |
| 3 | Brearley and Forsythe, 1978 | 1978 | 1 | M / 24 | Bangladeshi | UK | 14 | BMJ Case Rep. |
| 4 | Centers for Disease Control and Prevention (CDC), 1983a | 1983 | 2 | M / 1 | Californian | USA | N/A | MMWR-Morb. Mortal. Wkly. Rep. |
| 5 | Centers for Disease Control and Prevention (CDC), 1983a | 1983 | 2 | N/A / 3 | Californian | USA | " | " |
| 6 | Centers for Disease Control and Prevention (CDC), 1983b | 1983 | 1 | F / <1 | Hmong | USA | N/A | MMWR-Morb. Mortal. Wkly. Rep. |
| 7 | Bose et al., 1983 | 1983 | 3 | M / <1 | Mexican-american | USA | 63 | Pediatrics |
| 8 | Bose et al., 1983 | 1983 | 3 | F / 6 | Hispanic | USA | " | " |
| 9 | Bose et al., 1983 | 1983 | 3 | F / 2 | Mexican-american | USA | " | " |
| 10 | Centers for Disease Control and Prevention (CDC), 1984 | 1984 | 1 | M / <1 | Asian Indian | USA | N/A | MMWR-Morb. Mortal. Wkly. Rep. |
| 11 | Pontifex and Garg, 1985 | 1985 | 2 | M / 35 | Asian Indian | Canada | 22 | Can. Med. Assoc. J. |
| 12 | Pontifex and Garg, 1985 | 1985 | 2 | F / N/A | Indian | Canada | " | " |
| 13 | Rahman et al., 1986 | 1986 | 6 | F / <1 | N/A | UAE | 18 | Ann. Trop. Paediatr. |
| 14 | Rahman et al., 1986 | 1986 | 6 | N/A / <1 | N/A | UAE | " | " |
| 15 | Rahman et al., 1986 | 1986 | 6 | N/A / <1 | N/A | UAE | " | " |
| 16 | Rahman et al., 1986 | 1986 | 6 | N/A / <1 | N/A | UAE | " | " |
| 17 | Rahman et al., 1986 | 1986 | 6 | N/A / <1 | N/A | UAE | " | " |
| 18 | Rahman et al., 1986 | 1986 | 6 | N/A / <1 | N/A | UAE | " | " |
| 19 | Karri et al., 1987 | 1987 | 1 | M / <1 | N/A | China | 22 | Chin. Med. J. |
| 20 | Mitchell-Heggs et al., 1990 | 1990 | 1 | F / 33 | Korean | UK | 31 | Hum. Exp. Toxicol. |
| 21 | McElvaine et al., 1990 | 1990 | 1 | F / 41 | White, non-Hispanic | USA | 27 | JAMA-J. Am. Med. Assoc. |
| 22 | Smitherman and Harber, 1991 | 1991 | 1 | M / 36 | Indian | USA | 32 | Am. J. Ind. Med. |
| 23 | Dolan et al., 191 | 1991 | 1 | M / 50 | Pakistani | UK | 16 | J. R. Soc. Med. |
| 24 | Saryan, 1991 | 1991 | 1 | M / 27 | Asian Indian | USA | 8 | J. Anal. Toxicol. |
| 25 | Dunbabin et al., 1992 | 1992 | 1 | M / 37 | N/A | Australia | 73 | Med. J. Aust. |
| 26 | Kew et al., 1993 | 1993 | 2 | M / 35 | Asian | UK | 90 | BMJ-British Medical Journal |
| 27 | Kew et al., 1993 | 1993 | 2 | M / 32 | Asian | UK | " | " |
| 28 | Centers for Disease Control and Prevention (CDC), 1993 | 1993 | 1 | M / 2 | Mexican | USA | 1 | MMWR-Morb. Mortal. Wkly. Rep. |
| 29 | Keen et al., 1994 | 1994 | 1 | M / 39 | Indian | UK | 62 | Postgrad. Med. J. |
| 30 | Markowitz, 1994 | 1994 | 1 | M / 45 | Korean | USA | 69 | JAMA-J. Am. Med. Assoc. |
| 31 | Perharic et al., 1994 | 1994 | 5 | M / 33 | N/A | UK | 39 | Drug Saf. |
| 32 | Perharic et al., 1994 | 1994 | 5 | M / 35 | N/A | UK | " | " |
| 33 | Perharic et al., 1994 | 1994 | 5 | M / 53 | N/A | UK | " | " |
| 34 | Perharic et al., 1994 | 1994 | 5 | F / 33 | N/A | UK | " | " |
| 35 | Perharic et al., 1994 | 1994 | 5 | M / 49 | N/A | UK | " | " |
| 36 | Bayly et al., 1995 | 1995 | 5 | F / 42 | N/A | UK | 38 | Hum. Exp. Toxicol. |
| 37 | Bayly et al., 1995 | 1995 | 5 | M / 42 | N/A | UK | " | " |
| 38 | Bayly et al., 1995 | 1995 | 5 | F / 58 | Asian | UK | " | " |
| 39 | Bayly et al., 1995 | 1995 | 5 | F / 28 | Asian | UK | " | " |
| 40 | Bayly et al., 1995 | 1995 | 5 | M / 23 | N/A | UK | " | " |
| 41 | Wu et al., 1996 | 1996 | 3 | F / 52 | N/A | Taiwan | 54 | Sci. Total Environ. |
| 42 | Wu et al., 1996 | 1996 | 3 | F / 72 | N/A | Taiwan | " | " |
| 43 | Wu et al., 1996 | 1996 | 3 | M / N/A | N/A | Taiwan | " | " |
| 44 | Prpić-Majić et al., 1996 | 1996 | 6 | F / 37 | N/A | Croatia | 31 | J. Toxicol.-Clin. Toxicol. |
| 45 | Prpić-Majić et al., 1996 | 1996 | 6 | M / 37 | N/A | Croatia | " | " |
| 46 | Prpić-Majić et al., 1996 | 1996 | 6 | F / 37 | N/A | Croatia | " | " |
| 47 | Prpić-Majić et al., 1996 | 1996 | 6 | M / 41 | N/A | Croatia | " | " |
| 48 | Prpić-Majić et al., 1996 | 1996 | 6 | F / 51 | N/A | Croatia | " | " |
| 49 | Prpić-Majić et al., 1996 | 1996 | 6 | M / 4 | N/A | Croatia | " | " |
| 50 | Kulshrestha, 1996 | 1996 | 1 | F / 27 | Asian | UK | 3 | J. Toxicol.-Clin. Toxicol. |
| 51 | Beigel et al., 1998 | 1998 | 1 | M / 43 | Libyan | Israel | 31 | N. Engl. J. Med. |
| 52 | Spriewald et al., 1999 | 1999 | 1 | M / 37 | Western European | Germany | 18 | Occup. Environ. Med. |
| 53 | Centers for Disease Control and Prevention (CDC), 1999 | 1999 | 1 | F / 33 | Cambodian | USA | N/A | MMWR-Morb. Mortal. Wkly. Rep. |
| 54 | Vonderen et al., 2000 | 2000 | 1 | F / 35 | N/A | Netherlands | 21 | Am. J. Gastroenterol. |
| 55 | Li et al., 2000 | 2000 | 1 | M / 5 | Chinese | China | 35 | Arch. Dis. Child. |
| 56 | Moore and Adler, 2000 | 2000 | 1 | M / 5 | Indian | USA | 45 | Pediatrics |
| 57 | Gerdsen et al., 2000 | 2000 | 1 | F / 38 | Pakistani | Germany | 10 | Acta Derm.-Venereol. |
| 58 | Muzi et al., 2001 | 2001 | 1 | M / 5 | N/A | Italy | 14 | J. Pediatr. |
| 59 | Anderson et al., 2001 | 2001 | 1 | M / 23 | Asian | UK | 15 | Ann. Clin. Biochem. |
| 60 | Auyeung et al., 2002 | 2002 | 3 | F / 23 | N/A | China | N/A | Hong Kong Med. J. |
| 61 | Auyeung et al., 2002 | 2002 | 3 | F / 35 | N/A | China | " | " |
| 62 | Auyeung et al., 2002 | 2002 | 3 | F / 48 | N/A | China | " | " |
| 63 | Ibrahim and Latif, 2002 | 2002 | 1 | F / 56 | Indian | Qatar | 26 | Saudi Med. J. |
| 64 | Centers for Disease Control and Prevention (CDC), 2002 | 2002 | 2 | M / 4 | Hispanic | USA | N/A | MMWR-Morb. Mortal. Wkly. Rep. |
| 65 | Centers for Disease Control and Prevention (CDC), 2002 | 2002 | 2 | F / 6 | Hispanic | USA | " | " |
| 66 | Tait et al., 2002 | 2002 | 2 | F / 24 | Indian | Australia | 45 | Med. J. Aust. |
| 67 | Tait et al., 2002 | 2002 | 2 | F / <1 | Indian | Australia | " | " |
| 68 | Weide et al., 2003 | 2003 | 1 | F / 39 | N/A | Germany | 19 | Dtsch. Med. Wochenschr. |
| 69 | Chakraborti et al., 2003 | 2003 | 3 | F / 42 | N/A | India | 35 | J. Toxicol.-Clin. Toxicol. |
| 70 | Chakraborti et al., 2003 | 2003 | 3 | M / 44 | N/A | India | " | " |
| 71 | Chakraborti et al., 2003 | 2003 | 3 | M / 39 | N/A | India | " | " |
| 72 | Lee et al., 2004 | 2004 | 1 | F / 51 | N/A | South Korea | 26 | J. Korean Med. Sci. |
| 73 | Centers for Disease Control and Prevention (CDC), 2004 | 2004 | 12 | F / 37 | N/A | USA | N/A | MMWR-Morb. Mortal. Wkly. Rep. |
| 74 | Centers for Disease Control and Prevention (CDC), 2004 | 2004 | 12 | F / 31 | N/A | USA | " | " |
| 75 | Centers for Disease Control and Prevention (CDC), 2004 | 2004 | 12 | M / 34 | N/A | USA | " | " |
| 76 | Centers for Disease Control and Prevention (CDC), 2004 | 2004 | 12 | M / 62 | N/A | USA | " | " |
| 77 | Centers for Disease Control and Prevention (CDC), 2004 | 2004 | 12 | F / 56 | N/A | USA | " | " |
| 78 | Centers for Disease Control and Prevention (CDC), 2004 | 2004 | 12 | F / 19 | N/A | USA | " | " |
| 79 | Centers for Disease Control and Prevention (CDC), 2004 | 2004 | 12 | F / 25 | N/A | USA | " | " |
| 80 | Centers for Disease Control and Prevention (CDC), 2004 | 2004 | 12 | M / 52 | N/A | USA | " | " |
| 81 | Centers for Disease Control and Prevention (CDC), 2004 | 2004 | 12 | F / 57 | N/A | USA | " | " |
| 82 | Centers for Disease Control and Prevention (CDC), 2004 | 2004 | 12 | F / 40 | N/A | USA | " | " |
| 83 | Centers for Disease Control and Prevention (CDC), 2004 | 2004 | 12 | M / 56 | N/A | USA | " | " |
| 84 | Centers for Disease Control and Prevention (CDC), 2004 | 2004 | 12 | M / 50 | N/A | USA | " | " |
| 85 | Schilling et al., 2004 | 2004 | 1 | F / 60 | N/A | Germany | 11 | Med. Klin. |
| 86 | Choi et al., 2005 | 2005 | 2 | M / 13 | Asian | South Korea | N/A | Korean J. Pediatr. |
| 87 | Choi et al., 2005 | 2005 | 2 | M / 11 | Asian | South Korea | " | " |
| 88 | Kanen and Perenboom, 2005 | 2005 | 1 | M / 46 | N/A | Netherlands | N/A | Ned. Tijdschr. Geneeskd. |
| 89 | Atre et al., 2006 | 2006 | 1 | M / 41 | N/A | India | 28 | Am. J. Neuroradiol. |
| 90 | Garnier and Poupon, 2006 | 2006 | 1 | M / 32 | Indian-Portuguese | France | 14 | Presse Med. |
| 91 | Madan et al., 2007 | 2007 | 1 | M / 24 | N/A | India | 7 | Auton. Neurosci-Basic Clin. |
| 92 | Geraldine et al., 2007 | 2007 | 1 | F / 27 | Indian | India | N/A | Arch. Gynecol. Obstet. |
| 93 | Levit et al., 2007 | 2007 | 1 | F / 35 | Nepalese | Israel | 0 | Isr. Med. Assoc. J. |
| 94 | Madhusudhanan and Lall, 2007 | 2007 | 1 | M / <1 | Omani | Oman | N/A | Oman Med. J. |
| 95 | Hanjani et al., 2007 | 2007 | 1 | F / 42 | Chinese | USA | 11 | Cutis |
| 96 | Creemers et al., 2008 | 2008 | 2 | F / 42 | Belgian | Belgium | 1 | Acta Clinica Belgica |
| 97 | Creemers et al., 2008 | 2008 | 2 | F / 48 | Belgian | Belgium | " | " |
| 98 | Raviraja et al., 2008 | 2008 | 1 | M / 39 | N/A | India | N/A | Indian J. Case Reports |
| 99 | Khandpur et al., 2008 | 2008 | 1 | F / 11 | N/A | India | 38 | Int. J. Lab. Hematol. |
| 100 | Woolf et al., 2008 | 2008 | 1 | N/A / 1 | Thai | USA | 23 | Clin. Toxicol. |
| 101 | Prakash et al., 2009 | 2009 | 1 | M / 60 | South Asian | Canada | 9 | Nat. Rev. Nephrol. |
| 102 | Shamshirsaz et al., 2009 | 2009 | 2 | F / >18 | White | USA | 7 | Obstet. Gynecol. |
| 103 | Shamshirsaz et al., 2009 | 2009 | 2 | M / <1 | N/A | USA | " | " |
| 104 | Singh et al., 2009 | 2009 | 1 | M / 58 | Indian | India | N/A | Indian J. Med. Sci. |
| 105 | Jayachandar and Kotabagi, 2010 | 2010 | 1 | M / 33 | N/A | India | N/A | Med. J. Armed Forces India |
| 106 | Kumar et al., 2010 | 2010 | 1 | M / 35 | N/A | India | N/A | N. Engl. J. Med. |
| 107 | Lee et al., 2010 | 2010 | 1 | F / 31 | N/A | South Korea | N/A | Korean J. Intern. Med. |
| 108 | Raviraja et al., 2010 | 2010 | 3 | M / 45 | N/A | India | N/A | Indian J. Clin. Biochem. |
| 109 | Raviraja et al., 2010 | 2010 | 3 | M / 36 | Indian | India | " | " |
| 110 | Raviraja et al., 2010 | 2010 | 3 | M / 46 | N/A | India | " | " |
| 111 | Leiba et al., 2010 | 2010 | 1 | F / 29 | N/A | USA | 5 | J. Matern.-Fetal Neonatal Med. |
| 112 | Wijeratne et al., 2011 | 2011 | 1 | M / 28 | N/A | Australia | 5 | Med. J. Aust. |
| 113 | Giampreti et al., 2011 | 2011 | 1 | M / 23 | Indian | Italy | 4 | Clin. Toxicol. |
| 114 | Gunturu et al., 2011 | 2011 | 3 | F / 58 | Indian | USA | 35 | J. Hematol. Oncol. |
| 115 | Gunturu et al., 2011 | 2011 | 3 | M / >18 | Indian | USA | " | " |
| 116 | Gunturu et al., 2011 | 2011 | 3 | F / >18 | Indian | USA | " | " |
| 117 | Toniolo et al., 2011 | 2011 | 1 | M / 39 | Sri Lankan | Switzerland | 5 | Br. J. Clin. Pharmacol. |
| 118 | Lin et al., 2012 | 2012 | 1 | M / 25 | N/A | Taiwan | 7 | Tohoku J. Exp. Med. |
| 119 | Tsitsikas et al., 2012 | 2012 | 1 | M / 37 | Indian | UK | 6 | BMJ-British Medical Journal |
| 120 | Kim et al., 2012 | 2012 | 1 | M / 43 | N/A | South Korea | 16 | J. Occup. Med. Toxicol. |
| 121 | Lin et al., 2012 | 2012 | 2 | M / 3 | Chinese | China | 16 | Clin. Chim. Acta |
| 122 | Lin et al., 2012 | 2012 | 2 | F / <1 | Chinese | China | " | " |
| 123 | Centers for Disease Control and Prevention (CDC), 2012 | 2012 | 6 | F / 30 | Indian | USA | N/A | MMWR-Morb. Mortal. Wkly. Rep. |
| 124 | Centers for Disease Control and Prevention (CDC), 2012 | 2012 | 6 | F / 36 | Colombian | USA | " | " |
| 125 | Centers for Disease Control and Prevention (CDC), 2012 | 2012 | 6 | F / 24 | Indian | USA | " | " |
| 126 | Centers for Disease Control and Prevention (CDC), 2012 | 2012 | 6 | F / 35 | Indian | USA | " | " |
| 127 | Centers for Disease Control and Prevention (CDC), 2012 | 2012 | 6 | F / 33 | Indian | USA | " | " |
| 128 | Centers for Disease Control and Prevention (CDC), 2012 | 2012 | 6 | F / 35 | Indian | USA | " | " |
| 129 | Desai and Staszewski, 2012 | 2012 | 1 | M / 56 | N/A | USA | 6 | Am. J. Med. |
| 130 | Pierce et al., 2012 | 2012 | 1 | M / 29 | Indian | USA | 6 | J. Gen. Intern. Med. |
| 131 | Babu et al., 2012 | 2012 | 1 | M / 39 | N/A | India | 2 | Am. J. Med. |
| 132 | Zhu and Zheng, 2012 | 2012 | 1 | M / 64 | N/A | China | 1 | J. Cutan. Med. Surg. |
| 133 | Wu et al., 2013 | 2013 | 2 | M / 51 | N/A | Taiwan | 55 | Am. J. Med. |
| 134 | Wu et al., 2013 | 2013 | 2 | M / 75 | N/A | Taiwan | " | " |
| 135 | Tsutsui et al., 2013 | 2013 | 1 | M / 40 | Asian Indian | New Zealand | N/A | N. Z. Med. J. |
| 136 | Sathe et al., 2013 | 2013 | 1 | F / 2 | N/A | India | N/A | Indian J. Nephrol. |
| 137 | Breeher et al., 2013 | 2013 | 1 | M / 69 | Caucasian | USA | 21 | J. Occup. Med. Toxicol. |
| 138 | Wu and Deng, 2013 | 2013 | 1 | M / 24 | N/A | Taiwan | 12 | Clin. Toxicol. |
| 139 | Muller et al., 2013 | 2013 | 1 | M / 42 | Bhutanese | Switzerland | N/A | F1000Res. |
| 140 | Kim et al., 2013 | 2013 | 1 | F / 59 | Korean | South Korea | 4 | Int. J. Dermatol. |
| 141 | Chang et al., 2013 | 2013 | 1 | F / 38 | N/A | South Korea | 1 | Korean J. Intern. Med. |
| 142 | Gupta et al., 2014 | 2014 | 1 | M / 28 | N/A | India | 12 | Clin. Chim. Acta |
| 143 | Hochholzer et al., 2014 | 2014 | 1 | F / 34 | N/A | Australia | 0 | Aust. Fam. Physician |
| 144 | Pinto et al., 2014 | 2014 | 3 | F / 32 | N/A | India | N/A | J. Med. Toxicol. |
| 145 | Pinto et al., 2014 | 2014 | 3 | M / 20 | N/A | India | " | " |
| 146 | Pinto et al., 2014 | 2014 | 3 | F / 19 | N/A | India | " | " |
| 147 | Fernández et al., 2014 | 2014 | 2 | F / 45 | N/A | Spain | 5 | Med. Clin. |
| 148 | Fernández et al., 2014 | 2014 | 2 | F / 57 | N/A | Spain | 5 |  |
| 149 | Jeon et al., 2015 | 2015 | 1 | M / 61 | N/A | South Korea | N/A | Korean J. Gastroenterol. |
| 150 | Datta-Mitra and Ahmed, 2015 | 2015 | 1 | M / 1 | N/A | USA | 3 | Clin. Pediatr. |
| 151 | Mathee et al., 2015 | 2015 | 8 | F / 16 | N/A | South Africa | 22 | Int. J. Environ. Res. Public Health |
| 152 | Mathee et al., 2015 | 2015 | 8 | M / 16 | N/A | South Africa | " | " |
| 153 | Mathee et al., 2015 | 2015 | 8 | F / 14 | N/A | South Africa | " | " |
| 154 | Mathee et al., 2015 | 2015 | 8 | F / 15 | N/A | South Africa | " | " |
| 155 | Mathee et al., 2015 | 2015 | 8 | F / 15 | N/A | South Africa | " | " |
| 156 | Mathee et al., 2015 | 2015 | 8 | F / 14 | N/A | South Africa | " | " |
| 157 | Mathee et al., 2015 | 2015 | 8 | M / 18 | N/A | South Africa | " | " |
| 158 | Mathee et al., 2015 | 2015 | 8 | F / 26 | N/A | South Africa | " | " |
| 159 | Meiman et al., 2015 | 2015 | 1 | F / 64 | N/A | USA | 6 | MMWR-Morb. Mortal. Wkly. Rep. |
| 160 | Orchard et al., 2015 | 2015 | 1 | M / 53 | N/A | Australia | 3 | Anaesth. Intensive Care |
| 161 | Gulia et al., 2015 | 2015 | 1 | F / 44 | N/A | India | 5 | J. Canc. Res. Ther. |
| 162 | Zhou et al., 2015 | 2015 | 1 | M / 70 | N/A | China | 8 | Int. J. Environ. Res. Public Health |
| 163 | Breyre and Green-McKenzie, 2016 | 2016 | 1 | M / 26 | N/A | USA | N/A | BMJ-British Medical Journal |
| 164 | Ying et al., 2016 | 2016 | 1 | M / 6 | N/A | China | 7 | Clin. Chim. Acta |
| 165 | Budnik et al., 2016 | 2016 | 1 | M / 31 | N/A | Germany | 12 | J. Occup. Med. Toxicol. |
| 166 | Deng et al., 2016 | 2016 | 1 | M / 56 | N/A | China | 3 | Gastroenterology |
| 167 | Senthilkumaran et al., 2017 | 2017 | 1 | F / 15 | N/A | India | N/A | Int. J. Trichology |
| 168 | Tsai et al., 2017 | 2017 | 1 | M / 48 | N/A | Taiwan | N/A | Case Rep. Emerg. Med. |
| 169 | Chambial et al., 2017 | 2017 | 1 | M / 73 | N/A | India | N/A | Indian J. Clin. Biochem. |
| 170 | Sadler and Bell, 2017 | 2017 | 1 | M / 64 | Indian | New Zealand | 5 | Intern. Med. J. |
| 171 | Tang et al., 2017 | 2017 | 2 | M / 66 | N/A | China | 15 | Tohoku J. Exp. Med. |
| 172 | Tang et al., 2017 | 2017 | 2 | F / >18 | N/A | China | " | " |
| 173 | Karwowski et al., 2017 | 2017 | 1 | F / <1 | Malaysian-Taiwanese | USA | 3 | Environ. Geochem. Health |
| 174 | Philips et al., 2018 | 2018 | 1 | M / 54 | N/A | India | N/A | BMJ-British Medical Journal |
| 175 | Ying et al., 2018 | 2018 | 3 | M / 7 | N/A | China | 4 | BMC Pediatr. |
| 176 | Ying et al., 2018 | 2018 | 3 | F / 8 | N/A | China | " | " |
| 177 | Ying et al., 2018 | 2018 | 3 | M / 5 | N/A | China | " | " |
| 178 | Moorthy et al., 2018 | 2018 | 1 | M / 24 | N/A | India | N/A | Adv. J. Emerg. Med. |
| 179 | Zhao and Lv, 2018 | 2018 | 1 | F / 66 | N/A | China | 2 | Turk. J. Hematol. |
| 180 | Siefring et al., 2018 | 2018 | 1 | M / 46 | Vietnamese | Vietnam | N/A | BMJ-British Medical Journal |
| 181 | Soni and Dayal, 2019 | 2019 | 1 | M / 8 | N/A | India | 1 | Indian Pediatrics |
| 182 | Jain et al., 2019 | 2019 | 1 | M / 59 | N/A | India | N/A | BMJ-British Medical Journal |
| 183 | Spilchuk and Thompson, 2019 | 2019 | 1 | M / 73 | Cantonese | Canada | 3 | Can. Med. Assoc. J. |
| 184 | Zheng et al., 2019 | 2019 | 1 | M / 35 | Chinese | China | 17 | Forensic Sci.Int. |
| 185 | Hsiao et al., 2019 | 2019 | 1 | M / 28 | N/A | New Zealand | 9 | BMC Nephrol. |
| 186 | Kang et al., 2019 | 2019 | 1 | M / 50 | N/A | South Korea | 0 | J. Korean Med. Sci. |
| 187 | Lim et al., 2019 | 2019 | 7 | F / 67 | N/A | South Korea | N/A | Ann. Occup. Environ. Med. |
| 188 | Lim et al., 2019 | 2019 | 7 | F / 66 | N/A | South Korea | " | " |
| 189 | Lim et al., 2019 | 2019 | 7 | F / 63 | N/A | South Korea | " | " |
| 190 | Lim et al., 2019 | 2019 | 7 | F / 78 | N/A | South Korea | " | " |
| 191 | Lim et al., 2019 | 2019 | 7 | M / 80 | N/A | South Korea | " | " |
| 192 | Lim et al., 2019 | 2019 | 7 | F / 55 | N/A | South Korea | " | " |
| 193 | Lim et al., 2019 | 2019 | 7 | M / 53 | N/A | South Korea | " | " |
| 194 | Yanamandra et al., 2020 | 2020 | 1 | M / 30 | N/A | India | N/A | BMJ-British Medical Journal |
| 195 | Mahdi et al., 2020 | 2020 | 1 | M / 31 | N/A | India | N/A | J. Health Pollut. |
| 196 | Gopinath et al., 2021 | 2021 | 1 | M / 32 | N/A | India | N/A | BMJ-British Medical Journal |
| 197 | Ciocan et al., 2021 | 2021 | 1 | M / 30 | Indian | Italy | 1 | Med. Lav. |
| 198 | Raut et al., 2021 | 2021 | 2 | F / 54 | N/A | India | 0 | Neurol. India |
| 199 | Raut et al., 2021 | 2021 | 2 | F / 45 | N/A | India | " | " |
| 200 | Chen et al., 2021 | 2021 | 3 | M / 2 | Tibetan | China | 0 | Int. J. Lab. Hematol. |
| 201 | Chen et al., 2021 | 2021 | 3 | N/A / >18 | Tibetan | China | " | " |
| 202 | Chen et al., 2021 | 2021 | 3 | N/A / >18 | Tibetan | China | " | " |
| 203 | Ma et al., 2022 | 2022 | 1 | M / 21 | N/A | China | 0 | Front. Public Health |
| 204 | Ferson et al., 2022 | 2022 | 1 | N/A / <6 | N/A | Australia | 0 | Med. J. Aust. |
| 205 | Horiuchi et al., 2022 | 2022 | 1 | M / 41 | N/A | Japan | 1 | Intern. Med. |
| 206 | Philips et al., 2022 | 2022 | 1 | F / 14 | N/A | India | N/A | Clin. Case Rep. |
| 207 | Shinde et al., 2022 | 2022 | 1 | M / 53 | N/A | India | N/A | J. Med. Toxicol. |
| 208 | Pham and Sharma, 2022 | 2022 | 1 | F / 53 | South Asian | USA | N/A | Proc. (Bayl. Univ. Med. Cent.) |
| 209 | Gitelman et al., 2023 | 2023 | 1 | F / 39 | N/A | Canada | 0 | Can. Med. Assoc. J. |
| 210 | Hardin et al., 2023 | 2023 | 1 | F / 75 | Caucasian | USA | N/A | Clin. Case Rep. |

*Tab. S2A: Raw data table of all patient cases – Reference, Publication year, Number of patients per article, Sex / Age (in years), Ethnic origin, Publication country, Citations, Journal*

| **Case num-ber** | **Kind of T&AM** | **Intake form** | **Prescribed/Pro-cured by whom?** | **Acquired while traveling or imported?** | **Contamination** | **Form of metal** | **Metals mentioned in the title** | **Drug formulation analysis for metals** |
| --- | --- | --- | --- | --- | --- | --- | --- | --- |
| 1 | Chinese herbal medicine (TCM) | N/A (p.o.) | N/A | no | lead | N/A | Lead | mean lead content of 7,5 mg per unit dose (range of 4,004 to 9,44 mg) |
| 2 | Chinese herbal medicine (TCM) | pill | herbalist-acupuncturist | imported | lead | N/A | Lead | lead content of the red pill: 0,5 mg per pill |
| 3 | Aphrodisiac | powder | N/A | imported | lead | N/A | Lead | sample 1: lead content 1,02% (w/w) [other constituents: calcium carbonate and quartz, "fool's gold"]; sample 2: lead content 46% (w/w) [other constituents: sodium chloride, aluminium] |
| 4 | Mexican folk remedy | powder | N/A | yes | lead | azarcon (lead tetroxide, Pb3O4) | Lead | total lead contents varying from 70% to greater than 90% |
| 5 | Mexican folk remedy | powder | N/A | yes | lead | azarcon (lead tetroxide, Pb3O4) | " | total lead contents varying from 70% to greater than 90 |
| 6 | Folk remedy | powder | N/A | imported | lead | N/A | Lead | red powder: lead concentration of 8% |
| 7 | Azarcón (Mexican folk remedy) | powder | baby healer | yes | lead | lead tetroxide (Pb3O4) | Lead | Azarcón contains 86-95% lead tetroxide; in this case it contained 86% lead |
| 8 | Azarcón (Mexican Folk Remedy) | powder | N/A | imported | lead | lead tetroxide (Pb3O4) | " | Azarcón contains 86-95% lead tetroxide |
| 9 | Azarcón (Mexican Folk Remedy) | powder | sent from family | imported | lead | lead tetroxide (Pb3O4) | " | Azarcón contains 86-95% lead tetroxide |
| 10 | Indian folk remedy | powder | N/A | imported | lead | N/A | Lead | samples of 3 folk remedies: all 3 contained lead; highest concentration (1,6%) was in ghasard |
| 11 | Ayurvedic medicine | powder | N/A | yes | lead | N/A | Lead | lead content: 8 mg/g |
| 12 | Ayurvedic medicine | N/A (p.o.) | husband had obtained it in India | yes | lead | N/A | " | N/A (medication was not available) |
| 13 | Indigenous traditional preparation | powder | brought from Iran by traders dealing with folk remedies | imported | lead | N/A | Lead | lead content of 82,5% |
| 14 | Indigenous traditional preparation | powder | brought from Iran by traders dealing with folk remedies | imported | lead | N/A | " | lead content of 82,5% |
| 15 | Indigenous traditional preparation | powder | brought from Iran by traders dealing with folk remedies | imported | lead | N/A | " | lead content of 82,5% |
| 16 | Indigenous traditional preparation | powder | brought from Iran by traders dealing with folk remedies | imported | lead | N/A | " | lead content of 82,5% |
| 17 | Indigenous traditional preparation | powder | brought from Iran by traders dealing with folk remedies | imported | lead | N/A | " | lead content of 82,5% |
| 18 | Indigenous traditional preparation | powder | brought from Iran by traders dealing with folk remedies | imported | lead | N/A | " | lead content of 82,5% |
| 19 | Chinese herbal medicine (TCM) | powder | N/A | N/A | lead | N/A | Lead | powder 1: contained 23,3% lead by weight (leachable lead content: 15,6%); powder 2: contained 44,6 ppm leachable lead by weight; powder 3: contained <1 ppm lead |
| 20 | Korean herbal medicine | pill | traditional practitioner | imported | lead, arsenic | N/A | Lead, arsenic | lead content: 26,4 mg/g; arsenic content: 9,65 mg/g |
| 21 | Indian herbal medicine | pill | Indian medical practitioner | imported | lead, mercury, arsenic, gold; traces of cadmium | N/A | Lead | 1. 6% lead (7,5mg per pill); 2. >70% mercury and significant amounts of arsenic & gold; 3. trace amounts of cadmium |
| 22 | Indian herbal medicine | powder | herbalist | yes | lead | N/A | Lead | one of them, a grayish white powder, had 49% lead |
| 23 | Traditional medicine | paste, pellet, powder | traditional Asian practitioner | imported | lead | mixtures of the lead compounds PbO, PbSO4, Pb(NO3)2 to a total of 84% of elemental lead by weight | Lead | the yellow-white powder was shown to have a very high lead content -> a total of 84% of elemental lead by weight |
| 24 | Indian medicine | tablet | obtained from a relative | imported | lead | N/A | Lead | lead content: 6,47% lead by weight (64.700 ppm) |
| 25 | Ayurvedic medicine | tablet | Indian pharmacist | yes | lead, mercury, arsenic | N/A | Lead | Pushap tablets contained 79,3 mg lead, 0,75 mg arsenic and >10 mg mercury per tablet, Shakti tablets contained 55,9 mg lead, 5,4 mg arsenic and >10 mg mercury per tablet |
| 26 | Indian ethnic remedy | powder | Hakim | imported | arsenic | inorganic arsenic trioxide (As2O3) | Arsenic, mecury | packets of the mixed white and orange-brown powder most strongly implicated in this case each contained an average of 105 mg of inorganic arsenic trioxide |
| 27 | Indian ethnic remedy | pill | Hakim | imported | mercury | inorganic mercuric sulphide | " | handmade red-brown pills: each contained 30-42 mg of inorganic mercuric sulphide |
| 28 | Mexican folk remedy | powder | N/A | imported | lead | N/A | Lead | N/A |
| 29 | Ayurvedic medicine | powder | N/A | imported | lead | N/A | Lead | inorganic lead content of the brown powder: 19% by weight |
| 30 | Chinese herbal medicine (TCM) | powder | Chinese herbalist | imported | lead; traces of arsenic | N/A | Lead | tea contained 301 mg/l of lead and 64 µg/l of arsenic |
| 31 | Oriental medicine | N/A (p.o.) | Hakim | imported | arsenic, mercury; traces of lead & antimony | N/A ("mercury was in its insoluble form and was probably poorly absorbed") | / | As 7,54 mg/g, Hg 340 mg/g, Pb 0,012 mg/g, Sb 0,224 mg/g |
| 32 | Oriental medicine | N/A (p.o.) | Hakim | imported | arsenic, mercury, lead; traces of antimony | N/A ("mercury was in its insoluble form and was probably poorly absorbed") | " | As 686 mg/g, Hg 69,5 mg/g, Pb 64,43 mg/g, Sb 0,307 mg/g |
| 33 | Oriental medicine | N/A (p.o.) | N/A | N/A | arsenic, mercury | N/A | " | As 370 mg/g, Hg 540 mg/g |
| 34 | Oriental medicine | N/A (p.o.) | N/A | N/A | arsenic, lead; traces of antimony | N/A | " | As 8,87 mg/g, Pb 18,66 mg/g, Sb 0,5 mg/g |
| 35 | Oriental medicine | N/A (p.o.) | N/A | N/A | lead | N/A | " | Pb 167 mg/g |
| 36 | Indian traditional medicine | tablet | received from a family member | yes | lead | N/A | Lead | lead content (% by weight): 10%; weight of 1 tablet: 180 mg |
| 37 | Indian traditional medicine | powder | Hakim | imported | lead | N/A | " | lead content (% by weight): 55% |
| 38 | Indian traditional medicine | tablet, powder | N/A | imported | lead; traces of mercury | N/A | " | lead content (% by weight): 6%; weight of 1 tablet: 75 mg; traces of mercury |
| 39 | Traditional medicine | powder | healer | imported | lead; small amounts of aluminium & tin | N/A | " | lead content (% by weight): 12%; it also contained 0,1% aluminium and 0,5% tin by weight |
| 40 | Traditional medicine | powder, tablet | Hakim | imported | lead; traces of arsenic | N/A | " | lead content (% by weight) of one of the powders: 60%; trace amounts of arsenic (0,08% by weight) |
| 41 | Chinese herbal medicine (TCM) | powder, N/A | N/A | N/A | lead | N/A | Lead | lead concentration in the Cordyceps powder: 414 µg/g |
| 42 | Chinese herbal medicine (TCM) | powder | N/A | N/A | lead | N/A | " | lead content in the herbal medicine was as high as 20.000 µg lead per g of Cordyceps powder |
| 43 | Chinese herbal medicine (TCM) | powder | N/A | N/A | lead | N/A | " | lead content in the herbal medicine was as high as 20.000 µg lead per g of Cordyceps powder |
| 44 | Ayurvedic medicine | capsule | N/A | imported | lead | N/A | Lead | lead content: 56.340-72.990 µg Pb/g; 22.135-29.900 µg Pb/capsule or tablet |
| 45 | Ayurvedic medicine | capsule | N/A | imported | lead | N/A | " | lead content: 56.340-72.990 µg Pb/g; 22.135-29.900 µg Pb/capsule or tablet |
| 46 | Ayurvedic medicine | capsule | N/A | imported | lead | N/A | " | lead content: 56.340-72.990 µg Pb/g; 22.135-29.900 µg Pb/capsule or tablet |
| 47 | Ayurvedic medicine | capsule | N/A | imported | lead | N/A | " | lead content: 56.340-72.990 µg Pb/g; 22.135-29.900 µg Pb/capsule or tablet |
| 48 | Ayurvedic medicine | capsule | N/A | imported | lead | N/A | " | lead content: 56.340-72.990 µg Pb/g; 22.135-29.900 µg Pb/capsule or tablet |
| 49 | Ayurvedic medicine | capsule | N/A | imported | lead | N/A | " | 72.990 µg Pb/g or 29.900 µg Pb/capsule |
| 50 | Traditional medicine | powder | traditional healer | imported | lead | N/A | Lead | powder contained 12% lead by weight |
| 51 | Indian herbal medicine | tablet | "by a person whom the patient had consulted for the treatment of his diabetes" | imported | lead | N/A | Lead | each tablet contained 10 mg of lead |
| 52 | Ayurvedic medicine | paste, N/A | traditional Ayurvedic medical centre | yes | lead; amount of arsenic & silver not measured | N/A | Lead | lead content of the metallic paste: 238 µg/g; it was assumed that the patient additionally ingested lead with the other ayurvedic drugs, but these were used up |
| 53 | Asian remedy alternative medicine (produced in China) | pill | N/A | yes | lead | N/A | Lead | lead content of the red pills: 3,5 ppm in pills from bottle A and 1,2 ppm in pills from bottle B (laboratory results from CDPH State Laboratory); laboratory results from New York showed lead in amounts of 12.5 ppm in pills from bottle A and 4.5 ppm in pills from bottle B |
| 54 | Ayurvedic medicine | N/A (p.o.) | N/A | imported | lead | N/A | Lead | lead concentration in one preparation: 31 mg/kg |
| 55 | Chinese herbal medicine (TCM) | spray (p.o.) | pharmacist | no | mercury | mercury mainly in inorganic form; methyl-mercury constituted 2% of total mercury | Mercury | mercury content of the spray: 878 ppm (2% methylmercury & 98% inorganic mercury) |
| 56 | Indian herbal vitamin | tablets | traditional medicine healer | imported | lead, mercury | N/A | Lead | tablet 1: 35.300 mg/kg lead, 12.800 mg/kg mercury; tablet 2: 5.780 mg/kg lead, 65,5 mg/kg mercury; tablet 3: 54,8 mg/kg lead, 3 mg/kg mercury; tablet 4: 72 mg/kg lead, 3,8 mg/kg mercury |
| 57 | herbal medicine | liquid | Indian doctor | yes | arsenic | N/A | Arsenic | N/A |
| 58 | Indian ethnic remedy | pill, powder | Indian healer | imported | arsenic | N/A | Arsenic | arsenic concentrations were 184 mg/g in one sample and 0,01 mg/g, 0,11 mg/g, and 0,30 mg/g in the other three |
| 59 | Indian herbal medicine | N/A (p.o.) | N/A | yes | lead | N/A | / | N/A |
| 60 | Chinese herbal medicine (TCM) | pill | Chinese medicine TCM practitioner | no | lead | N/A | Lead | N/A |
| 61 | Chinese herbal medicine (TCM) | pill | Chinese medicine TCM practitioner | no | lead | N/A | " | N/A |
| 62 | Chinese herbal medicine (TCM) | pill | Chinese medicine (TCM) practitioner | no | lead | N/A | " | N/A |
| 63 | Indian herbal medicine | powder | N/A | yes | lead | N/A | Lead | N/A (patient refused to provide a sample) |
| 64 | Mexican folk remedy | powder | N/A | imported | lead | greta (PbO) | Lead | lead content: 777.000 ppm |
| 65 | Mexican folk remedy | powder | N/A | imported | lead | greta (PbO) | " | lead content: 777.000 ppm |
| 66 | Ayurvedic medicine | tablet | Ayurvedic doctor | yes | lead | N/A | Lead | high lead content of 2 of the tablet types: 45 mg lead per brown tablet (8,9%) and 23 mg lead per red tablet (4,5%) |
| 67 | / (Ayurvedic medicine) | / (indirect intake) | / (indirect intake) | / (indirect intake) | lead | N/A | " | elevated BLL due to lead intake by mother |
| 68 | Indian herbal medicine | pill | Ayurvedic health centre | imported | lead | N/A | Lead | one pill had a lead concentration of 50,4 mg/g; one pill weights 729 mg -> 37 mg per pill -> 2 pills daily for 9 months -> lead intake of about 20 g |
| 69 | homeopathic medication | liquid | homeopathic physician | no | arsenic | short-term use of Arsenic Bromide 1-X followed by long-term use of other arsenic-containing homeopathic preparations (Arsenic Alb. 1M) | Arsenic | The arsenic in the Arsenic Bromide 1-X preparation the patient had consumed was analyzed using a flow-injection hydride generation atomic absorption spectrometry (FI-HG-AAS) method (2,3). The concentration of arsenic in the prescribed medicine was 26 g/L. |
| 70 | homeopathic medication | powder | amateur homeopath | no | arsenic | Arsenic Sulfuratum Flavum-1-X; As3+ | " | The medicinal Arsenic S.F. 1-X was analyzed and found to contain 42 g of arsenic per kilogram of medication |
| 71 | homeopathic medication | liquid | homeopathic doctor | no | arsenic | Arsenic Bromide 1-X; As3+ | " | The Arsenic Bromide 1-X the patient consumed was in an alcohol solution and an analysis showed the arsenic content to be 53.5 g/L |
| 72 | Chinese herbal medicine (TCM) | N/A | N/A | N/A | arsenic | N/A | Arsenic | N/A |
| 73 | Ayurvedic medicine | powder, tablet | Ayurvedic physician | yes | lead | N/A | Lead | lead concentration: powders 12.000-17.000 ppm, tablets 60-100 ppm |
| 74 | Ayurvedic medicine | pill | Indian practitioner | yes | lead | N/A | " | lead concentration: 73.900 ppm lead in the pill taken 4 times daily and 21, 65 & 285 ppm lead in 3 other remedies |
| 75 | Ayurvedic medicine | powder, pill, syrup | Indian practitioner | yes | lead | N/A | " | lead concentration: one of the tablets contained 78.000 ppm lead; a second variety of pill contained 36 ppm lead |
| 76 | Ayurvedic medicine | tablet | N/A | N/A | lead | N/A | " | lead concentration: 14.000 ppm |
| 77 | Ayurvedic medicine | tablet | N/A | N/A | lead | N/A | " | lead concentration: 14.000 ppm |
| 78 | Ayurvedic medicine | pill, liquid | N/A | N/A | lead | N/A | " | lead concentration: pill 96.000 ppm, liquid 0 ppm |
| 79 | Ayurvedic medicine | pill | N/A | N/A | lead | N/A | " | lead concentration: 79.000 ppm |
| 80 | Ayurvedic medicine | N/A | N/A | N/A | lead | N/A | " | N/A |
| 81 | Ayurvedic medicine | N/A | N/A | N/A | lead | N/A | " | N/A |
| 82 | Ayurvedic medicine | N/A | N/A | N/A | lead | N/A | " | lead concentration: 44.000 ppm |
| 83 | Ayurvedic medicine | powder | N/A | N/A | lead | N/A | " | N/A |
| 84 | Ayurvedic medicine | N/A | N/A | N/A | lead | N/A | " | lead concentration: 22.700-26.700 ppm |
| 85 | Ayurvedic medicine | pill | pharmacy | yes | lead, mercury | N/A (inorganic lead?) | Lead | lead content: 67-82 g/kg; mercury content: 75 g/kg; total of 17,6-21,6 g lead intake |
| 86 | Herbal medicine | pill | clergyman | N/A | lead | N/A | Lead | lead content: 2.612 μg/g |
| 87 | Herbal medicine | pill | clergyman | N/A | lead | N/A | " | lead content: 2.612 μg/g |
| 88 | Ayurvedic medicine | pill | internet | imported | lead; traces of arsenic & mercury | N/A | Lead | lead content: 15 mg/kg, 16.000 mg/kg and 85 mg/kg |
| 89 | Ayurvedic medicine | tablet | N/A | no | lead | N/A | Lead | N/A |
| 90 | Ayurvedic medicine | powder | self-medication | imported | lead, mercury; small amounts of manganese | N/A (inorganic lead?) | Lead | powder 4: lead content 119.699 µg/g, manganese content: 1.209 µg/g; powder 2: mercury content 165.575 µg/g |
| 91 | Ayurvedic medicine | N/A | N/A | no | lead | N/A | Lead | N/A |
| 92 | Indian herbal medicine | powder, tablet | traditional practitioner | N/A | lead | N/A | Lead | lead content: 160 µg/g in brown powder, 2.300 µg/g in another brown powder & 35 µg/g in black tablets |
| 93 | Herbal medicine | N/A | N/A | yes | lead | N/A | / | N/A (lead & mercury both exceeding the acceptable levels for human ingestion) |
| 94 | Omani traditional medicine | N/A | N/A | no | lead | high content of inorganic lead (20.2%) | Lead | high content of lead (20.2%) |
| 95 | Chinese herbal medicine | p.o. (not further specified) | Chinese herbalist | imported | arsenic | inorganic arsenic | Arsenic | N/A |
| 96 | Ayurvedic medicine | pill | Indian healer | imported | lead | N/A | Lead | orange-red pills: 31 mg lead per tablet |
| 97 | Ayurvedic medicine | pill | Indian healer | yes | lead | N/A | " | orange-red pills: 28,9 mg lead per tablet |
| 98 | Indian herbal medicine | syrup | traditional practitioner | no | lead | N/A | Lead | N/A (patient had consumed the entire syrup given to him) |
| 99 | Ayurvedic medicine | globules, tablets, liquid, powder | Indian practitioner | no | arsenic | N/A (inorganic arsenic?) | Arsenic | arsenic was detected in all 8 preparations: 5-248 mg/l (5, 6, 35, 55, 79, 81, 126 & 248 mg/l) |
| 100 | Thai traditional medicine | powder | over the counter (shop) | imported | lead; small amounts of mercury | N/A | Lead | lead content: 109,000 ppm (>10% product weight); mercury was detected at 17 ppm |
| 101 | Ayurvedic medicine | tablet | N/A | imported | lead | N/A | Lead | lead content: 236,7 µmol (49.000 µg) lead per tablet |
| 102 | Ayurvedic medicine | N/A (p.o.) | received from a friend | imported | lead | N/A | Lead | Garbhpal ras: lead content 3.000 µg/dl |
| 103 | / (Ayurvedic medicine) | / (indirect intake) | / (indirect intake) | / (indirect intake) | lead | N/A | " | / (Garbhpal ras: lead content 3.000 µg/dl) |
| 104 | Ayurvedic medicine | pellet (tablet) | Ayurvedic practitioner | no | lead | N/A | Lead | 2 of the 5 tablets had a lead content of 5,5 mg/g and 8,0 mg/g; the other 3 had a lead content of between 0,15 and 3,0 mg/g |
| 105 | Indian traditional medicine | N/A (p.o.) | quack | no | lead | N/A | Lead | N/A |
| 106 | Ayurvedic medicine | powder | N/A | no | lead | N/A | / | N/A (Qualitative chemical analysis of the Ayurvedic powder was done which revealed very high lead levels) |
| 107 | Chinese herbal medicine (TCM) | N/A (p.o.) | N/A | N/A | cadmium | N/A | Cadmium | N/A |
| 108 | Ayurvedic medicine | pill, powder | over the counter (shop) | no | arsenic, mercury, lead; traces of cadmium | N/A | Lead | 75% of the products contained high levels of lead, arsenic and mercury in magnitudes higher than the daily permissible limits |
| 109 | Ayurvedic medicine | powder | local Vaidya | no | lead | N/A | " | Gulkand (Semisolid): 11,798 µg/g Pb; arsenic and mercury concentrations were below the permissible limits |
| 110 | Ayurvedic medicine | pill | Ayurvedic physician | no | lead, mercury; small amounts of arsenic; traces of cadmium | N/A | " | Chandraprabha Vati: 70 ng/g Cd, 0,96 µg/g As; EVR Tablet-Unja: 36044,07 µg/g Pb, 521,75 µg/g As, 2244,38 µg/g Hg |
| 111 | Ayurvedic medicine | N/A | N/A | imported | lead; small amounts of arsenic & mercury | N/A | Lead | lead (1200 ppm), arsenic (790 ppm) and mercury (62 ppm) |
| 112 | Ayurvedic medicine | tablet, N/A | N/A | yes | lead | N/A | Lead | Vatyog tablet tested contained 448μg of lead; Gandharvahastadi was not available for analysis |
| 113 | Ayurvedic medicine | capsule, sphere, tablet | over the counter (market) | yes | lead | N/A | / | lead concentrations detected in the herbal preparations were 286 mg/g in the red capsules, 61 mg/g in the dark brown tablets and 0,001 mg/g in the golden spheres and dark brown spheres; the estimated intake in the patient was 40 µg/kg/week |
| 114 | Ayurvedic medicine | pill | pharmacy | yes | lead | N/A | Lead | lead content: 21,5 mg per pill; the pills contain approximately 3.5% lead by weight or 35,000 μg/g |
| 115 | Ayurvedic medicine | pill | pharmacy | yes | lead | N/A | " | lead content: 21,5 mg per pill; the pills contain approximately 3.5% lead by weight or 35,000 μg/g |
| 116 | Ayurvedic medicine | pill | pharmacy | yes | lead | N/A | " | lead content: 21,5 mg per pill; the pills contain approximately 3.5% lead by weight or 35,000 μg/g |
| 117 | Ayurvedic medicine | pill | ordered from India | imported | lead; traces of arsenic, chromium & mercury | inorganic lead (?) | / | concentrations up to 7.3 mg of lead per pill and traces of arsenic, chromium and mercury were found |
| 118 | Chinese herbal medicine (TCM) | powder | N/A | N/A | lead | N/A | Lead | lead content: 80.309,95 µg/g |
| 119 | Ayurvedic medicine | tablet | sent from India | imported | lead | N/A | Lead | lead content of the tablets: 5.8-6.2% lead per dry weight |
| 120 | Herbal medicine | N/A | N/A | N/A | arsenic | arsenic compounds in urine: Arsenite (AsIII), monomethyl-arsonic acid (MMA), dimethylarsinic acid (DMA) and arsenobetaine (AsBe) | Arsenic | N/A (a sample of the relevant herbal medicine was not available) |
| 121 | Chinese herbal medicine (TCM) | powder (dermal) | purchased in hometown | no | lead | mainly lead tetraoxide (Pb3O4) | Lead | lead concentrations of 214.000 mg/kg -> approximately 5000 times above the limit allowed by the National Cosmetic Hygienic Standard of China, which permits lead content below 40 mg/kg of total weight |
| 122 | Chinese herbal medicine (TCM) | powder (dermal) | purchased in hometown | no | lead | mainly lead tetraoxide (Pb3O4) | " | lead concentrations of 214.000 mg/kg |
| 123 | Ayurvedic medicine | capsule | Indian practitioner | yes | lead | N/A | Lead | Pregnita contained 1,2% lead |
| 124 | Ayurvedic medicine | tablet | bought in New York City (made in India) | imported | lead, mercury; small amounts of arsenic | N/A | " | Vatvidhwansan Ras contained 2% lead, 1,5% mercury and 130 ppm arsenic; Kankayan Bati (Gulma) contained 12 ppm lead, 35 ppm mercury, and 9,5 ppm arsenic |
| 125 | Ayurvedic medicine | tablet | Ayurvedic practitioner | imported | lead, mercury; small amounts of arsenic | N/A | " | Garbhapal Ras contained 2,2% lead, 1,9% mercury & 410 ppm arsenic |
| 126 | Ayurvedic medicine | capsule | Ayurvedic practitioner | yes | lead, mercury; small amounts of arsenic | N/A | " | Ovarin contained 1,2% lead, 1.000 ppm arsenic & 1,8% mercury |
| 127 | Ayurvedic medicine | capsule | Ayurvedic practitioner | yes | lead, mercury; small amounts of arsenic | N/A | " | Ovarin contained 2,4% lead, 7% mercury & 100 ppm arsenic; Garbha Dharak Yog contained 10% mercury, 140 ppm arsenic & 110 ppm lead; Laxmana Louh contained 180 ppm lead, 120 ppm mercury & 12 ppm arsenic; Garbha Chintamani Ras (Vrihat) (Swarna Yukt) contained 5,2% arsenic & 120 ppm lead; Pigmento contained 2,9% mercury, 27 ppm arsenic & 7,3 ppm lead |
| 128 | Ayurvedic medicine | tablet | Ayurvedic practitioner | yes | lead, mercury; small amounts of arsenic | N/A | " | Garbhapal Ras (one of the medications) was found to contain 1,5% lead, 0,44% mercury & 81 ppm arsenic |
| 129 | Ayurvedic medicine | powder | N/A | yes | lead | N/A | Lead | lead content: 62% by weight |
| 130 | Ayurvedic medicine | powder | locally made in India | imported | lead | N/A | Lead | lead concentration: 36.000 µg/g |
| 131 | Ayurvedic medicine | capsule | N/A | no | lead | N/A | " | The ayurvedic medication was subjected to chemical analysis for lead content, and it contained 16.307,27 +/- 447 µg lead per capsule |
| 132 | Chinese folk medicine | capsule | N/A | N/A | arsenic | N/A | Arsenic | arsenic content: 0,052 mg/capsule |
| 133 | Chinese herbal medicine (TCM) | ointment, powder | N/A | N/A | lead; small amounts of arsenic & mercury | N/A | Lead, mercury, arsenic | 2 red herbal ointments contained lead 166.700 ppm and mercury 24,5 ppm, and lead 12.200 ppm and mercury 12,5 ppm; one deep blue herbal powder contained lead 71,9 ppm, arsenic 13,3 ppm & mercury 4,7 ppm |
| 134 | Chinese herbal medicine (TCM) | patch | N/A | N/A | lead | N/A | " | lead content of the herbal patch: 517 mg/g (51,7% lead by weight) |
| 135 | Ayurvedic medicine | tablet | N/A | yes | lead | N/A | Lead | lead concentration: Jambrulin 14,8 mg/kg, Himalaya Liv 52 DS 2,7 mg/kg & Neem Guard 4,4 mg/kg -> the patient recalled taking these tablets consistently for the past 8 years, which equated to approximate cumulative exposure of 166 mg (56.8 µg/day) |
| 136 | Ayurvedic medicine | N/A | traditional healer | no | mercury | N/A | Mercury | mercury content: 2066,6 ppm |
| 137 | Ayurvedic medicine | N/A | N/A (he bought it while traveling in India) | yes | lead, arsenic | N/A | Lead | lead concentration: 19.400 mg/kg; arsenic concentration: 1.430 mg/kg |
| 138 | Chinese herbal medicine (TCM) | ointment, N.A. (p.o.) | Chinese medicine practitioner (TCM practitioner) | no | arsenic | arsenic sulfide (Realgar) -> with an active ingredient of arsenic sulfide compound (As4S4) | Arsenic | very high concentration of arsenic in 3 unlabeled realgar-containing ointments (45427, 5512 & 4229 ppm) |
| 139 | Bhutanese traditional medicine | pellet | N/A | yes | lead | N/A | Lead | high level of lead on the red paint surrounding the pellets (1,4 mg lead in each pellet) |
| 140 | Chinese herbal medicine (TCM) | N/A | N/A | N/A | arsenic | N/A (inorganic arsenic?) | Arsenic | the arsenic level of the herbal medicine was 52,040 mg/kg |
| 141 | herbal medicine | N/A | N/A | N/A | lead | N/A | Lead | N/A |
| 142 | Ayurvedic medicine | N/A | N/A | no | lead | N/A | Lead | contained 30% w/w of lead (approximately 300.000 ppm) |
| 143 | Herbal medicine | tablet, paste, powder | Ayurvedic practitioner | imported | lead, mercury, arsenic, chromium | N/A | / | high levels of lead (4% w/w), mercury (12% w/w), arsenic and chromium |
| 144 | Ayurvedic medicine | N/A (p.o.) | N/A | no | arsenic | N/A | Arsenic | arsenic concentration >100 µg/kg |
| 145 | Ayurvedic medicine | N/A | Ayurvedic practitioner | no | arsenic | N/A | " | N/A (unable to procure the ayurvedic medication he had been taking) |
| 146 | Ayurvedic medicine | N/A (p.o.) | N/A | no | arsenic | N/A | " | arsenic concentration >100 µg/kg |
| 147 | Ayurvedic medicine | tablets | "esthetic and therapy center" (Ayurvedic center, same center as in the other case) | imported | lead | N/A | Lead | Lead concentrations: 2.003 µg lead per g tablet (Mahavatvidhwansa Rasa); other drugs also contained lead, but in smaller amounts, raging from 4 to 86 µg/g tablet |
| 148 | Ayurvedic medicine | pills | "esthetic and therapy center" (Ayurvedic center, same center as in the other case) | imported | lead | N/A | " | Lead concentration: 19.650 µg lead per g of tablet |
| 149 | Chinese herbal medicine (TCM) | pill, powder | Oriental University Hospital and Oriental Clinic | N/A | lead | N/A | Lead | lead content: 25,229 mg/g and 10,269 mg/g were detected in 2 types of powders, and 0,2327 µg/g and 0,3842 µg/g in 2 types of pills |
| 150 | Ayurvedic medicine | powder | received from a family member | yes | lead, mercury, iron; small amounts of gold, calcium, copper and arsenic | N/A | Lead | the black powder contained approximately 2.144 ppm lead, 16.829 ppm mercury and 41.900 ppm iron |
| 151 | Ayurvedic medicine | capsule | over the counter (shop or market) or by a homeopathic practitioner | imported | lead | N/A | Lead | lead content: 125.235 μg/g (average 71.208 µg/capsule); at the maximum recommended dose, participants would have ingested around 284.832 μg of lead per day |
| 152 | Ayurvedic medicine | capsule | over the counter (shop or market) or by a homeopathic practitioner | imported | lead | N/A | " | lead content: 125.235 μg/g (average 71.208 µg/capsule); at the maximum recommended dose, participants would have ingested around 284.832 μg of lead per day |
| 153 | Ayurvedic medicine | capsule | over the counter (shop or market) or by a homeopathic practitioner | imported | lead | N/A | " | lead content: 125.235 μg/g (average 71.208 µg/capsule); at the maximum recommended dose, participants would have ingested around 284.832 μg of lead per day |
| 154 | Ayurvedic medicine | capsule | over the counter (shop or market) or by a homeopathic practitioner | imported | lead | N/A | " | lead content: 125.235 μg/g (average 71.208 µg/capsule); at the maximum recommended dose, participants would have ingested around 284.832 μg of lead per day |
| 155 | Ayurvedic medicine | capsule | over the counter (shop or market) or by a homeopathic practitioner | imported | lead | N/A | " | lead content: 125.235 μg/g (average 71.208 µg/capsule); at the maximum recommended dose, participants would have ingested around 284.832 μg of lead per day |
| 156 | Ayurvedic medicine | capsule | over the counter (shop or market) or by a homeopathic practitioner | imported | lead | N/A | " | lead content: 125.235 μg/g (average 71.208 µg/capsule); at the maximum recommended dose, participants would have ingested around 284.832 μg of lead per day |
| 157 | Ayurvedic medicine | capsule | over the counter (shop or market) or by a homeopathic practitioner | imported | lead | N/A | " | lead content: 125.235 μg/g (average 71.208 µg/capsule); at the maximum recommended dose, participants would have ingested around 284.832 μg of lead per day |
| 158 | Ayurvedic medicine | capsule | over the counter (shop or market) or by a homeopathic practitioner | imported | lead | N/A | " | lead content: 125.235 μg/g (average 71.208 µg/capsule); at the maximum recommended dose, participants would have ingested around 284.832 μg of lead per day |
| 159 | Ayurvedic medicine | tablet | internet | imported | lead; small amounts of arsenic & thallium; traces of cadmium, chromium & aluminum | N/A | Lead | BVCR contained 16,4 mg/kg (0,2%) lead, MG contained 48.700 mg/kg (4,9%) lead; both supplements also contained trace amounts of cadmium, chromium & aluminum, as well as substantial amounts of arsenic (3.830 mg/kg in MG) and thallium (14,7 mg/kg in MG and 17,2 mg/kg in BVCR) |
| 160 | Ayurvedic medicine | tablets | purchased on his business trips to India | yes | lead | N/A | Lead | one 0,728g tablet contained 11% (80 mg) lead, 5,2% zinc, 4% iron, 0,8% tin and 0,1% aluminum |
| 161 | Ayurvedic medicine | powder | N/A | no | many heavy metals, but all in relatively small amounts: lead, mercury, manganese, nickel | N/A | / | manganese 122,3±4,8 µg/g; nickel 22,7±0,7 µg/g; lead: 25,9±4,3 µg/g; mercury 124,3±1,5 µg/g |
| 162 | traditional Chinese medicine (realgar) | ointment | N/A | no | arsenic | realgar | Arsenic | N/A |
| 163 | Ayurvedic medicine | tablet | N/A | yes | lead | N/A | Lead | N/A (a peak with lead was found) |
| 164 | Chinese folk medicine | pill | traditional healer | no | lead | lead sulfide | Lead | 5 homemade pills contained 0,3g lead sulfide -> 110,000 mg/kg lead (other measure method: 100,000 mg/kg = 10% lead) |
| 165 | Asian alternative medicine | globules | N/A | yes | lead; small amounts of mercury | N/A | Lead, mercury | 3 different NHPs: 45 μg/g, 53.000 μg/g & 28 μg/g lead (for morning, midday and evening globules, respectively); 15,72 μg/g mercury in the “evening globules” |
| 166 | Chinese herbal medicine (TCM) | powder | N/A | no | lead | N/A | / | lead content in Jineijin + Squama Mantis: 3.389 mg/kg |
| 167 | Indian alternative medicine | N/A | N/A | no | thallium | N/A | Thallium | N/A |
| 168 | Chinese herbal medicine (TCM) | pill | Chinese medicine clinic | N/A | lead | N/A | Lead | lead content: up to 90 ppm in each pill |
| 169 | Ayurvedic medicine | powder | Ayurvedic practitioner (quack) | no | lead | N/A | Lead | lead concentration: 638,19 g/kg |
| 170 | Ayurvedic medicine | tablet | Ayurvedic practitioner | yes | lead | N/A | Lead | lead content of the different tablets (µg/mg): 24,3 & 27 & 9,85 |
| 171 | Chinese herbal medicine (TCM) | pill | Chinese medicine practitioner | no | lead | N/A | Lead | lead content: >4000 mg/kg |
| 172 | Chinese herbal medicine (TCM) | pill | Chinese medicine practitioner | no | lead | N/A | " | lead content: >4000 mg/kg |
| 173 | Malaysian folk diaper powder | powder (dermal) + probably oral intake via hand-mouth transfer | over the counter (store/pharmacy) | imported | lead; small amounts of antimony, arsenic, bismuth & thallium | primarily lead oxide; mainly lead monoxide (litharge); lead phosphate-chloride (pyromorphite) & lead-copper-chromate-phosphate; include kyanite (Al2SiO5), magnesite (MgCO3), and talc (Mg3Si4O10(OH)2) | Lead | powder contains 62-64% lead by weight (primarily lead oxide, 620.000-639.500 ppm) and elevated antimony (1005 ppm), arsenic (55 ppm), bismuth (110 ppm), and thallium (31 ppm) |
| 174 | Ayurvedic medicine | powder | N/A | no | all in relatively small amounts: barium, chromium, lead, vanadium, arsenic, thallium, cadmium, cobalt | N/A | / | barium 8,25 mg/kg; chromium: 2,85 mg/kg; lead: 0,73 mg/kg ; vanadium: 0,47 mg/kg; arsenic: 0,25 mg/kg; thallium: 0,13 mg/kg; cadmium: 0,083 mg/kg, cobalt: 0,13 mg/kg; beryllium & mercury below detection limit |
| 175 | Chinese herbal medicine (TCM) | liquid (nasal spray) | Chinese medicine practitioner (TCM practitioner) | no | lead | N/A | Lead | lead concentration: 148.000 mg/kg (14,8%) |
| 176 | Chinese herbal medicine (TCM) | liquid (nasal spray) | Chinese medicine practitioner (TCM practitioner) | no | lead | N/A | " | lead concentration: 223.000 mg/kg (22,3%) |
| 177 | Chinese herbal medicine (TCM) | liquid (nasal spray) | Chinese medicine practitioner (TCM practitioner) | no | lead | N/A | " | lead concentration: 334.000 mg/kg (33,4%) |
| 178 | Ayurvedic medicine | N/A | Ayurvedic practitioner | no | lead | N/A | Lead | N/A (toxicological analysis showed the presence of a high concentration of lead) |
| 179 | Chinese dietary supplement | N/A | N/A | N/A | lead | N/A | Lead | N/A |
| 180 | Traditional medicine | pill | N/A | imported | arsenic | N/A | Arsenic | 100 pills (equivalent to 30g) were tested -> arsenic concentration: 3,5 g/kg |
| 181 | Ayurvedic medicine | tablet | over the counter (probably in a pharmacy) | no | lead | N/A | Lead | lead content of 2.87 and 2.29 μg/g |
| 182 | Indian herbal medicine | N/A | indigenous medical practitioner | no | lead | N/A | Lead | N/A |
| 183 | Traditional herbal medicine | "ball" (p.o.) | homemade | imported | arsenic | inorganic arsenic | Arsenic | N/A |
| 184 | Chinese folk medicine | ointment | quack doctor | no | arsenic | Realgar: 90% As4S4; mainly arsenic disulfide (As2S2) or tetra-arsenic tetra-sulfide (As4S4); uncertain amount of other inorganic arsenic impurities, such as arsenate (As2O5) and arsenite (As2O3) | Arsenic | arsenic concentration in the ointment: about 6% (6,01x10^4 µg/ml) |
| 185 | Ayurvedic medicine | N/A (p.o.) | N/A | N/A | lead, arsenic, mercury | N/A | Lead, arsenic | contained high levels of Ars (50 to 290 mg/kg), Pb (28 to 12,000 mg/kg) and mercury (5 to 75,000 mg/kg) |
| 186 | Traditional herbal medicine | N/A (p.o.) | he bought the ingredients himself at various traditional markets and made the medicine himself | no | lead | N/A | Lead | N/A |
| 187 | natural product-derived drug | capsule | N/A | no | lead | N/A | Lead | 5 samples were analyzed, and the arithmetic average of the lead content was 2,547 ± 1,821.9 ppm: 1. 911 ppm, 2. 4411 ppm; 3. 1477 ppm; 4. 5083 ppm; 5. 853 ppm |
| 188 | natural product-derived drug | capsule | N/A | no | lead | N/A | " | 6 samples were analyzed, and the arithmetic average of the lead content was 2,547 ± 1,821.9 ppm: 1. 911 ppm, 2. 4411 ppm; 3. 1477 ppm; 4. 5083 ppm; 5. 853 ppm |
| 189 | natural product-derived drug | capsule | N/A | no | lead | N/A | " | 7 samples were analyzed, and the arithmetic average of the lead content was 2,547 ± 1,821.9 ppm: 1. 911 ppm, 2. 4411 ppm; 3. 1477 ppm; 4. 5083 ppm; 5. 853 ppm |
| 190 | natural product-derived drug | capsule | N/A | no | lead | N/A | " | 8 samples were analyzed, and the arithmetic average of the lead content was 2,547 ± 1,821.9 ppm: 1. 911 ppm, 2. 4411 ppm; 3. 1477 ppm; 4. 5083 ppm; 5. 853 ppm |
| 191 | natural product-derived drug | capsule | N/A | no | lead | N/A | " | 9 samples were analyzed, and the arithmetic average of the lead content was 2,547 ± 1,821.9 ppm: 1. 911 ppm, 2. 4411 ppm; 3. 1477 ppm; 4. 5083 ppm; 5. 853 ppm |
| 192 | natural product-derived drug | capsule | N/A | no | lead | N/A | " | 10 samples were analyzed, and the arithmetic average of the lead content was 2,547 ± 1,821.9 ppm: 1. 911 ppm, 2. 4411 ppm; 3. 1477 ppm; 4. 5083 ppm; 5. 853 ppm |
| 193 | natural product-derived drug | capsule | N/A | no | lead | N/A | " | 11 samples were analyzed, and the arithmetic average of the lead content was 2,547 ± 1,821.9 ppm: 1. 911 ppm, 2. 4411 ppm; 3. 1477 ppm; 4. 5083 ppm; 5. 853 ppm |
| 194 | Ayurvedic medicine | N/A (p.o.) | over the counter | no | lead | N/A | Lead | N/A |
| 195 | Indian herbal health supplement | N/A | N/A | N/A | lead | N/A | Lead | lead concentration: supplement #11 had 9265,97 ppm; #1: 1,59 ppm; #2: 1,89 ppm; #4: 0,94 ppm; #7: 0,80 ppm; #12: 0,84 ppm |
| 196 | Indian traditional medicine | powder | quack | no | lead | N/A | Lad | N/A |
| 197 | Ayurvedic medicine | powder | Ayurvedic practitioner | yes | lead, mercury; small amounts of arsenic, copper, manganese, iron, chromium & nickel | inorganic mercury, … | Lead | sample 1 (mg/kg): 2,31 lead, 2,54 mercury, 0,35 arsenic, 3,96 copper, 1,14 chromium, 26,34 manganese, 288,04 iron, 0,66 nickel; sample 2A (mg/kg): 12.638,54 lead, 89.076,70 mercury, 134,94 arsenic, 1.511,04 copper, 8,86 chromium, 146,74 manganese, 14.990,34 iron, 7,24 nickel; sample 2B (mg/kg): 23.043,02 lead, 126.640,27 mercury, 103,98 arsenic, 2.168.79 copper, 7,68 chromium, 172,53 manganese, 16498,80 iron, 8,02 nickel; sample 2C (mg/kg): 21.352,97 lead, 145.710,64 mercury, 122,30 arsenic, 2.356,80 copper, 12,04 chromium, 149,75 manganese, 15.767,59 iron, 9,21 nickel |
| 198 | Ayurvedic medicine | tablet | Ayurvedic practitioner | no | lead | N/A | Lead | N/A |
| 199 | Ayurvedic medicine | tablet | Ayurvedic practitioner | no | lead | N/A | " | N/A |
| 200 | Chinese herbal medicine (TCM) | N/A | temple | no | lead | N/A | Lead | N/A |
| 201 | Chinese herbal medicine (TCM) | N/A | temple | no | lead | N/A | " | N/A |
| 202 | Chinese herbal medicine (TCM) | N/A | temple | no | lead | N/A | " | N/A |
| 203 | Chinese herbal medicine (TCM) | powder, pill | N/A | no | lead | N/A | Lead | lead content in the pills: 8.421 μg/g; lead content in the powder: 5,85 µg/g |
| 204 | Ayurvedic medicine | tablets | naturopath | imported | lead | N/A | Lead | 0.96% lead by weight |
| 205 | Ayurvedic medicine | capsule | purchased online | imported | lead | N/A | Lead | A capsule was found containing 12 mg of lead |
| 206 | Ayurvedic medicine | liquid, powder, granula, tablet | Ayurveda Chikitsalayam and Research Center | no | arsenic | N/A | / | extremely high levels of arsenic in multiple herbal formulations; 2.460,6 mg/kg arsenic in the 9 medications |
| 207 | Ayurvedic medicine | N/A | N/A | no | arsenic | Arsenic Disulphide and Arsenic Trioxide | Arsenic | N/A |
| 208 | Ayurvedic medicine | powder | N/A | yes | lead | N/A | Lead | N/A |
| 209 | Ayurvedic medicine | pill | practitioner | imported | lead | N/A | Lead | 11 pills contained lead levels greater than the detection limit and 1 pill contained 129 000 μg/g of lead (about 13% lead by weight). 4 additional pills contained 7900—33 000 μg/g of mercury. |
| 210 | Ayurvedic medicine | tablets | holistic energy healer | imported | arsenic, mercury, lead | inorganic arsenic | Arsenic | 8 out of 10 contained arsenic, 9 out of 10 mercury, and 7 out of 10 lead. 3 (#1, 4, 7) had high heavy metal concentrations, #1 9.3% arsenic by weight.; #1 was labeled to contain 20 mg “shuddh hartal” = translates to “pure arsenic” -> 16 mg arsenic per tablet |

*Tab. S2B: Raw data table of all patient cases – Kind of T&AM, Intake form, Prescribed/Procured by whom?, T&AM acquired while traveling or imported?, Contamination, Form of metal, Metals mentioned in the title, Drug formulation analysis for metals*

| **Case num-ber** | **Metal inake amount** | **Daily lead intake amount (mg)** | **Total lead intake amount (mg)** | **Intake duration** | **Latency period** | **Reason(s) for taking the medicine** | **Clinical symptoms** | **Lead ence-phalopathy** |
| --- | --- | --- | --- | --- | --- | --- | --- | --- |
| 1 | ~120 mg in 4 months (at least 64mg, but not more than 151mg) | 1.07 | 120 | sample 1: 1 year; sample 2: 1 month | 26 days | sexual difficulties (including failure to maintain an erection) | constipation, precordial pain, generalised abdominal pain, colicky loin pain, nausea & vomiting; he was apyrexial, obviously unwell, and irritable | no |
| 2 | 1800 mg lead in 4 months; 15mg lead daily | 15 | 1,800 | 4 months | 3,75 months (15 weeks) | minor ailments | fever, cough, anorexia and vomiting; immediately prior to admission he had his first attack of grand mal seizure (generalized tonic-clonic seizure); on physical examination he was pale and comatose with repeated tonic-clonic convulsions; generalized hypertonia and hyperreflexia | yes |
| 3 | sample 1: total intake of 200 mg elemental lead in 1 year; sample 2: total intake of 1.400 mg elemental lead in 1 month | sample 1: 0.55; sample 2: 46.67 -> 47.22 | 1,600 | N/A | N/A | chronic diarrhea | N/A | N/A |
| 4 | N/A | N/A | N/A | N/A | N/A | chronic diarrhea | seizures | yes |
| 5 | N/A | N/A | N/A | N/A | asymptomatic | high fever | asymptomatic | no |
| 6 | N/A | N/A | N/A | N/A | N/A | empacho | vomiting & diarrhea; stool was watery and green initially, but later contained blood; rectal temperature of 37,5°C | no |
| 7 | N/A | N/A | N/A | N/A | asymptomatic | improves blood circulation in patients recovering from cerebral thrombosis; relief from angina pectoris symptoms | no symptoms | no |
| 8 | N/A | N/A | N/A | N/A | N/A | improves blood circulation in patients recovering from cerebral thrombosis; relief from angina pectoris symptoms | chest discomfort, dizziness, dysesthesia in the upper and lower limbs, skin rash | no |
| 9 | N/A | N/A | N/A | N/A | asymptomatic | improves blood circulation in patients recovering from cerebral thrombosis; relief from angina pectoris symptoms | no symptoms | no |
| 10 | N/A | N/A | N/A | N/A | N/A | empacho | weakness, malaise, jaundice | no |
| 11 | 1.680 mg lead in 6 weeks; approximately 40 mg lead per day | 40 | 1,680 | N/A | N/A | empacho | N/A (probably asymptomatic) | no |
| 12 | N/A | N/A | N/A | 7 months | 6 months | N/A | developed normally until he was 8 months old, then became lethargic, less responsive and stopped crawling; within a few weeks, the behavioral abnormalities worsened, he refused bottle-feeding and began to have tremors; had seizures at his home, he became apneic | yes |
| 13 | N/A | N/A | N/A | 6 weeks | N/A (<6 weeks) | diabetes | weakness, malaise, nausea, abdominal pain | no |
| 14 | N/A | N/A | N/A | N/A | N/A | hemorrhoids | cramping abdominal pain | no |
| 15 | N/A | N/A | N/A | 3 weeks | 3 weeks intake, first symptoms 8 weeks after last intake | for abdominal colic and early passage of meconium after birth | she developed a generalized seizure, lasting about 1h; pallor; she showed marked irritability, but muscle tone and reflexes were normal | probably yes |
| 16 | N/A | N/A | N/A | N/A (case 2-6 of this article: periods varying from 2 days to 1 month) | N/A (case 2-6 of this article: periods varying from 2 days to 1 month) | for abdominal colic and early passage of meconium after birth | vomiting, constipation, drowsiness, irritability, seizure activity, hypertonia with exaggerated reflexes | probably yes |
| 17 | N/A | N/A | N/A | N/A (case 2-6 of this article: periods varying from 2 days to 1 month) | N/A (case 2-6 of this article: periods varying from 2 days to 1 month) | for abdominal colic and early passage of meconium after birth | vomiting, drowsiness, seizure activity, hypertonia with exaggerated reflexes | probably yes |
| 18 | N/A | N/A | N/A | N/A (case 2-6 of this article: periods varying from 2 days to 1 month) | N/A (case 2-6 of this article: periods varying from 2 days to 1 month) | for abdominal colic and early passage of meconium after birth | vomiting, constipation, irritability | no |
| 19 | approximately 562 mg lead in 9 weeks; powder 1: weekly lead intake about 62,4 mg/week; powder 2: 0,028 mg lead per week | 8.92 | 562 | N/A (case 2-6 of this article: periods varying from 2 days to 1 month) | N/A (case 2-6 of this article: periods varying from 2 days to 1 month) | for abdominal colic and early passage of meconium after birth | vomiting, drowsiness, irritability, seizure activity, hypertonia with exaggerated reflexes | probably yes |
| 20 | 4.200 mg lead in 1 month, 1500 mg arsenic in 1 month; approximately 140 mg lead per day and 50 mg arsenic per day | 140 | 4,200 | N/A (case 2-6 of this article: periods varying from 2 days to 1 month) | N/A (case 2-6 of this article: periods varying from 2 days to 1 month) | for abdominal colic and early passage of meconium after birth | constipation, abdominal pain | no |
| 21 | N/A | N/A | N/A | 8,5 weeks | N/A (<8,5 weeks) | powder 1: frequent regurgitation; powder 2: "to pacify him"; powder 3: constipation | repeated convulsions (status epilepticus), constipation; on admission, he was semiconscious, pale and afebrile | yes |
| 22 | 220,5-441 mg lead in 1 month; 7,35-14,7 mg lead per day | 11.06 | 330.75 | 1 month | a few days (<1 month), last intake 2 months before admission | haemorrhoids | malaise, severe difficulty walking, arthralgia, periorbital & ankle oedema; abdominal pain with diarrhoea; on examination she was languid, anaemic, icteric, had generalized abdominal tenderness, hyperaesthesia of her feet, tender patellae and toes, weakness and inability to walk | no |
| 23 | N/A | N/A | N/A | N/A | N/A | N/A | increasing abdominal pain, obstipation & weight loss | no |
| 24 | N/A | N/A | N/A | 1 month | N/A (<1 month) | psoriasis | leg pain (localized mainly in the heel), pain worsened and spread to involve more extensive areas of his leg; episode of severe chest pain; nausea; significant weight loss | no |
| 25 | 18018 mg lead, 579,6 mg arsenic & 2.520 mg mercury in 6 weeks; lead 429 mg/day, arsenic 13,8 mg/day, mercury at least 60 mg/day for about 6 weeks | 429.00 | 18,018 | 5 weeks | N/A (<5 weeks) | impotence | progressive general malaise, anorexia, vague abdominal discomfort & impaired taste | no |
| 26 | 28.350 mg arsenic in 4,5 months; packets each contained an average of 105 mg of inorganic arsenic trioxide -> 2 packets daily für 4,5 months | / | / | 6 weeks | no symptoms specified (<6 weeks) | back pain | N/A | N/A |
| 27 | 5.040-7.056 mg mercury in 4 weeks; 180-252 mg mercury per day | / | / | 6 weeks | 5 weeks | N/A | abdominal pain, anorexia, malaise, constipation, arthralgia; on examination he was pale and mildly icteric | no |
| 28 | N/A | N/A | N/A | 4,5 months | 1,5 months | atopic eczema | progressive weakness of his hands and legs associated with distal sensory disturbance; Mees' lines in the finger nails, hyperkeratosis of the soles of the feet; symmetrical wasting and weakness in the upper and lower limbs, all tendon reflexes were absent; he could not stand from a siting position without using his arms and walked with difficulty unsupported; peripheral sensorimotor neuropathy | no |
| 29 | N/A | N/A | N/A | 4 weeks | 1 week | eczema | poor appetite, weight loss, diarrhoea, sweating, tremorof the hands, paraesthesia in the extremities and face; neurological examination showed reduced pinprick sensation in both feet; fine tremor of the outstretched hands | no |
| 30 | estimated 4,7g lead and 1,1 mg arsenic in 5 weeks | 134.29 | 4,700 | ~1,5 years | asymptomatic (>1 year without symptoms) | laxative | no apparent clinical manifestations | no |
| 31 | N/A | N/A | N/A | 6 weeks | 4,5 weeks | diabetes | malaise, anorexia, upper abdominal pain; on examination he appeared unwell, clinically anaemic, icteric and pyrexial (37.4°C) | no |
| 32 | N/A | N/A | N/A | 5 weeks | 4 weeks | chronic cough, sputum production, dyspnea (25-year history of smoking) -> chronic obstructive pulmonary disease | abdominal colic (severe colicky pain), weight loss, anorexia, constipation, muscle pain (diffuse muscular discomfort), fatigue, decreased libido | no |
| 33 | N/A | N/A | N/A | N/A | N/A | eczema | insomnia, anorexia, diarrhoea, headache, limb pains, face numbness | no |
| 34 | N/A | N/A | N/A | N/A | N/A | eczema | limb weakness, sensory disturbance, Mees' lines, hyperkeratosis of the soles, peripheral polyneuropathy | no |
| 35 | N/A | N/A | N/A | N/A | N/A | N/A | nephrotic syndrome | no |
| 36 | N/A | N/A | N/A | N/A | N/A | N/A | N/A | N/A |
| 37 | N/A | N/A | N/A | N/A | N/A | N/A | pain, malaise | no |
| 38 | N/A | N/A | N/A | N/A | N/A | N/A | acute abdominal pain (plus recurrent abdominal pain since 4 years) | no |
| 39 | N/A | N/A | N/A | N/A | N/A | impotence | headache, nausea & malaise | no |
| 40 | N/A | N/A | N/A | N/A | N/A | enable to walk again (patient had a stroke causing left hemiparesis) | constipation, increasing leg weakness & depression; peripheral neuropathy in addition to the hemiparesis | no |
| 41 | N/A | N/A | N/A | N/A | N/A | for infertility | anorexia, nausea, vomiting, lower abdominal pain and constipation | no |
| 42 | N/A | N/A | N/A | 4 weeks | 2,5 weeks | impotence | generalized abdominal pain, vomiting & constipation | no |
| 43 | N/A | N/A | N/A | 6 months | asymptomatic (6 months without symptoms) | lower gastro-intestinal bleeding | asymptomatic | no |
| 44 | N/A | N/A | N/A | >1 year | 1 year | diabetes, hypertension | loss of appetite and general malaise | no |
| 45 | N/A | N/A | N/A | >1 year | asymptomatic (>1 year without symptoms) | N/A | asymptomatic | no |
| 46 | N/A | N/A | N/A | several days to several months (not further specified) | N/A (several days to several months) | N/A | colic or diffuse abdominal pain, musculoskeletal pain, nausea, weakness and loss of appetite, headache | no |
| 47 | N/A | N/A | N/A | several days to several months (not further specified) | N/A (several days to several months) | N/A | colic or diffuse abdominal pain, musculoskeletal pain, nausea, weakness and loss of appetite, headache | no |
| 48 | N/A | N/A | N/A | several days to several months (not further specified) | N/A (several days to several months) | N/A | colic or diffuse abdominal pain, nausea, weakness and loss of appetite | no |
| 49 | N/A | N/A | N/A | several days to several months (not further specified) | N/A (several days to several months) | N/A | colic or diffuse abdominal pain, musculoskeletal pain, constipation, nausea, weakness and loss of appetite | no |
| 50 | N/A | N/A | N/A | 7 days | a few days (<1 week) | rhinitis | feel dizzy, headache; poor appetite, mouth-bitterness, repeated vomiting, abdominal pain, intense joint pain, fatigue, lead line; intense joint pain and fatigue occurred causing him to be unable to walk by himself | no |
| 51 | approximately 4 g lead in 4 months | 43.84 | 4,000 | several days to several months (not further specified) | N/A (several days to several months) | N/A | colic or diffuse abdominal pain, musculoskeletal pain, constipation, nausea, weakness and loss of appetite | no |
| 52 | N/A | N/A | N/A | N/A (he had taken 81 capsules) | no symptoms specified | treat an allergy | N/A | N/A |
| 53 | N/A | N/A | N/A | 10 days | <10 days | rhinitis | severe abdominal colic, vomiting, constipation, fatigue | no |
| 54 | N/A | N/A | N/A | N/A | N/A | infertility | anorexia, nausea, vomiting, constipation and lower abdominal pain | no |
| 55 | N/A | N/A | N/A | 4 months | 2 months | posttraumatic arthralgias | diffuse pain (pain in shoulder, neck, low back, breasts, abdomen), knee & hip arthralgias, insomnia, irritability, paranoia, constipation, difficulty using her hands, small lead lines on the right and left mandibular bicuspids; pallor | no |
| 56 | 63g lead in 4 years (3 tablets/day) | 43.15 | 63,000 | 6 months | <6 months | improves blood circulation in patients recovering from cerebral thrombosis; relief from angina pectoris symptoms | headache, itching, chest discomfort, tongue numbness, depressive symptoms, left eyeball pain | no |
| 57 | N/A | N/A | N/A | >1 year | >1 year | infertility | abdominal pain, constipation, nausea, vomiting, fatigue; shortness of breath, headaches and tinnitus | no |
| 58 | N/A | N/A | N/A | 3 months | 11,5 weeks | diabetes (type 2) | history of epigastric pain (intermittent upper abdominal pain), pain in the right upper quadrant and constipation, low-grade fever (37,4-37,8°C) | no |
| 59 | N/A | N/A | N/A | 1-2 months | <1 month | unknown | weakness, dizziness, nausea and diffuse muscle pain (developed over the past few weeks) | no |
| 60 | N/A | N/A | N/A | 3-4 years | asymptomatic (3-4 years without symptoms) | menstrual cramps | no symptoms | no |
| 61 | N/A | N/A | N/A | for years (not further specified) -> one preparation contains lead -> she used this one for 6-8 months before the first symptoms | 6-8 months | N/A | severe colicky abdominal pain, vomiting, obstipation, weight loss, severe pain in the extremities, loss of concentration & short-term memory | N/A |
| 62 | N/A | N/A | N/A | 4 weeks | <4 weeks | mouth ulcers (oral ulceration) -> controlling pain and healing difficult mucosal wounds | motor and vocal tics (eye blinking, head turning, shoulder shrugging); irritable; he had been clearing his throat frequently; transient skin rash on his trunk | no |
| 63 | N/A | N/A | N/A | 4 years | N/A | to strengthen his brain (he was developmentally delayed) -> promote brain growth and improve his mental capabilities | on physical examination he was alert and active but nonverbal; he was able to stand with support but not ambulate and had no focal neurologic defects | no |
| 64 | N/A | N/A | N/A | 12 months | verrucous papules on her palms and soles had developed 3 years after she had been treated orally with a herbal solution for a period of 12 months | vitiligo ("white spot disease") | developed multiple verrucous papules on her palms and soles several years after receiving "herbal treatment''; multiple hyperkeratotic papules on her palms and soles, some of which were coalescing into large leathery plaques | no |
| 65 | N/A | N/A | N/A | 13-14 months | <13-14 months | congenital bilateral retinoblastma (specialists recommended right eye enucleation) -> attempt to avoid enucleation | the child became anorexic and restless, nausea, fatigue, paraesthesia, progressive weakness of the lower limbs; vomiting, cough, hoarseness, recurrent fever | no |
| 66 | N/A | N/A | N/A | N/A | N/A | for vague non-specific ailments | severe, diffuse abdominal pain, vomiting and diarrhoea, followed by constipation | no |
| 67 | indirect intake | / | / | 2 months | 7,5 weeks | acne | non-specific musculoskeletal pain; pallor | no |
| 68 | 20 g lead in 9 months | 73.06 | 20,000 | 6 months | asymptomatic (6 months without symptoms) | excrete the toxin of psychiatric medication from the body & stop her left groin pain | asymptomatic apart from non-specific malaise | no |
| 69 | It was calculated that she had consumed about 36 mg of arsenic (as As3+) during the week of November 1995 from Arsenic Bromide 1-X alone. The amount of arsenic in her other medications was not quantified. | 5.14 | 36 | N/A | N/A | aphrodisiac (increase libido) | anemic palpebral conjunctivae, lower abdominal pain and upper limb tremor | no |
| 70 | In 1 year, the patient could have ingested about 1.3g of arsenic | / | / | ~5 years | ~5 years | seizure disease | complaints of progressive, painless and non-cholestatic jaundice associated with loss of appetite and lethargy; on examination, the patient was conscious and oriented, without flapping tremors and had a hyperpigmented facial and upper body; ascites | no |
| 71 | It was estimated that the patient had consumed a total of 144 mg of arsenic (as As3+) over 6 days | / | / | N/A (<1 year) | N/A (<10 months) | eczematous dermatitis | glove-and-stocking paresthesia; loss of distal proprioception and a hyperesthetic response to pinprick; loss of dexterity, which initially manifested as difficulty in buttoning-unbuttoning a shirt -> this progressed to an inability to pick up small objects -> also began experiencing slippage of sandals while walking; multiple episodes of loose stools per day and abdominal pain along with significant weight loss; dark patches of hyperpigmentation, with hyperkeratotic scaly plaques on the extensor aspects of the forearms and legs; axonal sensorimotor polyneuropathy | no |
| 72 | N/A | N/A | N/A | 3 days | asymptomatic (3 days without symptoms) | treat a common cold | asymptomatic apart from common cold symptoms | no |
| 73 | N/A | N/A | N/A | many years (not further specified) | many years | generalized weakness | abdominal pain, jaundice, chronic polyarthralgia, nausea, loose bowel motion, yellowish discoloration of sclera, dark urine; long history of generalized weakness (musculoskeletal symptoms), headaches, recurrent dark urine and vague aches and pains; pale | no |
| 74 | N/A | N/A | N/A | N/A | N/A | stomachache or intestinal illness | N/A (identified during routine screening) | N/A |
| 75 | N/A | N/A | N/A | N/A | N/A | stomachache or intestinal illness | N/A (identified during routine screening) | N/A |
| 76 | N/A | N/A | N/A | periodically over the course of the past 9 years (also while being pregnant) | N/A | gastrointestinal complaint, periodically over the vourse of the past 9 years | at 30 weeks’ gestation, she presented with abdominal pain and a progressive confusional state culminating in seizures -> chelation therapy -> 36h later: antepartum haemorrhage -> induction of labor -> she gave birth to a 1,6kg baby | yes |
| 77 | N/A | N/A | N/A | / (indirect intake) | indirect intake | / (indirect intake) | the infant was flaccid and areflexic and did not move in response to noxious stimuli, although spontaneous ocular movements were present; as she had emerging alveolar hypoventilation and no gag reflex, she was intubated and ventilated; bilateral diaphragmatic palsy | yes |
| 78 | N/A | N/A | N/A | 9 months | <9 months | to improve her muscle dystrophia | progressive muscle weakness, loss of appetite, constipation, sleep disorders, muscle pain, back pain; abilateral radial paresis was found with a loss of power | no |
| 79 | N/A | N/A | N/A | 18 months | N/A | diabetes (hyperglycemia) | melanosis (diffuse, spotted & leuco); keratosis (palm & sole); a dermatologist described her lesions as blackish discoloration of wide areas of skin over the extremities and trunk with some areas of hypopigmentation | no |
| 80 | N/A | N/A | N/A | 1 year | "some months" | to treat his white skin patches | melanotic arsenical skin lesions (melanosis: diffuse & spotted); after some months, not only did his white patches become brown but the skin of his entire body became darker (diffuse melanosis); after 1 year on the medicine, he had a mild spotted melanosis on his trunk along with the diffuse melanosis but no keratosis on the palms or soles was noted | no |
| 81 | N/A | N/A | N/A | 6 days | 6 days | diabetes (hyperglycemia) | acute gastrointestinal illness, diffuse dermal melanosis with patchy desquamation (melanosis: diffuse & spotted); diarrhea and vomiting; became wery weak and anorexic; several patches of altered skin pigmentation with pruritis; within ~2 weeks, he developed a toxic polyneuropathy resulting in quadriparesis: first he had tingling, numbness, and pain in both feet, which progressed upward to involve the lower legs and thighs as well as the upper extremities -> weakness in his lower extremities that caused difficulty in walking and rising from a squatting position -> his muscle strength continued to diminish in all four extremities -> flaccid quadriparesis | no |
| 82 | N/A | N/A | N/A | for about 12 years intermittently, but recently for 6 months | <6 months | seizure attacks due to neurocysticercosis | exertional dyspnea, dizzines, tingling sensations in her palms & soles; she appeared chronically ill and had anemic conjunctiva and scleral icterus | no |
| 83 | N/A | N/A | N/A | N/A | N/A | rheumatoid arthritis | diffuse abdominal pain, nausea, vomiting | no |
| 84 | N/A | N/A | N/A | 2 months | <2 months | for fertility | nausea, vomiting & lower abdominal pain 2 weeks after a spontaneous abortion | no |
| 85 | 17,6-21,6 g lead in 7,5 months (-> 262,68g of the medicine ingested in 7,5 months -> 19,7 g mercury intake in 7,5 months) | 85.92 | 19,600 | 3 months | <3 months | to increase fertility | back pain & abdominal pain | no |
| 86 | N/A | N/A | N/A | >6 years | <6 years | arthritis | status epilepticus | probably yes |
| 87 | N/A | N/A | N/A | N/A | N/A | patients 5, 6, 7, 9 & 10: arthritis (1), menstrual health (1), diabetes (3) | N/A | N/A |
| 88 | about 2,5 g lead intake in 2 months | 40.98 | 2,500 | N/A | N/A | patients 5, 6, 7, 9 & 10: arthritis (1), menstrual health (1), diabetes (3) | N/A | N/A |
| 89 | N/A | N/A | N/A | N/A | N/A | patients 5, 6, 7, 9 & 10: arthritis (1), menstrual health (1), diabetes (3) | N/A | N/A |
| 90 | 6.468,53 mg lead in 4 weeks; 231,02 mg lead per day | 231.02 | 6,468.53 | N/A | N/A | diabetes | N/A | N/A |
| 91 | N/A | N/A | N/A | N/A | N/A | patients 5, 6, 7, 9 & 10: arthritis (1), menstrual health (1), diabetes (3) | N/A | N/A |
| 92 | 1.037,9 mg lead in 13 weeks | 11.41 | 1,037.90 | N/A | N/A | patients 5, 6, 7, 9 & 10: arthritis (1), menstrual health (1), diabetes (3) | N/A | N/A |
| 93 | N/A | N/A | N/A | N/A | N/A | diabetes | N/A | N/A |
| 94 | N/A | N/A | N/A | N/A | N/A | diabetes | N/A | N/A |
| 95 | N/A | N/A | N/A | several months | N/A | N/A | extensive chronic fatigue; vague abdominal complaints | no |
| 96 | N/A | N/A | N/A | 7,5 months | 2,5 months | rheumatoid arthritis | recurrent nausea, vomiting, constipation, loss of appetite, generalized myalgias, pain in the back, sternal, rib and jaw regions; headache and muscle pain in the upper arms; involuntary weight loss of 9 kg and repeated domestic falls, always after previous periods of vomiting; discrete sclerenicterus | no |
| 97 | N/A | N/A | N/A | 1 month | 1 week | weight loss | severe colicky abdominal pain (epigastric pain), vomiting, 13kg weight loss, pale face | no |
| 98 | N/A | N/A | N/A | 1 month | 3 weeks | weight loss | severe colicky abdominal pain (epigastric pain), pale face, vomiting, 10kg weight loss | no |
| 99 | N/A | N/A | N/A | 2 months | 1 month | multiple sclerosis | bright red rectal blood loss and nausea, vomiting, weight loss, anorexia, abdominal pain, loss of appetite (weight loss of 3kg in a few weeks) and constipation, severe generalized pain; for some time he had "pitting" edema at the ankles, and during the course of treatment it extended above the knees; exertional dyspnea, orthopnea; pallor | no |
| 100 | N/A | N/A | N/A | 6 months | <6 months | hypertension | short-term memory loss, loss of appetite, disinterest in his surroundings, loss of balance, mild disorientation, weakness in the limbs with bilateral hypertonia and hyperreflexia, plantar upgoing & spastic gait | yes |
| 101 | N/A | N/A | N/A | 4 weeks | 2,5 weeks | improve his erections | paroxysmal abdominal pain (colic), constipation, weight loss of 10 kg | no |
| 102 | N/A | N/A | N/A | 6 months | 4,5 months | erectile dysfunction | colicky abdominal pain, constipation, sweating, palpitation, tremor, restlessness, postural symptoms, obstipation, gaseous distension of the abdomen; autonomic dysfunction & intestinal pseudo-obstruction | no |
| 103 | indirect intake | / | / | 13 weeks | 5 weeks | infertility treatment | abdominal pain, nausea, sleeplessness, giddiness and constipation | no |
| 104 | N/A | N/A | N/A | a few months (not further specified) | a few months | N/A | severe abdominal colic, recurrent vomiting, profound weakness, arthralgia, myalgia; lead line | no |
| 105 | N/A | N/A | N/A | N/A | N/A | constipation | constipation | no |
| 106 | N/A | N/A | N/A | 5 years | 4 years | acute myelogenous leukemia (to prolong remission of her AML) | cutaneous manifestations: 1-year history of thick skin on her palms and soles, and dark spots on her body; physical examination revealed numerous punctate atrophic papules within yellow keratotic plaques over the palms and soles that were more prominent on weight-bearing skin; her skin had diffuse, ashy gray, hyperpigmented patches with superimposed hypomelanotic macules on the trunk, most notable on the abdomen and chest, including nipple-areolar complexes; transverse white lines were noted on her fingernails | no |
| 107 | N/A | N/A | N/A | N/A | N/A | N/A | abdominal cramps, constipation and nausea | no |
| 108 | N/A | N/A | N/A | 2 years | 2 years | N/A | abdominal cramps | no |
| 109 | N/A | N/A | N/A | 10 days | <10 days | jaundice | vomiting, severe abdominal pain, fever, extreme weakness of both lower limbs; fever with chills | no |
| 110 | N/A | N/A | N/A | 2 years | 6 months | epilepsy | asymptomatic hypo- and hyperpigmented macules all over the body, with thickening of the palms and soles (generalized hyperpigmentation with multiple, small, hyper- and hypopigmented macules scattered all over the body); acute abdominal discomfort & distension and vomiting; portal hypertension with ascites | no |
| 111 | approximately 650 mg in 18 months; 3,6 mg lead daily | 3.6 | 650 | 7 months | asymptomatic (7 months without symptoms) | to absorb toxins, reduce white patches after milk feedings and preserve the infant's health | asymptomatic | no |
| 112 | 80,64 mg lead in 3 months; 896 μg lead daily for 3 months | 0.90 | 80.64 | 2 months | no symptoms specified (<2 months) | N/A | N/A | N/A |
| 113 | N/A | N/A | N/A | N/A | N/A | cure gastrointestinal problems associated with pregnancy | diffuse, acute abdominal pain; loss of appetite, diarrhea | no |
| 114 | 1.505-1.806 mg lead in 5-6 weeks; 43 mg lead daily | 43 | 1,655.50 | / (indirect intake) | indirect intake | / (indirect intake) | N/A | N/A |
| 115 | N/A | N/A | N/A | 2 months | 2 months | generalized weakness | numbness and tingling sensations in both hands and feet; mild sensory loss (touch, pinprick, vibration sense) of about 50% below both wrists and ankles; fatigue; peripheral neuropathy (it is quite possible that the patient had subclinical peripheral neuropathy as a result of type II diabetes and lead exposure made it manifest) | no |
| 116 | N/A | N/A | N/A | 2-3 months | <1 month | primary infertility due to poor sperm count | weight loss, loss of appetite, non-anginal chest pain, breathlessness, generalized weakness, headache, nausea, vomiting, abdominal colic, severe pallor; feeling of chest compression while sleeping; abdominal colic | no |
| 117 | not more than 2.190 mg lead in total (2 months = 60 days * 5 pills/day * up to 7,3 mg lead/pill = 2.190 mg) | 36.02 | 2,190 | 3 years | <3 years | for muscle building and increasing testosterone level (patient is an amateur bodybuilder) | fatigue for unknown reasons, numbness of the palms & soles, irritability, frequent headache, mental dullness, generalized pain, muscle weakness & tingling, twitching & shaking of the legs while sleeping; he reported having foamy urine | N/A |
| 118 | N/A | N/A | N/A | 6 months | 2 months | psoriasis | upper abdominal colicky pain | no |
| 119 | N/A | N/A | N/A | 1. medicine: 6 months; 2. medicine: for 5 days (3 months ago) | 1. medicine: 3 months; 2. medicine: 5 days, but no intake since 3 months | 1. medicine: as a supplement for her health; 2. medicine: due to low milk supply (childbirth 6 months ago) | generalized muscle pain and weakness, nausea, vomiting, could hardly eat; constant thirst; numbness in the lower extremities, ankles and wrists, accompanied by upper extremity weakness; the upper limb weakness made it difficult to write, brush teeth, and comb hair, and it was accompanied by tingling in the jaw and cheeks; difficulty keeping her head down and lifting it; weight loss of 13 kg in the last 3 months | no |
| 120 | N/A | N/A | N/A | 4 years | <4 years | stress relief, maintenance of health | generalized weakness, vomiting, abdominal pain | no |
| 121 | N/A | N/A | N/A | 1 month | <1 month | psoriasis | abdominal pain, headache, insomnia, generalized weakness, facial pallor, joint pain | no |
| 122 | N/A | N/A | N/A | 1 month | <1 month | weakness in right hand | severe abdominal pain, peripheral neuropathy | no |
| 123 | 1,1–2,2 g of lead over the 4 months; 9-18 mg of lead daily | 13.5 | 1,650 | 18 months | <18 months | hypothyroidism | fatigue | no |
| 124 | 270 mg of lead during the 3 months; approximately 3 mg of lead daily | 3 | 270 | 3 months | <2 months | back pain | epigastric pain and constipation | no |
| 125 | 300 mg of lead over the 6-week period; approximately 7 mg of lead daily | 7 | 300 | 6 months | a few days (<1 week) | dietary supplement | general malaise, anorexia, persistent griping abdominal pain and loss of 15 kg of weight | no |
| 126 | 360-720 mg of lead during the 2 months; approximately 6-12 mg of lead daily | 9 | 540 | 5-6 weeks | 2 weeks | diabetes | progressively worsening post-prandial lower abdominal pain and nausea accompanied by non-bilious and non-bloody vomiting; 5 days later, the patient returned to the emergency department with worsening abdominal pain, nausea and bilious vomiting; pale conjunctivae | no |
| 127 | 1,4-2,9 g of lead during the 4 months; approximately 12-24 mg of lead daily | 18 | 2,150 | N/A | N/A | N/A | N/A | N/A |
| 128 | 300-1.000 mg over the 5 months; approximately 2-7 mg of lead daily | 4.5 | 650 | N/A | N/A | N/A | N/A | N/A |
| 129 | N/A | N/A | N/A | 2 months | ~1 month | to increase his fertility (aphrodisiac) | constipation and abdominal pain | no |
| 130 | 22.680 mg lead in 6 weeks; 540 mg/day | 540.00 | 22,680 | 3 months | 1 month | maintaining health | progressive exertional dyspnea; abdominal pain; intermittent pulling-like pain over the anterior subcostal region; pale conjunctiva | no |
| 131 | N/A | N/A | N/A | 7 days | a few days (<1 week) | rhinitis | severe abdominal pain, vomiting | no |
| 132 | N/A | N/A | N/A | 2 days | 2 days | to treat fever & abdominal pain | abdominal pain, fever, loose stools, vomiting, decreased urine output | no |
| 133 | N/A | N/A | N/A | N/A | N/A | diabetes (type 2) | worsening colicky abdominal pain, associated with nausea and vomiting; irritability, mood swings, and sleep disturbance; erectile dysfunction; mildly reduced power in both legs, with absence of the left knee jerk, while the right one could be elicited only after reinforcement | no |
| 134 | N/A | N/A | N/A | 25 days | 20 days | psoriasis | progressive quadriparesis: bilateral arm weakness and hand paresthesia -> weakness in both legs developed -> he found it difficult to raise either arm, to walk and to climb steps -> moderate proximal dominant symmetric arm and leg weakness; reflexes were brisk in the arms but reduced in the knees and ankles; mild paresthesia in his feet and hands, but no impairment in distal vibration or joint position sense was evident | no |
| 135 | approximate cumulative exposure of 166 mg (56.8 µg/day) | 0.06 | 166 | 3 years; since birth: daily for 1 year; the next 2 years no daily use | 2,5 years | treat and prevent dermatitis (instead of baby powder) -> enhance the health of the skin and to treat the children's skin problems | chronic constipation, abdominal pain | no |
| 136 | 43.398 mg mercury in 5 months; daily intake of 289,32 mg mercury | / | / | 6 months | no symptoms specified (<6 months) | treat and prevent dermatitis (instead of baby powder) -> enhance the health of the skin and to treat the children's skin problems | N/A | N/A |
| 137 | N/A | N/A | N/A | 4 months | asymptomatic (4 months without symptoms) | pregnancy-related nausea & vomiting | asymptomatic | no |
| 138 | N/A | N/A | N/A | 3 months | asymptomatic (3 months without symptoms) | skin problems | asymptomatic | no |
| 139 | N/A | N/A | N/A | 6 weeks | asymptomatic (6 weeks without symptoms) | maintaining health (“keep her pregnancy and fetus healthy") | asymptomatic | no |
| 140 | N/A | N/A | N/A | more than 1 year | a few months | N/A (dietary supplement) | abdominal colic and fatigue | no |
| 141 | N/A | N/A | N/A | 1 month | <1 month | to aid bodybuilding | pain in the epigastrium, episodic vomiting, constipation, easy fatigability, pallor | no |
| 142 | N/A | N/A | N/A | 2 months | asymptomatic (2 months without symptoms) | to promote fertility (she had a history of miscarriages) | asymptomatic | no |
| 143 | N/A | N/A | N/A | 4 months | asymptomatic (4 months without symptoms) | 5 medications to improve fertility & one to improve skin | asymptomatic | no |
| 144 | N/A | N/A | N/A | 5 months | asymptomatic (5 months without symptoms) | increase chance of "having a male baby" | asymptomatic | no |
| 145 | N/A | N/A | N/A | 3 months | <3 months | diabetes | diffuse abdominal pain, decreased oral intake, constipation | no |
| 146 | N/A | N/A | N/A | 6 weeks | 4 weeks | for pain control after removal of a parathyroid adenoma | abdominal pain, nausea, constipation, fatigue, vomiting, unable to eat for 2 days | no |
| 147 | ~916 µg lead per day; ~60 mg lead over the 2 months of treatment | 0.92 | 60 | about 3-4 months | <3-4 months | lower back pain | persistent abdominal pain, emesis, dark stool, latent jaundice, asthenia | no |
| 148 | N/A | N/A | N/A | long time, exact duration unknown | N/A | diabetes, hypertension | acute confusional state, memory disturbances (forgetfulness), abdominal colicky pain, disorientation and irrelevant talking; pallor; patient was confused, disoriented and restless with difficulty in understanding complex commands; plantar response was extensor; gait was ataxic | yes |
| 149 | N/A | N/A | N/A | 2 years | 1 year 10 months | diabetes | loss of appetite; vomiting, severe colicky abdominal pain; pallor; a purple blue line (Burtonian line) was present in the gingiva | no |
| 150 | N/A | N/A | N/A | 1 year | 6 months | psoriasis | many papules appeared on his right leg, which eventually developed into a large tumor in the next few months (verrucous carcinoma) | no |
| 151 | at the maximum recommended dose, the patient ingested around 25.634,7 mg lead in 3 months; at the maximum recommended dose, participants would have ingested around 284.832 μg of lead per day | 284.83 | 25,634.7 (3,5 weeks: 6,978.38) | 2 weeks | 1 week | anal fistula | mild fever, anal pain, dizziness, a pruritic rash, anorexia; perianal gangrene, high fever, gastrointestinal and constitutional symptoms, skin rash, anemia, hair loss, peripheral neuropathy, muscle atrophy, progressive weakness of extremities, leg edema | no |
| 152 | at the maximum recommended dose, the patient ingested around 25.634,7 mg lead in 3 months; at the maximum recommended dose, participants would have ingested around 284.832 μg of lead per day | 284.83 | 25,634.7 (3,5 weeks: 6,978.38) | 3 months | 1 month | chronic leg ulcer (chronic wound over his right lower leg) | anorexia, weight loss, intermitten headache, dizziness, nausea, vomiting, constipation, weakness, anemia | no |
| 153 | at the maximum recommended dose, the patient ingested around 8544,9 mg lead in 1 month; at the maximum recommended dose, participants would have ingested around 284.832 μg of lead per day | 284.83 | 8,544.9 (3,5 weeks: 6,978.38) | 8 years | 7 years 10 months | for wellbeing | lethargy, malaise, myalgia, arthralgia, lead line (blue pigmentation seen along the gum line); his spouse had noted pallor, intermittent memory loss and personality changes as well | N/A |
| 154 | at the maximum recommended dose, the patient ingested around 51.269,4 mg lead in 6 months; at the maximum recommended dose, participants would have ingested around 284.832 μg of lead per day | 284.83 | 51,269.4 (3,5 weeks: 6,978.38) | 5 months | 5 months | to promote natural growth | anuria, intermittent vomitting, resulting in anuric renal failure due to acute interstitial nephritis; anuria of 4 days duration; the anuria was preceded by intermittent vomiting for 2 days | no |
| 155 | at the maximum recommended dose, the patient ingested around 205.077,6 mg lead in 2 years; at the maximum recommended dose, participants would have ingested around 284.832 μg of lead per day | 284.83 | 205,077.6 (3,5 weeks: 6,978.38) | 9,5 months | 1,5 months | to treat partial aphasia, cognitive impairment and right-sided motor weakness resulting from a spontaneous left temporoparietal hemorrhagic stroke experienced 2 years previously | depression, lethargy, fatigue, memory impairment, generalized weakness, severe constipation, anorexia, weight loss of 18kg | N/A |
| 156 | at the maximum recommended dose, the patient ingested around 153.808,2 mg lead in 18 months; at the maximum recommended dose, participants would have ingested around 284.832 μg of lead per day | 284.83 | 153,808.2 (3,5 weeks: 6,978.38) | 18 days | 1 week | atopic dermatitis | diminished appetite, dizziness, abdomen discomfort, itching rash, and skin scaling; he later developed generalized edema, nausea, vomiting, decreased urine amount, diarrhea, vesico-edematous exanthems, malodorous perspiration, fever, shortness of breath, 2-3 episodes of near syncope | no |
| 157 | at the maximum recommended dose, the patient ingested around 34.179,6 mg lead in 4 months; at the maximum recommended dose, participants would have ingested around 284.832 μg of lead per day | 284.83 | 34,179.6 (3,5 weeks: 6,978.38) | a few months (not further specified) | a few months | Bell's palsy (idiopathic facial paresis) | abdominal pain, nausea, vomiting | no |
| 158 | at the maximum recommended dose, the patient ingested around 5.981,43 mg lead in 3 weeks; at the maximum recommended dose, participants would have ingested around 284.832 μg of lead per day | 284.83 | 5,981.43 (3,5 weeks: 6,978.38) | >10 years | 10 years | vitiligo | slowly-enlarging ulcerative nodule on her left sole; in the prior few years, she also had a history of well-defined hyperkeratotic papules and plaques on her palms and soles; squamous cell carcinoma (SCC) from arsenic keratosis | no |
| 159 | N/A | N/A | N/A | 2 weeks | <2 weeks | N/A | severe colicky abdominal pain, constipation, nausea, vomiting | no |
| 160 | N/A | N/A | N/A | N/A | N/A | improves blood circulation in patients recovering from cerebral thrombosis; relief from angina pectoris symptoms | lethargy, anxiety, constipation | no |
| 161 | N/A | N/A | N/A | 6 months | 5,5 months | hypertension | headaches followed by multiple episodes of vomiting & drowsiness, extreme stupor | yes |
| 162 | N/A | N/A | N/A | for many years | N/A | imbalance of her bodily humors toward the vata (air) element causing anxiety, inability to gain weight, anemia, palpitations, and heartburn | fatigue and decreased sensation in her distal extremities; diminished deep tendon reflexes without clonus or spasticity, and normal gait; peripheral neuropathy with sensory greater than motor deficits | no |
| 163 | N/A | N/A | N/A | 15 days | 10 days | weakness (regain strenght) | abdominal pain (pain was episodic, diffuse & had no aggravating on relieving factors), intestinal obstruction (absolute constipation); pallor | no |
| 164 | ~2,52-4,62g lead in 3 weeks | 170 | 3,570 | 6 months | 5 months | enhancement of fertility | progressive shortness of breath, palpitations, decreased exercise tolerance and generalised arthralgia over the previous month | no |
| 165 | N/A | N/A | N/A | 5 years | 4,5 years | eczematous dermatitis | hyperpigmented skin of the palms & soles, diminished sensation & paresthesia of her feet, neuropathy, abdominal pain, loose stools, significant weight loss & 2 episodes of jaundice over the past year without cholestatic symptoms; pallor; There was hyperkeratosis of both palms and soles; rain drop pigmentation over the arms, lower limbs, abdomen, and back; transverse white lines (Mee’slines)on the nails; systemic examination revealed enlarged liver, weakness of both plantar flexors and dorsiflexors of the foot along with graded sensory loss to all sensory modalities below the knee with allodynia; bilateral ankle jerks were absent. | no |
| 166 | approximately 54.750 mg lead in 3 years; daily lead intake approximated 50 mg/day | 50 | 54,750 | 4 months | 2 months | diabetes | weakness of both lower limbs followed by that of upper limbs, bilateral foot drop and wrist drop with graded sensory loss; bilateral severe axonal polyneuropathy with absent sensory nerve action potentials in the median, ulnar, peroneal, and sural nerves | no |
| 167 | N/A | N/A | N/A | 9 years | 9 years | vitiligo | fever, cough, shortness of breath, pallor, pedal edema, raindrop pigmentation of the skin, hyperkeratosis of the palms and soles, coarse crepitations in both lung fields and splenomegaly, bronchiectasis | no |
| 168 | N/A | N/A | N/A | 2 months | a few days | shoulder abcess | asthenia and colicky abdominal pain | no |
| 169 | N/A | N/A | N/A | 3 months | 3 months | fibromyalgia | abdominal pain; Burton's Line | no |
| 170 | 9643,3 mg (9,64 g) in 6 months | 52.84 | 9,643.30 | 1 month | a few days (<1 week) | to treat facial palsy (facial paralysis) | intermittent severe abdominal pain, constipation | no |
| 171 | N/A | N/A | N/A | 5 months | asymptomatic (5 months without symptoms) | global developmental delay (to enhance his muscle strength) | asymptomatic | no |
| 172 | N/A | N/A | N/A | 3 months -> then a new batch was imported from India -> 3-4 weeks after that symptoms occurred | 3-4 weeks | acne & skin problems | tiredness, nausea, abdominal cramps, shortness of breath, severe itching, weight loss | no |
| 173 | N/A | N/A | N/A | 3 months -> then a new batch was imported from India -> 3-4 weeks after that symptoms occurred | 3-4 weeks | acne & skin problems | nausea, tiredness, emotional instability, weight loss | no |
| 174 | total intake in 2 weeks: 5,775 mg barium; 1,995 mg chromium; 0,511 mg lead; 0,329 mg vanadium; 0,175 mg arsenic; 0,091 mg thallium; 0,0581 mg cadmium; 0,091 mg cobalt | 0.04 | 0.51 | 1 month -> then a new batch was imported from India -> 3-4 weeks after that symptoms occurred | 3-4 weeks | acne & skin problems | weight loss | no |
| 175 | N/A | N/A | N/A | 6 months -> then a new batch was imported from India -> 3-4 weeks after that symptoms occurred | 3-4 weeks | acne & skin problems | abdominal cramps, headaches, soreness over entire body, weight loss | no |
| 176 | N/A | N/A | N/A | 2 years -> then a new batch was imported from India -> 3-4 weeks after that symptoms occurred | 3-4 weeks | acne & skin problems | leg pains, loss of appetite, tiredness, difficulty concentrating, irritability, weakness & pain in joints & muscles, clumsiness, weight loss | no |
| 177 | N/A | N/A | N/A | 18 months -> then a new batch was imported from India -> 3-4 weeks after that symptoms occurred | 3-4 weeks | acne & skin problems | abdominal pain, nausea, tiredness, extreme shortness of breath, vomiting, difficulty concentrating, lethargy, drowsiness, extreme shortness of impaired memory, irritability, weakness & pain in joints & muscles, severe itching, clumsiness, weight loss | no |
| 178 | N/A | N/A | N/A | 4 months -> then a new batch was imported from India -> 3-4 weeks after that symptoms occurred | 3-4 weeks | acne & skin problems | abdominal pain, tiredness, headaches, difficulty concentrating, weight loss | no |
| 179 | N/A | N/A | N/A | 3 weeks -> then a new batch was imported from India -> 3-4 weeks after that symptoms occurred | 3-4 weeks | acne & skin problems | backache that worsened over time, severe nausea, abdominal cramps, loss of appetite, weakness, confined to bed, weight loss | no |
| 180 | approximately 5.743,5 mg arsenic in 18 months; 10,5 mg of arsenic daily | / | / | 18 months | 12-15 months | psoriasis | he presented with two progressive, ulcerative palmoplantar lesions; developed widespread, numerous and concurrent cutaneous squamous cell carcinomas (sCCs) in non-sun exposed skin areas after taking a traditional medicine; acute onset of nausea, and occasional vomiting, anorexia, fatigue, alopecia and noted the emergence of white horizontal lines on his fingernails; waxing and waning mouth ulcers, alopecia (hair loss) worsened; small foci of non-healing ulceration began to develop at the right distal palm proximal to the fifth finger and at the right calcaneal sole -> these lesions progressed and during this time he also noted, either by palpation or by sight, the emergence of nodular lesions occurring at his perianal, right axilla and right inguinal skin areas | no |
| 181 | 366 µg lead in 2 months; 6,1 µg/day | 0.01 | 0.37 | 3 months | <2 months | N/A | progressive fatigue, shortness of breath during a period of multiple weeks | no |
| 182 | N/A | N/A | N/A | 9 months | 3 months | to ameliorate his impaired glucose tolerance (family history of diabetes mellitus) | acute onset of confusion & word-finding difficulty; nausea, epigastric discomfort, constipation, anorexia, weight loss of about 6 kg; transient encephalopathy, lethargy, jaundice | yes |
| 183 | N/A | N/A | N/A | 4 weeks | ~26 days | to reduce the side effects of chemotherapy (she started taking the medication after surgery) | 5 days after receiving the first cycle of chemotherapy she presented with altered sensorium of a few hours duration preceded by severe vomiting for 2 days; she was conscious, dehydrated, disoriented in time, place and person and responsive to painful stimulus -> case of early breast cancer with non‐oliguric acute renal failure | no |
| 184 | N/A | N/A | N/A | 2 months | <2 months | chronic eczema | arsenical keratosis; rash with pruritus for more than one year, followed by exacerbation with chromatosis for two months; rashes appeared and chromatosis with diffuse verrucous hyperplasia as big as granule or mung bean and Mees’ lines appeared on finger nails | no |
| 185 | N/A | N/A | N/A | 3 months | 2,5 months | low back pain | progressive epigastric pain without radiation; dark coloured stools, decreased appetite, weight loss and 1 day of nausea associated with an episode of blood-streaked emesis, decreased appetite | no |
| 186 | N/A | N/A | N/A | 3 weeks | 3 weeks intake, first symptoms 6 weeks after last intake | epilepsy (seizures) | abdominal pain (colic), constipation, irritability (irritation), skin turned pale (pallor), weight loss; he had not defecated during the previous week | no |
| 187 | N/A | N/A | N/A | 2 months | 7 weeks | diabetes (type 1) | abdominal pain, non-bilious vomiting, constipation; pallor | no |
| 188 | N/A | N/A | N/A | 1 year | 9 months | diabetes (type 2) | colicky abdominal pain, decreased appetite and generalised weakness, pallor, bluish pigmentation of the gum-tooth line (Burtonian line) | no |
| 189 | N/A | N/A | N/A | 5 years | 4,5 years | N/A | worsening fatigue, weakness & numbness in his hands and feet; hyperkeratosis on the patient’s soles & palms, scattered actinic keratosis-appearing lesions over the dorsi of the hands and plantar surfaces, freckling of the trunk; impaired 2-point discrimination and reduced sensory perception to monofilaments in a glove-and-stocking pattern | no |
| 190 | N/A | N/A | N/A | 4 days | 2 days | psoriasis | on the second day, he started to experience diarrhea, vomiting, pain and swelling of the affected skin; in the following 2 days, he experienced yellow-water diarrhea, severe vomiting and skin eruptions; on the 4th day, his condition became to general weakness and he was referred to the emergency department due to progressive nausea and 3 days of diarrhea; multiple patches of the skin throughout the body showed large dark purple spots, scaling, partial ulceration and exudation; erythematous, slightly scaling, indistinct borders are usually seen; at post-mortem examination, his skin patches that almost ranged at complete body coverage (except the face, hands and feet) | no |
| 191 | N/A | N/A | N/A | 2-3 months | <3 months | N/A (patient has an end-stage kidney disease and is on chronic hemodialysis) | progressive global motor weakness, tremor and hallucinations; polyarthralgia, constipation, abdominal pain, and nausea and vomiting; severe neuropathic pain in all limbs, nightmares and involuntary vocalization; alopecia was notable; moderate-to-severe muscle weakness involving the face, neck and all limbs; absent reflexes at C6, C7 and S1; impaired proprioception in the hands and feet; and a patchy disturbance of light touch and pinprick over all limbs | no |
| 192 | N/A | N/A | N/A | 6 months | 3 months | N/A (the worse the patient got, the harder he took the herbal medicine) | severe abdominal pain and 11 kg weight loss | no |
| 193 | N/A | N/A | N/A | 6 months | <6 months | improves blood circulation in patients recovering from cerebral thrombosis; relief from angina pectoris symptoms | itching, chest discomfort | no |
| 194 | N/A | N/A | N/A | 3 weeks | a few days (<1 week) | to reduce stress | severe peripheral poly neuropathy and sensory motor symptoms with neuropathic pain; headache, fatigue | no |
| 195 | N/A | N/A | N/A | 3 years | 1,5 years (18 months) | Jineijin: to alleviate nausea, vomiting, etc. | severe colic periumbilical pain and constipation (unbearable pain that came on and off every 2-3 months) | no |
| 196 | N/A | N/A | N/A | 15 days | <15 days | menstrual problems | recurrent abdominal pain, severe tingling, burning sensation followed by weakness in both lower limbs, severe hair loss -> she virtually became total alopecic; on examination, she was conscious, disoriented, febrile (100 F), and dehydrated; neurological examination revealed generalized wasting and hypotonia in all the four limbs with predominately distal weakness; deep tendon reflexes were depressed and planter responses were extensor | no |
| 197 | N/A | N/A | N/A | 1 month | <1 month | difficulty urinating | abdominal pain, poor appetite, nausea, abdominal distention, cold sweating, general weakness | no |
| 198 | N/A | N/A | N/A | 8 months | <8 months | diabetes | pain in the lower abdomen, decreased appetite, general body ache, tiredness, constipation, nausea, vomiting, pallor | no |
| 199 | N/A | N/A | N/A | 6 months | 4,5 months | diabetes type 2 control | progressive cramping abdominal pain, malaise, nausea and vomiting, Burton line of the gums; conjunctival pallor | no |
| 200 | N/A | N/A | N/A | 8 months | 7 months | atherosclerosis prevention | periumbilical pain, constipation | no |
| 201 | N/A | N/A | N/A | 8 months | asymptomatic (8 months without symptoms) | atherosclerosis prevention | asymptomatic | no |
| 202 | N/A | N/A | N/A | 9 months | asymptomatic (9 months without symptoms) | to prevent and treat dermatitis | no symptoms noticed | no |
| 203 | 8.788,26 mg lead in 1 year; pills: 168.420 µg/week; powder: 585 µg/week | 24.14 | 8,788.26 | for 2 weeks | < 2 weeks | severe dyspepsia & loss of appetite (appetite enhancement) | nausea, loss of appetite and general well-being followed by yellowish discolouration of skin and darkening of urine associated with severe itching all over the body, especially worse at night time in the absence of clay-coloured stools, abdominal pain and fever | no |
| 204 | N/A | N/A | N/A | N/A | asymptomatic | improves blood circulation in patients recovering from cerebral thrombosis; relief from angina pectoris symptoms | no symptoms | no |
| 205 | N/A | N/A | N/A | N/A ("for a long time", not further specified) | N/A | N/A | poor appetite and vomiting; after admission, he developed a convulsion, characterized with loss of consciousness, eyes staring, froth at the mouth and limb stiffness | yes |
| 206 | N/A | N/A | N/A | N/A ("for a long time", not further specified) | N/A | N/A | N/A | N/A |
| 207 | N/A | N/A | N/A | about 1 year | 11 months | facial acne | severe colic periumbilical pain, constipation | no |
| 208 | N/A | N/A | N/A | N/A | N/A | autism | N/A (probably asymptomatic) | N/A |
| 209 | N/A | N/A | N/A | 25 days | 10 days | infertility | abdominal pain and vomiting, constipation; abdominal pain was associated with multiple episodes of non-bilious, nonbloodstained vomiting | no |
| 210 | Daily arsenic dose of greater than 650 mg/day and a mercury dose of greater than 300 mg/day.; exactly: daily arsenic dose: ~672,76 mg; mercury: 442, 15 mg; lead: 15,53 mg | 15.53 | N/A (5,668.45/year) | N/A ("for a long time", not further specified) | N/A | N/A | N/A | N/A |

*Tab. S2C: Raw data table of all patient cases – Metal intake amount, Daily lead intake amount (mg), Total lead intake amount (mg), Intake duration, Latency period, Reason(s) for taking the medicine, Clinical symptoms, Lead encephalopathy*

| **Case num-ber** | **BLL (µmol/l), highest measured level** | **BLL normal range specified in the article** | **Other metal concentrations in blood/urine (except BLL)** | **Anemia yes/no** | **Kind of anemia** | **Baso-philic stipp-ling** | **Hb-level (mmol/l)** | **Hema-tokrit** | **Liver** |
| --- | --- | --- | --- | --- | --- | --- | --- | --- | --- |
| 1 | 6 | <2 µmol/l | urinary excretion of lead: 2,27 µmol/24h (472 µg/24h; normal: <0,4 µmol/24h, <80 µg/24h); chelation therapy: urinary lead excretion rose to 10,8 µmol (2240 µg)/24 h | yes | red cell anisocytosis, polychromasia, and 10 nucleated red cells per 100 white cells | yes | 6.39 | N/A | plasma bilirubin 24 µmol/l (1,44 mg/100 ml) (normal 2-17 µmol/l (0,1-1,0 mg/100 ml)) |
| 2 | 6.62 | <40 µg/dl; 24h-urine lead level: normal <50 µg lead/24h | 24h urine lead level was 52 µg | yes | hypochromia, microcytosis & anisocytosis of the red cells, with marked polychromasia | yes | 4.9 | N/A | liver function tests were normal |
| 3 | 5.99 | N/A | N/A | N/A | N/A | N/A | N/A | N/A | N/A |
| 4 | N/A | " | N/A | N/A | N/A | N/A | N/A | N/A | N/A |
| 5 | 2.9 | N/A | N/A | N/A | N/A | N/A | N/A | 38% | N/A |
| 6 | 2.17 | N/A | / | N/A | N/A | N/A | N/A | N/A | N/A |
| 7 | 1.31 | " | N/A | yes | N/A | no | 8.25 | 38,9% | N/A |
| 8 | 0.64 | " | N/A | no | / | no | 9.49 | 44,4% | N/A |
| 9 | 0.3 | " | N/A | no | / | no | 9.37 | 44,6% | N/A |
| 10 | N/A | " | N/A | yes | N/A | yes | 4.34 | N/A | N/A |
| 11 | 6.62 | " | / | N/A | N/A | N/A | N/A | N/A | N/A |
| 12 | N/A | N/A | / | N/A | N/A | N/A | N/A | N/A | N/A |
| 13 | 7.29 | <40 µg/dl | / | yes | N/A | yes | 6.39 | N/A | N/A |
| 14 | 7.92 | " | N/A | yes | N/A | yes | 4.96 | N/A | N/A |
| 15 | 6.76 | 0-1.9 µmol/l | N/A | yes | hypochromia and microcytosis | yes | 5.15 | N/A | N/A |
| 16 | 6.85 | " | N/A | yes | N/A | no | 5.09 | N/A | N/A |
| 17 | 7.4 | " | N/A | N/A | N/A | no | N/A | N/A | N/A |
| 18 | 2.25 | " | N/A | no | / | no | normal | normal | N/A (no abnormalities) |
| 19 | 3.12 | " | N/A | yes | N/A | no | 5.96 | N/A | N/A |
| 20 | 4.03 | " | N/A | yes | N/A | no | 5.4 | N/A | N/A |
| 21 | 9.42 | N/A | / | yes | mild hypochromia of the red cells | N/A | 3.91 | N/A | albumin 40,5 g/l, globulin 15,5 g/l |
| 22 | 3.38 | BLL normal <250 µg/l; blood arsenic level: normal <40 µg/l | / | yes | normochromic, normocytic picture | yes | 5.34 | N/A | total bilirubin 22 µmol/l (normal range 0-17 µmol/l); gamma GT 45 U/l (normal range 7-41 U/l) |
| 23 | 3.43 | N/A | N/A | yes | hypochromic | yes | under normal range | 28,8 | N/A |
| 24 | 3.86 | N/A | urine lead: 2820 µg/dl (1,37 µmol/l) | yes | sideroblastic anemia | N/A | 5.03 | 24% | N/A |
| 25 | 4.64 | N/A | N/A | yes | N/A | yes | 7.38 | N/A | N/A |
| 26 | 4.25 | <15 µg/dL | N/A | N/A | N/A | N/A | N/A | N/A | N/A |
| 27 | 4.55 | < 1.45 µmol/L | 24h urine specimen: lead excretion was 2,3 µmol/24h (normal <0,37 µmol/24h), arsenic excretion was 0,1 µmol/24h (normal <1,6 µmol/24h), mercury excretion was 0,03 µmol/24h (normal <0,2 µmol/24h); urinary lead excretion rose to 81.9 µmol in the first 24 hours after therapy, falling to 19.8 µmol/24h by day 5 | yes | normocytic anaemia (normocytic red cells with polychromasia); Screening tests for haemolysis and abnormal haemoglobinopathies gave negative results | yes | 6.14 | N/A | low-grade hepatitis; Liver function tests showed apredominant hepatitic picture, with alkaline phosphatase 150 u/L (normal range, 30-120 u/L), alanine aminotransferase 270 u/L « 40 u/L), aspartate aminotransferase 200 u/L « 30 u/L) and bilirubin 45 flmol/L « 20 flmoI/L) |
| 28 | / | / | urinary concentration of inorganic arsenic: 63 µg/l (normal: <2 µg/l, toxic concentration >2 µg/l); hair arsenic concentration: 20 µg/g (normal: <1 µg/g) | N/A | N/A | N/A | N/A | N/A | N/A |
| 29 | / | / | urinary mercury concentration: 105 µg/l (normal: <10 µg/l, toxic concentration >100 µg/l) 12 weeks after the last exposure to the ethnic remedies | N/A | N/A | N/A | N/A | N/A | N/A |
| 30 | 4.01 | <10 µg/dL | N/A | N/A | N/A | N/A | N/A | N/A | N/A |
| 31 | 5.3 | <0.7 µmol/l | N/A | yes | normocytic | yes | 6.21 | N/A | hepatitis; Liver function tests: alanine aminotransferase (ALT) 222 IU/l (2-29), aspartate aminotransferase (AST) 75 IU/I (15-37), bilirubin 45 mmol/l (2-17) |
| 32 | 3.7 | N/A | N/A | yes | N/A | yes | under normal range | 0.26 | Total bilirubin level peaked June 16 at 79 μ/L (4.6 mg/dL) and was mostly indirect bilirubin (direct bilirubin, 12 μ /L [0.7 mg/dL]); June 1st: alanine aminotransferase, 703 U/L; aspartate aminotransferase, 459 U/L; total bilirubin, 44 μ /L (2.6 mg/dL); and alkaline phosphatase, 233 U/L; June 8th: liver enzyme levels were slightly elevated but lower than previously; lactate dehydrogenase, 813 U/L |
| 33 | / | N/A | blood: As 15 µg/l; urine: Hg 87 µg/l | N/A | N/A | N/A | N/A | N/A | N/A |
| 34 | 1.16 | " | blood: As 13 µg/l, Hg 0 µg/l; urine: As 61 µg/l | N/A | N/A | N/A | N/A | N/A | N/A |
| 35 | / | " | blood: As 9 µg/l, Hg 24 µg/l; Urine: As 180 µg/l, Hg 340 µg/l | N/A | N/A | N/A | N/A | N/A | N/A |
| 36 | 2.17 | " | blood: As 53 µg/l; urine: As 400 µg/l, Hg 5 µg/l, Pb 1.460 µg/l, Sb 2 µg/l | yes | haemolytic anaemia | N/A | under normal range | N/A | N/A |
| 37 | 3.96 | " | urine: Pb 600 µg/l | yes | N/A | N/A | under normal range | N/A | N/A |
| 38 | 4.54 | <20 µg/dl | N/A | yes | N/A | yes | 6.76 | N/A | N/A |
| 39 | 7.25 | " | N/A | yes | N/A | yes | 5.9 | N/A | N/A |
| 40 | 8.55 | " | blood mercury concentration 5 nmol/L (<30), but urine mercury was elevated at 16,7 nmol/mmol creatinine (<5,5) | yes | N/A | yes | under normal range | N/A | N/A |
| 41 | 3.38 | " | N/A | yes | N/A | yes | 4.96 | N/A | N/A |
| 42 | 3.28 | " | blood arsenic concentration was <20 µg/l | yes | N/A | yes | 7.2 | N/A | N/A |
| 43 | 2.22 | lead-exposed workers: 40 µg/dl for males and 30 µg/dl for females | N/A | N/A | N/A | N/A | N/A | N/A | N/A |
| 44 | 6.28 | " | N/A | yes | N/A | yes | 4.41 | N/A | mildly abnormal liver function (SGOT: 36 U/l, SGPT: 24 U/l) |
| 45 | 3.57 | " | N/A | N/A | N/A | N/A | N/A | N/A | N/A |
| 46 | 1.1-4.45 | N/A | N/A | yes | N/A | yes | 4.96 | N/A | N/A |
| 47 | 1.1-4.45 | " | N/A | no | N/A | yes | 8.69 | N/A | N/A |
| 48 | 1.1-4.45 | " | N/A | no | N/A | no | 8.44 | N/A | N/A |
| 49 | 1.1-4.45 | " | N/A | yes | N/A | yes | 6.64 | N/A | N/A |
| 50 | 4.4 | N/A | N/A | yes | N/A | N/A | 5.96 | N/A | At his first presentation 2 months earlier, laboratory examinations found elevated serum liver enzymes: alanine transaminase (ALT) 145 U/L and aspartate aminotransferase (AST) 78 U/L |
| 51 | 1.1-4.45 | " | N/A | yes | N/A | no | 6.33 | N/A | N/A |
| 52 | N/A | " | N/A | N/A | N/A | N/A | N/A | N/A | N/A |
| 53 | 4.4 | " | N/A | N/A | N/A | N/A | N/A | N/A | elevated serum liver enzymes; AST 119 U/L (reference range 838 U/L), ALT 390 U/L (reference range 0-75 U/L). |
| 54 | 3.38 | N/A | N/A | N/A | N/A | N/A | N/A | N/A | N/A |
| 55 | 4.38 | 10-60 µg/dl | 24h urinary lead level (on the day before she was transferred to Stanford Hospital): 1044 µg/24h (normal: 0-80 µg/24h); 24h excretion of lead (measured in Stanford Hospital): 281 µg/day (normal: 0-80 µg/day) | yes | N/A | yes | N/A | 26.60% | N/A |
| 56 | 1.54 | " | N/A | no | / | no | 9.12 | 42,6% | N/A |
| 57 | 2.66 | <2 µg/dl | N/A | yes | slight microcytosis and hypochromasia with no hemolysis | yes | 4.16 | N/A | liver enzymes were normal |
| 58 | 5.3 | <20 mg/dl [0.97 mmol per liter] | urinary excretion of lead: 651 µg/24h (3,14 µmol/24h; normal: <80 µg/day); no urinary excretion of thallium, arsenic or mercury was found | yes | hypochromia, slight microcytosis and anisocytosis | yes | 5.52 | N/A | hepatocellular abnormalities (see article); total bilirubin, 1.9 mg per deciliter (32.5 mmol per liter); conjugated bilirubin, 0.5 mg per dec- iliter (8.6 mmol per liter); alkaline phosphatase, 83 U per liter; g-glutamyltransferase, 85 U per li- ter; aspartate aminotransferase, 46 U per liter; alanine aminotransferase, 97 U per liter; albu- min, 4.4 g per liter; indirect hyperbilirubinemia |
| 59 | 2.8 | <100 µg/l; normal urinary lead: <15 µg/l; urinary ARSENIC: normal <20 µg/l | urinary lead concentration: 385 µg/l (normal: <15 µg/l); slightly increased urinary concentrations of arsenic 24,1 μg/l (normal <20 μg/l) and silver 1,3 μg/l (normal <0,5 µg/l); the urinary concentrations of mercury, chromium, manganese, antimony, and cadmium were within normal limits | yes | normochromic anaemia; mild haemolysis -> Reduced haptoglobin of 78.9 mg/dl (normal 123–320 mg/dl) indicated a mild haemolysis | yes | 4.84 | N/A | increase of AST, ALT & y-GT; Laboratory tests showed an increase of aspartate aminotransferase (AST) to 44 U/l (normal 1–18 U/l), of alanine aminotransferase (ALT) to 107 U/l (normal 1–22 U/l), and of „y-glutamyl transpeptidase (y-GT) to 34 U/l (normal 6–28 U/l); Lactate dehydrogenase and bilirubin were not increased |
| 60 | 1.93 | N/A | N/A | N/A | N/A | N/A | N/A | N/A | N/A |
| 61 | 6.76 | N/A | N/A | yes | hypochromic, normocytic anemia without signs of iron deficiency or hemolysis | yes | 5.2 | N/A | N/A |
| 62 | 0.31 | <1.5 µmol/l | blood mercury concentration: 83 nmol/l (normal for adults <50 nmol/l); the blood concentrations for manganese was 246 nmol/l (normal 70–280 nmol/l); urine arsenic was 10 nmol/mmol creatinine (normal <68 nmol/mmol) | no | / | N/A | normal | N/A | liver enzymes were normal |
| 63 | 4.15 | N/A | N/A | yes | N/A | no | 5.71 | N/A | N/A |
| 64 | / | / | N/A | N/A | N/A | N/A | N/A | N/A | N/A |
| 65 | / | / | urinary arsenic level was normal (15 μg/g creatinine; reference value <40 μg/g creatinine); the hair arsenic level was 6,6 mg/kg (normal <1 mg/kg) | yes | severe normochromic anemia | N/A | 5.46 | N/A | N/A |
| 66 | 3.7 | <0.5 µmol/l | N/A | no | / | N/A | N/A | N/A | elevated serum bilirubin concentrations & alanine transaminase activity, but normal alkaline phosphatase activity, albumin concentrations and prothrombin time; bilirubin: 46 µmol/l (reference range: 2-17); alanine transaminase: 129 IU/l (5-40) |
| 67 | 3.03 | <1.21 μmol/L | N/A | yes | normochromic & normocytic anemia; polychromasia, anisocytosis | yes | 4.47 | N/A | liver dearangement, elevated serum alkaline phosphatase level & serum alanine aminotransferase level; serum alkaline phosphatase level was 138 U/L (normal range, 50-120 U/L) and the serum alanine aminotransferase level was 181 U/L (normal range, 10-40 U/L) |
| 68 | 5.6 | " | N/A | yes | normochromic and normocytic indices | no | 7.32 | N/A | liver function tests were normal. |
| 69 | 11.59 | N/A | N/A | yes | normochromic, normocytic anemia | yes | under normal range | N/A | N/A |
| 70 | / | N/A | N/A | yes | N/A | N/A | 6.08 | N/A | liver histopathology was suggestive of severe alcoholic hepatitis in whom the liver histopathology was suggestive of severe AH with additional findings of dense fibrosis localized mostly to the portal regions; predominantly direct hyperbilirubinemia, aspartate aminotransferase (AST) to alanine aminotransferase (ALT) ratio > 2, hypoalbuminemia and hyponatremia with raised prothrombin time; AST188 IU/l; ALT 54 IU/l, Alkaline phosphatase 214 IU/l; Gamma-glutamyl transpeptidase 164 IU/l; total bilirubin 12.8 mg/dl; direct bilirubin 8.8 mg/dl; -> Ayurvedic treatment induced severe alcoholic hepatitis and non-cirrhotic portal hypertension in a 14-year-old girl -> ggf. Artikel nochmal lesen, wenn genauere Infos benötigt |
| 71 | / | 0-9 µg/dl | Her blood analysis showed elevated arsenic levels; 12 times the acceptable upper limit, with normal lead, cadmium, mercury, and chromium levels; blood level of arsenic: 145 µg/l (biological reference interval: 0,4-11,9 µg/l), | N/A | N/A | N/A | N/A | N/A | N/A |
| 72 | 6.7 | " | N/A | yes | normochromic & normocytic anemia | N/A | 6.08 | N/A | N/A |
| 73 | 7.39 | N/A | 24h ULL: 4785 ug/24h | yes | hemolytic anemia (normocytic normochromic) | yes | 5.19 | N/A | hepatitis; total bilirubin 68mmol/L, direct bilirubin 18mmol/L, alanine transaminase (ALT) 103U/L, aspartate transaminase (AST) U/ L72,gamma glutamyl transferase (GGT) 109U/L, alkaline phosphatase 182U/L; lactic dehydrogenase (LDH) 561U/mL; albumin 39gm/dl; haptoglobin <6mg/dl(27-139mg) |
| 74 | 4.25 | <10 µg/dl | N/A | N/A | N/A | N/A | N/A | N/A | N/A |
| 75 | 3.33 | " | N/A | N/A | N/A | N/A | N/A | N/A | N/A |
| 76 | 5.2 | ≤0.48 µmol/L | N/A | yes | N/A | yes | 4.34 | N/A | N/A |
| 77 | 11.8 | " | the high urinary lead concentration of 52 µmol/L on Day 3 of chelation therapy was an indicator that lead was being excreted | N/A | N/A | no | N/A | N/A | N/A |
| 78 | 4.25 | <90μg/l | N/A | yes | hypochromic, microcytic anaemia; Hematological parameters showed anemia | yes | 4.9 | N/A | N/A |
| 79 | / | / | hair and nails contained elevated levels of arsenic, urine arsenic concentration normal; arsenic concentration in hair: 2.600 µg/kg (normal: 80-250 µg/kg; 1000 µg/kg is an indication of toxicity); nails: 10.200 µg/kg (normal: 430-1080 µg/kg); skin scale: 2.700 µg/kg (normal value for skin arsenic is not defined in literature); urine: 29 µg/l (normal: 5-40 µg/1,5l per day) | no | / | no | 8.13 | 37 g/dl | N/A |
| 80 | / | " | arsenic concentration: urine 277 µg/l (normal: 5-40 µg/1,5l per day), hair 5.900 µg/kg (normal: 80-250 µg/kg), nail 6.900 µg/kg (normal: 430-1.080 µg/kg) | no | / | no | normal | normal (N/A) | N/A |
| 81 | / | " | arsenic concentration: urine 23 µg/l (normal: 5-40 µg/1,5l per day), hair 7.500 µg/kg (normal: 80-250 µg/kg), nail 11.500 µg/kg (normal: 430-1.080 µg/kg); skin scale: 20.400 µg/kg (normal value for skin arsenic is not defined in literature); blood concentrations of arsenic, lead, mercury, and thallium were normal but hair and nail samples were positive for arsenic | yes | N/A | no | 4.59 | N/A | N/A |
| 82 | / | / | increased urinary excretion of arsenic of 67,2 µg/day (normal range: 0-25 µg/day); other heavy metals included: serum cadmium 3.1 g/dL (0-10 g/dL), serum lead 5 g/dL (0-60 g/dL), and urine lead 6 g/L (0150 g/L). | yes | hemolytic anemia; polychromato-philia, anisocytosis & macrocytosis; peripheral pancytopenia | N/A | 3.54 | N/A | total serum protein 6.6 g/dL, albumin 4.1 g/dL, alkaline phosphatase 62 IU/L, AST 132 IU/L, ALT 29 IU/L, total bilirubin 1.6 mg/dL (direct, 0.3 mg/dL); lactate dehydrogenase (LDH) 4,989 IU/L; serum haptoglobin was 7.25 mg/dL (normal range: 30 to 200 mg/dL) |
| 83 | 3.91 | N/A | N/A | yes | microcytic anemia | yes | under normal range | N/A | N/A |
| 84 | 5.41 | " | N/A | yes | severe, persistent microcytic anemia | yes | under normal range | N/A | N/A |
| 85 | 3.86 | " | N/A | N/A | N/A | N/A | N/A | N/A | N/A |
| 86 | 4.3 | " | N/A | yes | N/A | yes | under normal range | N/A | N/A |
| 87 | 2.9 | " | N/A | N/A | N/A | N/A | N/A | N/A | N/A |
| 88 | 2.22 | " | N/A | N/A | N/A | N/A | N/A | N/A | N/A |
| 89 | 4.4 | " | N/A | N/A | N/A | N/A | N/A | N/A | N/A |
| 90 | 2.37 | " | N/A | N/A | N/A | N/A | N/A | N/A | N/A |
| 91 | 3.48 | " | N/A | N/A | N/A | N/A | N/A | N/A | N/A |
| 92 | 4.4 | " | N/A | N/A | N/A | N/A | N/A | N/A | N/A |
| 93 | 4.83 | " | N/A | N/A | N/A | N/A | N/A | N/A | N/A |
| 94 | 5.7 | " | N/A | N/A | N/A | N/A | N/A | N/A | N/A |
| 95 | 3.67 | N/A | N/A | yes | microcytic anemia | yes | 4.59 | N/A | N/A |
| 96 | 4.12 | < 100 μg/l | urinary lead concentraton: 200 µg/l (normal: <18 µg/l), 1.443 µg/l after a single dose of 300 DMPS; normal mercury blood level | yes | normochromic anaemia; hemolysis signs, marked polychromasia, anisocytosis, poikilocytosis | yes | 4.41 | 25,4% | Haptoglobin was decreased at 19 mg/dl |
| 97 | 3.52 | <10 g/dL | N/A | yes | normocytic normochromic anemia | yes | 4.34 | 21,3% | GOT: 60 IU/L; GPT: 39 IU/L (highest measured GPT: 60 IU/L) |
| 98 | 3.53 | " | N/A | yes | normocytic normochromic anemia | yes | 3.85 | 18,6% | GOT: 60 IU/L; GPT: 52 IU/L |
| 99 | 4.69 | N/A | N/A | yes | normocytic anaemia without haemolysis and with normal iron load; anisocytosis | yes | 5.2 | N/A | Serum activity of lactate dehydrogenase was slightly elevated: 437 U/l (150-400); the concentration of haptoglobin was 0.95 g/l (0.16-2.00). |
| 100 | 7.78 | < 10 µg/dL | N/A | N/A | N/A | N/A | N/A | N/A | N/A |
| 101 | 2.34 | < 100 µg/L | N/A | yes | N/A | N/A | 7.94 | N/A | mild elevation of liver enzyme levels; transaminase and gamma-glutamyltranspeptidase activities were discretely increased to 2 times normal |
| 102 | 4.77 | <14.00 μg/dL | N/A | yes | N/A | yes | under normal range | N/A | N/A |
| 103 | 3.84 | ≤0.48 µmol/l | N/A | yes | microcytic hypochromic blood picture; an abnormal haem compound found elevated in iron deficiency anemia | no | 4.96 | N/A | increased alanine aminotransferase: 80 U/l (normal range: ≤65 U/l) |
| 104 | 3.14 | ≤ 30 μg/ dl | N/A | yes | normocytic, normochromic anemia | N/A | 4.28 | N/A | increased bilirubin (3.1 mg/dl, mostly indirect) and mildly elevated alanine aminotransferase |
| 105 | 4.02 | <10 μg/dL; different source: <5 µg/dl | N/A | yes | normocytic normochromic blood picture | yes | 6.33 | N/A | N/A |
| 106 | / | blood ARSENIC level: normal 0.0-62.0 µg/L | elevated urine arsenic level | no | / | no | normal | normal (N/A) | liver transaminase levels were within reference range |
| 107 | 3.86 | <25 µg/dl | during therapy urinary excretion of lead was 376 μg/24h (normal 4-50 μg/24h) and the lead/creatinine ratio was 783 μg/g creat. (normal ≤50 μg/g creat.) | yes | normochromic normocytic anaemia | yes | 6.21 | N/A | N/A |
| 108 | 3.62 | " | N/A | yes | normochromic normocytic anaemia | yes | 5.03 | N/A | N/A |
| 109 | 2.77 | < 9 µg/dl | N/A | yes | mycrocytic hypochromic anemia (hemolytic anemia) | N/A | 5.46 | N/A | Liver enzymes and the renal parameters were within normal limits indicating that the liver and kidneys were not affected at this blood lead level.; mild elevation of total serum bilirubin -1.6 mg/dl (normal -0.2 to 1.0 mg/dl) and unconjugated bilirubin -1.3mg/dl (normal -0.2 to 0.6 mg/dl) |
| 110 | / | normal serum ARSENIC level: <60 μg/ L | serum arsenic level: 202,20 μg/L | no | / | N/A | normal | N/A | liver function tests were wthin normal limits; Ultrasonography & CT scan of the abdomen revealed gross ascites, a nodular liver surface, and an atrophied right lobe of the liver, with compensatory hypertrophy of the left and caudate lobe; liver biopsy showed spotty necrosis and focal perisinusoidal fibrosis, suggestive of noncirrhotic portal fibrosis |
| 111 | 2.95 | 10 µg/dL | N/A | yes | N/A | N/A | 6.58 | N/A | N/A |
| 112 | 1.89 | 0–0.48 μmol/l | body lead burden in the 72h urine sample collected following the chelat infusion was 5,07 μmol (1.050 μg) (acceptable range <2,9 μmol [600 µg]) | N/A | N/A | N/A | N/A | N/A | N/A |
| 113 | 4.93 | < 10 µg/dL | N/A | yes | microcytic anemia | N/A | 5.77 | N/A | aspartate transaminase and alanine transaminase levels were elevated to 74 and 54 units/L, respectively, with normal bilirubin; during 9 days of hospitalization, the patient’s aspartate transaminase and alanine transaminase levels increased to as high as 178 and 144 units/L |
| 114 | 2.89 | " | N/A | N/A | N/A | N/A | N/A | N/A | N/A |
| 115 | 3.57 | <5-10 µg/dl; urinary lead level: normal <50 µg/dl |  | yes | normocytic normochromic | no | 8.87 | N/A | SGOT 23 IU, serum glutamic pyruvic transaminase (GPT) 53 IU; lactate dehydrogenase (LDH) 0.88 IU; serum proteins 6.9 g/dL with albumin 4.3 g/dl |
| 116 | 4.35 | <40 µg/dl | N/A | yes | microcytic hypochromic anaemia with no evidence of haemolysis | N/A | 4.03 | N/A | liver functions were within normal limits |
| 117 | 3.79 | ≤5 μg/dL | N/A | no | / | N/A | N/A | N/A | the hemogram, liver and renal function tests of the patient were normal except for a few parameters |
| 118 | 5.56 | <14 μgm/dl; <20 μg/dl for adults & <10 μg/dl in children | N/A | yes | normocytic normochromic anaemia with increased polychromasia; haemolysis | yes | 4.72 | N/A | raised bilirubin (indirect hyperbilirubinemia) and transaminases; bilirubin-2.3g/dl (predominantly indirect), aspartate aminotransferase (AST)-146 IU/L, alanine aminotransferase (ALT)-180 IU/L, alkaline phosphatase-195 IU/L |
| 119 | / | / | blood Cd: normal (1,1 µg/l); urine Cd: 58 µg/g creatinine (29x higher than the normal -> urinary cadmium excretion increased to 58 μg/g Cr, normal <2 μg/g Cr) | no | N/A | N/A | 8.25 (normal) | 39.1% | total bilirubin 0.4 mg/dL, GOT 23 IU/L, GPT 22 IU/L; albumin 4.0 g/dL |
| 120 | 5.91 | N/A | N/A | no | N/A | N/A | 8.81 (normal) | N/A | N/A |
| 121 | 5.56 | " | N/A | yes | N/A | yes | 4.96 | N/A | N/A |
| 122 | 2.04 | " | N/A | N/A | N/A | N/A | N/A | N/A | N/A |
| 123 | 2.8 | <5 μg/dl | N/A | yes | N/A | yes | under normal range | 27% | N/A |
| 124 | 4.12 | <0.48μmol/L | N/A | yes | normochromic, normocytic anaemia | yes | under normal range | N/A | N/A |
| 125 | 2.71 | <30 µg/dl | N/A | yes | N/A | yes | 5.83 | N/A | AST 139 IU/L and ALT 186 IU/L |
| 126 | 4.93 | normal < 10 μg/dL | N/A | yes | normochromic normocytic anemia | yes | 4.78 | 22.6% | liver function abnormalities; total bilirubin: 1,35 mg/dl (<1,2 mg/dl); AST: 54 U/L (0-34 U/L); ALT: 90 U/L (0-34 U/L) |
| 127 | 2.46 | " | N/A | N/A | N/A | N/A | N/A | N/A | N/A |
| 128 | 4.83 | " | N/A | N/A | N/A | N/A | N/A | N/A | N/A |
| 129 | 3.05 | <5 µg/dl | N/A | yes | poikilocytosis, polychromasia, anisocytosis | yes | 5.65 | N/A | Liver enzymes, bilirubin and LDH were mildly elevated |
| 130 | 3.65 | <35 µg/dl | N/A | yes | hypochromic microcytic anemia; the red blood cell morphology showed anisocytosis | yes | 5.15 | N/A | liver function was unremarkable |
| 131 | 5.07 | " | N/A | N/A | N/A | N/A | N/A | N/A | markedly elevated liver enzyme levels (ALT 1083 U/L, AST 972 U/L) |
| 132 | N/A | N/A | N/A (not measured) | no | N/A | N/A | 8.38 | N/A | total bilirubin 0.5 md/dl, and direct bilirubin 0.1 mg/dl |
| 133 | 4.5 | adult men <100 μg/L, equivalent to 4.8 μmol/L | N/A | yes | normochromic normocytic anaemia; no evidence of haemolysis | yes | 5.96 | N/A | N/A |
| 134 | / | / | urinary total arsenic concentration was increased: 240,7 μg As/l; the levels of blood mercury and lead were normal | no | / | no | normal | normal | N/A (no abnormalities) |
| 135 | 1.59 | N/A | N/A | no | / | no | normal | normal | N/A (no abnormalities) |
| 136 | 1.86 | " | N/A | no | / | no | normal | normal | N/A (no abnormalities) |
| 137 | 3.09 | <10 μg/dL | N/A | N/A | N/A | N/A | N/A | N/A | N/A |
| 138 | 0.77 | " | N/A | N/A | N/A | N/A | N/A | N/A | N/A |
| 139 | 2.37 | " | N/A | N/A | N/A | N/A | N/A | N/A | N/A |
| 140 | 5 | Lead permissible: <400 µg/L; MERCURY: permissible <15 µg/L | ULL: 246 µg/l (permissible: <70 μg/L); blood mercury level was below the permissible level (permissible: <15 μg/L) | yes | N/A | yes | 4.47 | N/A | N/A |
| 141 | 4.04 | N/A | N/A | yes | microcytic hypochromic anemia, no haemolysis | no | 5.96 | N/A | N/A |
| 142 | 2.03 | " | N/A | N/A | N/A | N/A | N/A | N/A | N/A |
| 143 | 2.51 | " | N/A | N/A | N/A | N/A | N/A | N/A | N/A |
| 144 | 1.16 | " | N/A | N/A | N/A | N/A | N/A | N/A | N/A |
| 145 | 4.88 | N/A | N/A | yes | normocytic anemia | yes | 6.02 | N/A | mildly elevated liver enzymes |
| 146 | 3.48 | <25 µg/dl | N/A | yes | normocytic anemia | N/A | 5.59 | N/A | liver function tests unremarkable; haptoglobin, and lactose dehydrogenase (LDH) were all within normal limits |
| 147 | 4.93 | 0.5-10 µg/dl | N/A | yes | microcytic anemia | N/A | 6.14 | N/A | total bilirubin 3.5 mg/dL (direct bilirubin 1.1 mg/dL), SGOT 90 mg/dL, SGPT 160 mg/dL; ALAU - Delta-aminolevulinic acid: 14,89 mg/g creat (reference value: <4,5) |
| 148 | 5.07 | <10 μg/dL for children and <25 μg/dL for adults | N/A | yes | microcytic hypochromic picture | N/A | 5.21 | N/A | Biochemical tests including renal and liver function tests were normal. |
| 149 | 3.73 | <10 µg/dl | N/A | yes | microcytic hypochromic anemia | yes | 3.79 | N/A | N/A |
| 150 | / | N/A | N/A | yes | N/A | N/A | under normal range | N/A | liver enzymes were only slightly elevated |
| 151 | 0.29 (normal) | normal <10 µg/dl; blood ARSENIC level: normal <18 µg/l | urinary arsenic level: 541 µg/g creatinine (normal: <100 µg/g creatinine), mercury level of 5,8 µg/l (normal: <5 µg/l); BLL: 5,9 µg/dl (normal: <10 µg/dl), arsenic blood level: 6,5 µg/l (normal: <18 µg/); during the chelation therapy the excretion peak of urine arsenic was 827 µg/g creatinine and mercury was 291 µg/d (normal: <15 µg/d) | yes | normocytic anemia | N/A | 5.83 | N/A | N/A |
| 152 | 10.92 | " | N/A | yes | microcytic anemia | N/A | 4.41 | N/A | elevated alanine transaminase (82 U/L) and aspartate transaminase (64 U/L) levels |
| 153 | 3.5 | 0.0–0.47 µmol/l | N/A | yes | N/A | yes | 6.02 | N/A | Liver function tests were within normal range |
| 154 | / | / | N/A | yes | N/A | N/A | 6.89 | N/A | serum lactate dehydrogenase 2000 U/L |
| 155 | 4.54 | 0.0-4.9 μg/dL | N/A | yes | N/A | N/A | 3.97 | N/A | N/A |
| 156 | / | antemortem blood ARSENIC level: 18 μg/L | post-mortem blood arsenic level: 1225 μg/L | yes | microcytic anemia | N/A | 6.14 | N/A | alanine transaminase 83 U/L (normal <40 U/L), aspartate transaminase 115 U/L (normal 5–45 U/L) |
| 157 | 3.9 | N/A | urinary lead level: 208,8 µg/g of creatinine | yes | hemolytic anemia | yes | 5.59 | N/A | mean corpuscular volume 55 fl, white blood cell count 11,264/cumm with 7% banded neutrophils, 73% segmented neutrophils, and 12% lymphocytes; The prothrombin time was 1.72 INR, and activated partial thromboplastin time was 58.1 s (control 27.5 s). C-reactive protein 15.4 mg/ dL (normal < 0.5 mg/dL). Arterial blood gas while on 3 L of oxygen showed pH of 7.396, PCO2 of 22.5 mmHg, PO2 of 144.9 mmHg, and HCO3 of 13.5 mmol/L. |
| 158 | / | / | 24-hour urine arsenic level was 68,441 ug/l, and the hair arsenic level was 0,489 ug/g, which were within normal limits | no | / | no | normal | normal | normal |
| 159 | 3.76 | ≤ 10 μg/dL | N/A | yes | hemolytic anemia, including a peripheral smear showing polychromasia; normocytic, normochromic anemia | yes | 5.09 | N/A | elevated bilirubin level (1.97 mg/dL), elevated indirect bilirubin, elevated lactate dehydrogenase, decreased haptoglobin |
| 160 | 0.62 | " | N/A | no | / | no | 8.5 | 39,8% | N/A |
| 161 | 6.76 | " | N/A | yes | microcytic picture | N/A | 5.9 | N/A | liver profile was normal |
| 162 | 1.28 | normal <10μg/dL; blood ARSENIC level: normal <50μg/L | elevated blood arsenic (140.6μg/L, normal <50μg/L) concentrations, elevated urine arsenic (total 871.3μg/L; inorganic 1683.8μg/L, methylated 1730.4 μg/L, and organic <5 μg/L) and lead (21.2μg/L) concentrations; 1 month after discontinuation: decreasing blood arsenic (13.6μg/L) and stable blood lead (27.7μg/dL) concentrations; testing of a 24-h urine collection demonstrated a lead concentration of 24.1μg/L and a methylated arsenic level of 37.8μg/L with undetectable inorganic and organic arsenic | yes | pancytopenia | no | under normal range | N/A | N/A |
| 163 | 7 | <40 µg/dl | N/A | yes | peripheral smear demonstrated microcytic and hypochromic picture | N/A | 4.96 | N/A | liver function tests were within normal limits |
| 164 | 5.07 | <0.48 μmol/L or 10 μg/dL | mercury and arsenic levels were also detected at very low levels | yes | normochromic normocytic anaemia | yes | 4.1 | N/A | N/A |
| 165 | / | / | As levels: blood 93,4 µg/l, 24h urine 8,9 µg/l, hair 18,2 µg/kg, nails 32,4 µg/kg | yes | pancytopenia with macrocytosis | N/A | 4.9 | N/A | elevated transaminases; Abdominal ultrasound revealed hepatomegaly with normal echotexture and cholelithiasis; AST/ALT: 43/19 IU/L |
| 166 | / | " | As levels: blood 330 µg/l, 24h urine N/A, hair 1.330 µg/kg, nails N/A | yes | N/A | N/A | 7.57 | N/A | AST/ALT: 10/19 IU/L |
| 167 | / | " | As levels: blood 133 µg/l, 24h urine 187 µg/l, hair 333 µg/kg, nails 30 µg/kg | yes | N/A | N/A | 4.34 | N/A | Liver function tests revealed mild hypoalbuminemia; AST/ALT: 11/7 IU/L |
| 168 | 3.57 | < 20 mg/dl | N/A | yes | microcytic and hypochromic | yes | 4.1 | 20.6% | N/A |
| 169 | 2.51 | " | urine lead level: 43,2 µg/l | no | / | N/A | 7.76 | N/A | N/A |
| 170 | 3.08 | 8 µg/dL or less | the blood copper concentration was 92.3 µg/dL (normal reference value: 75.0-145.0 µg/dL), which was within the normal range | yes | peripheral blood smears were performed, which showed sanguineous hypochromic hemoglobin | N/A | 4.53 | N/A | had stopped taking herbal medicine after being diagnosed with toxic hepatitis due to elevated liver enzymes and serum bilirubin levels in a blood test performed for a health checkup 1 month ago. He is being treated for toxic hepatitis on an outpatient basis and his labs are showing improvement; increased serum aspartate and alanine aminotransferase level; AST 159 IU/L and ALT 231 IU/L were significantly elevated, and blood bilirubin level was also increased to 2.0 mg/dL. |
| 171 | 2.62 | <10 µg/dl | N/A | yes | N/A | no | 8.19 | 39.3% | AST 29 U/L (reference range: 0-34 U/L), ALT: 18 U/L (reference range: 0-34 U/L) |
| 172 | 5.6 | N/A | N/A | yes | N/A | N/A | under normal range | N/A | N/A |
| 173 | 1.64 | " | N/A | N/A | N/A | N/A | N/A | N/A | N/A |
| 174 | 4.15 | " | N/A | N/A | N/A | N/A | N/A | N/A | N/A |
| 175 | 3.48 | " | N/A | N/A | N/A | N/A | N/A | N/A | N/A |
| 176 | 3.19 | " | N/A | N/A | N/A | N/A | N/A | N/A | N/A |
| 177 | 4.44 | " | N/A | yes | N/A | N/A | under normal range | N/A | N/A |
| 178 | 3.67 | " | N/A | N/A | N/A | N/A | N/A | N/A | N/A |
| 179 | 2.03 | " | N/A | N/A | N/A | N/A | N/A | N/A | N/A |
| 180 | / | / | analysis of a hair sample from the patient was obtained approximately 18 months after the use of the TM had been halted -> arsenic was present at a concentration of 2,15 μg/g (normal <1 μg/g) | N/A | N/A | N/A | N/A | N/A | N/A |
| 181 | 4.14 | investigate any single BLL ≥20 μg/dL or BLLs that are persistently ≥15 μg/dL | urine toxic metals tests revealed mercury and aluminum levels in the normal range; inorganic urine arsenic levels were slightly elevated at 53,3 μg/L (normal = <18.9 μg/L) | yes | normochromic, normocytic red blood cells | yes | 5.15 | N/A | N/A |
| 182 | 5 | ≤0.48 μmol/l [≤10 μg/dl] | N/A | yes | normocytic | yes | 5.83 | N/A | liver dysfunction |
| 183 | N/A | N/A | N/A | yes | N/A | N/A | 7.2 | N/A | N/A |
| 184 | / | / | urine arsenic test: 232 μg/L (reference value 2.62-22.7 μg/L) | N/A | N/A | N/A | N/A | N/A | liver function: Alanine Aminotransferase (ALT) 26 U/L, Alkaline Phosphatase (ALP) 173 U/L↑, γ-glutamyl transpeptidase (GGT) 128 U/ L↑, α-hydroxybutyric dehydrogenase (HBDH) 203 U/L↑; abdomen CT: diffuse enlargement and ascites formed in liver |
| 185 | 4.58 | <10 µg/dl | N/A | yes | normocytic red blood cells | yes | 5.65 | N/A | the total and indirect bilirubin and transaminases were mildly elevated |
| 186 | 3.07 | <10 μg/dl | ULL: 15,4 µg/dl | yes | microcytic | N/A | 5.46 | N/A | abnormal/increased liver function tests (extremely elevated enzyme concentrations; aspartate aminotransferase 581 U/l; alanine aminotransferase 680 U/l) -> drug induced hepatitis |
| 187 | 3.53 | <5 µg/dl | N/A | yes | N/A | N/A | 4.84 | N/A | N/A |
| 188 | 4.4 | N/A | N/A | yes | microcytic hypochromic anaemia | yes | 6.27 | N/A | N/A |
| 189 | / | / | urine level of inorganic arsenic: 1235 µg/L (normal: less than 10) | N/A | N/A | N/A | N/A | N/A | N/A |
| 190 | / | N/A | blood arsenic level: 1,76 µg/ml; arsenic concentration in skin (back, chest & right leg): 4,71 µg/g, 4,23 µg/g & 5,68 µg/g | N/A | N/A | N/A | N/A | N/A | N/A |
| 191 | 6.3 | normal <0.47 µmol/L; blood MERCURY level: normal <50 nmol/L | blood mercury level was normal at 5 nmol/L (normal < 50 nmol/L); his blood was not tested for Ars, although pooled (scalp, chest and arms) hair samples were sent for testing at this time; after a month, the results of initial testing for Ars became available. The level in his hair samples was 1.21 mg/kg (normal < 0.1 mg/kg), suggesting ongoing exposure in the 2 to 3 months prior to hospital admission. | yes | normocytic normochromic anemia | yes | under normal range | N/A | N/A |
| 192 | 7.92 | <10 μg/dL | N/A | yes | microcytic hypochromic anemia | yes | 5.4 | 27,9% | N/A |
| 193 | 1.7 | <10 μg/dL | N/A | no | / | no | 8.38 | 40,3% | N/A |
| 194 | 1.64 | 60 µg/l; different source: 42 µg/l | blood metal levels: 15 μg/L Hg | N/A | N/A | N/A | N/A | N/A | N/A |
| 195 | 2.5 | N/A | urine lead level: 131,7 µg/l (normal: 0-70,38 µg/l) | yes | N/A | N/A | 6.39 | N/A | Laboratory investigation showed Hepatitis B infection and liver function within normal range |
| 196 | / | THALLIUM: normal <10 µg/ml (blood) | blood & urine thallium levels: 988 μg/ml (normal <10 μg/ml) & 701 μg/ml (normal 10 μg/ml); Thallium concentration in her hair was 2850 ppb (normal <10 ppb) | N/A | N/A | N/A | N/A | N/A | N/A |
| 197 | 3.03 | <40 µg/dl | ULL: 823,0 µg/dl (normal range, <23 𝜇g/dL); On day 3 of chelation therapy, we noted a high body lead burden, as indicated by an increased urinary lead excretion up to 1853 𝜇g/dL/24 h. | yes | hypochromic microcytic anemia | yes | 5.83 | 27,9% | liver function test results were mildly abnormal; AST 57 U/L, ALT 87 U/L |
| 198 | 5.72 | N/A | N/A | yes | normocytic hypochromic anemia (peripheral blood smear was suggestive of normocytic hypochromic anemia which was supported by serum iron and total iron binding capacity levels) | N/A | 6.08 | 28,2 % | N/A |
| 199 | 6.8 | <0.47 µmol/l | N/A | yes | significant hypochromic normocytic anaemia | N/A | 5.4 | N/A | N/A |
| 200 | 3.17 | normal: <400 µg/L; urine lead level: normal <70.38 µg/L | ULL: 127,5 µg/l (normal < 70.38 μg/L) | yes | moderate normocytic anemia | N/A | 4.65 | N/A | liver function test showed hyperbilirubinemia (total bilirubin 56.5 μmol/L, direct bilirubin 24.9 μmol/L) and a slightly increased level of liver transaminases (aspartateaminotransferase 54 IU/L) |
| 201 | 2.36 | " | ULL: 97,1 µg/l | N/A | N/A | N/A | N/A | N/A | N/A |
| 202 | 0.87 | <5 µg/dL | N/A | no | N/A | N/A | normal | N/A | N/A |
| 203 | N/A | N/A | N/A | N/A | N/A | N/A | N/A | N/A | acute severe liver injury; Blood work revealed a total serum bilirubin level 28.8 (upper limit of normal, ULN 1.1 mg/dL), direct bilirubin 19.6 (ULN 0.2mg/dL), aspartate aminotransferase 228 (ULN 36U/L), alanine aminotransferase 302 (ULN 45U/L), alkaline phosphatase 324 (ULN 120U/L), gamma-glutamyl transferase level 563 (ULN 35U/L), albumin 3.1 (ULN 5.5 g/dL) and international normalised ratio 2.7 (normal <1.2) |
| 204 | 0.48 | " | N/A | no | / | no | 8.5 | 40,2% | N/A |
| 205 | 4.66 | <100 μg/L | the results of other heavy metals were in the normal limits (blood cadmium, 0.13 μg/L; blood chromium, 6.5 μg/L; blood nickel, 7.7 μg/L; blood manganese, 13.0 μg/L; and blood thallium, 0.08 μg/L) | yes | microcytic hypochromic anemia | yes | 4.53 | 21.8% | LDH was 420 U/L |
| 206 | 2.61 | " | N/A | N/A | N/A | yes | N/A | N/A | N/A |
| 207 | 6.13 | <400 µg/l | N/A | yes | moderate normocytic anemia, hemolytic anemia | yes | 5.52 | N/A | test results showed liver damage; A liver function test showed hyperbilirubinemia (total bilirubin level of 43.6 μmol/L, indirect bilirubin level of 27.1 μmol/L) and a slightly increased level of liver transaminases (alanine aminotransferase level of 51 U/L, aspartateaminotransferase level of 57 U/L, gamma glutamyl transpeptidase level of 164 U/L, alkaline phosphatase level of 143 U/L). |
| 208 | 0.34 | < 0.24 μmol/L | N/A | N/A | N/A | N/A | N/A | N/A | N/A |
| 209 | 4.26 | <15 μg/dL; different source: <5 μg/dL | N/A | yes | normocytic normochromic anaemia | no | 5.34 | N/A | his alanine transaminase, aspartate transaminase and alkaline phosphatase were 223 U/L, 101 U/L and 129,8 U/L |
| 210 | 1.83 | " | N/A | N/A | N/A | yes | N/A | N/A | N/A |

*Tab. S2D: Raw data table of all patient cases – Highest measured BLL (µmol/l), Other metal concentrations in blood/urine, Anemia, Kind of anemia, Basophilic stippling, Hb-level (mmol/l), Hematokrit, Liver*

| **Case num-ber** | **Kidney & urine** | **Serum iron & ferritin** | **Bone marrow** | **Reticulocyte count** | **Erythrocytes** | **Leukocytes & thrombocytes** | **Porphyrins** | **ALAD** | **Electrolytes** | **Other laboratory results** |
| --- | --- | --- | --- | --- | --- | --- | --- | --- | --- | --- |
| 1 | N/A | serum iron 26 µmol/l (140 µg / 100 ml) (normal 13-32 µmol/I (70-180 µg / 100 ml)); iron binding capacity 42 µmol/l (237 µg/100 ml) (normal 40-80 µmol/l (223-446 µg / 100 ml)) | a bone-marrow aspirate was hypercellular with dyserythropoietic erythroid hyperplasia | reticulocytes 12% | Perls's stain showed iron granules in peripheral blood red cells, normal marrow iron stores, and 16% partial and 9% complete ring sideroblasts; ESR 15 mm in 1st h (Westergren) | white cell count 6,0 x 10^9/l (6000/mm^3); platelets 434x10^9/l (434.000/ mm^3) | erythrocyte protoporphyrin 150 µmol/l (84,4 ng/l; normal: <50 µmol/l = 28 ng/l) | N/A | N/A | hydroxybutyrate dehydrogenase 278 IU/l (normal 40-125 IU/l) |
| 2 | urine spot test for coproporphyrin was strongly positive; urinalysis: proteinuria, glycosuria, generalized aminoaciduria, but no phosphaturia | N/A | bone marrow was hypercellular with normal myeloid:erythroid ratio & maturation of all three cell lines was appropriate; erythroid series was hyperplastic | reticulocyte count was 13% | N/A | N/A | N/A | N/A | Serum electrolytes were normal except for a potassium of 2.8 meq/liter | N/A |
| 3 | N/A | N/A | N/A | N/A | N/A | N/A | N/A | N/A | N/A | N/A |
| 4 | N/A | N/A | N/A | N/A | N/A | N/A | N/A | N/A | N/A | N/A |
| 5 | N/A | N/A | N/A | N/A | N/A | N/A | erythrocyte PP 263 µg/dl | N/A | N/A | N/A |
| 6 | N/A | N/A | N/A | N/A | N/A | N/A | N/A | N/A | N/A | N/A |
| 7 | Cr: 0,41 mg/dl; renal function test results were normal | N/A | N/A | N/A | MCV: 88,6 fL; MCH: 30,2 pg | WBC: 6400/mm^3; platelet: 254 10^3/mm^3 | ZPP: 48,3 ug/dl | N/A | N/A | N/A |
| 8 | Cr: 0,75 mg/dl | N/A | N/A | N/A | MCV: 93,3 fL; MCH: 32,2 pg | WBC: 6100/mm^3; platelet: 220 10^3/mm^3 | ZPP: 19,5 ug/dl | N/A | N/A | N/A |
| 9 | Cr: 0,57 mg/dl | N/A | N/A | N/A | MCV: 95,7 fL; MCH: 32,4 pg | WBC: 8500/mm^3; platelet: 210 10^3/mm^3 | ZPP: 20,9 ug/dl | N/A | N/A | N/A |
| 10 | urine analysis showed 1+ protein and a trace of sugar | N/A | N/A | N/A | blood showed Howell-Jolly bodies | N/A | N/A | N/A | N/A | N/A |
| 11 | N/A | N/A | N/A | N/A | N/A | N/A | N/A | N/A | N/A | N/A |
| 12 | N/A | N/A | N/A | N/A | N/A | N/A | N/A | N/A | N/A | N/A |
| 13 | N/A | N/A | bone marrow aspiration showed numoerus sideroblast & erythroid hyperplasia | reticulocyte count 172 X 10^9/L | presence of circulating nucleated erythrocytes | N/A | free-erythrocyte protoporphyrin level 317 µg/dL (normally 22 to 87) (5.61 [normally 0.39 to 1.54] µmol/L) | N/A | N/A | N/A |
| 14 | N/A | N/A | N/A | N/A | N/A | N/A | N/A | N/A | N/A | N/A |
| 15 | urinary coproporphyrin positive | N/A | N/A | reticulocytes 12% | N/A | 40 lymphocytes/mm^3 | N/A | N/A | serum calcium 7,0 mg/100 ml, phosphorus 4,1 mg/100 ml | Hb electrophoresis HbA and 16,3% HbF; ; CSF pressure raised, protein 107 mg/100 ml, glucose 51 mg/100 ml, culture negative |
| 16 | N/A | N/A | N/A | N/A | N/A | N/A | N/A | N/A | N/A | N/A |
| 17 | N/A | N/A | N/A | N/A | N/A | N/A | N/A | N/A | N/A | N/A |
| 18 | N/A (no abnormalities) | N/A (no abnormalities) | N/A (no abnormalities) | N/A (no abnormalities) | N/A (no abnormalities) | N/A | N/A (no abnormalities) | N/A (no abnorma-lities) | N/A | N/A (no abnormalities) |
| 19 | N/A | N/A | N/A | N/A | N/A | N/A | N/A | N/A | N/A | N/A |
| 20 | N/A | N/A | N/A | N/A | N/A | N/A | N/A | N/A | N/A | N/A |
| 21 | urine prophyrin and coproporphyrin were not detectable | serum iron was 16 µmol/l and ferritin 2000 pmol/l (normal 89-642 pmol/l) | N/A | reticulocytes 4,6% | N/A | white blood cell count 7,4x10^9/L; platelets 432x10^9/L | N/A | D-alanine laevulinic acid dehydratase was 32 Burch units (normal: 110-280 units) | serum calcium 1,89 mmol/l, phosphate 1,68 mmol/l, serum magnesium 1,08 mmol/l | capillary Astrup showed pH 7,159, PCO2 6,83 kPa, HCO3 17,5 mmol/l and B.E.-10.2.; blood zinc was 11,9 µmol/l (normal 10-19 µmol/l) |
| 22 | urine contained some haemoglobin | N/A | N/A | raised reticulocyte count (15%) | erythrocyte fragmentation | N/A | N/A | N/A | N/A | N/A |
| 23 | N/A | N/A | N/A | reticulocytosis (2,7x10^9/L) | N/A | normal leukocyte and platelet counts | N/A | N/A | N/A | the results of serum chemistry and other diagnostic tests were unremarkable, except for an elevated blood lead level |
| 24 | N/A | N/A | sideroblastic anemia (was diagnosed based on a bone marrow biopsy) | N/A | N/A | N/A | free erythrocyte protoporphyrin: 226 µg/dl | N/A | N/A | N/A |
| 25 | normal urinary porphobilinogen (5.8 mol/l, normal less than 8.8) | N/A | bone marrow: erythroid hyperplasia with prominent siderotic granules & ringed sideroblasts | N/A | occasional circulating nucleated red cells | N/A | N/A | N/A | N/A | N/A |
| 26 | N/A | N/A | N/A | N/A | N/A | N/A | N/A | N/A | N/A | N/A |
| 27 | urobilinogen( + ), bilirubin and ketones.; Urinary porphyrin levels were increased to four times the upper limit of normal | normal | N/A | reticulocyte count of 4% | N/A | white blood cells 8.6 x 10'/L and platelets 250 x 10'/L | N/A | N/A | electrolytes and calcium levels were normal | amylase normal; vitamin B'2 & folate normal |
| 28 | N/A | N/A | N/A | N/A | N/A | N/A | N/A | N/A | N/A | N/A |
| 29 | N/A | N/A | N/A | N/A | N/A | N/A | N/A | N/A | N/A | N/A |
| 30 | N/A | N/A | N/A | N/A | N/A | N/A | N/A | N/A | N/A | N/A |
| 31 | urinary porphyrin screen was positive; Blood urea, electrolytes and dipstick urinalysis were normal; urinary-5-aminolevulinic acid (5-ALA) 706 µmol/24 hour (< 46) and coproporphyrin (predominately III isomer) 11,600 nmol/ 24 hour (<250) | N/A | N/A | N/A | ESR 5 mm/hour | white cell count 9.4 x 109/l, platelets 265 x 109/l | Red cell zinc protoporphyrin 1,980 nmol/l (<1,600) | N/A | N/A | Glucose was 8.5 mmol/l; Amylase was normal |
| 32 | high level of urinary 𝛿-aminolevulinic acid (378 µmol/L [4,95 mg/dL]), acute intermittent porphyria; Urine porphobilinogen level was slightly elevated (12 μ /L [0.27 mg/dL]) | N/A | N/A | N/A | N/A | the white blood cell count was 7.1 x 10^9/L, with 0.72 neutrophils and 0.24 lymphocytes | 70% of the patient's cells contained elevated levels of zinc protoporphyrin (consistent with the duration of lead exposure and effect of lead on heme synthesis); ZPP level of 2,1 μmol/L | N/A | N/A | N/A |
| 33 | N/A | N/A | N/A | N/A | N/A | N/A | N/A | N/A | N/A | N/A |
| 34 | N/A | N/A | N/A | N/A | N/A | N/A | N/A | N/A | N/A | N/A |
| 35 | N/A | N/A | N/A | N/A | N/A | N/A | N/A | N/A | N/A | N/A |
| 36 | N/A | N/A | N/A | N/A | N/A | N/A | N/A | N/A | N/A | N/A |
| 37 | N/A | N/A | N/A | N/A | N/A | N/A | N/A | N/A | N/A | N/A |
| 38 | N/A | N/A | N/A | N/A | N/A | N/A | elevated blood ZPP (550 µg/dl; normal: <40) | N/A | N/A | N/A |
| 39 | N/A | N/A | N/A | N/A | N/A | N/A | N/A | N/A | N/A | N/A |
| 40 | N/A | N/A | N/A | N/A | N/A | N/A | ZPP 495 µg/dl | N/A | N/A | N/A |
| 41 | N/A | N/A | N/A | N/A | N/A | N/A | ZPP 109 µg/dl | N/A | N/A | N/A |
| 42 | N/A | N/A | N/A | N/A | N/A | N/A | ZPP 170 µg/dl | N/A | N/A | N/A |
| 43 | N/A | N/A | N/A | N/A | N/A | N/A | N/A | N/A | N/A | N/A |
| 44 | N/A | N/A | N/A | N/A | N/A | N/A | N/A | N/A | N/A | N/A |
| 45 | N/A | N/A | N/A | N/A | N/A | N/A | N/A | N/A | N/A | N/A |
| 46 | N/A | N/A | N/A | Retics/10^3 Rbc: 14 (normal: 5-15) | Rbc (10^12/L, red blood cells): 4,00 (normal: 3,8-4,9) | N/A | N/A | N/A | N/A | N/A |
| 47 | N/A | N/A | N/A | Retics/10^3 Rbc: 19 (-> reticulocytes increased, normal: 5-15) | Rbc (10^12/L): 4,29 (-> erythrocytes decreased, normal: 4,4-5,8) | N/A | N/A | N/A | N/A | N/A |
| 48 | N/A | N/A | N/A | Retics/10^3 Rbc: 10 (normal: 5-15) | Rbc (10^12/L): 4,00 (normal: 3,8-4,9) | N/A | N/A | N/A | N/A | N/A |
| 49 | N/A | N/A | N/A | Retics/10^3 Rbc: 14 (normal: 5-15) | Rbc (10^12/L): 4,70 (normal: 4,4-5,8) | N/A | N/A | N/A | N/A | N/A |
| 50 | N/A | N/A | N/A | N/A | red blood cell (RBC) count of 3.67 × 10^12/L; 10 days later: RBC count was 3.68 × 10^12/L (reference range 4.0-5.5 × 10^12/L) | N/A | N/A | N/A | N/A | N/A |
| 51 | N/A | N/A | N/A | Retics/10^3 Rbc: 8 (normal: 5-15) | Rbc (10^12/L): 4,28 (normal: 3,8-4,9) | N/A | N/A | N/A | N/A | N/A |
| 52 | N/A | N/A | N/A | N/A | N/A | N/A | N/A | N/A | N/A | N/A |
| 53 | N/A | N/A | N/A | N/A | N/A | N/A | N/A | N/A | N/A | creatine kinase 747 U/L (reference 30–135 U/L), XK isoenzyme MB 14.8 ng/mL (reference 0–6.8 ng/mL); |
| 54 | N/A | N/A | N/A | N/A | N/A | N/A | ZPP 1.35mg/L | N/A | N/A | N/A |
| 55 | urine positive for coproporphyrin fluorescence, the 24h excretion of ∂-aminolevulinic acid was 51.9 mg/day (normal, 0 to 7.5 mg/day), and the 24-hour excretion of lead was 281 Hg/day (normal, 0 to 80 /µg/day). The free RBC protoporphyrin level was 403 µg per 100 milliliters RBCs (normal, 40 to 100 µg per 100 milliliters RBCs), and the RBC uroporphyrinogen-1-synthetase activity was 61.9 nmole (normal, 13 to 26 nmole). | the serum iron to iron binding capacity ratio was 98:260 mg/dl, | the bone marrow biopsy specimen showed moderate erythroid hyperplasia with ringed sideroblasts | reticulocyte count of 5,7% (a few days later: 7.8% reticulocytes) and 5 to 10 cells with basophilic stippling per high-power field) | A Heinz body preparation was reported as positive; The ESR was 28 mm/hr (normal, 0 to 20 mm/hr); Erythrocyte pyrimidine 5'-nucleotidase levels were severely diminished | N/A | N/A | N/A | N/A | Hemoglobin electrophoresis showed an increased level of HbA2 fraction |
| 56 | Cr: 0,74 mg/dl; renal function test results were normal | N/A | N/A | N/A | MCV: 86,4 fL; MCH: 29,9 pg | WBC: 5700/mm^3; platelet: 242 10^3/mm^3 | ZPP: 28,1 ug/dl | N/A | N/A | N/A |
| 57 | Cultures of blood and urine samples were negative; A urine sample was sent out for porphyria screening. Her porphyria screen showed elevated levels of coproporphyrin III and δ-aminolevulinic acid | bone marrow biopsy showed a moderate to marked increase in iron storage. | bone marrow biopsy showed a moderate to marked increase in iron storage | N/A | mean corpuscular volume of 88.5 (normal 80.0– 98.0) fL | N/A | N/A | N/A | Levels of electrolytes, calcium, magnesium, phosphate were normal | Levels of vitamin B12 and thyroid-stimulating hormone were normal |
| 58 | creatinine, 1.2 mg per deciliter (106 mmol per liter); uric acid, 5.9 mg per deciliter (351 mmol per liter); the finding that the concentration of aminolevulinic acid in urine was elevated by a factor of 41, while the urinary concentration of porphobilinogen was increased by a factor of 4; urinary excretion of porphyrins and their precursors was elevated: aminolevulinic acid, 204 mg (1556 mmol) per day (normal, <5 mg [38 mmol] per day); porphobilinogen, 8 mg (35 mmol) per day (normal, <2 mg [9 mmol] per day); uro-porphyrin, 76 mg (92 nmol) per day (normal, <30 mg [36 nmol] per day); coproporphyrin I, 556 mg (849 nmol) per day (normal, <60 mg [92 nmol] per day); and coproporphyrin III, 16,470 mg (25,150 nmol) per day (normal, <180 mg [275 nmol] per day). | serum iron concentration: 150 mg/dl (26.865 µmol/l) | N/A | reticulocyte count: 45% | normal red-cell indexes; the erythrocyte sedimentation rate was 40 mm per hour (by the Westergren method); a few target cells | the white-cell count was 8300 per cubic millimeter, with a normal differential count; the platelet count was 214,000 per cubic millimeter | blood zinc protoporphyrin concentration: 659 µmol per mole of hemoglobin (normal range:30-80) | N/A | calcium, 9.7 mg per deciliter (2.4 mmol per liter) | serum: glucose, 175 mg/dl (9.7 mmol/liter); total cholesterol, 189 mg/dl (4.9 mmol/liter); triglycerides, 191 mg per deciliter (2.2 mmol per liter) |
| 59 | increased urinary excretion of ∂-aminolaevulinic acid; The increased urinary excretion of ∂-aminolaevulinic acid of 200 mg/l (normal <6 mg/l) indicated an earlier much higher blood lead concentration | Serum iron was increased to 40 μmol/l (normal 10.7–28.7 μmol/l); Serum ferritin was in the upper normal range | bone marrow biopsy showed a slight erythropoietic hyperplasia with siderosis of reticulum cells in an otherwise normal bone marrow. | reticulocyte count was 5,7% (normal 0,5-1,5%) | N/A | N/A | N/A | N/A | N/A | N/A |
| 60 | N/A | N/A | N/A | N/A | N/A | N/A | N/A | N/A | N/A | N/A |
| 61 | raised excretion of porphobilinogen and ∂-aminolevulinic acid in the urine sample | no signs of iron deficiency | N/A | N/A | N/A | N/A | N/A | N/A | N/A | N/A |
| 62 | renal function tests and electrolytes and urine analysis were all normal | N/A | N/A | normal | normal | normal | N/A | N/A | N/A | N/A |
| 63 | N/A | N/A | N/A | N/A | N/A | N/A | N/A | N/A | N/A | N/A |
| 64 | N/A | N/A | N/A | N/A | N/A | N/A | N/A | N/A | N/A | N/A |
| 65 | N/A | N/A | N/A | N/A | erythrocytes: 2.75 million/mm^3 | leukopenia (2700 leukocytes/mm^3) with relative (16%) and absolute neutropenia | N/A | N/A | N/A | N/A |
| 66 | porphyrin screen was done, which showed increased urine porphyrins suggesting the possibility of an acute porphyrin; total urine porphyrin: 2017 nmol/l (20-320); urea & creatinine normal | N/A | N/A | normal | erythrocyte sedimentation rate normal | normal | raised ZPP concentrations & porphyrins; ZPP: 145 µmol/mol Hb (<70); total faeces porphyrin: 435 nmol/g dry wt (10-200) | N/A | sodium, potassium, calcium normal | Serum amylase, C-reactive protein & glucose normal |
| 67 | N/A | N/A | N/A | elevated reticulocyte count 0,12 (normal range: 0,005-0,015) | target cells, increased sideroblasts, occasional ringed sideroblasts | low lymphocyte count 1,0x10^9/L (normal range: 1,0-4,8x10^9/L) | N/A | N/A | N/A | N/A |
| 68 | renal function tests were normal. | N/A | N/A | N/A | N/A | N/A | N/A | N/A | N/A | N/A |
| 69 | N/A | N/A | N/A | N/A | N/A | N/A | N/A | N/A | N/A | N/A |
| 70 | Blood urea 34 mg/dl; serum creatinine 0,8 mg/dl | N/A | N/A | N/A | N/A | thrombocytopenia | N/A | N/A | hyponatremia | N/A |
| 71 | N/A | N/A | N/A | N/A | N/A | N/A | N/A | N/A | N/A | N/A |
| 72 | N/A | N/A | N/A | N/A | N/A | N/A | N/A | N/A | N/A | N/A |
| 73 | creatinine (Cr) 105mmol/L; blood urea nitrogen (BUN) 7mmol/L; uric acid 169 umol/L | serum ferritin 305ng/ml | N/A | reticulocyte 15.1% | mean corpuscular volume (MCV) 76.8fl, mean corpuscular hemoglobin (MCH) 25.1pg/cell | white blood count (WBC) 10.0x10^3/mm^3, platelets 218x10^3/mm^3 | N/A | N/A | sodium (Na) 138mmol/L, potassium (K) 3.6mmol/L, chloride (Cl) 99mmol/L, bicarbonate radical (HCO3) 25mmol/L, calcium (Ca) 2.3mmol/L | random blood sugar 6.1mmol/L, serum amylase 25U/L, total protein 64gm/dl, physical therapy 14.1sec; G6PD was normal; INR 1.0, PTT 37 sec |
| 74 | N/A | N/A | N/A | N/A | N/A | N/A | N/A | N/A | N/A | N/A |
| 75 | N/A | N/A | N/A | N/A | N/A | N/A | N/A | N/A | N/A | N/A |
| 76 | N/A | N/A | N/A | N/A | N/A | N/A | N/A | N/A | N/A | N/A |
| 77 | N/A | N/A | N/A | N/A | basophilic stippling was not seen on the infant’s blood films, although Heinz bodies were present | N/A | concentration of erythrocyte porphyrins was 20.3 µmol/L (normal range, 0.4–1.7 µmol/L) | N/A | N/A | N/A |
| 78 | N/A | serum iron was decreased at 25 μg/dl, ferritin was normal | bone marrow biopsy revealed a marked dyserythropoiesis with 50% ring sideroblasts (Bone marrow cytology revealed marked dysplasia of the erythropoiesis with unremarkable granulopoiesis and megakaryopoiesis) | reticulocytes were elevated at 5.4% | erythrocyte count 3.23 million/μl | N/A | N/A | N/A | N/A | CK was elevated at 71 U/l, vitamin B6 was slightly decreased at 3.7 ng/ml |
| 79 | urine showed a trace of sugar, no protein or albumin; Though her hair and nails contained elevated levels of arsenic, the urine arsenic concentration was within the normal range | N/A | bone marrow aspiration was normal | Reticulocytes were 1.5% | ESR of 8mm/h | WBC of 3500/mm3 with a normal differential; platelet count 68,000/mm^3 | N/A | N/A | N/A | glucose levels of 186mg/dL (fasting), and 208 mg/dL post-prandial |
| 80 | urinalysis was normal | N/A | N/A | N/A | N/A | N/A | N/A | N/A | N/A | N/A |
| 81 | N/A | N/A | normal | N/A | N/A | leukopenia (2800/mm3), thrombocytopenia (46,000/mm3) | N/A | N/A | N/A | N/A |
| 82 | positive finding of urinary hemosiderin; creatinine 0.7 mg/dL; BUN (blood urea nitrogen) 19.0 mg/dL | N/A | bone marrow biopsy showed a mild hypocellular marrow with 40% cellularity (bone marrow hypocellularity) & erythroid hyperplasia | 11.5% reticulocytes | N/A | white cell count of 2.1×109/L (neutrophil: 54.7%, lymphocyte: 31.6%, monocyte: 9.8%, eosinophil: 0.5%), with platelets 107×10^9/L | N/A | N/A | N/A | total serum protein 6.6 g/dL; coagulation profile revealed a prothrombin time of 11.4 sec (control: 12.5 sec), a partial thromboplastin time of 33.8 sec (control: 28 to 40 sec), and a fibrinogen level of 158 mg/dL. |
| 83 | N/A | N/A | N/A | N/A | N/A | N/A | N/A | N/A | N/A | N/A |
| 84 | N/A | N/A | N/A | N/A | N/A | N/A | N/A | N/A | N/A | N/A |
| 85 | N/A | N/A | N/A | N/A | N/A | N/A | N/A | N/A | N/A | N/A |
| 86 | N/A | N/A | N/A | N/A | N/A | N/A | FEP: 487 µg/dl | N/A | N/A | N/A |
| 87 | N/A | N/A | N/A | N/A | N/A | N/A | N/A | N/A | N/A | N/A |
| 88 | N/A | N/A | N/A | N/A | N/A | N/A | N/A | N/A | N/A | N/A |
| 89 | N/A | N/A | N/A | N/A | N/A | N/A | N/A | N/A | N/A | N/A |
| 90 | N/A | N/A | N/A | N/A | N/A | N/A | N/A | N/A | N/A | N/A |
| 91 | N/A | N/A | N/A | N/A | N/A | N/A | N/A | N/A | N/A | N/A |
| 92 | N/A | N/A | N/A | N/A | N/A | N/A | N/A | N/A | N/A | N/A |
| 93 | N/A | N/A | N/A | N/A | N/A | N/A | N/A | N/A | N/A | N/A |
| 94 | N/A | N/A | N/A | N/A | N/A | N/A | N/A | N/A | N/A | N/A |
| 95 | N/A | N/A | N/A | N/A | N/A | N/A | N/A | N/A | N/A | N/A |
| 96 | elevated retention values (creatinine 2.1 mg/dl, urea 58 mg/dl), hyponatremia (sodium 125 mval/l) and hypochloremia (chloride 91 mval/l) | Ferritin increased with 205 μg/l | In bone marrow histology hyperplasia of erythropoiesis | reticulocytes 54% | N/A | Leukocyte count was elevated at 14 000/μl | N/A | N/A | Serum potassium normal at 4 mval/l | N/A |
| 97 | ∂-ALA (24h urine): 105,9 mg/day; Neither glucosuria nor proteinuria was detected on urinalysis | N/A | basophilic stippling cells were noted in both bone marrow aspiration and peripheral blood smear, in which RNA is condensed around the destroyed RBC | reticulocyte: 1,89% | N/A | N/A | ZPP: 200,62 µg/dl | N/A | N/A | N/A |
| 98 | Neither glucosuria nor proteinuria was detected on urinalysis | N/A | basophilic stippling cells were noted in both bone marrow aspiration and peripheral blood smear, in which RNA is condensed around the destroyed RBC | reticulocyte: 3,5% | N/A | N/A | N/A | N/A | N/A | N/A |
| 99 | urinalysis had a negative result for protein | iron spectrum was not abnormal; normocytic anaemia without haemolysis and with normal iron load | N/A | Erythrocyte-indexed reticulocyte count was 24 fl (2-20) | BSE: 22 mm/1st hour (0-10); 'mean corpuscular volume' (MCV): 81 fl (80-100); some "teardrop" cells; A left shift was seen with 82 segmental nuclei, 13 lymphocytes and further anisocytosis | platelet count 313 × 10^9/l (150-400). There was minor leukocytosis: 12.8 × 10^9/l (3-10). | N/A | N/A | N/A | C-reactive protein (CRP): 8 mg/l (0-8) |
| 100 | N/A | N/A | N/A | N/A | N/A | N/A | N/A | N/A | N/A | N/A |
| 101 | elevations of delta-aminolevulinic acid (11.2 μmol/mmol creatinine; n < 3) and coproporphyrins (113 nmol/mmol creatinine; n < 20) in the urine | N/A | N/A | N/A | erythrocyte protoporphyrin: 12500 nmol/L (n < 1900) | N/A | N/A | N/A | N/A | N/A |
| 102 | The renal functions were normal. Urine porphobilinogen was negative | serum iron studies were within normal limits | N/A | N/A | N/A | N/A | N/A | N/A | N/A | N/A |
| 103 | N/A | an abnormal haem compound found elevated in iron deficiency anemia | N/A | N/A | N/A | N/A | high ZPP levels of 0,832 µmol/l (normal range ≤0,56 µmol/l) | N/A | N/A | N/A |
| 104 | N/A | N/A | N/A | N/A | N/A | N/A | N/A | N/A | N/A | N/A |
| 105 | Renal function tests and urine analysis were within normal limits | N/A | N/A | N/A | N/A | white blood count of 10,000/uL and platelet count of 490,000/uL | N/A | N/A | N/A | N/A |
| 106 | N/A | N/A | N/A | N/A | normal | N/A | N/A | N/A | N/A | N/A |
| 107 | Renal function was normal. | normal iron level | bone marrow aspirate and trephine biopsy demonstrated a marked erythropoiesis with basophilic stippling, 10% ring sideroblasts and dyserythropoiesis | elevated reticulocyte count of 0,21x10^6/μL (normal: 0,05x10^6 - 0,1x10^6/μL) | N/A | N/A | free erythrocyte protoporphyrin was 903 μg/L RBC (normal: 200-550 μg/L RBC) | N/A | N/A | N/A |
| 108 | renal function was normal | normal iron levels | bone marrow aspirate and trephine biopsy showed a marked dyserythropoiesis | elevated reticulocyte count of 0,28x10^6/μL (normal: 0,024x10^6 - 0,084 x10^6/μL) | N/A | N/A | N/A | N/A | N/A | N/A |
| 109 | Liver enzymes and the renal parameters were within normal limits indicating that the liver and kidneys were not affected at this blood lead level. | N/A | N/A | N/A | N/A | W.B.C count was progressively coming down, leucopenia | N/A | δ-Aminolevulinic acid dehydra-tase (U): 23,6 (normal range: 54.5 ± 9.8) | N/A | N/A |
| 110 | renal function tests and urine examination were wthin normal limits | N/A | N/A | N/A | N/A | N/A | N/A | N/A | sodium, potassium, calcium, and phosphate levels normal | N/A |
| 111 | N/A | N/A | N/A | reticulocyte hemoglobin concentration of 22,6 pg/L (reference >28,5 pg/L) | RBC mean corpuscular volume of 56,6 (reference >72) | N/A | blood zinc-chelated protoporphyrin concentration was 880 µg/mol of heme (reference 25-65 µg/mol for hematocrit 35%) | N/A | N/A | N/A |
| 112 | patient has CKD (not due to the lead poisoning but maybe the lead poisoning increased the CKD); showed a serum creatinine level of 118.4 μmol/l (normal 61.9–114.9 μmol; value of 88.4 μmol/l 6 years before presentation), random blood glucose value of 7.5 mmol/l (normal 3.84-7.72 mmol/l), and glycated hemoglobin of 8.6% (normal 4.9–6.7%). GFr, as estimated by the modification of Diet in renal Disease equation,1 was 55 ml/min/1.73 m2, which corresponds to stage 3 chronic kidney disease (CKD). serum electrolytes were normal. urinalysis revealed 1+ glucose, a pH of 7.0, a specific gravity of 1.010 and an albumin:creatinine ratio of 11.2 mg/g (normal range in men <17 mg/g). | N/A | N/A | N/A | N/A | N/A | N/A | N/A | N/A | N/A |
| 113 | no urinary symptoms; Persistent abdominal pain led to testing for porphyria, which revealed a negative urine porphobilinogen; a 24-hour urine collection revealed a markedly elevated aminolevulinic acid (ALA) level (87,0 mg/24 hours; normal range <6,4) | N/A | N/A | N/A | mean corpuscular volume of 80 fL | white blood cell count of 8,1 k/mm^3; platelets of 119 k/mm^3 | N/A | N/A | N/A | N/A |
| 114 | N/A | N/A | N/A | N/A | N/A | N/A | N/A | N/A | N/A | N/A |
| 115 | creatinine 0.9 mg/dL; blood urea nitrogen (BUN)-11 mg/dL | N/A | N/A | N/A | N/A | platelets 159,000/μl | N/A | N/A | N/A | TLC 5400 with normal differential count; serum proteins 6.9 g/dL with albumin 4.3 g/dl; His HbA1C was 6.3% with normal blood sugar. The vitamin B12 level in blood was 904 μg/mL; and serum folate, 18 μg/L (normal, 3-20 μg/L). |
| 116 | Metabolic and renal function parameters were within normal limits. | Although iron stores were marginally depleted, there was no evidence of chronic blood loss in stools or urine | N/A | N/A | erythroid hyperplasia | N/A | N/A | N/A | N/A | N/A |
| 117 | the hemogram, liver and renal function tests of the patient were normal except for a few parameters; elevated serum creatinine level (between 1.8 to 2.1 mg/dL) and he reported having foamy urine over the previous one month. | N/A | N/A | N/A | N/A | N/A | N/A | N/A | N/A | N/A |
| 118 | renal function evaluation did not reveal any abnormality; normal routine urine examination | N/A | N/A | The reticulocyte count was 3%; Repeat hemogram showed rising reticulocyte count of 6% and 8% respectively; reticulocytosis | N/A | The routine preoperative evaluation revealed total leucocyte count (TLC)-6,000/cumm (65% polymorphs, 35% lymphocytes), platelets-1.8 lakh/cumm | N/A | N/A | N/A | N/A |
| 119 | a renal biopsy showed degeneration of the proximal tubules with normal glomeruli; renal ultrasonography showed increased bilateral kidney diameters; serum creatinine had increased from 1.2 to 3.2 mg/dL over 8 months; β2-microblobulin was increased to 6.153 μg/g Cr; creatinine (Cr) 1.2 mg/dL; BUN 8 mg/dL | N/A | N/A | N/A | N/A | white blood cell count of 7000/µl; platelet count 404.000/µl | N/A | N/A | sodium 136 mEq/L, potassium 2.5 mEq/L, calcium 9.4 mg/dL | total protein 6.7 g/dL, albumin 4.0 g/dL |
| 120 | N/A | N/A | N/A | N/A | N/A | N/A | N/A | N/A | N/A | N/A |
| 121 | N/A | N/A | N/A | N/A | N/A | N/A | N/A | N/A | N/A | N/A |
| 122 | N/A | N/A | N/A | N/A | N/A | N/A | N/A | N/A | N/A | N/A |
| 123 | N/A | N/A | N/A | N/A | N/A | N/A | markedly increased protopor-phyrins – free erythrocyte protoporphyrin IX – FEP- 795 μg/dl | N/A | N/A | N/A |
| 124 | N/A | N/A | N/A | N/A | N/A | N/A | N/A | N/A | N/A | N/A |
| 125 | total urine porphyrin 903 mg per 24h (normal value 15-200) | N/A | N/A | reticulocytes 0,39x10^6/mm^3 | mean corpuscular volume (MCV) 81,4 fL | WBC-count 21,6x10^3/mm^3 | red cell zinc protoporphyrin 603 mmol/mol eme (normal value 20-85) | N/A | serum sodium 128 mmol/L | N/A |
| 126 | N/A | normal iron studies | N/A | reticulocyte count of 7% | MCV 87 fL | N/A | N/A | N/A | N/A | N/A |
| 127 | N/A | N/A | N/A | N/A | N/A | N/A | N/A | N/A | N/A | N/A |
| 128 | N/A | N/A | N/A | N/A | N/A | N/A | N/A | N/A | N/A | N/A |
| 129 | Increased concentrations of coproporphyrine III, delta-aminolevulinic-acid (urine) | N/A | N/A | elevated reticulocytes | N/A | N/A | elevated free- and ZPP (erythrocytes) were observed | N/A | N/A | N/A |
| 130 | renal function was unremarkable | N/A | N/A | N/A | N/A | N/A | N/A | N/A | N/A | N/A |
| 131 | N/A | N/A | N/A | N/A | N/A | N/A | N/A | N/A | N/A | N/A |
| 132 | acute kidney injury (due to interstitial nephritis); deficient renal function (his serum creatinine level was 10.3 mg/dl); acute kidney injury (AKI) due to heavy metal poisoning; blood urea 121, serum creatinine 11.33 mg/dl | N/A | N/A | N/A | N/A | N/A | N/A | N/A | serum sodium 139 mmol/l, serum potassium 3.9 mmol/l | His ABG showed compensated metabolic acidosis with a pH of 7.36, PCO2 of 28 mmHg and HCO3 of 18meq/l |
| 133 | a porphyria screen showed a urine 5-aminolevulinic acid (5-ALA) of 24.2μmol/mmol (<3.8), normal urine porphobilinogen. Urine coproporphyrin III markedly raised | iron studies normal | N/A | N/A | N/A | N/A | erythrocyte zinc protoporphyrin was subsequently found to be markedly raised | N/A | N/A | N/A |
| 134 | N/A (no abnormalities) | N/A (no abnormalities) | N/A (no abnormalities) | N/A (no abnormalities) | N/A (no abnormalities) | N/A | N/A (no abnormalities) | N/A (no abnormalities) | N/A | N/A (no abnormalities) |
| 135 | N/A (no abnormalities) | N/A (no abnormalities) | N/A (no abnormalities) | N/A (no abnormalities) | the mean red blood cell corpuscular volume was 76 fl | N/A | N/A (no abnormalities) | N/A (no abnormalities) | N/A | N/A (no abnormalities) |
| 136 | N/A (no abnormalities) | N/A (no abnormalities) | N/A (no abnormalities) | N/A (no abnormalities) | N/A (no abnormalities) | N/A | N/A (no abnormalities) | N/A (no abnormalities) | N/A | N/A (no abnormalities) |
| 137 | N/A | N/A | N/A | N/A | N/A | N/A | N/A | N/A | N/A | N/A |
| 138 | N/A | N/A | N/A | N/A | N/A | N/A | N/A | N/A | N/A | N/A |
| 139 | N/A | N/A | N/A | N/A | N/A | N/A | N/A | N/A | N/A | N/A |
| 140 | N/A | increase in serum ferroprotein | bone marrow & peripheral blood smears: extensive erythrocytes with coarse basophilic stippling; bone marrow smears showed 4+ iron stores and ring sideroblasts -> ineffective heme synthesis | N/A | N/A | N/A | N/A | N/A | N/A | N/A |
| 141 | N/A | N/A | bone marrow showed cellular marrow with increased iron stores (Perls’ stain: grade 4), with no ring sideroblasts and dyspoietic megakaryocytes | reticulocyte count of 0,06% | N/A | total leukocyte count 6.92×10^9/L and platelets 214×10^9/L | N/A | N/A | N/A | N/A |
| 142 | N/A | N/A | N/A | N/A | N/A | N/A | N/A | N/A | N/A | N/A |
| 143 | N/A | N/A | N/A | N/A | N/A | N/A | N/A | N/A | N/A | N/A |
| 144 | N/A | N/A | N/A | N/A | N/A | N/A | N/A | N/A | N/A | N/A |
| 145 | Urine porphyrins were elevated | normal serum iron & transferrin | N/A | N/A | N/A | N/A | N/A | N/A | N/A | N/A |
| 146 | urinalysis unremarkable; creatinine of 0.8 mg/dl (GFR estimate 114 ml/min/1.73 m2). | Ferritin, transferrin normal | N/A | N/A | N/A | N/A | N/A | N/A | N/A | N/A |
| 147 | hyperchromic urine; CPU – Coproporphyrin: 423 μg/dL creat (reference value: <100) | hyperferritinemia | N/A | reticulocytosis | N/A | N/A | N/A | N/A | N/A | N/A |
| 148 | Biochemical tests including renal and liver function tests were normal. | N/A | N/A | N/A | N/A | N/A | N/A | N/A | N/A | N/A |
| 149 | Urine porpho- bilinogen was negative; Urine ketone bodies were negative. | serum iron, serum ferritin normal | N/A | reticulocyte count was 10% | N/A | N/A | N/A | N/A | N/A | N/A |
| 150 | creatinine was only slightly elevated | N/A | N/A | N/A | N/A | leukopenia | N/A | N/A | N/A | serum level of total sec antigen extremely high at 38.5 ng/mL (1.5 ng/mL as the cutoff value) |
| 151 | N/A | N/A | N/A | N/A | N/A | N/A | N/A | N/A | serum potassium of 2,9 mmol/l | elevated C-reactive protein of 11,6 mg/dl (normal <0,5 mg/dl) |
| 152 | N/A | N/A | N/A | N/A | mean corpuscular volume 70 fl | N/A | N/A | N/A | sodium level of 128 mmol/L, potassium level of 2.9 mmol/L | N/A |
| 153 | N/A | N/A | N/A | N/A | N/A | N/A | N/A | N/A | N/A | N/A |
| 154 | blood urea nitrogen 71 mg/dL, serum creatinine 11 mg/dL, eGFR 4.7 mL/min, Na 127 mEq/L, K 5.7 meq/L; proteinuria; enlarged kidneys. Renal biopsy revealed normal‐looking glomeruli, tubules showed moderate neutrophilic infiltration, dilation, and cellular casts comprising neutrophils and tubular epithelial cells. Interstitium revealed edema along with mixed infiltration with neutrophils, lymphocytes, and occasional eosinophils, without any evidence of granulomas or fungal elements. Histopathology was suggestive of acute tubulointerstitial nephritis. | N/A | N/A | N/A | N/A | platelet count 3.7 × 103/μL, total leucocyte count 13,900 × 103/μL | N/A | N/A | N/A | N/A |
| 155 | N/A | N/A | N/A | N/A | N/A | N/A | N/A | N/A | N/A | N/A |
| 156 | blood urea nitrogen 35 mg/dL (normal 7–20 mg/dL), creatinine of 1.6 mg/dL (normal 0.7–1.5 mg/dL), sodium 122 mmol/L (normal 135–147 mmol/L); His urinalysis revealed ketonuria (2+) and proteinuria (1+) | N/A | N/A | N/A | mean corpuscular volume 55 fl | white blood cell count 11,264/cumm with 7% banded neutrophils, 73% segmented neutrophils, and 12% lymphocytes | N/A | N/A | N/A | prothrombin time was 1.72 INR, and activated partial thromboplastin time was 58.1 s (control 27.5 s). C-reactive protein 15.4 mg/ dL (normal < 0.5 mg/dL). Arterial blood gas showed pH of 7.396, PCO2 of 22.5 mmHg, PO2 of 144.9 mmHg, and HCO3 of 13.5 mmol/L. |
| 157 | increase in the urinary coproporphyrin III level up to 155.9 nmol/mmol (n<150nmol/mmol) | N/A | N/A | N/A | N/A | N/A | N/A | N/A | N/A | N/A |
| 158 | normal | normal | normal | normal | normal | N/A | normal | normal | normal | N/A |
| 159 | N/A | N/A | N/A | In the anemia work-up, marked reticulocytosis (9.15%) was noted | N/A | N/A | N/A | N/A | N/A | N/A |
| 160 | Cr: 0,62 mg/dl | N/A | N/A | N/A | MCV: 91,5 fL; MCH: 31,5 pg | WBC: 5500/mm^3; platelet: 214 10^3/mm^3 | ZPP: 34,1 ug/dl | N/A | N/A | N/A |
| 161 | Renal profile was normal | N/A | N/A | N/A | N/A | N/A | N/A | N/A | N/A | N/A |
| 162 | N/A | N/A | bone marrow biopsy: erythroid hyperplasia, erythroid dysplasia, reticulocytosis, small hypolobated megakaryocytes | reticulocytosis | N/A | N/A | N/A | N/A | N/A | N/A |
| 163 | kidney function tests were within normal limits | N/A | N/A | N/A | N/A | N/A | N/A | N/A | N/A | N/A |
| 164 | N/A | N/A | N/A | N/A | N/A | N/A | N/A | N/A | N/A | N/A |
| 165 | N/A | N/A | N/A | N/A | N/A | platelets: 30.000/mm^3 | N/A | N/A | N/A | N/A |
| 166 | N/A | N/A | N/A | N/A | N/A | platelets: 300.000/mm^3 | N/A | N/A | N/A | N/A |
| 167 | N/A | N/A | N/A | N/A | N/A | normal leukocyte & platelet counts; platelets: 230.000/mm^3 | N/A | N/A | N/A | N/A |
| 168 | δ-Aminolevulinic acid was found in urine | N/A | N/A | N/A | N/A | N/A | free erythrocyte protoporphyrin was 163 µg/dL, mainly ZPP (85% of erythrocyte PP) | N/A | N/A | N/A |
| 169 | N/A | N/A | N/A | N/A | N/A | N/A | no anemia, but erythrocyte porphyrin was very high (262 µg/dL), at the expense of ZPP (91%) | N/A | N/A | N/A |
| 170 | N/A | Serum iron 204 µg/dL (normal: 65-157 µg/dL), ferritin slightly elevated at 546 mg/mL (normal: 30-400 mg/mL), total iron binding capacity 319. 0 µg/dL (normal: 250-450 µg/dL), transferrin saturation was 64% | N/A | N/A | N/A | N/A | N/A | N/A | N/A | vitamin B12 and folic acid were within normal limits at 661 pg/mL (normal reference: 187-883 pg/mL) and 17.6 ng/mL (normal reference: 3.1-20.5 ng/mL), respectively. |
| 171 | normal | N/A | normal | normal | mean corpuscular volume of 80.6 fL. | N/A | ZPP elevated: 179 µmol/mol (= 285 µg/dl, normal: <69 µmol/mol) | N/A | N/A | N/A |
| 172 | N/A | N/A | N/A | N/A | N/A | N/A | N/A | N/A | N/A | N/A |
| 173 | N/A | N/A | N/A | N/A | N/A | N/A | N/A | N/A | N/A | N/A |
| 174 | N/A | N/A | N/A | N/A | N/A | N/A | N/A | N/A | N/A | N/A |
| 175 | N/A | N/A | N/A | N/A | N/A | N/A | N/A | N/A | N/A | N/A |
| 176 | N/A | N/A | N/A | N/A | N/A | N/A | N/A | N/A | N/A | N/A |
| 177 | N/A | N/A | N/A | N/A | N/A | N/A | N/A | N/A | N/A | N/A |
| 178 | N/A | N/A | N/A | N/A | N/A | N/A | N/A | N/A | N/A | N/A |
| 179 | N/A | N/A | N/A | N/A | N/A | N/A | N/A | N/A | N/A | N/A |
| 180 | N/A | N/A | N/A | N/A | N/A | N/A | N/A | N/A | N/A | N/A |
| 181 | N/A | N/A | N/A | N/A | N/A | N/A | N/A | N/A | N/A | N/A |
| 182 | N/A | N/A | N/A | N/A | N/A | N/A | N/A | N/A | Hyponatrae-mia (115 mmol/l), hypokalaemia (3,0 mmol/l) | N/A |
| 183 | Investigations revealed blood urea 174 mg/dL, serum creatinine 5 mg/dL, estimated glomerular filtration rate 12.4 mL/min, Na 125 mEq/L, K 4.5 mEq/L | N/A | N/A | N/A | N/A | platelet count 2.67 × 10^6/μL, total leucocyte count 7.9 × 103/μL | N/A | N/A | N/A | N/A |
| 184 | N/A | N/A | N/A | N/A | normal red cell count | blood platelet 69×10^9/L↓, others normal | N/A | N/A | N/A | N/A |
| 185 | N/A | N/A | N/A | reticulocyte count of 3.8% | mean corpuscular volume of 87 fL | N/A | N/A | N/A | N/A | N/A |
| 186 | The renal function was not obviously abnor- mal (creatinine 19.6 μmol/l; urea nitrogen 2.0 mmol/l). | N/A | N/A | N/A | erythrocyte count (*10^12 per liter): 3,06 (reference range children: 4-5,5); MCV (μm^3): 77,4 (reference range: 82-95) | N/A | N/A | N/A | N/A | N/A |
| 187 | N/A | N/A | N/A | N/A | N/A | N/A | N/A | N/A | N/A | HbA1c 11,8%, positive GAD-65 autoantibodies |
| 188 | N/A | N/A | N/A | N/A | red blood cell count of 3.33×10^6 cells/μL | N/A | N/A | N/A | N/A | N/A |
| 189 | N/A | N/A | N/A | N/A | N/A | N/A | N/A | N/A | N/A | N/A |
| 190 | severe renal tissue degeneration, necrosis and desquamation of renal tubular epithelial cells, presence of protein cast and a widened edematous interstitium with interstitial fibrosis | N/A | N/A | N/A | N/A | N/A | N/A | N/A | N/A | N/A |
| 191 | N/A | N/A | N/A | N/A | N/A | N/A | his erythrocyte porphyrins level was 5 umol/L (normal < 1.8 umol/L) | N/A | N/A | Parathyroid hormone level was 7.5 pmol/L (normal 1.7 – 7.3 pmol/L) |
| 192 | N/A | N/A | N/A | N/A | red blood cell: 3,31 × 10^6/mm^3; basophilic stippling teardrop of red blood cells was observed in the peripheral blood smear | N/A | N/A | N/A | N/A | N/A |
| 193 | Cr: 0,75 mg/dl; renal function test results were normal | N/A | N/A | N/A | MCV: 91,8 fL; MCH: 30,9 pg | WBC: 5900/mm^3; platelet: 370 10^3/mm^3 | ZPP: 68,8 ug/dl | N/A | N/A | N/A |
| 194 | N/A | N/A | N/A | N/A | N/A | N/A | N/A | N/A | N/A | N/A |
| 195 | normal | normal | N/A | N/A | N/A | N/A | N/A | N/A | N/A | N/A |
| 196 | N/A | N/A | N/A | N/A | N/A | N/A | N/A | N/A | N/A | N/A |
| 197 | positive urine porphyrin test result; elevated urine delta-ALA level of 81.8 mg/24 h (normal range, <7 mg/24 h) with a normal urine porphobilinogen level of 1.0 mg/24 h (normal range, <4 mg/24 h) | N/A | N/A | N/A | N/A | white blood cell count normal | N/A | N/A | electrolytes normal | N/A |
| 198 | blood urea & serum creatinine normal | serum iron & total iron binding capacity levels suggestive of anemia | N/A | N/A | RBC count: 3,79x10^12/L | N/A | N/A | N/A | N/A | baseline glycated haemoglobin: 6,9% |
| 199 | creatinine of 128 μmol/L (baseline ~100 μmol/L), indicating a mild deterioration in renal function | N/A | N/A | N/A | N/A | N/A | N/A | N/A | N/A | N/A |
| 200 | N/A | N/A | bone marrow needle biopsy but found only erythroid hyperplasia in bone marrow | raised reticulocyte of 10,2% | N/A | N/A | N/A | N/A | N/A | N/A |
| 201 | N/A | N/A | N/A | N/A | N/A | N/A | N/A | N/A | N/A | N/A |
| 202 | N/A | no evidence of iron insufficiency | N/A | N/A | N/A | N/A | ZPP levels within normal limits at 28 per dL whole blood (reference <35) and 61 per mole of heme (reference <70) | N/A | N/A | N/A |
| 203 | Renal function tests were within normal range | N/A | N/A | N/A | N/A | N/A | N/A | N/A | N/A | N/A |
| 204 | Cr: 0,74 mg/dl | N/A | N/A | N/A | MCV: 93,7 fL; MCH: 31,9 pg | WBC: 5000/mm^3; platelet: 164 10^3/mm^3 | ZPP: 25,1 ug/dl | N/A | N/A | N/A |
| 205 | N/A | N/A | bone marrow smears also found extensive erythrocytes and nucleated erythrocytes with basophilic stippling | reticulocyte percentage, 4.5%; and reticulocyte count, 0.15 × 10^12/L) | red blood cell, 3.13 × 10^12/L; MCV 69.8 fL; mean corpuscular Hb 23.3 pg; mean corpuscular Hb concentration 318 g/L | N/A | N/A | N/A | N/A | N/A |
| 206 | N/A | N/A | N/A | N/A | N/A | N/A | N/A | N/A | N/A | N/A |
| 207 | The urine and stool tests yielded normal results. | Ferritin was moderately elevated | N/A | N/A | N/A | N/A | N/A | N/A | N/A | N/A |
| 208 | N/A | N/A | N/A | N/A | N/A | N/A | N/A | N/A | N/A | N/A |
| 209 | renal function tests were within normal limits | N/A | N/A | N/A | N/A | platelets of 120×10^9/L | N/A | N/A | N/A | N/A |
| 210 | N/A | N/A | N/A | N/A | N/A | N/A | N/A | N/A | N/A | N/A |

*Tab. S2E: Raw data table of all patient cases – Kindey & urine, Serum iron & ferritin, Bone marrow, Reticulocyte count, Erythrocytes, Leukozytes & thrombocytes, Porphyrins, ALAD (Delta-aminolevulinic acid dehydratase), Electrolytes, Other laboratory results*

| **Case num-ber** | **How many tests until correct diagnosis?** | **Treatment** | **Clinical outcome** | **Laboratory results after end of therapy** | **Last BLL measured (µmol/l)** |
| --- | --- | --- | --- | --- | --- |
| 1 | A plain x-ray film of a distended abdomen showed fluid levels in the small and large bowel. Chest radiographs and ECG were normal.; laboratory tests -> Common haemolytic anaemias were excluded and lead poisoning was suspected | chelation therapy: D-penicillamine (p.o.) | successful medical treatment: urinary lead excretion rose to 10,8 µmol (2240 µg)/24 h, the symptoms abated, haemoglobin rose to 14,8 g/dl, and the blood film became unremarkable | whole blood lead concentration: 1,6 µmol/l (32 µg/dl) (5 months later) | 1.55 |
| 2 | N/A; Lumbar puncture; Roentgenography of the knees revealed increased densities consistent with "lead lines" at the distal femoral and proximal tibial epiphyses; electroencephalogram (EEG) | chelation therapy: CaNa2EDTA | successful medical treatment; all laboratory data, including blood and urine lead levels and urine coproporphyrin excretion, returned to normal; he responded well and is currently 3 years 5 months of age and has no evidence of physical or mental retardation or recurrence of seizures | see diagram in article: whole BLL ~40 µg/dl (after 60 days) | 1.93 |
| 3 | N/A | N/A | N/A | N/A | N/A |
| 4 | N/A | N/A (patient died) | patient died | patient died | patient died |
| 5 | was found to have lead poisoning during screening for well-baby care; X-ray films of the wrists and knees revealed dense provisional zones of calcification suggestive of lead deposits | chelation therapy: CaNa2EDTA | N/A | N/A | N/A |
| 6 | Abdomen and chest roentgenograms showed radiopaque densities in the stomach. Gastric lavage produced an aspirate which contained orange particles. Stool examination also revealed smiliar orange particles mixed with blood. On prolonged and repeated questioning, the mother finally admitted to having given the child an orange powder | no chelation therapy, discontinue medication, no further treatment (conservative therapy) | improved condition | N/A | N/A |
| 7 | N/A | no chelation therapy, discontinue medication; multivitamin capsules, green tea & garlic (conservative therapy) | successful medical treatment; Chest tightness and palpitations persisted; thus, coronary angiography was performed. No other cause for the patient's chest symptoms was found. Seven months after discontinuing S. capsule intake, the patient remains under treatment for chest discomfort in the Cardiology Department. A persistent skin rash and pain are present in the upper and lower limbs, and lethargy is present throughout; the patient continues to show improvement and exacerbation in an alternating manner. Symptomatic treatment is performed in our Dermatology and Neurology Departments. | BLL: 15,72 µg/dl (~1 month later); it returned to the normal range (9.21 μg/dL) after 90 days | 0.45 |
| 8 | N/A | N/A | N/A | BLL: 9,21 µg/dl (~1 month later) | 0.45 |
| 9 | N/A | N/A | N/A | N/A | N/A |
| 10 | Further evaluation was not possible because the patient's family took her from the hospital against medical advice. Her family was known to use azarcón. | patient's family took her from the hospital against medical advice | N/A | N/A | N/A |
| 11 | N/A | N/A | N/A | N/A | N/A |
| 12 | The infant had seizures at his home and was taken to the hospital emergency room. During a lumbar puncture, he became apneic and was transferred to another hospital. The lumbar puncture ruled out meningitis as the cause of his neurologic signs. A computerized tomography scan suggested a midline cerebellar mass with enlargements of the third and lateral ventricles in the brain. He underwent an operation for decompression of the posterior fossa. During the operation, apparent necrotic tissue was excised from the cerebellum. The infant died the next day. Postmortem examination revealed severe lead poisoning as the underlying cause of death. High concentrations of lead were found in the blood, urine, liver, and kidneys. Lead lines were seen in radiographs of the long bones. | operation (patient died) | patient died | patient died | patient died |
| 13 | Patient told about medication -> tests were done, lead poisoning was recognized | chelation therapy: D-penicillamine | successful medical treatment | BLL returned to normal | normal |
| 14 | Patient told about medication -> tests were done, lead poisoning was recognized | no chelation therapy, discontinue medication, no further treatment (conservative therapy) | although her symptoms vanished, her blood lead level declined slowly | BLL: at last report it was 60 µg/dL (2,90 µmol/L) | 2.9 |
| 15 | The mother gave a history that the child had been given Bint Al Zahab daily; brain scan showed minimally enlarged cortical sulci in frontal lobes; sickling test negative | chelation therapy: Dimercaprol (i.m.), D-penicillamine | successful medical treatment: seizure activity was promptly controlled; follow-up for 1 year after admission has not revealed any residual psychomotor or developmental deficit | BLL: 2,76 µmol/l (after 7 days of chelation therapy) | 2.75 |
| 16 | N/A; dense metaphysial lines were seen on X-ray of the wrist; brain scan: widening of Sylvian fissure | N/A | successful medical treatment | BLL: 2,12 µmol/l (after 7 days of chelation therapy) | 2.13 |
| 17 | N/A; dense metaphysial lines were seen on X-ray of the wrist; EEG: abnormal | chelation therapy: D-penicillamine; calcium supplementation (p.o.) | successful medical treatment | BLL: 3,16 µmol/l (after 7 days of chelation therapy), then 2,98 µmol/l & 2,48 µmol/l (after 1 month) | 2.46 |
| 18 | N/A | chelation therapy: D-penicillamine; calcium supplementation (p.o.) | successful medical treatment | N/A | N/A |
| 19 | N/A; dense metaphysial lines were seen on X-ray of the wrist; brain scan: mixed type of brain atrophy mainly of frontal lobe (though on follow-up gross motor functions remained normal) | chelation therapy: D-penicillamine; calcium supplementation (p.o.) | successful medical treatment; abnormal walking with support at 1,5 years | BLL: 1,9 µmol/l (after 7 days of chelation therapy) | 1.88 |
| 20 | N/A; dense metaphysial lines were seen on X-ray of the wrist | chelation therapy: D-penicillamine; calcium supplementation (p.o.) | successful medical treatment | BLL: 3,69 µmol/l (after 7 days of chelation therapy), later 2,06 µmol/l | 2.08 |
| 21 | electroencephalogram showed slow wave activity; brain computerized axial tomography was normal and showed no signs of cerebral edema; cerebrospinal fluid contained no cells, protein was 6 g/l and sugar 3,5 mmol/l; therapy: intravenous diazepam, phenobarbitone and intravenous calcium therapy for convulsions + blood transfusion -> the convulsions persisted for 24 hours but responded to intravenous calcium therapy which raised the ionized calcium from 0,9 mmol/l (normal 1,18-1,47) to 1,05 mmol/l over a 3 day period requiring 12-16 mmol calcium per day, but he remained hypotonic and hyper-reflexic with bilateral ankle clonus though his general conditionimproved -> further investigation showed elevated BLL; X-ray showed dense metaphyseal lines in the long bones (lead lines at long bone metaphyses) | chelation therapy: Dimercaprol, CaNa2EDTA (i.v.) & D-penicillamine (p.o.) | successful medical treatment; the child remained mildly hypotonic and his development was only mildly delayed; X-ray showed clearing of metaphyseal dense lines | BLL: 81,4 µg/dl (4 days after therapy start), 69,8 µg/dl (10 days after therapy start), 47,4 µg/dl (2 months later) | 2.29 |
| 22 | N/A | chelation therapy: DMSA | successful medical treatment: she recovered clinically after the first course and her haemoglobin rose to 11.1 g/dl. After the second course, the blood lead level fell to the upper limit of normal and the blood arsenic level to normal | after the second course, the BLL fell to the upper limit of normal (~250 µg/l) and the blood arsenic level to normal (<20 µg/l) | 1.21 |
| 23 | A gastroenterologist was consulted. Laboratory test results revealed a hypochromic anemia, …; The results of serum chemistry and other diagnostic tests were unremarkable, except for a blood lead level of 3.43 µmol/l | chelation therapy: CaNa2EDTA | successful medical treatment; dra¬ matically after discontinuing use of the pills and receiving chelation therapy with calcium disodium edetate. | N/A | N/A |
| 24 | In September he developed an episode of severe chest pain, which was treated with anti-inflammatory medications; bone marrow biopsy; Although the patient’s very recent use of Indian herbal medicine was mentioned several times in his hospital records, none of his physicians ordered the substance analyzed. Diagnosis of lead toxicity was delayed until after a bone marrow biopsy. The origin of the lead was then incorrectly ascribed to the workplace until careful evaluation, delayed until an occupational medical consultation a few months later, uncovered the actual etiology. | chelation therapy: D-penicillamine (p.o.) | successful medical treatment | BLL: 33 µg/dl (1,6 µmol/l) (4 months later, therapy was in November, control in March), 28 µg/dl (1,35 µmol/l) (August) | 1.35 |
| 25 | N/A; Only after direct questioning did he admit to having taken three preparations | no chelation therapy, discontinue medication, no further treatment (conservative therapy) | successful medical treatment | BLL: 55 µg/dl (after 4 weeks) | 2.66 |
| 26 | mentioned as an aside that about six weeks previously he had begun taking a medication from India for the treatment of back pain; was employed in a lead-acid battery manufacturing plant in Canada between 1988 and 1990. The employee was a quality control worker, an occupation of moderate exposure in a facility where employee blood lead values generally run 50 µg/dL or lower. As part of a routine lead exposure control program, periodic blood samples were collected from this worker approximately every three months. Beginning in early May 1990, a 32 µg/dL increase in the blood lead test values for this worker was noted. Other workers in the same job classification and in the plant generally did not fluctuate significantly,nor were any changes in plant ventilation, working conditions, or airborne exposttrc noted which could account for the sudden increase. | no chelation therapy, discontinue medication, no further treatment (conservative therapy) | BLL began to fall shortly after stopping the intake | BLL: 44 µg/dl (2,5 months later) | 2.13 |
| 27 | He had been prescribed cimetidine (400 mg/day) by his general practitioner for abdominal pain, and had been taking this for two days prior to admission.; A number of examinations and laboratory investigations were performed -> all normal / negative results; abdominal ultrasound showed no evidence of biliary obstruction or liver abscesses; treatment included rehydration with intravenous infusions, pethidine for abdominal pain and enemata for constipation -> the symptoms gradually abated over a period of one week and liver function improved -> patient was discharged without a firm diagnosis for further investigation as an outpatient -> he represented one day later with recurring severe colicky abdominal pain and nausea; abdominal radiograph showed dilatation of the caecum, but gas in the rectum, consistent with colonic pseudoobstruction -> abdominal pain persisted and he underwent a colonoscopy, which showed no abnormality -> soon after, the results of blood lead estimations became available | chelation therapy: CaNa2EDTA (i.v.) | successful medical treatment | urinary lead excretion rose to 81,9 µmol in the first 24h after therapy, falling to 19,8 µmol/24h by day 5; BLL had fallen to 2,9 µmol/L one week after the initiation of chelation therapy; haemoglobin level had risen to 12,2 g/dL (2 weeks later) | 2.9 |
| 28 | N/A | chelation therapy: DMPS, DMSA | 2 years after the onset of symptom the patient was still unable to return to work (recovery from arsenical neuropathy is generally poor, even after treatment with chelating agents) | N/A | / |
| 29 | Standard laboratory investigations, including thyroid function tests, all gave normal findings | N/A | N/A | N/A | / |
| 30 | patient was tested for lead poisoning as part of a routine well-child examination in Los Angeles | N/A | N/A | N/A | N/A |
| 31 | Abdominal examination revealed minimal epigastric tenderness. Examination was otherwise unremarkable. Serology to hepatitis viruses A,B,C, cytomegalovirus and Epstein-Barr virus was negative. Urinary porphyrinscreen was positive -> further investigations (BLL, ...) | chelation therapy: CaNa2EDTA (i.v.) | successful medical treatment: dramatic improvement in his symptoms, haematological and biochemical indices. | N/A | N/A |
| 32 | Physical examination, including neurological examination, was normal, and a chest radiograph was normal except for an old granuloma. Abdominal pain was treated with intramuscular meperidine. In the hospital the patient did not ingest the herbal tea. He was discharged June21 with the diagnoses of acute intermittent porphyria, spastic colon, and anemia. A blood sample drawn during the hospital stay subsequently revealed a BLL of 76 μg/dL and a ZPP level of 2.1 μmol/L. These results were reported to the New York City Department of Health (NYCDOH) by the commercial laboratory performing the tests. When contacted by an NYCDOH investigator, the patient stated that he suspected the herbal medicine to be the source of his lead exposure. | chelation therapy: DMSA | successful medical treatment | BLL: 2,4 µmol/l (49 µg/dl) (after first course), 1,9 µmol/l (39 µg/dl) (a week after second course); 0,9 µmol/l (18 µg/dl) (13 months after initial visit) | 0.87 |
| 33 | N/A | N/A | N/A | N/A | / |
| 34 | N/A | N/A | N/A | N/A | N/A |
| 35 | N/A | N/A | N/A | N/A | / |
| 36 | N/A | N/A | N/A | N/A | N/A |
| 37 | N/A | N/A | N/A | N/A | N/A |
| 38 | She was then admitted with acute abdominal pain, and on investigation was found to have anaemia with basophilic stippling of red blood cells. Blood lead was elevated, but direct questioning and investigation of her home and other family members failed to reveal a source of poisoning. She was discharged to be followed up after 6 weeks. On review, blood lead had increased further, and elevated blood zinc protoporphyrin indicated chronic lead exposure. She was readmitted for chelation therapy with penicillamine. Extensive questioning finally revealed that an aunt had brought her tablets obtained from India | chelation therapy: D-penicillamine | successful medical treatment (Three months later her haemoglobin and blood lead concentration had returned to normal. She made an uneventful recovery.) | BLL: 13 µg/dl (3 months later her haemoglobin and BLL had returned to normal) | 0.63 |
| 39 | N/A; On examination he was clinically anaemic, but there were no other abnormal findings. Investigation confirmed lead poisoning. On questioning he said that he had been taking an Asian remedy | N/A | BLL fell slowly with improvement in his clinical condition | BLL: 23 µg/dl (follow up) | 1.11 |
| 40 | N/A; On examination she had evidence of a peripheral neuropathy in addition to the  hemiparesis. Investigation showed anaemia, and elevation of blood lead and ZPP. Her husband had obtained tablets and powders | chelation therapy: not further specified | her abdominal symptoms improved rapidly, but although her BLL fell to near-normal values there was only partial resolution of her neuropathy | BLL: 31 µg/dl (follow up) | 1.5 |
| 41 | Extensive investigation failed to reveal a cause for her symptoms until her husband told one of the medical staff that she had been taking a white powder for infertility; abdominal x-ray showed fine radiodense stippling within the bowel due to lead | chelation therapy: not further specified | successful medical treatment (symptoms resolved) | BLL: 37 µg/dl (follow up) | 1.79 |
| 42 | He had been seen 3 days earlier at another hospital where he was treated with laxatives for constipation. His symptoms had resolved only temporarily and returned the next day. He volunteered to his General Practitioner on this occasion that he had been taking powders and tablets for  impotence prescribed by a local Hakim. Lead poisoning was considered as a cause for his symptoms | no chelation therapy, discontinue medication, no further treatment (conservative therapy) | successful treatment, symptoms resolved over 3 days; however, blood lead remained elevated, and blood ZPP concentration increased from 170-304 µg/dl over the next 2 months, before both began to fall | BLL: 47 µg/dl (follow up) | 2.27 |
| 43 | was found to have an elevated BLL in a routine examination; follow-up examinations performed 3 and 6 months later showed increasing BLLs, respectively. Environmental investigation was conducted to find the possible sources of the elevated BLLs. There was no lead-related factory around her house. Neither family members nor neighbors had elevated BLLs. The building in which she lived was constructed in 1987. Lead content was not detected in the paints of house or in the pipe water or drinking water. Detailed history tracing revealed that the woman had been taking Chinese herbal medicines. | no chelation therapy, discontinue medication, no further treatment (conservative therapy) | successful treatment | BLL declined to 16,5 µg/dl 4 months later | 0.8 |
| 44 | was admitted to hospital on March 20,1995 for loss of appetite and general malaise of 1 month duration. Examination showed signs of anemia and mildly abnormal liver function. Increased basophilic stippling was found in blood smears. Lead poisoning was strongly suspected. | chelation therapy: D-penicillamine | successful medical treatment: mild anemia still persisted in May, but liver function returned to normal | BLL: 77 µg/dl in May (therapy started in March), 40 µg/dl in July | 1.93 |
| 45 | Husband of patient 2 -> also took the medication | N/A | N/A | N/A | N/A |
| 46 | N/A | chelation therapy: DMSA (p.o.) | successful medical treatment (recovered completely) | decreasing trend in PbB (not further specified) | N/A |
| 47 | N/A | chelation therapy: DMSA (p.o.) | successful medical treatment (recovered completely) | decreasing trend in PbB (not further specified) | N/A |
| 48 | N/A | chelation therapy: DMSA (p.o.) | successful medical treatment (recovered completely) | decreasing trend in PbB (not further specified) | N/A |
| 49 | N/A | chelation therapy: DMSA (p.o.) | successful medical treatment (recovered completely) | decreasing trend in PbB (not further specified) | N/A |
| 50 | Before admission, he had been hospitalized twice else- where. At his first presentation 2 months earlier, labora- tory examinations found elevated serum liver enzymes. He also had anemia. Superficial gastritis and bile reflux were found by endoscopy. An upper abdominal CT angiography showed “a general decrease in liver density; possible superior mesenteric artery syndrome”. A descriptive diagnosis of “chronic superficial gastritis, possible superior mesenteric artery syndrome, and abnormal liver function tests” was made. He was treated with omeprazole and sucralfate for 2 weeks which was accompanied by relief of his symptoms. He was discharged from the hospital without an identified etiology. Ten days after discharge, he was admitted to another hospital. Liver function tests, electroencephalogram and abdominal ultrasonography were normal. An incidental BLL test was performed (this hospital tests lead routinely) and reported as elevated. He was referred to our hospital for further evaluation and treatment.; No radiopaque point masses were identified on anteroposterior abdominal radiography; Anteroposterior radiograph of the knees showed increased linear radio-density at the distal femoral and proximal tibial and fibular metaphyses. | chelation therapy: DMSA (p.o.), CaNa2EDTA (i.v.); glutathione was added as an antioxidant | successful medical treatment, all the symptoms resolved | 21 µg/dl (after chelation therapy was completed) | 1.01 |
| 51 | N/A | chelation therapy: CaNa2EDTA (i.v.), DMSA (p.o.) | successful medical treatment (recovered completely) | decreasing trend in PbB (not further specified) | N/A |
| 52 | N/A | N/A | N/A | N/A | N/A |
| 53 | had consulted the same TCM practitioner as in case 1 and was prescribed the same nasal spray liquid to be used twice a day for 10 days.; Radiography of the abdomen revealed shadow of stool and gas as well as points of increased density; It could not be determined whether the radiopaque particles seen on the abdominal x-ray contained lead or not | chelation therapy: DMSA (p.o.), CaNa2EDTA (i.v.); folium sennae | successful medical treatment | 36 µg/dl (at the end of 5 days of chelation therapy) | 1.74 |
| 54 | the abdomen was tender, but there were no other physical signs, and no radiological evidence of intestinal obstruction; One week after admission, the patient's husband confided that she had been taking Deshi Dewa (traditional medicine), a white powder prescribed for infertility by a local traditional healer.; radiodensities of the bowel contents (Close inspection of the plain abdominal X rays taken on admission showed fine radio-dense stippling of the feces) | N/A | N/A | N/A | N/A |
| 55 | Fluorescent antinuclear antibody, Coombs, latex fixation, and stool guaiac tests were all negative. The abdomen was without hepatosplenomegaly or tenderness. Her affect was somewhat labile, with weepy and irritable spells. Examination of the cranial nerves and the sensory and motor examination were within normal limits. (…) -> many tests and clear hints, diagnosis took approx. 12 days | chelation therapy: CaNa2EDTA (i.v.) | patient noticed rapid and dramatic relief from her abdominal pains, myalgias, arthralgias, depression and irritability within the first 24h | by the second day of therapy, whole-blood lead concentration had fallen to 70 µg/dl and her 24h urinary lead excretion had risen to 8.216 µg/24h | 3.38 |
| 56 | N/A | no chelation therapy, discontinue medication; multivitamin capsules, green tea & garlic (conservative therapy) | successful medical treatment; The patient's constipation and anxiety were improved. After her BLL reached a normal level, follow-up was discontinued. | BLL: 18,05 µg/dl (~1 month later); it returned to the normal range (4.12 μg/dL) after 120 days | 0.2 |
| 57 | presented to the emergency department 3 times over 6 weeks with abdominal pain, constipation, nausea and vomiting; She was admitted to hospital to investigate the anemia and possible gastrointestinal bleeding. Results of esophagogastroduodenoscopy, colonoscopy and com- puted tomography and ultrasonography of her abdomen and pel- vis were normal. A bone marrow biopsy to investigate the anemia showed a moderate to marked increase in iron storage. Diagnostic laparoscopy for possible endometriosis showed no visible endo- metriosis. Biopsies of simple ovarian cysts were sent for patho- logic examination. The patient’s abdominal pain improved and her hemoglobin remained higher than 70 g/L after transfusions. Her discharge diagnosis was anemia of unknown cause and possible mild endometriosis. She was prescribed analgesics, laxatives and her usual medications, and was scheduled for follow-up with the internal medicine and hematology services. At the outpatient follow-up with the internal medicine service 2 weeks after discharge, the patient reported improved abdominal pain but ongoing fatigue, shortness of breath, headaches and tin- nitus. A urine sample was sent out for porphyria screening. At the second follow-up 4 weeks after discharge, at which point one of the authors became involved (H.A.), her porphyria screen showed ele- vated levels of coproporphyrin III and δ-aminolevulinic acid. Her biopsies did not show endometriosis. On further inquiry, the patient reported having taken Ayurvedic medications daily to treat infertility for more than a year. | chelation therapy: DMSA (p.o.) | successful medical treatment; The patient reported improving energy and no nausea, vomiting or abdominal pain. | Lead levels were 19.5 μg/dL shortly after chelation and 12.1 μg/dL 1 year later; Six months after chelation, her hemoglobin level was 119 g/L, with a mean corpuscular volume of 93.1 fL | 0.58 |
| 58 | plain abdominal and chest radiographs normal; abdominal ultrasonography howed a normal gallbladder and fatty infiltration of the liver; Abdominal computed tomography; gastroscopy and colonoscopy; the symptoms were not alleviated by intravenous histamine H2 blockers, antacids, analgesics, or non-steroidal antiinflammatory drugs; The heart sounds were normal, and the lungs were clear. There was severe tenderness of the right upper quadrant and epigastric region, with mild guarding but no rebound tenderness. Murphy’s sign was absent, peristalsis was slightly diminished, and there was no hematoma on the abdominal wall. No neurologic deficit was found. The rectal examination was normal.; The pancreas could not be clearly visualized because of gas in the intestines.; A barium evaluation of the upper gastrointestinal tract showed no abnormality of the esophagus, stomach, or small intestine. Abdominal computed tomography revealed slight distention of the colon, mild fatty infiltration of the liver, and no other abnormalities. The results of gastroscopy and colonoscopy were normal. Duplex ultrasonography of the superior mesenteric artery showed no blood-flow disturbances. The patient denied using barbiturates, anticonvulsants, or hormones, and he drank only socially. Urine, feces, blood concentrations of porphyrins and their precursors, and enzyme activity were measured and were not compatible with the presence of any known type of porphyria. Moreover, the finding that the concentration of aminolevulinic acid in urine was elevated by a factor of 41, while the urinary concentration of porphobilinogen was increased by a factor of 4, raised the possibility of heavy-metal (probably lead) poisoning. | chelation therapy: DMSA | successful medical treatment (patient became asymptomatic; 1 month later, liver function returned to normal, the hemoglobin concentration was 12,5 g/dl, the abnormalities in porphyrin metabolism were much less severe) | N/A | N/A |
| 59 | There was no indication of gastrointestinal bleeding by testing the stool for occult blood and by endoscopic investigation. The bone marrow biopsy showed a slight erythropoietic hyperplasia with siderosis of reticulum cells in an otherwise normal bone marrow. As the patient reported a febrile illness during his travels in south east Asia, we first assumed an infection as the cause of the anaemia. A diagnostic investigation for various infectious diseases showed hepatitis C infection with detection of anti-HCV-antibodies and of HCV-RNA in the serum. Otherwise there was no evidence of further viral, bacterial, or parasitic infections, including HIV, parvovirus B19, and malaria. A liver biopsy, obtained 8 weeks later, was consistent with a chronic hepatitis C of only marginal inflammatory activity, which could not explain the anaemia and the rather severe symptoms.  As no apparent cause of the anaemia could be established a new history was taken. The patient reported that during his stay in India he visited a traditional ayurvedic medical centre where he received herbal medicine and several bowel washings with oil. | chelation therapy: D-penicillamine | successful medical treatment: Urinary excretion of ∂-aminolaevulinic acid dropped after 4 weeks to the normal range and the haemoglobin increased to 119 g/l. As an interesting side effect, there was a stable reduction of the liver enzymes AST and y-GT to the normal range and of ALT to 34 U/l during chelation treatment. | BLL: 180 μg/l (after 4 months) | 0.87 |
| 60 | She was screened at a free lead-screening event sponsored by a nursing school community health promotion center. | no chelation therapy, discontinue medication, no further treatment (conservative therapy) | successful treatment | follow-up BLLs were 28 ug/dL in April, 21 ug/dL in May, 19 ug/dL in June, and 12 ug/dL in September 1997 (she stopped using the pills on April 1, 1997) | 0.58 |
| 61 | Extensive endoscopic and radiological investigation provided no explanation for the abdominal pain. Suspicion of lead intoxication was based on basophilic stippling in the red blood smear and raised excretion of porphobilinogen and delta-aminolevulinic acid in the urine sample. | chelation therapy: CaNa2EDTA (i.v.), DMSA (p.o.) | successful medical treatment | BLL: 0,2 mg/l (after 6 months) | 0.97 |
| 62 | The oral ulceration, which mainly affected the left lateral aspect of his tongue, appeared approximately five weeks prior to the onset of tics. Herpetic ulceration was diagnosed and confirmed by the isolation of herpes simplex virus (HSV) type 1 from his tongue swab. The lesion improved after treatment with a five day course of oral acyclovir (200 mg five times daily), but relapsed a few days after finishing the course of medication.; No skin rash or desquamation on the palms and soles were noted. There was a small healing ulcer at the tip of his tongue. His speech and gait were normal. Cardiovascular, respiratory, abdominal, and neurological examination did not reveal any abnormalities. Initial investigations including complete blood count, renal function tests and electrolytes, liver enzymes, immunoglobulins, complement, as well as urine analysis and toxicology screen were all normal. Electroencephalography, cranial computerised tomography, and magnetic resonance imaging were also normal. Serum antineuronal antibody as determined by flow cytometry (less than 5 MIF units) and ASOT (less than 60 Todd units) were not raised.; Sensory and motor nerve conduction velocities in our patient were normal. Detailed neuropsychological assessment was also normal.; On further questioning, our patient admitted that he had been using the CMH mouth spray. | no chelation therapy, discontinue medication, no further treatment (conservative therapy) | the patient was clinically stable and his neurological symptoms improving, chelating therapy was not considered to be necessary; his tics completely resolved at follow up four weeks later | blood mercury level returned to normal (4 weeks later) | / |
| 63 | He was referred to a hematologist for persistent anemia (hemoglobin: 9.2 g/dL) without basophilic stippling, refractory to iron therapy. Initial investigation revealed normal iron stores and normal hemoglobin electrophoresis. To complete the workup, a blood lead level was obtained. The mother who is well-educated and whose English is excellent, initially denied any exposure to lead or use of any folk medications. On further investigation, the mother had been giving S.P. a Tibetan Herbal Vitamin. | chelation therapy: DMSA, later EDTA & BAL, then again 6 chelations with DMSA | He has maintained his growth parameters and made developmental progress, achieving ambulation with minimum assistance and he understands simple directions but remains nonverbal. S.P.'s baseline developmental delay made the detection of the lead toxicity especially difficult. The effect of lead exposure during brain development has been well-documented. The impact on S.P. of this level of toxicity over 4 years, in the context of a prior neonatal asphyxia, is not quantifiable. Two months after chelation the patient was able to walk with minimal support and was more communicative. Within 6 months of stopping the herbal vitamin, and after chelation, his mother and grandmother felt that he made significant progress in social interactions characterized by more awareness and joy of others, and responsiveness to directions. | His lead level at the end of the chelation was 25.6 μg/dL. A 24-hour-urine collection (1245 mL) on day 3 of chelation revealed a lead level of 4480 μg/L or 5578 μg/24-hour (normally none detectable). | 1.18 |
| 64 | N/A; patient mentioned the herbal solution | discontinue medication; topical treatment with 10% salicylic acid; cryotherapy | At the follow-up examination 2 weeks later, a marked improvement was noticeable with only minor skin lesions left on the palms and less prominent lesions on both plantar areas. | N/A | / |
| 65 | N/A (the parents became aware of cases of lead poisoning caused by ethnic remedies -> parents have expressed suspicions to doctors); electromyography of the child’s legs showed moderate prevalently distal chronic axonal polyneuropathy | no chelation therapy, discontinue medication, no further treatment (conservative therapy) | successful medical treatment; In February 1997, the child’s right eye was enucleated. His general state of health, blood cell pattern, and electromyography findings gradually improved. | hair arsenic concentration: 0,06 mg/kg (1,5 years later) | / |
| 66 | X-rays of the chest and abdomen were all normal; laboratory tests; He was therefore managed with intravenous fluids, given pain relief and prescribed laxatives. His intestinal symptoms resolved very gradually. In view of his continuing abdominal pain, persistently elevated serum bilirubin concentrations and alanine transaminase activity, many other investigations were initiated: Synacthen test (normal), Autoantibody profile (negative), hapatitis viral serology (negative), liver biopsy (normal), abdominal ultrasound (normal); porphyrin screen -> positive -> test for lead | no chelation therapy, discontinue medication, no further treatment (conservative therapy) | patient became asymptomatic; his now normal liver function tests, with a marked decrease in blood lead (1 ,9 µmol/L) and ZPP (119 µmol/mol Hb), were entirely consistent with the diagnosis of lead toxicity due to herbal remedy poisoning | BLL: 1,9 µmol/l (3 months later) | 1.88 |
| 67 | Ultrasonography revealed a normal liver, and viral hepatitis markers including hepatitis A, B, and C were all negative.; laboratory & blood tests -> Lead poisoning was suspected | no chelation therapy, discontinue medication, no further treatment (conservative therapy) | successful medical treatment; the patient stopped taking the herbal pills and her liver function normalised within 3 weeks; haemoglobin level rose from 72 g/L to 106 g/L within 5 weeks, and was 124 g/L when measured 4 months later | BLL: 2,94 µmol/l (on discharge), 2,12 μmol/l (after 3 months), 1,66 μmol/l (4 months later) | 1.64 |
| 68 | She had consulted the same TCM practitioner as in case 1 | chelation therapy: Dimercaprol (i.m., developed facial flushing and chest tightness after the first dose!), CaNa2EDTA | successful medical treatment | 3,18 μmol/l (on discharge) | 3.19 |
| 69 | A hematologic ex- amination showed normochromic, normocytic anemia. Pe- ripheral blood smears showed basophilic stippling of eryth- rocytes (Picture 1). Since he worked in the metal processing industry, lead poisoning was suspected, but a further ques- tionnaire revealed that he had been taking Ayurvedic | chelation therapy: N/A | chelation therapy rapidly improved his symptoms | N/A | N/A |
| 70 | The use of herbal medicines was quickly detected.; Liver stiffness measurement (Shearwave Elastography, Logiq E9, GE, USA) was 28.8 kPa, and hepatic venous pressure gradient (HVPG) was 12 mm Hg. The baseline (at admission) and follow-up investigations are shown in Table 1. A transjugular liver biopsy showed features of macro- and microvesicular steatosis with lobular neutrophilic inf lammation, extensive ballooning of hepatocytes with MDB and pericellular fibrosis in the presence of lobular distortion due to the formation of irregular incomplete nodules of hepatocytes surrounded by thick fibrous bands at the portal areas with extension to peri-portal areas with enhanced and dilated portal venules. A diagnosis of severe AH was considered associated with incomplete cirrhosis. | discontinue medication, corticosteroid therapy | Six months after stopping herbal medicines, the repeat liver biopsy demonstrated resolution of alcohol-related changes but persistence of classical features of non-cirrhotic portal hypertension -> After 6 months of stopping the Ayurvedic medications, a repeat liver biopsy demonstrated resolution of AH but the persis- tence of classical features of ‘hepato-portal sclerosis’, a variant of NCPH (Non-cirrhotic portal hypertension); on follow-up, the thrombocytopenia, jaundice, ascites and coagulation failure resolved with the persistence of mild hypoalbuminemia; Repeat percutaneous liver biopsy revealed the persistence of dense fibrosis of the portal areas with portal vein sclerosis, segmental portal vein dilatation, peri-portal collateral formation, thin incomplete septae and vague nodularity in the absence of steatosis, inflammation or pericellular fibrosis | N/A | / |
| 71 | N/A | no chelation therapy, discontinue medication, no further treatment (conservative therapy) | There was a gradual but incomplete resolution of the paresthesia and distal weakness over the next several months. Hyperkeratosis and scaling of the limbs showed complete regression 3 months after the stoppage of arsenic. Neuropathy showed steady but incomplete improvement. She has currently regained complete strength in her upper limbs but there is still residual weakness and tingling in the lower limbs. Blood arsenic returned to acceptable levels 6 months after cessation of the drug. She has been now followed up for more than a year and is doing well. | N/A | / |
| 72 | She had consulted the same TCM practitioner as in case 1 and case 2 | chelation therapy: Dimercaprol (i.v.), CaNa2EDTA (i.v.) | successful medical treatment | 1,23 μmol/l (on discharge) | 1.21 |
| 73 | A year previously she had received a blood transfusion for anemia found during minor surgery for an ingrowing toenail. This anemia had not been investigated further.; Heart sounds, chest auscultation, neurological examination, chest x-ray, ultrasound (US) and computerized tomography (CT) scans of the abdomen were all normal; Hemoglobin electrophoresis showed a normal pattern. Sickling test, Coomb’s test, Ham test, antinuclear antibody (ANA), mycoplasma titer, Hepatitis (A, B, and C) serology were all negative as well as stool occult blood (SOB) | chelation therapy: D-penicillamine (p.o.) | successful medical treatment; abdominal pain weakened and her ALT, AST, alkaline phosphatase and bilirubin returned to normal | N/A | N/A |
| 74 | were identified during routine screening by California's Child Health and Disability Prevention (CHDP) Program | chelation therapy: not further specified | N/A (successful medical treatment) | N/A | N/A |
| 75 | were identified during routine screening by California's Child Health and Disability Prevention (CHDP) Program | chelation therapy: not further specified | N/A (successful medical treatment) | N/A | N/A |
| 76 | A 24-year-old pregnant woman, who had recently emigrated from India, was found to have a haemoglobin level of 70 g/L at 24 weeks’ gestation. At that time it was noted that she was a vegan and had a normal blood film and iron studies. At 30 weeks’ gestation, she presented with abdominal pain and a progressive confusional state culminating in seizures. The blood film now showed basophilic stippling, a typical sign of lead poisoning, and subsequent testing showed she had a blood lead concentration of 5.2 µmol/L; maternal encephalopathy; -> the diagnosis of intrauterine lead intoxication was made on the basis of a maternal encephalopathy with a high BLL, maternal anaemia with basophilic stippling, and a high cord-blood lead concentration | chelation therapy: Dimercaprol (i.m.), CaNa2EDTA (i.v.) | N/A | N/A | N/A |
| 77 | mother had lead poisoning while being pregnant -> the newborn was also diagnosed with lead poisoning; Radiographs of the long bones showed an increase in the bone density adjacent to the metaphyses; The infant was flaccid and areflexic and did not move in response to noxious stimuli, although spontaneous ocular movements were present. As she had emerging alveolar hypoventilation and no gag reflex, she was intubated and ventilated. The clinical suspicion of bilateral diaphragmatic palsy was later confirmed by fluoroscopy. | chelation therapy: Dimercaprol (i.m.), CaNa2EDTA (i.v.), DMSA (p.o.), again CaNa2EDTA (i.v.) | by Day 42, facial, bulbar, proximal-limb and diaphragmatic muscle activity had improved sufficiently to allow successful extubation; about 5,5 months after birth the infant was able to fully feed by sucking, although there was significant gastroesophageal reflux; her peripheral weakness had almost resolved, but bilateral wrist drop and poor head control persisted; brainstem auditory response showed right sensorineural deafness (6,5 months after birth); The infant was discharged home, on succimer, at five months’ corrected age with a blood lead concentration of 0.95 µmol/L. A neurodevelopmental examination at the time revealed a two-month delay. | BLL initially rose to 11.8 µmol/L within the first 48 hours of therapy, then fell rapidly over the next few days; urinary lead concentration after 7 days of therapy: 4,2 µmol/l; over the 3-week course of succimer, urinary lead concentrations fell further, while BLL remained relatively constant; BLL 5,5 months after birth: 1,8 µmol/l; BLL 7,5 months after birth: 0,95 µmol/l | 0.97 |
| 78 | history of slowly progressive muscular dystrophia (since she was 15 years old); In the course of the anemia diagnostics performed in advance, a gastroscopy, a colonoscopy, and a gynecological examination had already been performed without pathological findings; There was marked anemia of the skin and mucous membranes and kyphoscoliosis of the thoracic spine. The lungs were auscultatorily unremarkable with vesicular breath sound and sonorous tapping sound. Cardiac findings were normal, with pure, rhythmic heart sounds. The abdominal walls were soft and without tenderness or palpable resistance. Liver and spleen were not enlarged palpable, free renal bed. Neurologically, there was tetraparesis with absent muscle intrinsic reflexes and bilateral radial paresis. Advanced atrophy was seen in the muscles of the extremities. The patient required assistance with almost all physical movements and repositioning.; On initial presentation, the patient was initially treated with oral iron and vitamin B6 on the basis of an unclear iron deficiency anemia and a vitamin B6 deficiency. The patient did not show up at the scheduled follow-up appointment because she had been admitted to the hospital in a comatose state after a cerebral seizure. During the inpatient stay, the patient was diagnosed with exsiccosis and hypokalemia, which was corrected by appropriate infusion therapy. After the inpatient stay, the patient presented to us again for continuation of hematologic diagnosis. 2 months later, the significantly elevated lead level was detected. | chelation therapy: CaNa2EDTA (i.v.), DMPS (p.o.) | her neurological condition improved and the radial paresis resolved gradually so that she could return to work; her haematological parameters normalized | BLL: 320 µg/l (19 months after diagnosis) | 1.55 |
| 79 | Near the end of April 1999, a 42-yr-old female came to our medical facility to determine if she had arsenical skin lesions and to have hair, nail, and urine testing for arsenic. While watching a television program about arsenic, she noticed that her skin lesions resembled those of patients shown on the program. In early 1997, she observed spotted melanosis on her body though prior to that she thought she had merely been getting darker (diffuse melanosis). The clinical diagnosis was arsenic toxicity. (...) The woman presented with her husband and both were drinking hand-tube well water taken from a single source. Her husband had no skin lesions and his urine, hair, and nail analyses did not show elevated levels of arsenic. Samples of all water sources near her residence, including the hand-tube well near her work, had arsenic levels of less than 3µg/L (WHO acceptable arsenic level in drinking water is 10 µg/L). On further questioning, she related that she had been taking homeopathic medicines, including some containing arsenic.; Bone marrow aspiration, chest X-ray, renal ultrasound, and ECG were all normal. Fluorescein angiography confirmed ischemic maculae, the left greater than the right. | discontinue medication, N/A (no further treatment was mentioned) | Her hematological abnormalities improved gradually on discontinuation of the arsenic-containing medicines. | N/A | / |
| 80 | N/A; Routine hematology and biochemistry investigations, urinalysis, chest X-ray, and ECG were all normal. | discontinue medication, N/A (no further treatment was mentioned) | Although the arsenic concentration in his urine dropped 36 h after taking the last dose of medicine, it still remained higher than the normal, as did the arsenic levels in his hair and nails | N/A | / |
| 81 | N/A | discontinue medication; analgesics, vitamins and physical therapy | was discharged on June 16, 2000, after slow but gradual improvement in his pain and paraesthesias; On July 23, 2000, he still had global weakness of his limbs with inability to write or hold objects and continued to have difficulty in walking. His muscle power had improved slightly and some sensory symptoms persisted.; Urine analysis up to three months after he had consumed the arsenic preparation showed arsenic below toxic levels. A dermatology report described diffuse melanosis with some spotted melanosis on his soles. The patient reported that he had become completely black and his skin peeled off his palms and soles. | Hair, nail, and skin analyses continued to show elevated levels of arsenic | / |
| 82 | Hepatitis A, B, and C virus antigens and anti-bodies were all negative. Antinuclear antibodies, anti-Sm anti- bodies, anti-DNA antibodies, anti-cardiolipin antibodies, lupus anticoagulant, and anti- 2-glycoprotein I IgG were all negative. Both direct and indirect Coombs’ test results were negative. Osmotic fragility and glucose-6- phosphate dehydrogenase activity were appropriate to the retic- ulocyte count. Hemoglobin electrophoresis was normal. Sucrose lysis and Ham’s test were negative. The serum ceruloplasmin level and urine copper excretion were not elevated. However, numerous investigations failed to reveal a cause for the hemolysis. She was treated empirically with prednisolone 1 mg/kg per oral daily in three divided doses for 6 weeks, but there was no improvements in the hemolytic anemia. Subsequently, four sessions of plasma exchange were performed as a salvage ther- apeutic intervention, resulting in mild improvement in the hemolysis. We measured the levels of some heavy metals found in TCMs. | no chelation therapy, discontinue medication, plasma exchange, red cell exchange | successful treatment: Her hemolytic anemia improved gradually after therapeutic red cell exchange of 450 mL. After 1 month, her hemoglobin rose to 11.0 g/dL, and the reticulocyte count and urinary excretion of arsenic decreased to 2.2% and 13.0 g/ day, respectively. Her clinical course is described in Table 1. She is currently being followed monthly and there has been no deterioration in her condition. | urinary excretion of arsenic decreased to 13,0 µg/day (after 1 month) | / |
| 83 | N/A | chelation therapy: p.o. (not further specified) | successful medical treatment; 2 years later, the woman reported to her physician with joint symptoms from rheumatoid arthritis and was found to have microcytic anemia and a BLL of 64 μg/dL. She reported restarting ayurvedic medications 2 weeks previously. She agreed to stop taking the medications, and her physician decided against chelation therapy. | 1 week after completion, her BLL was 35 μg/dL | 1.69 |
| 84 | N/A | chelation therapy: p.o. (not further specified) | successful medical treatment; 1 spontaneous abortion (miscarriage) | BLL: 22 µg/dl (9,5 months after intial BLL testing) | 1.06 |
| 85 | N/A | chelation therapy: not further specified | successful medical treatment | BLL: 17 µg/dl (7,5 months after intial BLL test) | 0.82 |
| 86 | N/A | chelation therapy: multiple courses, not further specified; chronic anti-convulsant therapy | diagnosis: acute and chronic lead encephalopathy -> he improved after multiple courses of chelation and chronic anti-convulsant therapy, but had residual anoxic brain damage due to periods of status epilepticus | N/A | N/A |
| 87 | N/A | chelation therapy: not further specified | N/A | N/A | N/A |
| 88 | N/A | chelation therapy: not further specified | N/A | N/A | N/A |
| 89 | N/A | chelation therapy: not further specified | N/A | N/A | N/A |
| 90 | N/A | N/A | N/A | N/A | N/A |
| 91 | N/A | no chelation therapy, discontinue medication, no further treatment (conservative therapy) | N/A | N/A | N/A |
| 92 | N/A | chelation therapy: not further specified | N/A | N/A | N/A |
| 93 | N/A | chelation therapy: not further specified | N/A | N/A | N/A |
| 94 | N/A | chelation therapy: not further specified | N/A | N/A | N/A |
| 95 | Patient presented to her primary care pro- vider with fatigue, was diagnosed with thalassemia, and was referred to hematology/oncology. However, prior to the spe- cialist appointment, she felt more fatigued and presented to the emergency department. Her hemoglobin was 7.4 g/dL. She was transfused two units of packed red blood cells and remained hemodynamically stable during her admission. She did not need any blood transfusions except for a prior left knee replacement. She was discharged and later seen by hematology/oncology. At the hematology appointment, addi- tional laboratory tests were done, including a blood smear, low-density lipoprotein, haptoglobin, ferritin, hemoglobin electrophoresis, and hepatitis panel. The peripheral smear showed basophilic stippling, and lead levels were significantly elevated at 76 mg/dL. The patient was unsure of lead expo- sure. Since she was relatively asymptomatic, she was moni- tored for several months.  At follow-up, she was questioned further and indicated that the source of the lead was ayurvedic powder sourced from her trips to India. | chelation therapy: DMSA | successful medical treatment | A few months later, lead levels were 38,6 µg/dL.; At the next follow-up, she had an allergic reaction that resolved with antihistamines. Succimer therapy was discontinued due to lead levels decreasing below 20 µg/dL. | <0.97 |
| 96 | gastroscopy; abdominal sonography; chest X-ray and pelvic radiography; X-ray of the lumbar spine; ECG; bone marrow histology; 1 month after symptom manifestation, a therapy with proton pump inhibitors had been started after gastroscopic diagnosis of third-degree reflux esophagitis, which, however, had produced only a slight symptom improvement. Chronic polyarthritis had been treated with 15 mg of methotrexate weekly and 5 mg of prednisolone daily for 1.5 years and was currently asymptomatic. X-ray examinations of the chest and pelvis were unremarkable; Subsequently, assuming autoimmune hemolytic anemia in the context of rheumatoid arthritis, 100 mg prednisolone was administered orally daily. However, even this did not result in a significant increase in hemoglobin. | chelation therapy: DMPS (p.o., after 14 days the patient developed maculopapular exanthema on the face and in the trunk and arm areas!), D-penicillamine | successful medical treatment: complete cessation of abdominal discomfort, normalization of retention values and haptoglobin, and stabilization of hemoglobin at 11.4 g/dL | BLL: 559 µg/l (after 14 days of therapy) | 2.7 |
| 97 | On May 28, 2004, he got admissioned at department of pediatrics of our hospital. Then, he underwent gastroduo- denoscopy, colonoscopy, RBC-scanning and abdominal CT scan. All these examinations were normal. On small bowel series, no specific findings were observed except for the delayed intestinal motility. He was once discharged at his mother's request. At outpatient follow-up, he presented with aggravated anemia, and confessed that he got administered herb medicinal pills. On June 14, 2004, he got hospitalized for further investigation and treatment.; These patients presented anemia and severe coliky abdominal pain, but no neurologic symptoms. For this reason, they were initially misdiagnosed with gastrointestinal hemorrhagic disease.; These children came nearly being misdiagnosed with hemorrhagic enteritis.; However, we got a clue from their past history of herb medicinal pills; Both intestinal parasitology and occult blood test were negative.; No specific findings were seen on chest X-ray. Abnormal shadow was not seen in the epiphysis of long bones.; paralytic ileus was seen on abdominal X-ray | chelation therapy: CaNa2EDTA, DMSA (p.o.) | successful medical treatment | BLL: 24,8 µg/dl (2 weeks later), 45,3 µg/dl (7 weeks later, DMSA was re-administered), 38,78 µg/dl (5 months later) | 1.2 |
| 98 | no edema was found systemically; cardiac and respiratory sound was normal on chest auscultation; no abdominal distension was found; overall tenderness was present with no rebound tenderness; hepatosplenomegaly or palpable mass was not found; bowel sound was decreased; and no neurological deficits were noted. Both intestinal parasitology and occult blood test were negative. No specific findings were seen on chest X-ray. However, paralytic ileus was seen on ab- dominal X-ray. Abnormal shadow was not seen in the epiphysis of long bones. Based on the past history of herb medicinal pills, he was also strongly suspected of having lead poisoning. -> Bleivergiftung wie sein Bruder -> These patients presented anemia and severe coliky abdominal pain, but no neurologic symptoms. For this reason, they were initially misdiagnosed with gastrointestinal hemorrhagic disease.; These children came nearly being misdiagnosed with hemorrhagic enteritis | chelation therapy: CaNa2EDTA, DMSA (p.o.) | successful medical treatment | BLL: 42,6 µg/dl (2 weeks later), 67,9 µg/dl (7 weeks later, DMSA was re-administered), 37,45 µg/dl (6 months later) | 1.81 |
| 99 | electrocardiogram normal; thoracic radiograph normal; colonoscopy was planned, but patient did not appear at the appointment; -> upon inquiry, it turned out that in addition to the medication already mentioned, patient had also been taking Ayurvedic medication | chelation therapy: DMSA (p.o.) | successful medical treatment; Chelation therapy lowered the lead concentration and the symptoms and anemia disappeared. The patient's motor functions also improved. No complications of the treatment occurred. | N/A | N/A |
| 100 | N/A; patient mentioned the ayurvedic medicine; brain lesions (exact description of the extend of the brain lesions: see article) | chelation therapy: Dimercaprol | successful medical treatment (lesions resolved after chelation therapy -> lesions diminished and disappeared on posttreatment MR imaging; 6 months later, the patient is doing well clinically) | BLL: 38,4 µg/dl (postchelation therapy) | 1.86 |
| 101 | ultrasound examination of the adomen; radiography of the abdomen; colonoscopy; (…) The clinical examination was still normal, but in view of the work-up already carried out, the surgeon consulted carried out a laparotomy which found no abnormality; an appendectomy was performed; histological examination of the operative specimen showed no appendicitis; (...) The patient was admitted again to the same department as for the previous episode: abdominal CT scan found no abnormality; exploratory laparoscopy showed only dilatation of the small intestine; urinary cytobacteriological examination was normal. A new exploratory laparotomy was proposed; the patient refused it and returned home; (...) A porphyrin metabolism disorder was suggested; the analyst indicated that this profile was strongly suggestive of lead poisoning | chelation therapy: DMSA (p.o.) | successful medical treatment | BLL: 224 µg/l (2 weeks after the last intake) | 1.08 |
| 102 | plain radiograph of the abdomen revealed dilated small intestine and transverse colon with a few air fluid levels; Lead intoxication was suspected based on symptoms and preliminary blood findings; A contrast enhanced computerized tomography revealed diffused bowel dilatation and no area of narrowing of the intestine. The barium meal follow through examination did not reveal any narrowing or the stricture of the small intestine. He was thus diagnosed to have lead associated intestinal pseudo-obstruction | chelation therapy: DMSA | successful medical treatment; By day 4 of the therapy, the abdominal pain had improved. By day 6, he started moving bowel and the air fluid level present in the intestine disappeared. Clinically, autonomic symptoms such as restlessness, sweating, tachycardia, and postural hypotension had improved by day 5.; after 1 year: most of the autonomic parameters had normalized except for the persistence of mild loss of parasympathetic reactivity (gradual improvement in autonomic functions) | BLL: 56 µg/dl (6 weeks later), 40 µg/dl (1 year later) | 1.93 |
| 103 | N/A; An ultrasound scan showed mild hepatomegaly and endoscopy indicated antral gastritis. While she was being treated for antral gastritis, she was referred to our center for the evaluation of lead poisoning; patient was referred to the ‘‘National Referral Cen- ter for Lead Poisoning in India’’ (NRCLPI) of our medical college for the evaluation of presumptive lead poisoning | chelation therapy: D-penicillamine | successful medical treatment; blood lead and ZPP levels were found to be greatly decreased; Due to the in- creased blood lead levels during the equilibration period, the patient had to undergo repeated courses of chelation therapy and is on follow-up presently. | N/A | N/A |
| 104 | An abdominal computerized tomography scan and gastroscopy were performed and were both normal. Her vitamin B12, folic acid, rheumatoid and antinuclear factor levels were within normal range. At this time, a thick blood smear that was sent for evaluation in order to rule out malaria revealed basophilic stippling. | chelation therapy: DMSA (p.o.) | successful medical treatment: brought about prompt amelioration of the clinical symptoms and normalization of lead levels after a few weeks | BLL: 35 µg/dl (2 weeks after end of treatment) | 1.69 |
| 105 | Plain X-ray of abdomen revealed scattered radio opaque speckles and abdomen loaded with stools; The baby passed stools only after glycerine suppository; examination revealed mild distension of the abdomen; Stool analysis did not show any ova or cyst; plain X-ray of abdomen revealed scattered radio opaque speckles and abdomen loaded with stools | chelation therapy: CaNa2EDTA (i.v.) | successful medical treatment | BLL: 53 µg/dl (after therapy), 49 µg/dl (after 1 month) | 2.37 |
| 106 | At the time of presentation, her only medication was a Chinese herbal preparation, which she had been taking since her diagnosis of AML.; Results of a neurologic examination were nonfocal. Results of an electrocardiogram were normal. | discontinue medication, no further treatment (conservative therapy) | She has since discontinued use of this medicine, and her skin and nail findings have nearly resolved. | N/A | / |
| 107 | No explanation for the anaemia or ab- dominal pain was found after extensive clinical, endo- scopic and radiological investigation. Blood smear showed prominent basophilic stippling of erythrocytes (figure 1a). Bone marrow aspirate and trephine biopsy demonstrated a marked erythropoiesis with basophilic stippling (figure 1b), 10% ring sideroblasts (figure 1c) and dyserythropoiesis (figure 1d). These features, together with the abdominal cramps, were indicative of a lead intoxication. Upon further questioning the patient admitted to take some Ayurvedic medications. | chelation therapy: CaNa2EDTA (i.v.) | successful medical treatment; her haematological parameters, controlled after four months, were normalised (Hb 12.5 g/dL) | BLL: 40 µg/dl (after 1 week), 23 µg/dl (after 4 months); Hb 12,5 g/dl (after 4 months) | 1.11 |
| 108 | She mentioned taking a clay cure for internal purification since 3 weeks. No abnormalities were found after endoscopic and radio- logical investigations. Bone marrow aspirate and tre- phine biopsy showed a marked dyserythropoiesis. Be- cause of the striking basophilic stippling, her blood lead level was determined and showed to be elevated. | chelation therapy: DMSA (p.o.) | successful medical treatment | BLL: 19 µg/dl (after therapy), 3,6 µg/dl (after 6 months); Hb: 11,6 g/dl (after therapy), 13 g/dl (after 6 months) | 0.17 |
| 109 | History revealed his habitual consumption of herbal medicines-for any kind of illness. All possible investigations including HbSAg & HIV were carried out. An abdominal CT scan revealed the presence of a 3mm calculus on the left kidney. Repeat CT scan confirmed the presence of left renal calculus. Endoscopy was normal. A barium meal & follow through study did not reveal any abnormality in the small bowel. Test for malaria along with ICT, WIDAL, tests for Leptospira, Brucella, & dengue were carried out. All tests were negative. Emperic Falcigo (antimalarial) and ceftriaxone were started. A surgical opinion was sought because of the persistent abdominal pain and fever. During this time as a work up for anemia, a rectic count was done which was raised and suspected a possibility of hemolytic anemia; the entire work up for hemolytic anemia were found to be negative. As the fever was not coming down, the smear for malaria was repeated including the indirect Coomb’s test that was positive this time and Chloroquine was given. It was also noted that the W.B.C count was progressively coming down and showed leucopenia on the blood picture and a possibility of drug-induced cause was found out. Chloroquine was stopped and Piperacillin-Tazobactum was started. A bone marrow examination revealed Microcytic Blood picture. Diagnosis was made as a case of Vivax Malaria with Microcytic hypochromic anemia with renal stone. For renal stones treatment was advised at a later stage and in view of the persistent abdominal pain, doctors suspected lead Poisoning and the patient was sent for Blood Lead analysis to the NRCLPI at Bangalore. | chelation therapy: D-penicillamine; advised to take a diet rich in calcium, iron and zinc and to drink more water & other fluids | successful medical treatment: disappearance of abdominal pain, decreased blood lead level and an increase in Hemoglobin level | BLL: 32,1 µg/dl (after 1 month); 23 µg/dl (2 months after chelation therapy) | 1.11 |
| 110 | She was diagnosed with portal hypertension with ascites and was referred to our institute for further evaluation. Her Ayurvedic medications were stopped after this acute episode.; Electroencephalogram (EEG) showed cerebral dysrrhythmias. Screening tests for inborn errors of carbohydrate, lipid, and amino acid metabolism did not show any abnormality. The serum ceruloplasmin level was normal. Tests for antinuclear antibody and anti-liver–kidney microsomal antibody 1 (anti-LKM1) were negative. The α1-antitrypsin levels and phenotype were normal. The serum arsenic level was 202.20 μg/L (normal level, < 60 μg/ L). X-Ray of the chest was normal. Markers for viral hepatitis were negative. Ultrasonography and computed tomography (CT) scan of the abdomen revealed gross ascites, a nodular liver surface, and an atrophied right lobe of the liver, with compensatory hypertrophy of the left and caudate lobe. Upper gastrointestinal tract endoscopy showed pyloric varices. Liver biopsy showed spotty necrosis and focal perisinusoidal fibrosis, suggestive of noncirrhotic portal fibrosis.; a skin biopsy from the sole showed marked lamellar hyperkeratosis and a sparse perivascular infiltrate of lymphocytes and histiocytes in the dermis => cutaneous & hepatic manifestations | no chelation therapy, discontinue medication, topical keratolytic agent | successful medical treatment: after 6 months, there was significant flattening of the keratotic papules, and the pigmentation also improved to the patient’s satisfaction | N/A | N/A |
| 111 | Lead poisoning in this infant was discovered serendipitously during a well child care visit, as a result of the policy of universal lead screening of infants annually in Massachusetts.; An asymptomatic 12 month old infant of Thai parents was discovered to have a blood lead concentration of 61 mcg/dL; Investigation of the family’s apartment found no lead haz- ards. Neither parent had an occupational exposure or worked with lead in hobbies. They had no imported cookware or spices, but had spent the previous two months in Bangkok, Thailand, visiting relatives. Upon further questioning, mother revealed that she used a Chinese patent remedy Khao Gui in tablespoon amounts for treatment of fever as needed.; long bone radiographs showed metaphyseal sclerosis consistent with chronic lead poisoning; He already manifested the bone changes at the metaphyses usually associated with a long duration of exposure to a very high blood concentration of lead | chelation therapy: CaNa2EDTA (i.v.), DMSA (p.o.) | successful medical treatment | BLL: 33 µg/dl (after first cheation therapy), 23 µg/dl (after second chelation therapy) | 1.11 |
| 112 | was referred by his primary care physician to a nephrology clinic for evalua- tion of elevated serum creatinine levels; CKD was initially thought to be secondary to hyper- tension and diabetes, but 6 months later (because the absence of albuminuria was inconsistent with diabetic nephropathy and the patient had lived in south asia, where lead contamination might not be very tightly monitored), whole-blood lead level was measured and found to be 1.89 μmol/l; at the initial nephrology consultation, the patient’s serum creatinine level was elevated at 118.4 μmol/l. the elevation was originally attributed to chronic kidney disease secondary to hypertension or diabetes; however, the absence of proteinuria prompted measurement of his blood and body lead level 6 months later. Investigations revealed that the patient had been taking a lead-containing ayurvedic herbal remedy for the preceding 2 months. Following discontinuation of the remedy and infusion of calcium disodium ethylenediaminetetraacetic acid to eliminate the accumulated lead, decreases were observed in the patient’s blood and body lead levels and serum creatinine level.; as the patient’s serum creatinine level had been increasing before his known exposure to lead, he either had chronic lead nephropathy as a result of prior undocumented lead exposure, or was experiencing lead-induced progression of CKD; ultrasonography revealed two kidneys of normal echogenicity and size | chelation therapy: CaNa2EDTA (i.v.) | successful medical treatment; his serum creatinine value at final follow-up 16 months after the initial nephrology consultation was 91.9 μmol/l | BLL: 0,17 μmol/l (7 months after the final chelation session) | 0.19 |
| 113 | She was discharged with a diagnosis of gastroenteritis.; The patient presented twice during the next 5 days with similar and then worsening complaints and no relief with Tylenol 3 (McNeil-PPC, Inc., Fort Washington, PA). Diagnoses considered but deemed unlikely included abruption, chorioamnionitis, round-ligament pain and pain due to adhesions/scar tissue, and constipation. An amniocentesis showed no chorioamnionitis. She had negative tests for hepatitis A, B, and C. She had no evidence of antiphospholipid antibody syndrome. During this time, she also had a normal right upper quadrant ultrasound examination and an abdominal computed tomography examination. Persistent abdominal pain led to testing for porphyria, which revealed a negative urine porphobilinogen. Surgical and gastrointestinal consultations were noncontributory. During the hospitalization, she ad- mitted to using several “herbal” preparations that she had obtained from India. Persistent abdominal pain led to testing for porphyria, which revealed a negative urine porphobilinogen. How- ever, a 24-hour urine collection revealed a markedly elevated aminolevulinic acid (ALA) level (87.0 mg/24 hours; normal range less than 6.4). The differential diagnosis for the combination of an elevated ALA level in the absence of urinary porphobilinogens includes the exceedingly rare ALA dehydratase porphyria or lead poisoning. The health supplements were now thought to be the source of the lead poisoning. | chelation therapy: DMSA (p.o.) | successful medical treatment | BLL: 35 µg/dl (at birth of her son, after first chelation therapy) | 1.69 |
| 114 | mother had lead poisoning while being pregnant -> the newborn has been diagnosed with lead poisoning as well | chelation therapy: DMSA (p.o.) | successful medical treatment, normal developmental milestones | N/A | N/A |
| 115 | About 3 months prior to coming to us, he had started taking medication from an Ayurvedic practitioner, viz., 5 pellets a day for generalized weakness. At the end of about 2 months of this medication, he started having numbness and tingling sensations in hands and feet and also developed fatigue. For these complaints, he was seen in another hospital but as they could not reach a diagnosis, he was referred to us. Magnetic resonance imaging of brain and cervical spine gave normal results. Heavy metal concentration in blood was tested | chelation therapy: D-penicillamine | successful medical treatment; at the end of 8 weeks therapy, he had improved symptomatically by >50% and nerve conduction also showed mild improvement; he has been followed up for 4 months after completion of treatment and is doing well | BLL: 8,0 µg/dl (after treatment); urinary lead level: 5,6 µg/dl (after treatment) | 0.39 |
| 116 | Cardiac, respiratory and liver functions were within normal limits. Neurological assessment revealed no abnormality. He was treated symptomatically and his condition improved as the symptoms got relieved within a few days. Taking serious note of the poor response to treatment, the case was reviewed. On further questioning the patient came out with the history of having been treated for primary infertility by a quack in his native place with an unnamed preparation. | chelation therapy: D-penicillamine (p.o.) | successful medical treatment: his general condition improved, he continued to remain asymptomatic | BLL: 60 µg/dl (after 10 weeks of therapy), 55 µg/dl (after 16 weeks of therapy), 25 µg/dl (after 6 months of treatment) | 1.21 |
| 117 | detailed patient history revealed that the patient had been taking herbal supplements | no chelation therapy, discontinue medication; supplementation of iron, zinc and calcium (conservative therapy) | successful treatment: his symptoms resolved after six months of diet modification and essential mineral supplementation | BLL: 15 µg/dl (after 6 months); after one month it was reduced to 59.43 μg/dl, and further reduced to 54.3 μg/dl after three months | 0.73 |
| 118 | He was a known case of cholelithiasis for which he was under surgical follow-up and psoriasis for which he was on Ayurvedic medication for the past six months with excellent response. There were multiple healed psoriatic plaques over abdomen and lower limbs. Examination of abdomen and other systems did not reveal any abnormality. Ultrasonography (USG) abdomen revealed chronic calculus cholecystitis. Hence he was planned for elective cholecystectomy. In view of the anaemia, increased polychromasia, raised bilirubin and transaminases, the patient was further evaluated. Detailed liver function tests, upper gastro intestinal (UGI) endoscopy, hepatitis viral markers, isotope study for occult gastrointestinal (GI) haemorrhage and colonoscopy did not reveal any abnormality. The cause of anaemia and bilirubinemia remained obscure. (...) Basophilic stippling in the RBCs raised doubt of lead poisoning, more so as the patient was on Ayurvedic medication for psoriasis. | chelation therapy: CaNa2EDTA (i.v.) | successful medical treatment; Patient has recovered now and is asymptomatic. He has stopped the Ayurvedic medication totally. His hemogram and liver function tests (LFT) are normal. However, his psoriatic lesions have drastically increased. He is on regular followup. | BLL: 38 µg/dl (after 3 months of treatment) | 1.84 |
| 119 | Chest and abdominal X-ray were normal, and bone density test showed osteopenia. Renal ultrasonography showed increased bilateral kidney diameters of 11.6 cm and 10.9 cm; a renal biopsy showed degeneration of the proximal tubules with normal glomeruli; renal ultrasonography showed increased bilateral kidney diameters | N/A | N/A | N/A | / |
| 120 | N/A; patient was admitted to a hospital in Bangalore twice within a period of 10 days for the treatment of generalized weakness, vomiting and abdominal pain. Both times the treatment was unsuccess- ful. The patient’s history revealed that he had been con- suming 12 different Ayurvedic medicines for the past 4 years. The doctors suspected lead poisoning. | chelation therapy: D-penicillamine; advised to take calcium & B-complex and consume large quantity of liquids | successful medical treatment; abdominal pain was relieved following 2 months of chelation therapy | BLL: 48 µg/dl (after 2 months, end of chelation therapy), 46,5 µg/dl (15 days later), 27,4 µg/dl (after 1 year) | 1.32 |
| 121 | Doctors noticed a decrease in Hemoglobin level (8 g/dl) and suspected some internal bleeding. All the routine tests including tests for HIV and cancer were negative. Endos- copy, colonoscopy and barium meal follow through were carried out and internal bleeding was ruled out. The patient mentioned that he contacted a local Vaidya in Pune, Maharashtra for the treatment of psoriasis. The peripheral blood picture showed basophilic stippling, after consuming the product for 1 month. Doctors suspected lead poisoning. | chelation therapy: CaNa2EDTA (i.v.); course of calcium and multivitamin tablets | successful medical treatment; Hb 9,7 g/dl (after third cycle) | BLL: 88,4 µg/dl (after 12 days), 85,8 µg/dl (5 days after second cycle), 56,4 µg/dl (20 days after third cycle), 21,1 µg/dl (after 6 months) | 1.02 |
| 122 | He consulted a neurologist in Bangalore who diagnosed peripheral neuropathy. The patient had a history of consuming Ayurvedic medicines leading the neurologist to suspect lead poisoning. | no chelation therapy, discontinue medication; advised to take calcium and B-complex tablets, drink large quantity of fluids | successful medical treatment | BLL: 18,2 µg/dl (after 6 months) | 0.88 |
| 123 | N/A; her patellar bone lead was extremely high, peaking at 84 μg/g (expected upper limit for her age, 30 μg/g); her high tibial lead was also moderately elevated, peaking at 26 μg/g (expected upper limit for her age, 20 μg/g). | chelation therapy: DMSA; calcium supplementation (p.o.) | successful medical treatment | about 2.5 years from her first visit, she was given our advise to stop contraception and conceive; we followed her BLL and FEP during pregnancy as well; at 5th month of gestation she had BLL-9 μg/dl and FEP-52 μg/dl; there was mild decrease at 8th month of gestation with BLL-8 μg/dl and FEP-32 μg/dl | 0.39 |
| 124 | N/A; The patient was referred for toxicological review. Further questioning revealed he had used three Ayurvedic medicines | chelation therapy: DMSA (p.o.) | successful medical treatment | his blood lead concentration fell rapidly, with a moderate rebound 6 weeks after the completion of chelation therapy | N/A |
| 125 | He had undergone appendect- omy that revealed a normal appendix and then an exploratory laparotomy examination that revealed a normal intestine with mild mesenteric lymph nodes inflammation; abdominal X-ray was ordered at admission and showed significant abdominal distension and two radiopaque foreign bodies in the caecal and rectal region, respectively | chelation therapy: CaNa2EDTA (i.v.) | successful medical treatment: Resolution of clinical manifestations and a complete normalization of haematological and toxicological indices followed. | N/A | N/A |
| 126 | A recent colonoscopy was unremarkable. A CT scan of the abdomen and pelvis showed no specific abnormalities. The patient was discharged to home with anti-emetics and instructions to follow up with her pri- mary care physician. Five days later, the patient returned to the emergency department. The patient was admitted and subsequently underwent extensive eva- luation for gastrointestinal abnormalities including eso- phagogastroduodenoscopy and colonoscopy, both of which were unremarkable. Review of the peripheral blood smear demonstrated ... -> The clinical picture was consistent with lead poisoning. Upon further ques- tioning, the patient disclosed that she had been taking an Indian Ayurvedic medicine | chelation therapy: DMSA (p.o.) | successful medical treatment: patient’s symptoms resolved and laboratory abnormalities normalized | BLL: 46 μg/dL (at the end of chelation therapy) | 2.22 |
| 127 | N/A | no chelation therapy, discontinue medication, no further information | N/A | N/A | N/A |
| 128 | N/A | no chelation therapy, discontinue medication, no further information | N/A | N/A | N/A |
| 129 | An abdominal X-ray confirmed stool retention without perforation or bowel obstruction. (...) Haptoglobin was undetectable and the Coombs test was negative. The diagnosis of a Coombs negative haemolytic anaemia was made. Despite further investiga- tions, its origin remained unclear: infections, drugs, sickle cells or other haemoglobinopathies, glucose-6-phosphate dehydrogenase or pyruvate kinase deficiency and microangiopathic haemolytic anaemia were ruled out. Because of the abdominal colics, porphyria testing was performed. Increased concentrations of coproporphyrine III, delta-aminolevulinic-acid (urine) and elevated free- and zinc-protoporphyrin (erythrocytes) were observed. These results were not consistent with classical porphyrias because, instead of a single enzyme, a number of enzymes were inhibited. As heavy metals are known to inhibit enzymes such as ALA dehydratase, coproporphyrinogen oxidase and ferrochelatase [1], a blood lead concentration was obtained | chelation therapy: DMSA (p.o.) | successful medical treatment: After 3 weeks, the blood lead concentration had decreased to 17 µg/dl; anaemia and red blood cell morphology had normalized and the patient had become asymptomatic. | successful medical treatment | 0.82 |
| 130 | His renal and liver function, electrolytes, gastroscopy, colonoscopy, and computed tomography were all unremarkable. A review of his medical history found that for the past 3 months he had been taking a traditional Chinese herbal medicine | chelation therapy: CaNa2EDTA (i.v.) | successful medical treatment: His abdominal pain, blood lead concentration, and microcytic anemia resolved following the 3-week course of chelation treatment. | N/A | N/A |
| 131 | had consulted the same TCM practi- tioner as in Cases 1 and 2 and was prescribed the same medication to be used twice a day for 7 days.; No abnormality was found on abdominal x-ray. On examination, his liver was palpable 4 cm below the right costal margin. | chelation therapy: DMSA, CaNa2EDTA (i.v.); but first: medication to improve liver function (glutathione & disodium glycyrrhetate) | successful medical treatment | 34 µg/dl (post-chelation) | 1.64 |
| 132 | Respiratory and cardiovascular system examinations were normal. Abdomen examination showed moderate tenderness in the left lower quadrant with no mass palpable and no flapping tremors.; The patient was admitted to the nephrology department since he had metabolic disturbances and AKI. He was taken for emergency hemodialysis via a left double lumen femoral catheter. He started improving clinically after one session of dialysis and other supportive care. His renal function rapidly improved and his serum creatinine was 2.17 mg/dl on the fifth day of admission. The femoral catheter was then removed and he was discharged on day six. On follow up his serum creatinine was within normal limits (1.2 mg/dl). The ayurvedic supplement he had been taking was sent for toxicological analysis  His electrocardiogram (ECG) showed sinus bradycardia (Figure 1). Non-enhanced computed tomography (CT) scan of the abdomen showed no obvious renal or ureteric calculi, nor any pelvicalyceal/ureteric dilatation on either side. He had minimal ascites and bilateral basal pleural effusion. | no cheation therapy; emergency hemodialysis, other supportive care | renal function improved after only one cycle of dialysis | BLL: N/A; on follow up his serum creatinine was within normal limits (1,2 mg/dl) | N/A |
| 133 | He underwent extensive investigations, including computed tomography of his abdomen and pelvis as well as upper and lower gastrointestinal endoscopy, all of which failed to identify a cause for his abdominal symptoms | chelation therapy: DMSA | successful medical treatment; patient improved symptomatically and his anaemia resolved within two months of discontinuing the ayuverdic remedies | BLL dropped from a pretreatment level of 782 μg/L to 287 μg/L (after therapy) | 1.39 |
| 134 | Test results for hepatitis-associat- ed antigen, antinuclear antibody and a serum Venereal Disease Research Laboratory test (VDRL) were all negative. IgG and IgM antibodies to Herpes simplex, Mycoplasma pneumonia and human T-cell lymphoma virus type I and II were negative. Polymerase chain reaction for tuberculosis and Herpes simplex virus type I and type II produced normal results.; Electromyography; initially misdiagnosed as Guillain-Barré Syndrome; patient showed mild neuropathy in the absence of any systemic manifestation of arsenic intoxication | no chelation therapy, discontinue medication; i.v. immunoglobulin (Therapie der Wahl beim Guillain-Barre-Syndrom) | he responded with a clear improvement in limb strength | N/A | N/A |
| 135 | was taken to the local children's hospital several times by his parent but was not cured; X- ray of the chest and abdomen showed no specific findings. The doctor in charge then planned a colonoscopy procedure for further diagnosis. However, before performing the colonoscopy, the doctor recommended a blood lead level test | chelation therapy: CaNa2EDTA (i.v.) | successful medical treatment, disappearance of his abdominal symptoms | BLL: 118 µg/l (after treatment); the BLLs of both the brother and the sister reduced steadily; the brother's BLLs reduced relatively slowly whereas the sister's reduced relatively rapidly, a consequence of a longer lead exposure period for the brother | 0.57 |
| 136 | BLL wurde getestet, weil der Bruder einen zu hohen BLL hatte | chelation therapy: CaNa2EDTA (i.v.) | successful medical treatment | BLL: 88 µg/l (after treatment); the BLLs of both the brother and the sister reduced steadily; the brother's BLLs reduced relatively slowly whereas the sister's reduced relatively rapidly, a consequence of a longer lead exposure period for the brother | 0.43 |
| 137 | N/A | chelation therapy: CaNa2EDTA | successful medical treatment | BLL: 36 µg/dl (5 days after chelation), 20 µg/dl (3 months later, 2 weeks after delivering); her newborn's BLL was 23 µg/dl at 3 days after birth | 0.97 |
| 138 | N/A | N/A | BLL decreased | BLL: 10 µg/dl (2 months later), 1 µg/dl (another 4 months later, 3 months before delivery); newborn's BLL was not measured | 0.05 |
| 139 | N/A | N/A | BLL decreased | BLL: 26 µg/dl (7 weeks after discontinuing use); newborn's BLL was 7 µg/dl at birth | 1.26 |
| 140 | abdominal computed tomography scan and gastrointestinal endoscopy with negative assessments; blood analysis -> It was suggested that there may have been an accumulation of heavy metal in her body | chelation therapy: not further specified | successful medical treatment: symptoms improved | N/A | N/A |
| 141 | laboratory tests; Radiological evaluation revealed mild hepatomegaly. Due to history of prolonged ayurvedic medicine usage, he was subjected to heavy metal screening; skeletal survey did not reveal dense metaphyseal lines | chelation therapy: CaNa2EDTA (i.v.) | successful medical treatment, reduction in BLL and clinical improvement | N/A | N/A |
| 142 | N/A | N/A | N/A; 1 miscarriage (causal connection not proven) | N/A | N/A |
| 143 | N/A | N/A | N/A; 1 miscarriage (causal connection not proven) | N/A | N/A |
| 144 | N/A | no chelation therapy, discontinue medication, no further treatment (conservative therapy) | BLL decreased | BLL: 11 µg/dl (5 weeks after discontinuing the medications) | 0.53 |
| 145 | Abdominal computed tomography, upper gastrointestinal endoscopy, and colonos- copy results were unremarkable.; On further inquiry, the patient re- ported travel to India 3 months previously | chelation therapy: Dimercaprol, CaNa2EDTA, DMSA (p.o.) | N/A (successful medical treatment) | N/A | N/A |
| 146 | Initial evaluation revealed a normocytic anemia, but other workup including imaging and endoscopy was unre- vealing. Other evaluations for abdominal pain were unrevealing, including ultrasound and computed tomography (CT) of the abdomen and esophagogastroduodenoscopy (EGD); Given his recent use of Ayurvedic medicines, we tested for lead poisoning | chelation therapy: DMSA (p.o.) | successful medical treatment: On day 3 of treatment, his pain improved and he was discharged home. At a 2-week follow-up he was pain-free, and his hemoglobin had increased to 12 g/dl, while his lead level was 25 mcg/dl. One year after the initial admission, he is completely asymptomatic with a lead level of 7 mcg/dl and a creatinine of 1.2 mg/dl (GFR estimate 71 ml/min/1.73 m2). | BLL: 25 µg/dl (at a 2-week follow-up), 7 µg/dl (one year after initial admission) | 0.34 |
| 147 | At the beginning of March he underwert Esophagogastroduodenoscopy which was negative; The only finding in the chest and abdomi- nal CT was hepatic steatosis. At the end of April, the patient underwent haematological counselling for microcytic anaemia associated with hyperfer- ritinemia and reticulocytosis. A screening for lead poisoning showed a blood lead value of 102 μg/dL. | chelation therapy: CaNa2EDTA (i.v.) | successful medical treatment | BLL: 36,27 μg/dl (at discharge) | 1.75 |
| 148 | CT Abdomen pelvis was normal and endoscopy suggestive of mild antral gastritis for which she was initiated on proton pump inhibitors; Biochemical tests including renal and liver function tests were normal.; MRI brain showed symmetric basal ganglia and cortical signal changes and edema with significantly elevated lead levels in blood; MRI Brain study suggestive of multiple T2 FLAIR hyperintensities in putamen, thalami and subcortical white matter with gyral edema. DWI revealed subtle diffusion restriction in left occipital lobe. CSF study revealed 5 cells (mononuclear) with proteins of 15 mg% and normal sugars. CSF cultures were negative. EEG electroencephalogram revealed mild slowing of background. | chelation therapy: D-penicillamine (p.o.) | complete clinical and radiological resolution; Repeat MRI Brain at 3 months showed complete resolution of signal changes previously seen; Clinically patient had a normal neurological examination at follow-up | N/A | N/A |
| 149 | Stool examination, upper gastrointes- tinal endoscopy, ultrasound scan of abdomen | chelation therapy: D-penicillamine | successful medical treatment | BLL: <10 µg/dl (after 6 months of treatment) | <0.48 |
| 150 | However, in the last year, he had been taking an illegally produced folk drug. A biopsy specimen of the tumor confirmed the diagnosis as verrucous see (Figure 2). The patient was referred for reconstructive surgery but died of acute pulmonary embolism a week after hospitalization. Later, the illegal drug he had taken for almost a year was analyzed and found to contain arsenic at 0.052 mg/capsule. We speculate that his tumor was highly associated with daily inorganic arsenic ingestion, which strongly illustrates the impact of unconventional therapy on the pathogenesis of cutaneous carcinoma in high-risk individuals.; Ultrasound examination revealed right inguinal lymphadenectasis. A computed tomographic scan showed multiple arteriosclerosis plaques of the lower limbs. Magnetic resonance imaging showed bilateral bone infarction in the upper end of the tibia. | no (patient died) | The patient died of acute pulmonary embolism only aweek after hospitalization, when his tumor was pathologically confirmed as verrucous carcinoma. The causative relevance of the tumor with his daily arsenic intake is discussed. | patient died | / |
| 151 | perianal gangrene -> He was treated with wound debridement and colostomy; The initial nerve conduction study and electromyography disclosed partial conduction block, prolonged F-response of motor nerve, and reduced amplitude of sensory nerve. Drug eruption and acute inflammatory demyelinating polyneuropathy were suspected on day 14. The numbness and limb weakness worsened despite treatment with antihistamine, topical steroid, vitamin B12, and 6 sessions of plasmapheresis.; On the basis of the progressive numbness and tingling pain of the extremities, weakness, muscle atrophy, hair loss, and anemia, metal intoxication and peripheral polyneuropathy were suspected and the patient was transferred to the medicine service. | chelation therapy: DMPS | most of the patient’s symptoms resolved with chelation therapy except the wound and peripheral neuropathy; He was clinically stable and discharged on day 46. At the 4-year follow-up, polyneuropathy had partially resolved, and nerve conduction studies showed no response on bilateral peroneal and sural nerves.; partial improvement, but peripheral neuropathy persists 4 years later (polyneuropathy had partially resolved) | N/A | N/A |
| 152 | Panendoscopy showed reflux esophagitis, and he was discharged with that diagnosis. Heavy metal screen subsequently showed a blood lead level of … | chelation therapy: CaNa2EDTA, DMSA | clinical recovery | N/A | N/A |
| 153 | Laboratory tests -> high BLL | chelation therapy: DMSA | successful medical treatment; the haemoglobin improved from 94 g/l to 120 g/l at day 31 as did his symptoms | BLL: 1,9 µmol/L (after 29 days) | 1.88 |
| 154 | Neuroimaging and electroencephalograph were normal; Ultrasound of the kidneys revealed enlarged kidneys. Renal biopsy [Figure 1] revealed normal‐looking glomeruli, tubules showed moderate neutrophilic infiltration, dilation, and cellular casts comprising neutrophils and tubular epithelial cells. There was no evidence of crystals, viral inclusions, or rupture of tubular basement membrane. Interstitium revealed edema along with mixed infiltration with neutrophils, lymphocytes, and occasional eosinophils, without any evidence of granulomas or fungal elements. Histopathology was suggestive of acute tubulointerstitial nephritis. | chelation therapy: D-penicillamine; dialysis, oral steroids | successful medical treatment; On follow‑up, the urine mercury levels were within normal levels. She is now normotensive without medications, has normal creatinine levels (0.7 mg/dL), eGFR‑73.8 mL/min, and no proteinuria. On 6‑month follow‑up, she had begun talking, was friendly, and had started attending play school | on follow-up, the urine mercury levels were within normal levels | / |
| 155 | CT scan of the abdomen and pelvis, upper endoscopy and colonoscopy revealed no etiology for his anemia at that hospital; After the family suggested the Ayurvedic “Bhasma” medication could be the cause, it was analyzed for heavy metals | chelation therapy: Dimercaprol (i.m.), CaNa2EDTA (i.v.), DMSA (p.o.) | successful medical treatment, but took a long time | BLL: 27,1 µg/dl (after 2 days of chelation with BAL & EDTA), 25,5 µg/dl (on discharge, after 6 days), 23 µg/dl (after 19 day course of DMSA), 38 µg/dl (over the next 6 weeks -> additional chelation therapy), 20 µg/dl (after second course of oral DMSA), 29 µg/dl (6 months after initial diagnosis -> third oral chelation therapy), 7 µg/dl (after third course of chelation with DMSA) | 0.34 |
| 156 | Patient presented to the emergency department (ED) with a history of progressively worsening of skin con- dition, gastrointestinal upset for 10 days and fever, general- ized edema and shortness of breath for 3 days. Eighteen days prior to this ED visit, he was prescribed oral herbal medications (Table 1) and topical herbal ointments for his skin disease by a licensed TCM practitioner. The patient developed diminished appetite, dizziness, abdomen discomfort, itching rash, and scaling of his skin 7 days after starting TCM treatment. However, the topical use of herbs was recommended to be continued. On Day 15, the oral TCM prescription was changed to treat his edema. He presented to the TCM clinic for help on Day 18, and received acupuncture and bloodletting therapy. The patient was taken to the ED at midnight when his shortness of breath became worse. The patient was admitted to intensive care unit with a presumptive diagnosis of toxic epidermal necrolysis (TEN), complicated with soft-tissue infection and sepsis. | patient died | toxic epidermal necrolysis complicated with soft tissue infection and sepsis was noted and he later died of septic shock and multiple organ failure (direct cause of death was Pseudomonas aeruginosa bacteremia with septic shock and multiple organ failure) | patient died | / |
| 157 | A gastroscopy showed esophagitis and the patient was discharged with a proton pump inhibitor treatment. He consulted two days later complaining of persistent symptoms. Examination revealed pain on palpation of the right upper abdominal quadrant with no sign of peritonism. Blood tests revealed a cholestatic liver abnormality and a hemolytic anemia. An abdominal ultra- sound and a thoraco-abdominal CT scan proved normal. Serologies for HIV, cytomegalovirus, Epstein-Barr virus and hepatitis A, B, C were negative. A blood smear was performed and showed baso- philic stippling that was highly evocative of a heavy metal poison- ing. | chelation therapy: DMSA (p.o.) | successful medical treatment; The symptoms rapidly improved and the patient was discharged 5 days later from hospital with outpatient control by his general physician | N/A | N/A |
| 158 | N/A; With the history of arsenical Chinese herbal medicine and palmoplantar involvement, a diagnosis of SCC arising from lesions of arsenical keratosis was made; Chest and abdomen CT were performed, and internal malignancies were not found during the evaluation; 2 separate biopsies were done; one from the ulcerative nodule and the other from the surrounding hyperkeratotic plaque; histopathological examination of the ulcerative lesion revealed SCC and the biopsy of the hyperkeratotic plaque showed hyperkeratosis with keratinocytic atypia | no cheation therapy, discontinue medication; SCC was surgically excised | N/A | N/A | / |
| 159 | Her abdominal X-ray showed a dilated colonic loop; After the delivery, her bowel habits changed, with alternating constipation and di- arrhea. Consequently, she had taken over the counter medicine intermit- tently. To rule out gastrointestinal obstruction, we ordered abdominopelvic computed tomogra- phy, which showed no intraluminal lesion causing bowel obstruction. She was admitted for supportive care of ileus. Laboratory tests; The Coombs test was negative. A diagnosis of Coombs-negative hemolytic anemia was made, and hemoglobin electro- phoresis was ordered to work up hemoglobinopathies. During the evaluation, the abdominal pain resolved spontaneously and the anemia improved to 9.7 g/dL without transfusion. She wanted to be discharged without further evaluation or management. We planned to see her as an outpatient 2 weeks later, but she was lost to follow-up. Two months later, she revisited the emergency room with the same symptoms and hemolytic anemia. The previously ordered hemoglobin electrophoresis re- vealed a normal pattern. We could not identify the cause of the hemolytic anemia, so we reviewed the previous peripheral smears thoroughly and found basophilic stippling on the red blood cells.  We considered lead poisoning as a cause of hemo- lytic anemia and the other symptoms and checked the blood lead level. No cause of lead exposure was detected. On repeated questioning, she admitted taking an  herbal medicine. | chelation therapy: CaNa2EDTA (i.v.) & D-penicillamine (p.o.) | successful medical treatment; Her hemoglobin level and abdominal pain improved after this intervention. | She has remained stable, with a hemoglobin level of 12.3 g/dL and lead level of 21.0 μg/ dL at follow-up. | 1.01 |
| 160 | N/A | N/A | N/A | N/A | N/A |
| 161 | N/A; MRI brain showed diffuse cerebral edema with basal ganglia signal changes with elevated lead levels in blood; MRI Brain suggestive of diffuse cerebral edema, gyral swelling with basal ganglia signal changes. CSF Study revealed cell count of 4 cells with normal protein and sugar. Meningitis biofire and aerobic cultures were negative. | various treatments, no chelation therapy (patient died) | death (patient became comatose and succumbed to the illness; rapid deterioration in sensorium leading to coma and death) | patient died | patient died |
| 162 | blood tests, neurologic evaluation, … -> Based on these findings, the neurologist then obtained heavy metal testing that showed elevated blood lead and arsenic concentrations ; Biomarker studies including cytogenetics, fluorescence in situ hybridization, and an extensive molecular gene mutation panel showed no evidence of any clonal myeloid processes. There was also no evidence of plasma cell lymphoma, lymphoproliferative disorder, or infiltrative marrow processes. | no chelation therapy, discontinue medication, no further treatment (conservative therapy) | At 135 days follow-up, the patient reported that her weakness had improved but her distal extremity sensation remained diminished and unchanged | N/A | / |
| 163 | X-ray abdomen (supine and erect) showed grossly dilated colon loaded with fecal matter. The contrast enhanced computed tomography of the abdomen also reported colonic dilatation. Colonoscopy was planned but could not be carried out, as the bowel could not be prepared despite enema and laxatives for 2 days. On repeated questioning the patient revealed that he was taking ‘Ayurvedic’ drugs | chelation therapy: D-penicillamine | successful medical treatment: The patient was relieved of his symptoms after 2 days. He was discharged after 7 days and was followed up in the outpatient department. | BLL: 89 µg/dl (at the time of discharge), 45 µg/dl (after 2 weeks of therapy), 32 µg/dl (after 3 weeks of therapy) | 1.55 |
| 164 | N/A | chelation therapy: DMSA (p.o.); iron supplementation | successful medical treatment, symptoms improved over the subsequent 3 months | BLL: 13 µg/dl (after therapy); haemoglobin increased to 99 g/L | 0.63 |
| 165 | Mehrere positiv ausfallende Tests wie z.B. Abdominal ultrasound revealed hepatomegaly with normal echotexture and choleli- thiasis; he had also developed significant anemia unresponsive to hematinics and was requiring blood transfusions; dann: The blood, urine, hair, and nails were tested for arsenic which was elevated; Abdominal ultrasound revealed hepatomegaly with normal echotexture and cholelithiasis | chelation therapy: D-penicillamine (second choice, DMSA was not available) | successful medical treatment, but neuropathy only partially improved; Hematological parameters improved within 2 weeks with normalization of blood counts. Hyperkeratosis of the palms and soles resolved in the next 4 months. However, neuropathy only partially improved. Blood As levels normalized after 6 months. She has been now followed up for more than a year and is doing well. | blood As levels normalized after 6 months (blood 18,9 µg/l, urine 0,9 µg/l) | / |
| 166 | physical examination, nerve conduction etc. -> As levels in the blood and hair were elevated | chelation therapy: D-penicillamine | clinical improvement, but residual neuropathy; Blood As levels became normal at 6 months and were normal till 1 year when last estimated, and he improved clinically, though had residual neuropathy. | blood As levels became normal at 6 months and were normal till 1 year when last estimated (not further specified) | / |
| 167 | Computerized tomography of the chest revealed bilateral bronchiectasis, and ultrasound of the abdomen showed normal liver echotexture, splenomegaly, and dilated portal vein suggestive of non-cirrhotic portal hypertension (NCPF). Liver biopsy was done which was inconclusive. She was suspected with chronic As toxicity following ayurvedic medication as no other apparent source of it was there. | chelation therapy: D-penicillamine | successful medical treatment (cutaneous manifestations improved, blood As levels became normal after 6 months); she lived well for 2 year, after which, she succumbed to severe bronchopneumonia and sepsis | blood As levels became normal after 6 months (not further specified) | / |
| 168 | Several complementary examinations were performed (chest X-ray, fecal occult blood, upper and lower gastrointestinal endoscopy, bone marrow aspirate, ultrasound and abdominal computed tomography), which showed no significant abnormalities | chelation therapy: CaNa2EDTA (i.v.) & DMSA (p.o.) | successful medical treatment | treatment until plumbemia of 13 µg/dl was achieved with normalization of porphyrins and disappearance of clinical manifestations | 0.63 |
| 169 | She was warned by the health authorities of a risk of saturnism following the diagnosis of case 1, and underwent toxicological analyses that showed a high blood lead concentration | chelation therapy: DMSA (p.o.) | successful medical treatment | At the end of the treatment she was asymptomatic, blood lead was 14 µg/dl and erythrocyte protoporphyrin was 59 mg/dl. | 0.68 |
| 170 | An upper and lower gastrointestinal endoscopy to detect anemia due to gastrointestinal bleeding was not performed because there were no findings that could be the cause of anemia in the endoscopy performed for medical examination one month earlier. Abdominal computed tomography scan showed no abnormal findings other than mild fatty liver; -> based on the patient's history of taking Chinese medicine, it was assumed that there was a possibility of heavy metal poisoning caused by Chinese medicine -> diagnosis didn't take much time; Coomb's test to rule out hemolytic anemia due to liver disease as a cause of anemia was negative | no chelation therapy, discontinue medication, no further treatment (conservative therapy) | successful treatment | last confirmed lead level was 63.8 µg/dL -> much higher than the World Health Organization standard, and considering that the half-life of lead is 5-10 years, continuous follow-up is necessary, so it is planned to follow-up the blood lead level every 1 year. | 3.08 |
| 171 | At his 18th month, well-child check routine screening laboratory tests were again ordered. Quite surprisingly this time, his capillary lead level was found to be 54.0 μg/dL. | chelation therapy: DMSA (p.o.) | successful medical treatment | BLL: 18,4 µg/dl (at the end of chelation therapy) | 0.89 |
| 172 | There was a protracted delay in the diagnosis of lead poisoning. Most parents had observed and become concerned about the variety, and worsening, of symptoms in their ailing children around three to four weeks after commencing ingestion of the contaminated batch of Ayurvedic medicines. Despite this, over a period of six months and consulting general practitioners and a variety of medical specialists, including neurologists, orthopedic surgeons, pediatricians, hematologists, psychologists, cardiologists, endocrinologists, and gastroenterologists, and visiting emergency departments when symptoms were particularly severe, lead poisoning was never considered and no instruction for a diagnostic blood lead test was ever given in respect of any of the study subjects, even after being informed about the ingestion of an Ayurvedic treatment. Instead it was the fortuitous observation by a laboratory pathologist of basophilic stippling on a blood smear from one of the subjects that first raised the prospect of lead poisoning as an explanation for the range of generalized symptoms exhibited. Re-examination of a blood sample from a second subject, and the similar observation of blood stippling, strengthened the suspicion of a lead poisoning outbreak, leading to calls for blood lead tests in all subjects. By this time, the participants had been advised to have, or endured, a wide range of unnecessary, often invasive, diagnostic examinations, when a simple blood lead test would have sufficed. These included blood sampling, gastroscopy, colonoscopy, CT scanning, MRIs, thyroid functioning and other endocrinology testing, X-Rays, and ultrasound examinations. Prior to the conclusive diagnosis of lead poisoning, a range of diagnoses and explanations for the symptoms were offered by doctors, including depression, “burnout,” menstrual cramps, influenza, and “growing pains.” | chelation therapy: DMSA | current health status: ankylosing spondylitis, polycystic ovaries, back, knee and hip aches | venous BLL 2 years later (2014): 12,5 µg/dl | 0.6 |
| 173 | " | chelation therapy: DMSA | current health status: tiredness, headaches, nausea, mood swings, occasional vomiting | venous BLL 2 years later (2014): 24,2 µg/dl | 1.17 |
| 174 | " | chelation therapy: DMSA | current health status: headaches, stomage pains | venous BLL 2 years later (2014): 17,7 µg/dl | 0.86 |
| 175 | " | chelation therapy: DMSA | current health status: knee and back aches | venous BLL 2 years later (2014): 28,4 µg/dl | 1.37 |
| 176 | " | chelation therapy: DMSA | current health status: feels "fine" | venous BLL 2 years later (2014): 34,0 µg/dl | 1.64 |
| 177 | " | chelation therapy: D-penicillamine, DMSA (initially prescribed Penicillamine until DMSA became available) | current health status: constipation | venous BLL 2 years later (2014): 17,7 µg/dl | 0.86 |
| 178 | " | chelation therapy: DMSA | current health status: feels "good", but occasional tiredness & abdominal pains | venous BLL 2 years later (2014): 27,4 µg/dl | 1.32 |
| 179 | " | chelation therapy: DMSA | current health status: well | venous BLL 2 years later (2014): 10,4 µg/dl | 0.5 |
| 180 | Due to the previously confirmed acral-site SCCs and the history of long-term use of a TM antecedent to the lesions, chronic arsenic toxicity was suspected. On examination, palmar hyperkeratosis and a ‘raindrop’ pigmentation pattern appeared to be present on many skin areas. | no cheation therapy, discontinue medication; SCCs were surgically excised | The patient remained well for 15 months. His chronic plaque psoriasis was well controlled with a combination of topical corticosteroid-vitamin D analogue ointment and emollients. Due to his multiple SCCs, we advised that phototherapy for psoriasis must be avoided. He subsequently did develop an ulcerated nodular lesion on his right upper chest area. A complete excisional biopsy of the lesion showed the presence of both SCC and basal cell carcinoma (BCC). | N/A | / |
| 181 | N/A | chelation therapy: DMSA (p.o.) | successful medical treatment: patient discontinued Ayurvedic medication use and reported improvement in symptoms after 1 month of chelation therapy | N/A | N/A |
| 182 | The patient described no regular medication use except vitamin supplementation. There was no history of allergy and no tobacco or alcohol consumption. The patient reported regu- lar personal business travel to Thailand and India and a family history of diabetes mellitus and rheumatoid arthritis. (...) Given the symptomatology, biochemical picture and prominent basophilic stippling, heavy metal poisoning with lead was suspected. On further questioning, the patient revealed the ‘vitamins’ he was consuming were Ayurvedic products purchased on  his business trips to India. | chelation therapy: DMSA (p.o.) | successful medical treatment: The patient’s systemic lead toxicity, manifest as transient encephalopathy, lethargy, abdominal symptoms, jaundice, renal tubular dysfunction and anaemia, resolved rapidly with cessation of the Ayurvedic medication | N/A | N/A |
| 183 | Peripheral smear revealed no schistocytes or malarial parasites and serology was negative for leptospirosis and dengue. Ultrasound of the abdomen revealed enlarged kidneys. On questioning, she revealed regular consumption of an ayurvedic medication.; -> case of short term ingestion of ayurvedic medication along with adjuvant chemotherapy in a breast cancer patient resulting in non‐oliguric acute renal failure | intravenous hydration with normal saline at 150 ml/h along with antiemetics | Her sensorium improved within 12 h, and serum creatinine showed a falling trend in 48 h and it normalized in 1‐month. | Mercury levels in 24 h urine sample in our patient performed 2 months after the episode were within normal limits (1.3 μg/dL) as were serum lead levels (10.3 μg/dL). | N/A |
| 184 | N/A; abdomen CT: diffuse enlargement and ascites formed in liver | chelation therapy: DMPS | After two courses of removing arsenic treatment, the pruritus was eased. Desquamation appeared on hands and no verrucous hyperplasia was observed on new skin. Abdomen B-ultrasound showed that dropsy disappeared. | After the first course, the urine arsenic was re-examined as 686 μg/L and after the second one, it was 76 μg/L. Because of personal reason, the third course was not conducted. Blood routine examination showed that blood platelet was 108×10^9/L and liver function showed that ALP 60 U/L and GGT 102 U/L, and all indicators were improving. The patient is now in follow-up. | / |
| 185 | He denied any significant medical history and had never had surgery. Imaging included a CT scan and an abdominal ultrasound, neither of which revealed any abnormalities. Given his blood-streaked emesis and anaemia, and in absence of another diagnosis to explain his severe abdominal pain and weakness, he was treated with an intraven- ous proton pump inhibitor. An upper endoscopy performed the following day was unrevealing. He was administered morphine and ibuprofen as needed. He was discharged after a 2-day hospital stay, on morphine as needed for pain, and on omeprazole and ondansetron, for nausea and vomiting. He was instructed to follow-up with a haematologist, gastroenterologist and primary care physician. On discharge, the patient was advised to discontinue use of his four different types of Ayurvedic medications, which he did, as it was felt that they might be contribut- ing to his symptoms. Further work up as an outpatient included iron studies, vitamin B12, folate and thyroid-stimulating hormone, all of which returned within normal limits. | chelation therapy: DMSA | successful medical treatment (symptoms resolved after 3 months of treatment) | BLL: see article (many BLL-measurements!!!); 16,4 µg/dl (123 weeks since first BLL); This patient’s BLL did not decrease linearly, but, rather, rose and fell while overall trending downwards with the chelation therapy; For example, between days 139 and 181, after being treated with the third round of succimer, his blood lead levels increased from 8.7 mg/dL to 25 mg/dL; Overall, this patient’s BLL trended down over time | 0.79 |
| 186 | Anteroposterior abdominal radiography revealed radiopaque point masses throughout the abdomen. No evidence of obstruction or pneumoperitoneum was reported. An abdominal ultrasound scan revealed a hypoechoic nodule within the abdominal cavity, possibly accounted for by superior mesenteric lymph nodes. Three days prior to his presentation to the hospital, he visited a local emergency department. A diagnosis of drug hepatitis was made to account for the elevated liver transaminases. Mesenteric lymphadenitis was also considered as indicated by the abdominal ultrasound result but was not confirmed by the severely damaged liver function. Cefuroxime, creatine phosphate, cimetidine, vitamin B6, and racanisodamine hydrochloride (an antispasmodic) were administered. During 3 days of therapy, his liver function improved (Table 1), but was still far from normal. The abdominal pain increased. He was admitted to the hospital for further evaluation. It was suspected that his disease could be related to the folk remedy he had been prescribed | chelation therapy: CaNa2EDTA (i.v.); folium sennae, antioxidant glutathione | successful medical treatment, symptoms resolved after 2 days of chelation; a repeat abdominal X-ray in the hospital confirmed minimal residual intestinal radiopaque material; Initially high urinary lead concentrations declined markedly by the end of chelation, but they were still higher than spontaneous lead excretion post-chelation. The liver enzyme concentrations continued to improve, and were nearly normal by after hospital discharge for 5 days (aspartate aminotransferase 42U/l, alanine aminotransferase 48U/l). Anemia still existed after the first course was completed, but the hemoglobin concentration improved to 111 g/l within two weeks. | BLL & ULL: see article (many BLL- & ULL-measurements); BLL on day 6 after admission (during first chelation theapy): 23,6 µg/dl, BLL on day 12 after admission: 36,8 µg/dl; ULL on day 12: 2.364 µg/dl; the BLL declined steadily with the evidence of the beginning of a rebound 1 week after chelation. | 1.78 |
| 187 | There was history of constipation but no abdominal distension, fever or altered sensorium. There was no history of pica or environmental exposure to chemicals. He was operated for ileoileal intussusception elsewhere but pain abdomen persisted. Lead toxicity was suspected in view of ayurvedic medication use. | no chelation therapy, discontinue medication, no further treatment (conservative therapy) | successful treatment | BLL: 20 µg/dl (day 9 of hospitalization), 2 µg/dl (3 months later) | 0.1 |
| 188 | systemic examination was unremarkable; other investigations, including abdominal ultrasound, were essentially normal | chelation therapy: Dimercaprol (i.m., DMSA & CaNa2EDTA were not available) | successful medical treatment; Burtonian line disappeared and other symptoms alleviated | BLL: 40 µg/dl (post-treatment) | 1.93 |
| 189 | The patient’s reported use of herbal medications prompted testing for heavy metals, including arsenic. | no chelation therapy, discontinue medication, no further treatment (conservative therapy) | successful treatment | arsenic level decreased to the normal range (6 months after stopping use) | / |
| 190 | died within few days after using a local folk prescription ointment made by a quack doctor; His relatives want to find the cause of the death, so autopsy was performed; autopsy showed multiple punctate hemorrhages over the limbs, pleural effusion, edematous lungs with consolidation, mild myocardial hypertrophy and normal-looking kidneys; histopathological examination of renal tissue showed severe degeneration, necrosis and desquamation of renal tubular epithelial cells, presence of protein cast and a widened edematous interstitium with interstitial fibrosis; histopathological examinations: see article | various treatments, no chelation therapy (patient died) | patient died 4 days after hospital admission (the cause of death of the man was multiple organ dysfunction caused by acute arsenic poisoning) | patient died; in the local folk prescription ointment, arsenic was at a concentration of 6.01x10^4 mg/mL (about 6%), in heart blood: 1.76 mg/mL (1.76x10-3 mg/L), in the corium layer of the affected skin (back, chest and right leg): 4.71 mg/g, 4.23 mg/g and 5.68 mg/g; none of other heavy metal elements, such as Hg, Cu, Mn, Cd, Pb and Ba, was detected | / |
| 191 | To enquiry, he admitted taking Ayur- vedic medicine over a long but unquantifiable duration. Nerve conduction studies revealed axonal sensorimotor polyradiculoneuropathy. Other investigations included magnetic resonance imaging of brain, cerebral spinal fluid analysis, autoimmune, infection and metabolic screens, and were all unremarkable.; Laboratory tests -> At this point, a provisional diagnosis of heavy metal poisoning was considered, based upon his history of Ayurvedic medicine exposure. | chelation therapy: Dimercaprol (i.m., initial unavailability of CaNa2EDTA & DMPS), CaNa2EDTA, DMPS; extracorporeal blood purification | successful medical treatment; after first chelation therapy: his nightmares, involuntary vocalisation and gastrointestinal symptoms resolved over a few weeks, although his peripheral neuropathy and alopecia persisted with only mild improvement | BLL: <1,2 umol/l (after 8 weeks); over the following 8 months of follow up, there was only mild improvement of his tremors and weakness, and the peripheral neuropathy persisted, presumably on the basis of chronic arsenicosis | 1.21 |
| 192 | Various tests were performed, but no cause of severe periumbilical abdominal pain was found (abdominal computed tomography, ultrasonography, upper gastrointestinal endoscopy, and colonoscopy). After several interviews, the patient revealed that he had been taking traditional herbal medicine for about half a year. | chelation therapy: DMSA | successful medical treatment | BLL: 75,2 µg/dl (after 1 month) | 3.63 |
| 193 | N/A | no chelation therapy, discontinue medication; multivitamin capsules, green tea & garlic (conservative therapy) | successful medical treatment; The patient's tongue tingling and systemic symptoms improved within 4 months, but her anxiety, depression, and weakness persisted. Brain magnetic resonance imaging performed at our Neurology Department was normal except for mild chronic small vessel ischemic disease. Six months after discontinuing S. capsule intake, the patient remains under treatment for depression and anxiety in the Psychiatry Department, and for dysesthesia in the Neurology Department. | BLL: 23,76 µg/dl (~1 month later); it returned to the normal range (8.64 μg/dL) after 120 days | 0.42 |
| 194 | N/A | chelation therapy: not further specified | successful medical treatment; Slowly occurring clinical recovery after starting chelation therapy corroborated with the causal assumption proposed. He was released for further consultancy to his family physician. The administrated treatment and the improvement of his status corroborate lead and mercury intoxication. | N/A | N/A |
| 195 | gastroscopy and colonoscopy; abdominal CTA; right ileocolic artery stenosis was highly suspected; Then, the patient received treatment for ischemic bowel disease and no improvement in his symptoms was reported; Gastroscopy, colonoscopy and abdominal CTA were repeated and yet again produced normal; Magnetic resonance enterography showed parts of the small bowel walls thickening in the left upper abdomen (Figure B). Double-balloon endoscopy revealed patchy redness and congestion at two sites between 50 cm and 150cm from the pylorus. Some time after the patient was admitted, his symptoms deteriorated so much so that he attempted suicide. | chelation therapy: not further specified | successful medical treatment: three days after chelation treatment, his symptoms disappeared and did not recur in the follow-up. | N/A | N/A |
| 196 | The possibility of Guillain–Barre syndrome and porphyria was considered and investigated accordingly, but was in vain. | no chelation therapy, discontinue medication; potassium chloride i.v., multidose of activated charcoal & multivitamin supplements, hemodialysis | successful medical treatment, hair started growing after about 2 months | N/A | / |
| 197 | Abdominal radio- graphs and abdominal CT scan revealed no abnormalities. Liver echo, tumor marker, and viral hepatitis screening test results were negative. The patient was admitted to the internal medicine ward with a diagnosis of NSAP. The diagnosis of NSAP was made based on the clinical features and negative examination results. After admission, we performed a urine porphyrin test based on his acute porphyria-like symptoms. Acute intermittent porphyria was diagnosed based on a positive urine porphyrin test result, and hemin therapy was started. However, in spite of the hemin therapy, the pain did not improve, indicating a misdiagnosis. We performed a detailed medical history taking again. The patient reported visiting a local Chinese medicine clinic. We present a case of a 48-year-old man who had recurring abdominal pain with anemia that was misdiagnosed. His condition was initially diagnosed as nonspecific abdominal pain and acute porphyria. Acute porphyria-like symptoms with a positive urine porphyrin test result led to the misdiagnosis; testing for heme precursors in urine is the key to the differential diagnosis between LP and acute porphyria. The final definitive diagnosis of lead toxicity was confirmed based on high blood lead levels after detailed medical history taking. | chelation therapy: CaNa2EDTA (i.v.) | successful medical treatment: The clinical symptoms gradually improved. His blood lead level decreased to 31 𝜇g/dL, and his hemoglobin level and liver function had returned to normal levels when he was reevaluated 2 weeks after discharge. No relapse was observed thereafter and during the 1-year follow-up. | BLL: 31 µg/dl (2 weeks after discharge) | 1.5 |
| 198 | He was advised USG abdomen which reported mild prostatic enlargement. The patient underwent upper gastro intestinal endoscopy for recurrent vomiting which did not respond to conser- vative management. The report was suggestive of hiatus hernia and esophagitis. Further CT angiography was planned due to persistence of vomiting. The report was suggestive of atheromatous changes in abdominal aorta and its branches with mild luminal narrowing at origin of celiac artery, fatty liver and hemangioma in right lobe of liver with mild prostatic enlargement. The patient was started on conservative management. The herbal medicine being taken by the patient was sent for heavy metal analysis. | chelation therapy: D-penicillamine | successful medical treatment: symptoms & BLL have improved gradually | BLL: 54 µg/dl (11 days after admission), …, 17,3 µg/dl (about 7 months later) (more BLL's: see article) | 0.84 |
| 199 | Ini- tially, he presented to his diabetologist with the above symptoms, who – concerned that the patient may be developing pancreatitis as a side-effect of the Saxagliptin – requested a set of blood tests, including a full blood count, urea and electrolytes and lipase.; On further questioning, it was revealed ... | chelation therapy: DMSA (p.o.) | successful medical treatment: the patient’s symptoms and blood abnormalities resolved; He underwent serial blood tests to monitor his lead levels during treatment, which showed a good response, with haemoglobin increasing to 107 g/L and the lead level dropping to 1.4 μmol/L | BLL: 1,4 µmol/l (after treatment) | 1.4 |
| 200 | On physical examination, the patient’s vital signs, neurological examination, and chest examination were normal. (…); Tests for hepatitis B and autoimmune diseases were negative. Abdominal ultrasound and computed tomography (CT) scan located a liver cyst that was approximately 1.3 cm × 1.0 cm. Gastroscopy showed a fundic gland polyp and chronic non-atrophic gastritis; a colonoscopy revealed nothing except internal hemorrhoids. After supportive therapy for about 5 days, the patient’s symptoms had not remitted. We then revisited the patient’s detailed medication history and found that he had been taking homemade TCM; Electromyography showed distal multiple peripheral nerve damage in accordance with typical neurological damage owing to lead poisoning. | chelation therapy: not further specified | successful medical treatment: His periumbilical pain was noticeably relieved after treatment and the blood lead level returned to normal after three treatment rounds. | N/A | normal |
| 201 | Wife of patient 1 | no chelation therapy, discontinue medication, no further treatment (conservative therapy) | N/A; As she was asymptomatic and showed only a slightly elevated blood lead level, his wife did not receive chelation therapy | N/A | N/A |
| 202 | On routine screening at her 9-month well-child check, an infant girl born in the USA to a Malaysian father and Taiwanese mother had an elevated capillary blood lead level. A com- prehensive history, including detailed environmental and developmental histories, and physical examina- tion were conducted. At her initial consultation visit, the infant’s parents reported that their daughter was in good health without any concerning symptoms. Her birth and past medical histories were unremarkable without any complica- tions during pregnancy or delivery and no major illnesses, hospitalizations, or surgeries. She had received routine well-child care and had met all major developmental milestones. The initial environmental history did not reveal an obvious source of lead exposure in this non-ambula- tory patient. The family had lived in two residences since their daughter was born. Both homes were built long after lead-based paint was phased out of use in the USA. The family had moved into a newly constructed condominium 4 months prior to presentation, and their previous residence was an apartment in a complex built in the early 2000s.  A detailed exposure history excluded numerous sources of lead exposure (Table 1) but uncovered one particularly suspicious practice. Since birth, the infant’s parents had been applying a non-commercial diaper powder fabricated in a Malaysian corner store/ pharmacy. | no chelation therapy, discontinue medication, no further treatment (conservative therapy) | successful treatment | BLL: 8 µg/dl (4 weeks after discontinuing use of the powder) | 0.39 |
| 203 | CT scan of the abdomen revealed hepatomegaly with steatosis, patent hepatic vasculature and normal biliary anatomy without evidence of portal hypertension.; Liver histopathology revealed extensive periportal and perivenular necrosis (figure 2A). The portal tracts showed infiltration by neutrophils, lymphocytes and eosinophils (figure 2B) associated with marked cholangitis, hepatocytic ballooning, neutrophilic satellitosis (figure 2C, D) and severe canalicular and hepatocellular cholestasis (figure 2E). Masson Trichrome staining revealed extensive bridging fibrosis (figure 2F). | various treatments, no chelation therapy (patient died) | patient died | patient died | patient died |
| 204 | N/A | N/A | N/A | N/A | N/A |
| 205 | The pathogenesis of anemia was not found according to serum iron test, hemoglobin elec- trophoresis, G6PD activity detection, etc.; Bone marrow smears; diagnosis found quickly | chelation therapy: DMSA | successful medical treatment | BLL: 384,2 µg/l (after chelation therapy) | 1.86 |
| 206 | / (son was diagnosed with lead poisoning before) | chelation therapy: not further specified | successful medical treatment (discharged with a better condition 3 weeks later) | N/A | N/A |
| 207 | Two weeks before he came to our hospital, he went to another local hospital, where a colonoscopy and an abdominal ultrasound were performed and nothing abnormal was observed. The abdominal X-ray indicated irritable bowel syndrome. He was diagnosed with incomplete intestinal obstruction and was discharged after symptomatic treatment. However the symptoms persisted, so the patient was referred to our hospital. Tests for autoimmune diseases showed negative results. The urine and stool tests yielded normal results. An abdominal CT showed right inferior mesenteric lymph nodes. Sacral 1 occult spina bifida was found on the scan. A gastroscopy showed polyps of the fundus gland and chronic non-atrophic gastritis. After the treatment of spasmolysis and defecation, the patient’s symptoms were not relieved. We noticed that the patient’s main clinical manifestations were abdominal pain, constipation and hemolytic anemia, so we asked the patient again about his past and recent medication history. We found that the patient was treated with oral Chinese medicine powder and Chinese medicine pills. | chelation therapy: 2 rounds, not further specified | successful medical treatment, patient recovered; His periumbilical pain and constipation were noticeably relieved after treatment and his blood lead level returned to normal after two rounds of treatment. | BLL returned to normal | returned to normal |
| 208 | The BLL test had been requested by the child’s paediatrician due to concern about the child’s recent consumption of an oral Ayurvedic medicine prescribed by a naturopath. | N/A | N/A | N/A | N/A |
| 209 | Extensive investigations revealed no significant abnormalities. On further probing, he admitted taking traditional medications for infertility. An immediate erect chest X-ray and abdominal X-ray were done which were normal. Ultrasound of the whole abdomen was suggestive of mild hepato- megaly with fatty liver. Urgent contrast enhanced CT of the abdomen with angiography was done to rule out intestinal obstruction and aortic dissection. It revealed mild hepatomegaly. Serum amylase, lipase and renal function tests were within normal limits. ECG showed normal sinus rhythm. | chelation therapy: D-penicillamine | successful medical treatment, patient was asymptomatic after 6 weeks of therapy; The patient was discharged from the hospital after 4 days and advised to continue chelation therapy along with mebeverine and chlordiazepoxide–clinidium combination (Librax) for abdominal pain. Librax helps to relieve stomach spasms, abdominal cramps and anxiety related to gastric disorders.The patient had resolution of constipation in 2 weeks. | BLL: 45 μg/dL (after 15 days), 30 μg/dL (after 6 weeks), 14 μg/dL (after 12 weeks) | 0.68 |
| 210 | / (son was diagnosed with lead poisoning before) | chelation therapy: not further specified | successful medical treatment (discharged with a better condition 3 weeks later) | N/A | N/A |

*Tab. S2F: Raw data table of all patient cases – Test until correct diagnosis, Treatment, Clinical outcome, Laboratory results after end of therapy, Last BLL measured (µmol/l)*
